# Enzymatic Synthesis of Methylated Terpene Analogues Using the Plasticity of Bacterial Terpene Synthases

**DOI:** 10.1002/chem.201905827

**Published:** 2020-01-30

**Authors:** Anwei Hou, Lukas Lauterbach, Jeroen S. Dickschat

**Affiliations:** ^1^ Kekulé-Institute of Organic Chemistry and Biochemistry University of Bonn Gerhard-Domagk-Strasse 1 53121 Bonn Germany

**Keywords:** configuration determination, enzyme catalysis, isotopes, substrate analogues, terpenoids

## Abstract

Methylated analogues of isopentenyl diphosphate were synthesised and enzymatically incorporated into methylated terpenes. A detailed stereochemical analysis of the obtained products is presented. The methylated terpene precursors were also used in conjunction with various isotopic labellings to gain insights into the mechanisms of their enzymatic formation.

## Graphical abstract

**Naturally non‐natural**: The methylated isopentenyl diphosphate (IPP) analogues (*E*)‐ and (*Z*)‐4‐methyl‐IPP were synthesised and incorporated into terpenes by enzymatic reactions with FPP (farnesyl diphosphate ) synthase and terpene synthases, yielding methylated mono‐ and sesquiterpenes. The cyclisation mechanisms towards the obtained non‐natural terpene analogues were studied using isotopically labelled natural terpene precursors and their methylated analogues.

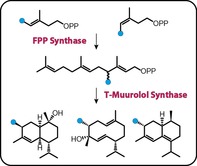

During the past decade the genome sequences of many bacteria and fungi became available, which allowed for the discovery and characterisation of various terpene synthases (TSs).^1^, ^2^, ^3^, ^4^, ^5^, ^6^, ^7^ Canonical TSs catalyse the conversion of isoprenoid diphosphates with the general formula (C_5*n*_H_8*n*+1_)OPP including dimethylallyl (DMAPP, *n*=1), geranyl (GPP, *n*=2), farnesyl (FPP, *n*=3), geranylgeranyl (GGPP, *n*=4) and geranylfarnesyl diphosphate (GFPP, *n*=5) into terpenes. For the larger precursors (*n*>1) the products are usually (poly)cyclic and contain multiple stereogenic centres. The TS‐catalysed transformations proceed through substrate ionisation by abstraction of diphosphate or by protonation, followed by a cationic cascade including cyclisation reactions, hydride or proton migrations and skeletal rearrangements. The cascade is terminated by deprotonation to yield a terpene hydrocarbon (C_5*n*_H_8*n*_) or by the addition of water resulting in a terpene alcohol (C_5*n*_H_8*n*+2_O). Deviations from these general molecular formulae may point to non‐canonical TSs that can catalyse the cyclisation of methylated terpene precursors (Scheme 1), exemplified by 2‐methylisoborneol (**1**) that is formed by a methyltransferase (MT) through *S*‐adenosyl‐l‐methionine (SAM) dependent methylation to 2‐Me‐GPP and cyclisation by a TS.^8^ For sodorifen (**3**) a MT catalyses a methylation‐induced cyclisation to presodorifen diphosphate (**2**), followed by a second TS‐dependent cyclisation.^9^ The recently investigated meroterpenoid longestin incorporates (4*R*,12*R*)‐4,12‐dimethyl‐GGPP (**4**) that is generated by methylation of isopentenyl diphosphate (IPP) to (*Z*)‐4‐methyl‐IPP, followed by a programmed skipped incorporation into the isoprenoid diphosphate chain.^10^ Based on the uptake of radioactivity from [1–^14^C]propionate into *Manduca sexta* juvenile hormone II (**5**) a biosynthetic hypothesis by the passage of propionyl‐CoA substituting for acetyl‐CoA through the mevalonate pathway was proposed.^11^ In addition to such natural enzyme systems for the biosynthesis of homoterpenes, several canonical TSs exhibit a remarkable plasticity that allows the conversion of non‐natural substrate analogues with additional Me groups or heteroatoms.^12^ This strategy can give a rapid and potentially enantioselective access towards methylated or functionalised terpene analogues, but previous work depended on the chemical synthesis of the oligoprenyl diphosphate analogues. Here we report on the stereoselective synthesis of homologated IPP derivatives, the enzymatic incorporation into methylated GPP and FPP analogues, and their TS‐catalysed conversion into non‐natural homoterpenes.

**Scheme 1:**
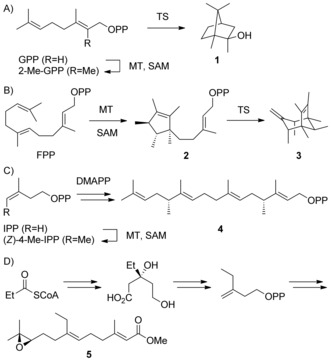
Biosynthesis of the homoterpenes A) 2‐methylisoborneol (**1**), B) sodorifen (**3**), C) (4*R*,12*R*)‐4,12‐dimethyl‐GGPP (**4**), D) juvenile hormone II (**5**).

For the enzymatic synthesis of homoterpenes, (*E*)‐4‐methyl‐IPP (**8 a**) was stereoselectively synthesised from (*E*)‐2‐bromobut‐2‐ene (**6 a**) by halogen–metal exchange with *t*BuLi and addition to oxirane to yield (*E*)‐3‐methylpent‐3‐en‐1‐ol (**7 a**) (Scheme 2 A). Conversion into the corresponding tosylate and nucleophilic substitution with (Bu_4_N)_3_HP_2_O_7_ gave **8 a**. Its stereoisomer (*Z*)‐4‐methyl‐IPP (**8 b**) was prepared via the same route starting from *Z* configured **6 b**. Subsequently, DMAPP was elongated with **8 a** and **8 b** using the FPP synthase (FPPS) from *Streptomyces coelicolor* A3(2),^13^ followed by dephosphorylation with calf intestinal phosphatase (CIP). GC/MS analysis of the products suggested that with both substrates **8 a** and **8 b** mainly only one extension step to 4‐methyl‐GPP (**9**) was catalysed, whereas only very small amounts of double extension products were observed (Figure S1, Supporting Information). A preparative‐scale incubation of DMAPP and **8 a** (100 mg each) with FPPS followed by treatment with CIP allowed to isolate the main product that was identified by NMR spectroscopy (Table S1, Figures S2–S8) as 4‐methylgeraniol (**11**). GC analysis using a chiral stationary phase revealed that the products **11** from **8 a** and **8 b** were enantiomers and were obtained in high enantiomeric purity (Figure S9). The elongation of GPP with **8 a** and **8 b** using FPPS also resulted in the two enantiomers of 4‐methyl‐FPP (**10**) with high enantiomeric purity, as demonstrated by dephosphorylation with CIP followed by GC/MS and enantioselective GC analysis (Figures S10 and S11). A preparative‐scale reaction with **8 a** and product isolation for structure elucidation by NMR (Table S2, Figures S12–S18) confirmed the structure of 4‐methylfarnesol (**12**). Its optical rotation [α]20d =−6.0 (CH_2_Cl_2_, *c* 0.25) allowed to assign the absolute configuration of (*S*)‐4‐methylfarnesol (**12 a**) for the product from **8 a** by comparison to literature data for the same enantiomer ([α]d =−10.7, hexane),^14^ and consequently (*R*)‐**12 b** was obtained from **8 b**. Based on the same sign for the optical rotation [α]20d =−5.5 (CH_2_Cl_2_, *c* 0.2) of **11 a** formed from **8 a**, and on the same GC elution order on a chiral stationary phase, analogous absolute configurations were assigned for (*S*)‐**11 a** and (*R*)‐**11 b**. These findings are in agreement with the classical experiments regarding FPP biosynthesis that demonstrated *Si* face attack at C4 of IPP (because of a change in priority orders this corresponds to the 4 *Re* face of **8 a** and the 4 *Si* face of **8 b**).^15^

**Scheme 2:**
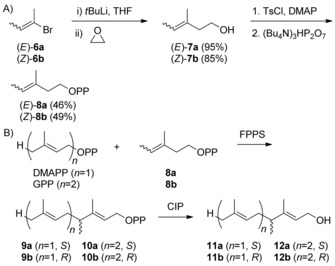
Preparation of both enantiomers of 4‐methyl‐GPP (**9 ab**) and 4‐methyl‐FPP (**10 ab**). A) Chemical synthesis of (*E*)‐ and (*Z*)‐4‐methyl‐IPP (**8 ab**). B) Enzymatic coupling of DMAPP and GPP with **8 ab** by FPPS.

In subsequent experiments bacterial terpene synthases were investigated for their potential to convert the methylated GPP and FPP analogues into methylated terpenes. For this purpose, a newly identified linalool synthase from *Chryseobacterium polytrichastri* DSM 26 899 (CpLS, accession number WP 073293738) and the known T‐muurolol synthase from *Roseiflexus castenholzii* DSM 13941 (TmS)^16^ were selected, because these enzymes showed a particularly effective conversion of their native substrates. While CpLS naturally accepts only GPP, but no FPP or GGPP, TmS shows most efficient conversion of FPP and also accepts GPP, but no GGPP. The main product obtained from GPP with purified CpLS (Figure S19, Supporting Information) was identified by GC/MS as linalool (**13**), besides minor amounts of geraniol and a few trace compounds (Figure S20). Compound **13** was obtained as a mixture of (*R*)‐ and (*S*)‐linalool (81:19, 60 % *ee*, Scheme 3 A), as determined by chiral GC in comparison to authentic (*R*)‐**13** (Figure S21). TmS converted GPP into a mixture of geraniol, (*R*)‐ and (*S*)‐**13** (66:34, 33 % *ee*), β‐myrcene and a few minor compounds (Figures S20 and S21). CpLS and TmS were used in this study to obtain the poorly described 4‐methyllinalools^17^ and to solve the difficult problem of their configurations.

**Scheme 3:**
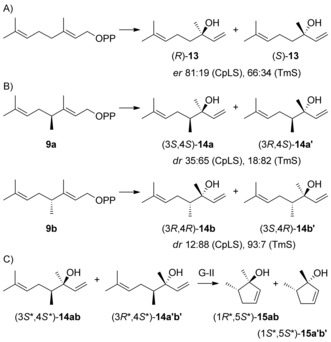
Enzyme reactions with CpLS and TmS. Conversion of A) GPP into **13**, B) enzymatically prepared **9 a** and **9 b** into diastereomeric mixtures of **14**, and C) cyclisation of **14** with Grubbs II (G‐II) catalyst.

The incubation of DMAPP and **8 a** with FPPS and CpLS gave the two stereoisomers (3*S*,4*S*)‐**14 a** and (3*R*,4*S*)‐**14 a’** (*dr* 35:65), while the same reaction with **8 b** yielded (3*R*,4*R*)‐**14 b** and (3*S*,4*R*)‐**14 b’** (*dr* 12:88) (Scheme 3 B). Their configurations at C4 were inferred from the stereostructures of **9 a** and **9 b**, but it could not be assigned at this stage which stereoisomer is major and which one is minor, because for the acyclic compounds it was not possible to determine the relative configuration at C3 by NOESY. For full structure elucidation the racemic mixture of all four stereoisomers of **14** was synthesised (Scheme S1, Supporting Information) and separated by chiral GC (Figure S22). This stereoisomeric mixture was then converted into the corresponding stereoisomers of **15** using the Grubbs II catalyst, followed by isolation of (1R*,5S*)‐**15 ab** and (1S*,5S*)‐**15 a'b’** by column chromatography (Scheme 3 C), for which the relative configurations were solved by NMR spectroscopy (Tables S3 and S4, Figures S23–S36). The stereoisomeric mixture of **14** was separated by chiral HPLC, giving access to two pure stereoisomers of **14** that were identical to the minor enzyme product from **8 a** with 4*S* and the major enzyme product from **8 b** with 4*R* configuration, whereas the remaining two stereoisomers coeluted (Figure S37). NMR spectroscopy of the pure stereoisomers confirmed that they were diastereoisomers (Tables S5 and S6, Figures S38–S51). The Grubbs II cyclisation of (4*S*)‐**14** yielded a product with the same retention time as one of the enantiomers of (1R*,5S*)‐**15 ab** on a chiral GC phase (Figure S52), establishing the full structure of (1*R*,5*S*)‐**15** for this material and, conclusively, of (3*S*,4*S*)‐**14** for its precursor, the minor enzyme product from **8 a**. Thus, the major product is (3*R*,4*S*)‐**14**. Similarly, (4*R*)‐**14** was converted into a product with the same retention time as one of the enantiomers of (1S*,5S*)‐**15 a'b’**, thus identified as (1*R*,5*R*)‐**15**. In conclusion, its precursor (3*S*,4*R*)‐**14** is the major and (3*R*,4*R*)‐**14** is the minor enzyme product from **8 b**.

With **9 b**, a high diastereoselectivity is observed for CpLS, even higher than its enantioselectivity with GPP, with attack of water at C3 preferentially from the substrates’ *Re* faces at C3 in both cases, whereas the selectivity with **9 a** is poor with attack of water mainly from the *Si* face. With TmS, **9 a** was converted into (3*R*,4*S*)‐**14 a’** and (3*S*,4*S*)‐**14 a**, whereas from **9 b** the products (3*R*,4*R*)‐**14 b** and (3*S*,4*R*)‐**14 b’** were obtained (Figure S22, Supporting Information). For this enzyme the attack of water at C3 of **9 a** and **9 b** proceeds with good to high diastereoselectivity mainly at the *Si* face, whereas for GPP *Re* face attack is preferred. These data suggest that the conformations of **9 a** and **9 b** in the active sites of CpLS and TmS in comparison to GPP are strongly influenced by the additional 4‐Me group.

TmS converts FPP into T‐muurolol (**16**) through a cascade with isomerisation to NPP, followed by cyclisation to **A**, a 1,3‐hydride shift to **B**, cyclisation to **C** and attack of water (Scheme 4 A). The installation of the stereocentre at C7 in **A** is explained by FPP isomerisation through *syn*‐allylic transposition to (*R*)‐NPP and *anti*‐S_N_2’ attack with 1*Re*,10*Re* cyclisation, with a defined stereochemical course for the enantiotopic C1 hydrogens of FPP. As a consequence, the 1‐*pro*‐*S* hydrogen of FPP selectively migrates into the *i*Pr group of **B**, as demonstrated experimentally by enantioselective deuteration of FPP.^18^

**Scheme 4:**
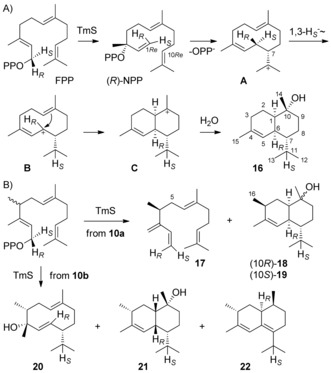
Enzymatic conversions with TmS. A) Cyclisation mechanism from FPP to **16**, B) products obtained from **10 a** and **10 b** with TmS. Carbon numberings for **18**–**22** as for **16**, the additional Me group is numbered C16.

GPP in conjunction with **8 a** or **8 b** was efficiently converted by FPPS and TmS, however, the observed product selectivity of TmS was much lower than with FPP and in both cases complex mixtures of sesquiterpenes were formed (Figure S53, Supporting Information). From substrate **8 a**, (*S*)‐4‐methyl‐(*E*)‐β‐farnesene (**17**), (3*S*)‐3‐methyl‐T‐muurolol (**18**) and its epimer (3*S*)‐3‐methyl‐10‐*epi*‐T‐muurolol (**19**), formed by attack of water from the opposite face at C10 of a cation **C** analogue, were isolated (Scheme 4 B), whereas the conversion of **8 b** yielded (3*R*,4*R*,7*R*)‐3‐methyl‐4‐hydroxygermacra‐1(10),5‐diene (**20**), (3*R*)‐3‐methyl‐1,6‐*diepi*‐T‐muurolol (**21**) and (1*S*,3*R*,10*S*)‐3‐methylzonarene (**22**) (NMR data: Tables S7–S12 and Figures S54–S95). For all these molecules the configurations at C3 were deduced from the structure of their precursor **10 a** or **10 b**, while the relative orientation of the additional stereocentres in compounds **18**–**22** was determined by NOESY spectroscopy, ultimately allowing for full structure elucidation of all six compounds (for **19** vide infra).

During the biosynthesis of **18**–**22** a similar 1,3‐hydride shift as from cation **A** to **B** can be assumed. To investigate this hydride migration, (1,1–^2^H_2_)‐**8 a** and (1,1–^2^H_2_)‐**8 b** were synthesised (Scheme S2A, Supporting Information) and enzymatically converted with (7–^13^C)GPP^19^ by FPPS and TmS. ^13^C‐NMR analysis of the obtained products established the proposed hydride migrations by slightly upfield shifted triplets for C11, indicating a direct ^13^C–^2^H bond (Figures S96 and S97). The hydride migrations were further supported by GC‐EIMS analysis in which the *i*Pr groups of **18**–**22** are lost by α‐cleavage, allowing to locate deuterium within this group (Figures S98 and S99). The specific migration of the enantiotopic C1 hydrogens in FPP in the 1,3‐hydride shift from **A** to **B** depends on the configuration at C7 of **A**, with migration of H_*S*_ for (*S*)‐**A** and of H_*R*_ for (*R*)‐**A**.^18^ Conclusively, if the stereocentre at C7 is retained in the final product, the fate of H_*S*_ and H_*R*_ can serve as a marker to determine absolute configurations. This strategy was also applied to the methylated sesquiterpene analogues obtained with TmS by synthesis of (*R*)‐ and (*S*)‐(1–^13^C,1–^2^H)‐**8 a**, and (*R*)‐ and (*S*)‐(1–^13^C,1–^2^H)‐**8 b** (Scheme S2B) that were all obtained in high enantiomeric purity (Figure S100). Their incubation with GPP, FPPS and TmS showed for **18**–**22** migration of the 1‐*pro*‐*S* hydrogen of **10 a** or **10 b** into the *i*Pr group (Figures S101–S105), whereas the 1‐*pro*‐*R* hydrogen remains bound to C6 of **18**–**21**, which further supports their assigned absolute configurations (Figures S101–S104). Only for **22** the deuterium label ends up in a neighbouring position (C1) as indicated by a characteristic Δ*δ*=−0.07 ppm for the labelled carbon (Figure S105). For **17** a specific stereochemical course for the C1 hydrogens was observed by product analysis through HSQC spectroscopy, revealing that the 1‐*pro*‐*R* hydrogen ends up in the H1_*Z*_ and the 1‐*pro*‐*S* hydrogen in the H1_*E*_ position (Figure S106), which supports its enzymatic formation and reflects the situation in the intermediate (*R*)‐NPP towards **16** (Scheme 4 A). For the biosynthesis of **22** another 1,2‐hydride shift from C6 to C7 of **10 b** was assumed. Direct evidence for this step was obtained by incubation of (3–^13^C,2–^2^H)GPP and **8 b** with FPPS and TmS, resulting in an upfield shifted triplet for C10 of **22** as a result of ^13^C–^2^H spin coupling (Figure S107). The terminal deprotonation to this compound was followed by incubation of (2‐^2^H)DMAPP,^20^ IPP and **8 b** with FPPS and TmS. The main product of this reaction was (7‐^2^H)‐**16**, but unlabelled **22** was also detected (Figure S108).

It is well known that the elongation of oligoprenyl diphosphates with IPP proceeds with inversion of configuration at C1 of the allyl diphosphate^21^ and *Si* face attack at C4 of IPP.^15^ As discussed above, the face selectivity regarding the IPP analogues **8 a** and **8 b** is the same. To investigate whether also configuration inversion at C1 of the allyl diphosphate occurs, (*R*)‐ and (*S*)‐(1–^13^C,1–^2^H)GPP^22^ and **8 a** or **8 b** were incubated with FPPS and TmS. HSQC analysis of the obtained products revealed selective deuterium incorporation into H2_β_ of **18**, **20** and **22** from (*R*)‐ and into H2_α_ from (*S*)‐(1–^13^C,1–^2^H)GPP (Figures S109–S111, Supporting Information), in agreement with configurational inversion. Based on these findings, an NMR data assignment for the diastereotopic C5 hydrogens of **17** was possible (Figure S112). Also, the NMR shifts of the C2 hydrogens of **19** could be assigned (Figure S113), which allowed for its unambiguous configuration determination, that was without this information obscured by the identical chemical shifts of H1 and H2_α_. Through this labelling strategy also the C2 hydrogens of **21** with almost identical chemical shifts were distinguished (Figure S114, 1.75 ppm for H2_α_ and 1.72 ppm for H2_β_).

In summary, we have demonstrated that methylated IPP analogues can be enzymatically incorporated into methylated terpenes, giving access to a chemical space that is naturally realised only in a few known cases. The incorporation sites of the extra Me groups set a stereochemical anchor in the products that allows to assign their absolute configurations. In conjunction with labelling experiments complex and interesting stereochemical problems and enzyme mechanistic aspects can be solved. We will extend this work in the future using further IPP analogues and TSs for the enzymatic synthesis of methylated terpenes.

## Conflict of interest

The authors declare no conflict of interest.

## Supporting information

As a service to our authors and readers, this journal provides supporting information supplied by the authors. Such materials are peer reviewed and may be re‐organized for online delivery, but are not copy‐edited or typeset. Technical support issues arising from supporting information (other than missing files) should be addressed to the authors.

Supplementary

## References

[1] J. S. Dickschat , Angew. Chem. Int. Ed. 2019, 58, 15964; 10.1002/anie.201905312 31183935

[2] J. S. Dickschat , Nat. Prod. Rep. 2016, 33, 87. 26563452 10.1039/c5np00102a

[3] M. B. Quin , C. M. Flynn , C. Schmidt-Dannert , Nat. Prod. Rep. 2014, 31, 1449. 25171145 10.1039/c4np00075g PMC4167380

[4] A. Minami , T. Ozaki , C. Liu , H. Oikawa , Nat. Prod. Rep. 2018, 35, 1330. 29855001 10.1039/c8np00026c

[5] T. Mitsuhashi , I. Abe , ChemBioChem 2018, 19, 1106. 10.1002/cbic.201800120 29675947

[6] D. W. Christianson , Chem. Rev. 2017, 117, 11570. 28841019 10.1021/acs.chemrev.7b00287 PMC5599884

[7] R. J. Peters , Nat. Prod. Rep. 2010, 27, 1521. 20890488 10.1039/c0np00019a PMC3766046

[8a] J. S. Dickschat , T. Nawrath , V. Thiel , B. Kunze , R. Müller , S. Schulz , Angew. Chem. Int. Ed. 2007, 46, 8287; 10.1002/anie.200702496 17899580

[8b] M. Komatsu , M. Tsuda , S. Omura , H. Oikawa , H. Ikeda , Proc. Natl. Acad. Sci. USA 2008, 105, 7422; 18492804 10.1073/pnas.0802312105 PMC2387273

[8c] C.-M. Wang , D. E. Cane , J. Am. Chem. Soc. 2008, 130, 8908. 18563898 10.1021/ja803639g PMC3023297

[9a] S. H. von Reuß , M. Kai , B. Piechulla , W. Francke , Angew. Chem. Int. Ed. 2010, 49, 2009; 10.1002/anie.200905680 20155769

[9b] S. von Reuß , D. Domick , M. C. Lemfack , N. Magnus , M. Kai , T. Weise , B. Piechulla , J. Am. Chem. Soc. 2018, 140, 11855. 30133268 10.1021/jacs.8b08510

[10] T. Ozaki , S. S. Shinde , L. Gao , R. Okuizumi , C. Liu , Y. Ogasawara , X. Lei , T. Dairi , A. Minami , H. Oikawa , Angew. Chem. Int. Ed. 2018, 57, 6629; 10.1002/anie.201802116 29603559

[11] D. A. Schooley , K. J. Judy , B. J. Bergot , M. S. Hall , J. B. Siddall , Proc. Natl. Acad. Sci. USA 1973, 70, 2921. 16592112 10.1073/pnas.70.10.2921 PMC427139

[12a] D. J. Miller , F. Yu , R. K. Allemann , ChemBioChem 2007, 8, 1819; 17683054 10.1002/cbic.200700219

[12b] J. A. Faraldos , Y. Zhao , P. E. O'Maille , J. P. Noel , R. M. Coates , ChemBioChem 2007, 8, 1826; 17886322 10.1002/cbic.200700398 PMC2735885

[12c] O. Cascón , S. Touchet , D. J. Miller , V. Gonzalez , J. A. Faraldos , R. K. Allemann , Chem. Commun. 2012, 48, 9702; 10.1039/c2cc35542f 22914774

[12d] S. Touchet , K. Chamberlain , C. M. Woodcock , D. J. Miller , M. A. Birkett , J. A. Pickett , R. K. Allemann , Chem. Commun. 2015, 51, 7550; 10.1039/c5cc01814e 25847629

[12e] M. Demiray , X. Tang , T. Wirth , J. A. Faraldos , R. K. Allemann , Angew. Chem. Int. Ed. 2017, 56, 4347; 10.1002/anie.201609557 PMC5396139 28294491

[12f] C. Oberhauser , V. Harms , K. Seidel , B. Schröder , K. Ekramzadeh , S. Beutel , S. Winkler , L. Lauterbach , J. S. Dickschat , A. Kirschning , Angew. Chem. Int. Ed. 2018, 57, 11802; 10.1002/anie.201805526 29953712

[13] P. Rabe , J. Rinkel , B. Nubbemeyer , T. G. Köllner , F. Chen , J. S. Dickschat , Angew. Chem. Int. Ed. 2016, 55, 15420; 10.1002/anie.201608971 27862766

[14] T. Koyama , K. Ogura , S. Seto , J. Am. Chem. Soc. 1977, 99, 1999.839020

[15] J. W. Cornforth , R. H. Cornforth , G. Popjak , L. Yengoyan , J. Biol. Chem. 1966, 241, 3970. 4288360

[16a] P. Rabe , J. S. Dickschat , Angew. Chem. Int. Ed. 2013, 52, 1810; 10.1002/anie.201209103 23307484

[16b] P. Rabe , T. Schmitz , J. S. Dickschat , Beilstein J. Org. Chem. 2016, 12, 1839. 27829890 10.3762/bjoc.12.173 PMC5082573

[17] M. Winter , H. Schinz , M. Stoll , Helv. Chim. Acta 1947, 30, 2213. 18897253 10.1002/hlca.19470300737

[18] J. Rinkel , P. Rabe , P. Garbeva , J. S. Dickschat , Angew. Chem. Int. Ed. 2016, 55, 13593; 10.1002/anie.201608042 27666571

[19] T. Mitsuhashi , J. Rinkel , M. Okada , I. Abe , J. S. Dickschat , Chem. Eur. J. 2017, 23, 10053. 28671289 10.1002/chem.201702766

[20] J. Rinkel , P. Rabe , X. Chen , T. G. Köllner , F. Chen , J. S. Dickschat , Chem. Eur. J. 2017, 23, 10501. 28696553 10.1002/chem.201702704

[21] J. W. Cornforth , R. H. Cornforth , C. Donninger , G. Popjak , Proc. R. Soc. London Ser. B 1966, 163, 492. 4379291 10.1098/rspb.1966.0004

[22] P. Rabe , J. Rinkel , E. Dolja , T. Schmitz , B. Nubbemeyer , T. H. Luu , J. S. Dickschat , Angew. Chem. Int. Ed. 2017, 56, 2776; 10.1002/anie.201612439 28146322

