# Supplementary material for: Enzymatic Synthesis of Methylated Terpene Analogues Using the Plasticity of Bacterial Terpene Synthases
Source: Chemistry. 2020 Jan 30;26(10):2178–82. doi: 10.1002/chem.201905827 (PMC7065205; doi:10.1002/chem.201905827)
Supplement: Supplementary file 1 — Supplementary [file CHEM-26-2178-s001.pdf]

CHEMISTRY
A European Journal

Supporting Information

Enzymatic Synthesis of Methylated Terpene Analogues Using the Plasticity of Bacterial Terpene Synthases

Anwei Hou, Lukas Lauterbach, and Jeroen S. Dickschat*[a]

chem_201905827_sm_miscellaneous_information.pdf

General synthetic methods

Chemicals were obtained from Sigma Aldrich (St. Louis, MO, USA), Thermo Fisher Scientific (Waltham, MA, USA) and TCI (Tokyo, Japan) and used without further purification. Solvents for reactions were dried according to standard procedures. POLYGRAM® SIL G/UV254 plates (Macherey-Nagel, Düren, Germany) were used for TLC. The staining solution for TLC was molybdophosphoric acid in EtOH (10 g/100 mL). The solvents used for purification were distilled before use. Flash chromatography was performed with silica Geduran® Si 60 (40 – 63 μm, Merck, Darmstadt, Germany).

Synthesis of (*E*)- and (Z)-4-methyl-IPP

Synthesis of (*E*)- and (Z)-3-methylpent-3-en-1-ol (7)

The synthesis of **7** was performed following a previously described procedure.[1] Briefly, (*E*)- or (*Z*)-2-bromobut-2-ene (540 mg, 4.00 mmol) was dissolved in THF (5 mL) and the solution was cooled to –78 °C. Then *t*-BuLi (1.7 M in pentane, 4.7 mL, 8.00 mmol, 2.0 eq.) was added dropwise. After stirring at –78 °C for 40 min, ethylene oxide (2.5-3.3 M in THF, 2.7 mL, 2.0 eq.) was added and the reaction mixture was allowed to warm to 0 °C over 4 h. The reaction was quenched by the addition of a solution of NH_4Cl (aq.) and the product was extracted with Et_2O (3x 30 mL). The combined organic layers were washed with brine and dried with $MgSO_4$. The solvent was removed under reduced pressure. The residue was purified by silica gel chromatography [pentane/Et_2O (1/1), R_f = 0.40] to afford the products **7a** and **7b** as a colorless oils.

(*E*)-3-Methylpent-3-en-1-ol (7a). Yield: 380mg (3.79 mmol, 95%). ^1H NMR (500 MHz, $CDCl_3$): δ = 5.35 – 5.29 (m, 1H, CH), 3.66 (t, $^3J_{H,H}$= 6.2 Hz, 2H, CH_2), 2.25 (t, $^3J_{H,H}$ = 6.1 Hz, 2H, CH_2), 1.63 (s, 3H, CH_3), 1.61 (d, $^3J_{H,H}$= 6.7 Hz, 3H, CH_3), 1.48 (br s, 1H, OH) ppm. ^{13}C NMR (126 MHz, $CDCl_3$): δ = 132.11 (C_q), 122.09 (CH), 60.23 (CH_2), 42.79 (CH_2), 15.50 (CH_3), 13.62 (CH_3) ppm. MS (EI, 70 eV): *m*/*z* (%) = 100 (28), 82 (23), 67 (70), 55 (33), 41(100).

(*Z*)-3-Methylpent-3-en-1-ol (7a). Yield: 340 mg (3.39 mmol, 85%). ^1H NMR (300 MHz, $CDCl_3$): δ = 5.46 – 5.36 (m, 1H, CH), 3.68 (t, $^3J_{H,H}$= 6.7 Hz, 2H, CH_2), 2.34 (t, $^3J_{H,H}$ = 6.7 Hz, 2H, CH_2), 1.73 – 1.70 (m, 3H, CH_3), 1.61 (d, $^3J_{H,H}$ = 6.7 Hz, 3H, CH_3), 1.48 (s, 1H, OH) ppm. ^{13}C NMR (75 MHz, $CDCl_3$): δ = 132.03 (C_q), 122.49 (CH), 60.71 (CH_2), 34.79 (CH_2), 23.47 (CH_3), 13.58 (CH_3) ppm. MS (EI, 70 eV): *m*/*z* (%) = 100 (31), 82 (24), 67 (84), 55 (41), 41(100).

Trisammonium (*E*) and (Z)-4-methylisopentenyl diphosphate (8)

The alcohol **7b** (340 mg, 3.39 mmol) and DMAP (1.37 g, 11.21 mmol, 3.3 eq.) were dissolved in DCM (20 mL). After the solution was cooled to 0 °C, a solution of TsCl (1.62 g, 8.47 mmol, 2.5 eq.) in DCM (5 mL) was added dropwise and the reaction mixture was stirred at room temperature for 5 h. The reaction was poured onto a mixture of ice (50 g) and sat. NH_4Cl (50 mL), followed by extraction with Et_2O (3x 50 mL). The combined organic layers were dried with $MgSO_4$ and evaporated under reduced pressure. The residue was purified by silica gel chromatography [pentane/Et_2O (5/1), R_f=0.28] to afford (*Z*)-3-methylpent-3-en-1-yl 4-methylbenzenesulfonate as a colorless oil. Following the same procedure, **7a** (380mg, 3.79 mmol) was converted to (*E*)-3-methylpent-3-en-1-yl 4-methylbenzenesulfonate that was obtained as a colourless oil.

(*E*)-3-Methylpent-3-en-1-yl 4-methylbenzenesulfonate. Yield: 0.51 g (2.01 mmol, 53%). ^1H NMR (500 MHz, $CDCl_3$): δ = 7.80 – 7.75 (m, 2H, 2x CH), 7.34 (d, $^3J_{H,H}$= 8.1 Hz, 2H, 2x CH), 5.24-5.18 (m, 1H, CH), 4.07 (t, $^3J_{H,H}$ = 6.9 Hz, 2H, CH_2), 2.45 (s, 3H, CH_3), 2.30 (t, $^3J_{H,H}$= 7.1 Hz, 2H, CH_2), 1.53 (d, $^3J_{H,H}$ = 6.7 Hz, 3H, CH_3), 1.51 (s, 3H, CH_3) ppm. ^{13}C NMR (126 MHz, $CDCl_3$): δ = 144.74 (C_q), 133.43 (C_q), 130.21 (C_q), 129.89 (2x CH), 128.05 (2x CH), 122.37 (CH), 69.20 (CH_2), 38.81 (CH_2), 21.78 (CH_3), 15.71 (CH_3), 13.57 (CH_3) ppm. GC (HP-5MS): *I* = 1956. MS (EI, 70 eV): *m*/*z* (%) = 173 (3), 155 (13), 91 (37), 82 (100), 67 (73), 55 (6), 41 (18).

(*Z*)-3-Methylpent-3-en-1-yl 4-methylbenzenesulfonate. Yield: 0.52 g (2.04 mmol, 60%). ^{1}H NMR (500 MHz, $CDCl_3$): δ = 7.80 – 7.77 (m, 2H, 2x CH), 7.36 – 7.32 (m, 2H, 2x CH), 5.30 (q, $^{3}J_{H,H}$ = 6.7 Hz, 1H, CH), 4.04 (t, $^{3}J_{H,H}$ = 7.2 Hz, 2H, CH_2), 2.45 (s, 3H, CH_3), 2.38 (t, $^{3}J_{H,H}$ = 7.2 Hz, 2H, CH_2), 1.61 – 1.59 (m, 3H, CH_3), 1.50 (d, $^{3}J_{H,H}$ = 6.9 Hz, 3H, CH_3) ppm. ^{13}C NMR (126 MHz, $CDCl_3$): δ = 144.79 (C_q), 133.37 (C_q), 130.02 (C_q), 129.91 (2x CH), 128.00 (2x CH), 123.08 (CH), 68.55 (CH_2), 31.21(CH_2), 23.50 (CH_3), 21.78 (CH_3), 13.45 (CH_3) ppm. GC (HP-5MS): *I* = 1935. MS (EI, 70 eV): *m/z* (%) = 173 (3), 155 (14), 91 (42), 82 (100), 67 (83), 55 (7), 41 (18).

$(Bu_4N)_3HP_2O_7$ (1.8 g, 1.99 mmol, 2.0 eq.) was dissolved in acetonitrile (0.8 mL), and (*Z*)-3-methylpent-3-en-1-yl 4-methylbenzenesulfonate (260 mg, 1.02 mmol) was added into the solution dropwise. After stirring at room temperature for 3 h, the solvent was evaporated under reduced pressure. The residue was loaded onto an ion exchange resin column (DOWEX® 50W-X8, 100-200 mesh, $NH4^+$ form), followed by washing with two column volumes of elution buffer (0.025 M NH_4HCO_3 in 2% *i*PrOH/H_2O). The eluted fraction was lyophilized to give the crude product which was purified by dissolving it into 0.1 M NH_4HCO_3 (3 mL) solution and extraction with acetonitrile/*i*PrOH (1/1, 3x 7 mL).[2] The combined extracts were evaporated under reduced pressure and lyophilized to give **8b** as a colourless powder. Following the same procedure, (*E*)-3-methylpent-3-en-1-yl 4-methylbenzenesulfonate (510 mg, 2.01 mmol) was converted to **8a** that was obtained as a colourless powder.

Trisammonium (*E*)-4-methylisopentenyl diphosphate (8a). Yield: 535 mg (1.72 mmol, 86%).^{1}H NMR (300 MHz, D_2O): δ = 5.46 – 5.36 (m, 1H, CH), 4.02 (q, $^{3}J_{H,H}$ = 6.8 Hz, $^{3}J_{P,H}$= 6.8 Hz, 2H, CH_2), 2.36 (t, $^{3}J_{H,H}$= 6.9 Hz, 2H, CH_2), 1.67 (s, 3H, CH_3), 1.60 (d, $^{3}J_{H,H}$= 6.8 Hz, 3H, CH_3) ppm. ^{13}C NMR (75 MHz, D_2O): δ = 133.32 (C_q), 121.20 (CH), 64.45 (d, $^{2}J_{P,C}$= 6.0 Hz, CH_2), 39.70 (d, $^{3}J_{P,C}$= 7.4 Hz, CH_2), 14.87 (CH_3), 12.66 (CH_3) ppm. ^{31}P NMR (121 MHz, D_2O): δ = –7.76 (d, $^{2}J_{P,P}$= 21.7 Hz, 1P), –10.58 (d, $^{2}J_{P,P}$= 21.5 Hz, 1P) ppm.

Trisammonium (*Z*)-4-methylisopentenyl diphosphate (8b). Yield: 261 mg (0.84 mmol, 82%). ^{1}H NMR (500 MHz, D_2O): δ = 5.43 – 5.32 (m, 1H, CH), 3.95 (q, $^{3}J_{H,H}$ = 7.1 Hz, $^{3}J_{P,H}$= 7.1 Hz, 2H, CH_2), 2.39 (t, $^{3}J_{H,H}$= 7.0 Hz, 2H, CH_2), 1.70-1.68 (m, 3H, CH_3), 1.56 (d, $^{3}J_{H,H}$ = 7.1 Hz, 3H, CH_3) ppm. ^{13}C NMR (126 MHz, D_2O): δ = 133.18 (C_q), 121.86 (CH), 64.09 (d, $^{2}J_{P,C}$ = 5.9 Hz, CH_2), 32.00 (d, $^{3}J_{P,C}$= 7.1 Hz, CH_2), 22.64 (CH_3), 12.65 (CH_3) ppm. ^{31}P NMR (202 MHz, D_2O): δ = –7.93 (d, $^{2}J_{P,P}$= 21.2 Hz, 1P), –10.59 (d, $^{2}J_{P,P}$= 21.2 Hz, 1P) ppm.

Incubation experiments with recombinant FPPS and CIP

FPPS was purified by the procedure described previously.[3] For small scale test reactions, purified FPPS (0.2 mL in elution buffer from Ni-NTA chromatography, 20 mM Na_2HPO_4, 500 mM NaCl, 500 mM imidazole, 1 mM $MgCl_2$, pH = 7.4, protein concentration ca. 1 mg/mL), and DMAPP or GPP (0.1 mL in 25 mM NH_4HCO_3, 1 mg/mL), **8a** or **8b** (0.1 mL in 25 mM NH_4HCO_3, 2 mg/mL) were added to incubation buffer (0.5 mL, 50 mM Tris-HCl, 10 mM $MgCl_2$, 20% glycerol, pH=8.2), followed by incubation at 28 °C for 4 h. Then CutSmart® buffer (2 μL) and calf intestinal phosphatase (CIP, 1 μL, NEB) were added. The mixture was further incubated at 37 °C for 1 h and then extracted with hexane (0.2 mL). The extract was dried with $MgSO_4$ and analysed by GC/MS.

For large scale reactions, purified FPPS (90 mL in elution buffer, protein concentration ca. 1 mg/mL, from 8 L of *E. coli* expression culture), binding buffer (60 mL), DMAPP or GPP (100 mg) in aqueous NH_4HCO_3 (20 mL, 25 mM NH_4HCO_3), **8a** (100 mg) in aqueous NH_4HCO_3 (20 mL, 25 mM NH_4HCO_3), and incubation buffer (200 mL) were mixed and incubated at 28 °C for 4 h. Then CutSmart® buffer (100 μL) and CIP (20 μL) were added to the reaction mixture. After further incubation at 37 °C for 1 h, the product was extracted with pentane (3x 100 mL). The combined organic layers were evaporated under reduced pressure and the residue was purified by silica gel chromatography [pentane/Et_2O (1/1)] to afford pure **11a** or **12a**.

(*S*)-4-Methylgeraniol, (*S*,*E*)-3,4,7-trimethylocta-2,6-dien-1-ol (11a). Colourless oil. Yield: 2.0 mg (0.012 mmol, 3.6%). R_f [pentane/Et_2O(1/1)] = 0.48. $[\alpha]_D^{20}$ = –5.5 (CH_2Cl_2, *c* 0.2). HRMS

(ToF): *m*/*z* = 168.1511 ($[M]^+$, calc. for $[C_{11}H_{20}O]^+$ 168.1509). GC (HP5-MS): *I* = 1303. IR (diamond ATR): $\tilde{\nu}$ / cm^{-1} = 3325 (w), 2963 (s), 2924 (s), 2871 (m), 2858 (m), 1664 (w), 1451 (m), 1376 (m), 1260 (w), 1095 (w), 1058 (w), 998 (s), 801 (m). The EI mass spectrum (70 eV) is shown in Figure S1. NMR data are given in Table S1 and Figures S2–S8.

(*S*)-4-Methylfarnesol, (*S*,2*E*,6*E*)-3,4,7,11-tetramethyldodeca-2,6,10-trien-1-ol (12a). Colourless oil. Yield: 2.5 mg (0.011 mmol, 4.0%). R_f [pentane/Et_2O(1/1)] = 0.53. $[\alpha]_D^{20}$ = –6.0 (CH_2Cl_2, *c* 0.25). HRMS (ToF): *m*/*z* = 218.2027 ($[M-H_2O]^+$, calc. for $[C_{16}H_{26}]^+$ 218.2029). GC (HP5-MS): *I* = 1764. IR (diamond ATR): $\tilde{\nu}$ / cm^{-1} = 3339 (w), 2962 (m), 2924 (m), 2854 (m), 1663 (w), 1448 (w), 1377 (w), 1259 (m), 1089 (m), 1015 (s), 797 (s), 684 (w), 662 (w). The EI mass spectrum (70 eV) is shown in Figure S10. NMR data are given in Table S2 and Figures S12–S18.

GC/MS analyses

GC/MS analyses were performed on a 7890B GC connected to a 5977A mass selective detector (Agilent, Santa Clara, CA, USA). The GC was equipped with a HP5-MS fused silica capillary column (30 m, 0.25 mm i. d., 0.50 μm film). Specific GC settings were 1) inlet pressure: 77.1 kPa, He at 23.3 mL min^{-1}, 2) injection volume: 2 μL, 3) temperature program: 5 min at 50 °C increasing at 5 °C min^{-1} to 320 °C, 4) 60 s valve time, and 5) carrier gas: He at 1.2 mL min^{-1}. MS settings were 1) source: 230 °C, 2) transfer line: 250 °C, 3) quadrupole: 150 °C and 4) electron energy: 70 eV. Retention indices (*I*) were determined from retention times in comparison to the retention times of of *n*-alkanes (C_7-C_{40}).

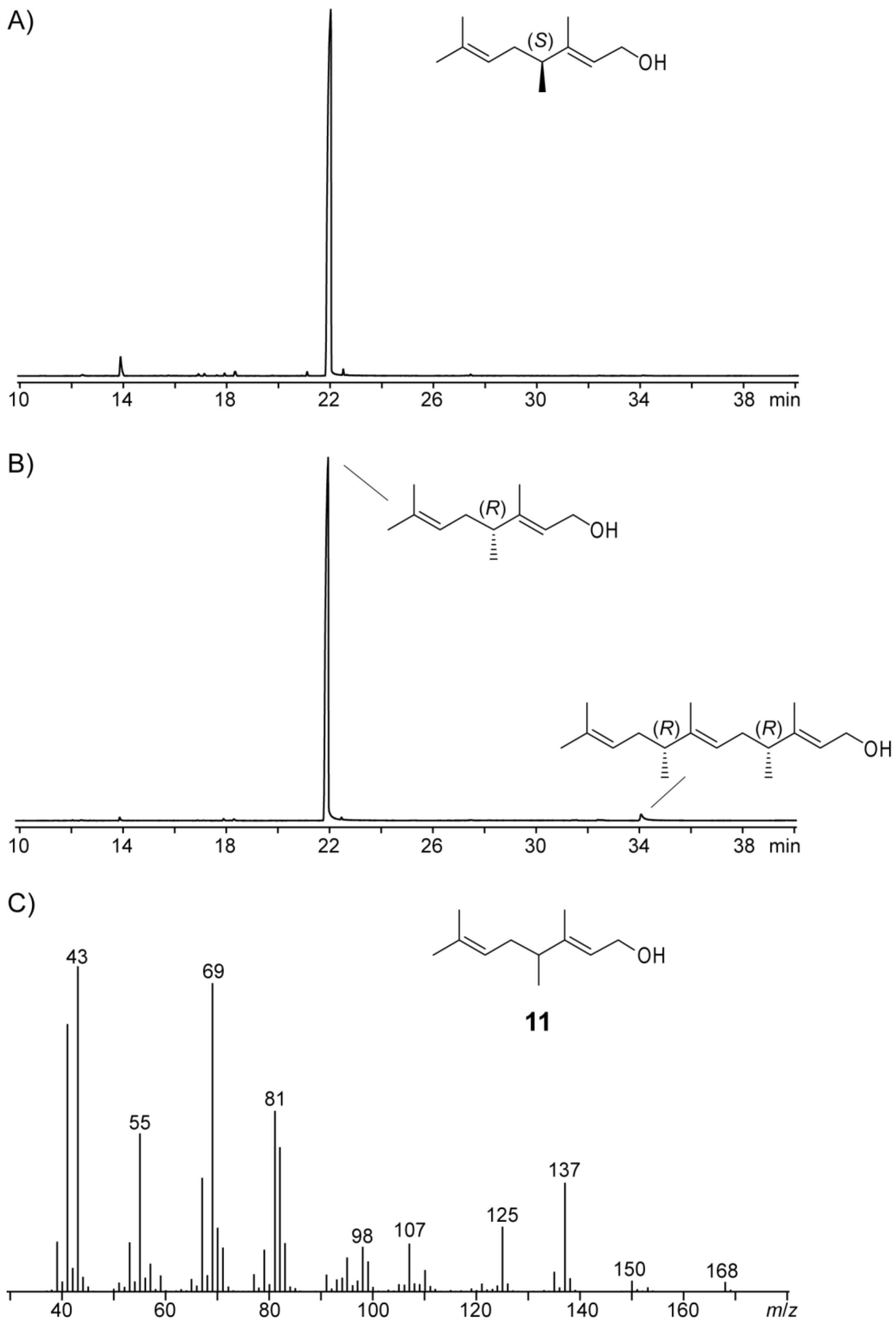

Figure S1. Total ion chromatograms of the products obtained with FPPS after dephosphorylation with CIP from A) DMAPP and (*E*)-4-methyl-IPP (**8a**) and B) DMAPP and (*Z*)-4-methyl-IPP (**8b**). C) EI mass spectrum of 4-methylgeraniol (**11**). Structure assignment for the double elongation product is based on its mass spectrum and tentative.

NMR spectroscopy

NMR spectra were recorded on a Bruker (Billerica, MA, USA) Avance I (300 MHz), Avance I (400 MHz), Avance I (500 MHz), Avance III HD Prodigy (500 MHz) or an Avance III HD Cryo (700 MHz) NMR spectrometer. Spectra were measured in C_6D_6 and referenced against solvent signals (^{1}H-NMR, residual proton signal: δ = 7.16; ^{13}C-NMR: δ = 128.06).[4]

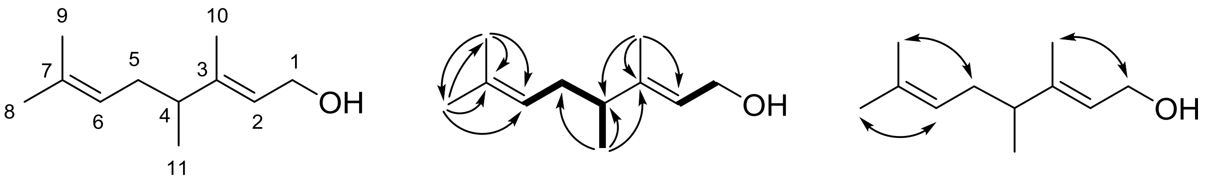

Table S1. NMR data of 4-methylgeraniol (**11**) in C_6D_6 recorded at 298 K.

C[a]		^{13}C[b]	^{1}H[b]
1	CH_2	59.38	3.97 (m)
2	CH	124.22	5.40 (m)
3	C_q	142.36	–
4	CH	43.21	2.08 (m)
5	CH_2	33.97	2.07 (m)
			1.96 (m)
6	CH	123.74	5.15 (m)
7	C_q	131.84	–
8	CH_3	25.92	1.65 (br s)
9	CH_3	17.92	1.53 (br s)
10	CH_3	13.38	1.43 (m)
11	CH_3	19.19	0.96 (d, *J* = 6.8)
–	OH	–	0.54 (br s)

[a] Carbon numbering as shown in the structure above the table (bold lines: ^{1}H,^{1}H-COSY correlations, single headed arrows: HMBC correlations, double headed arrows: NOESY correlations). [b] Chemical shifts δ in ppm, multiplicity: s = singlet, d = doublet, m = multiplet, br = broad, coupling constants *J* are given in Hertz.

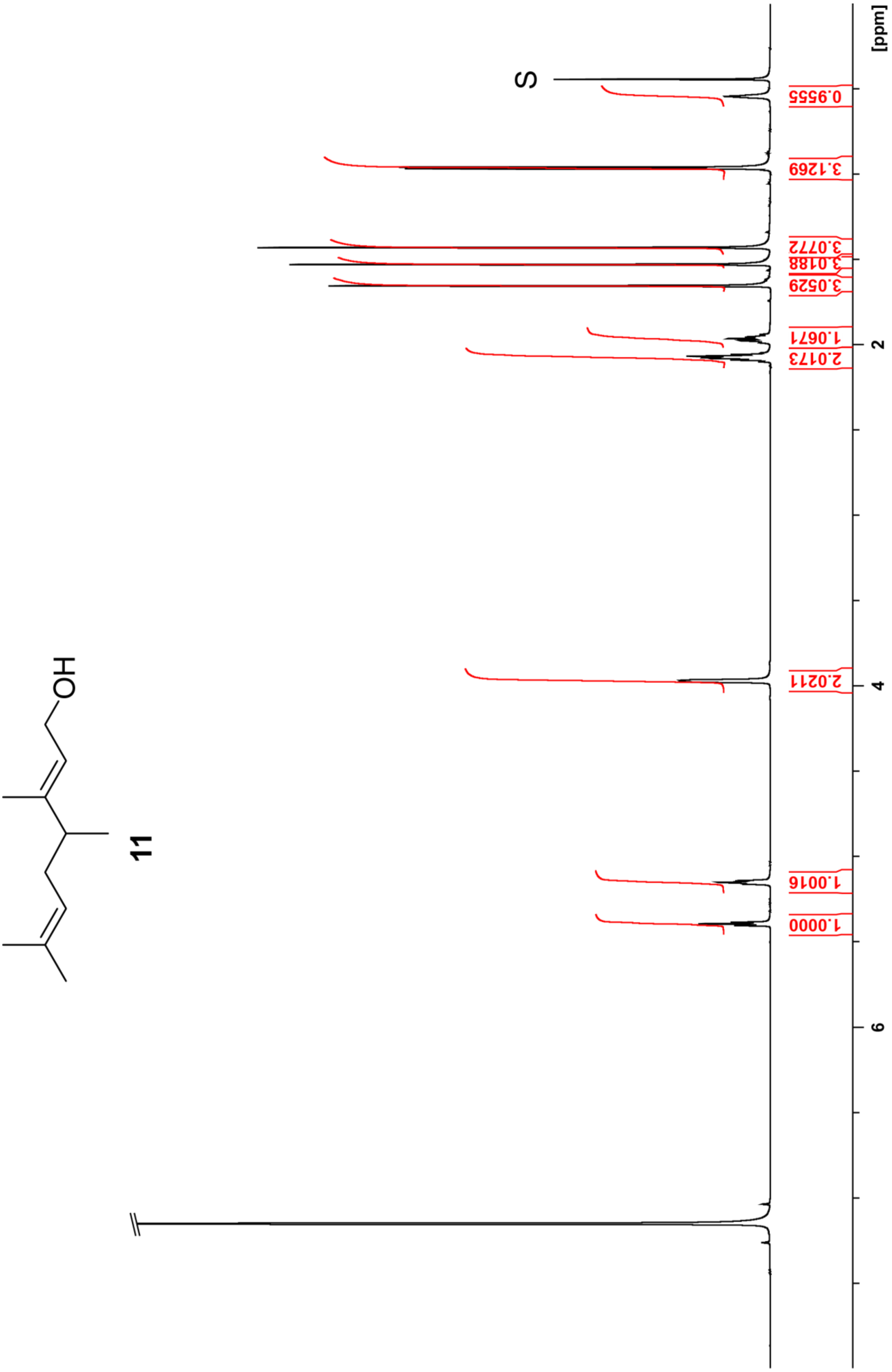

Figure S2. ^{1}H-NMR spectrum of **11** (700 MHz, C_6D_6). S: impurity from deuterated solvent.

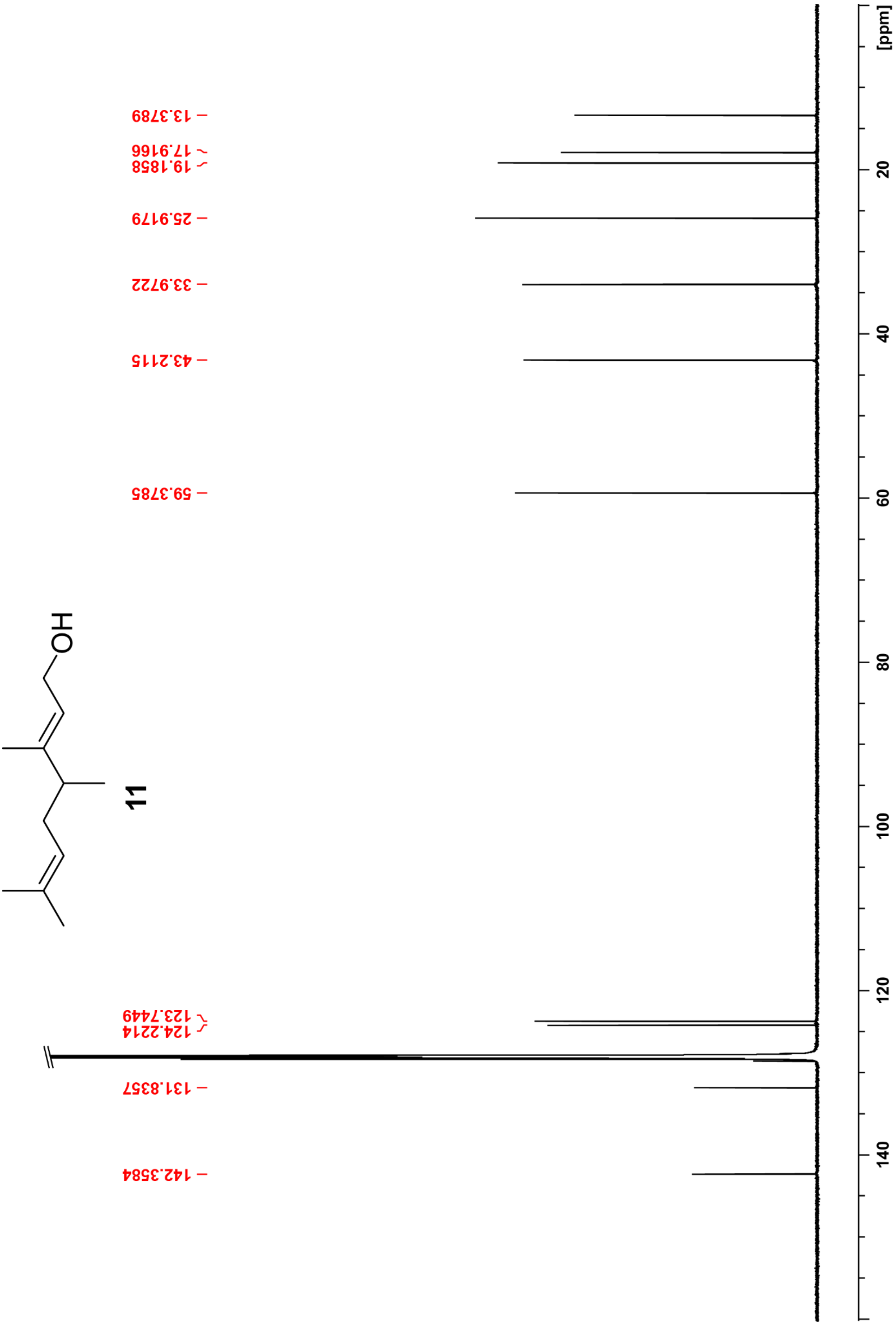

Figure S3. ^{13}C-NMR spectrum of **11** (175 MHz, C_6D_6).

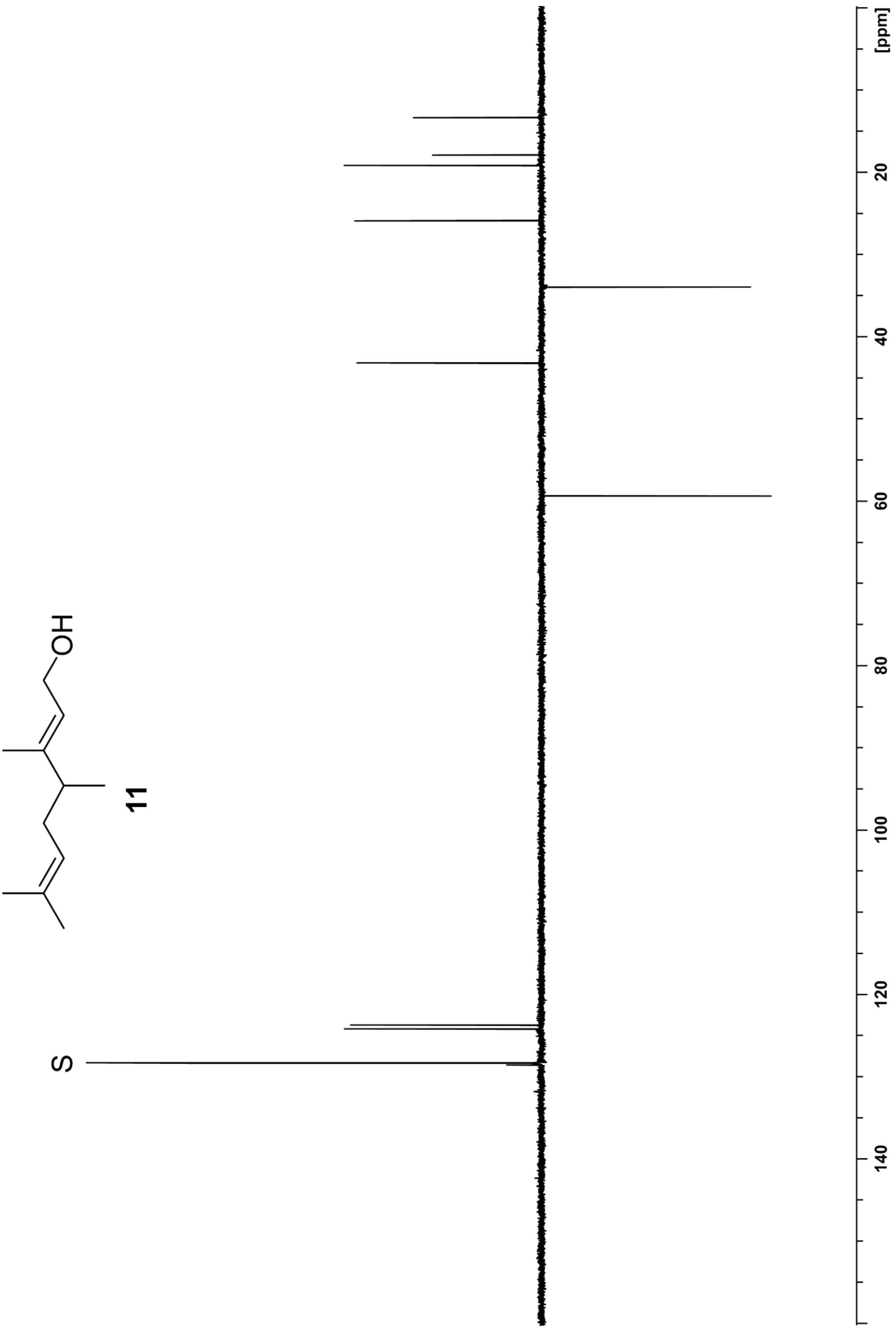

Figure S4. ^{13}C-DEPT spectrum of **11** (175 MHz, C_6D_6). S: residual solvent signal.

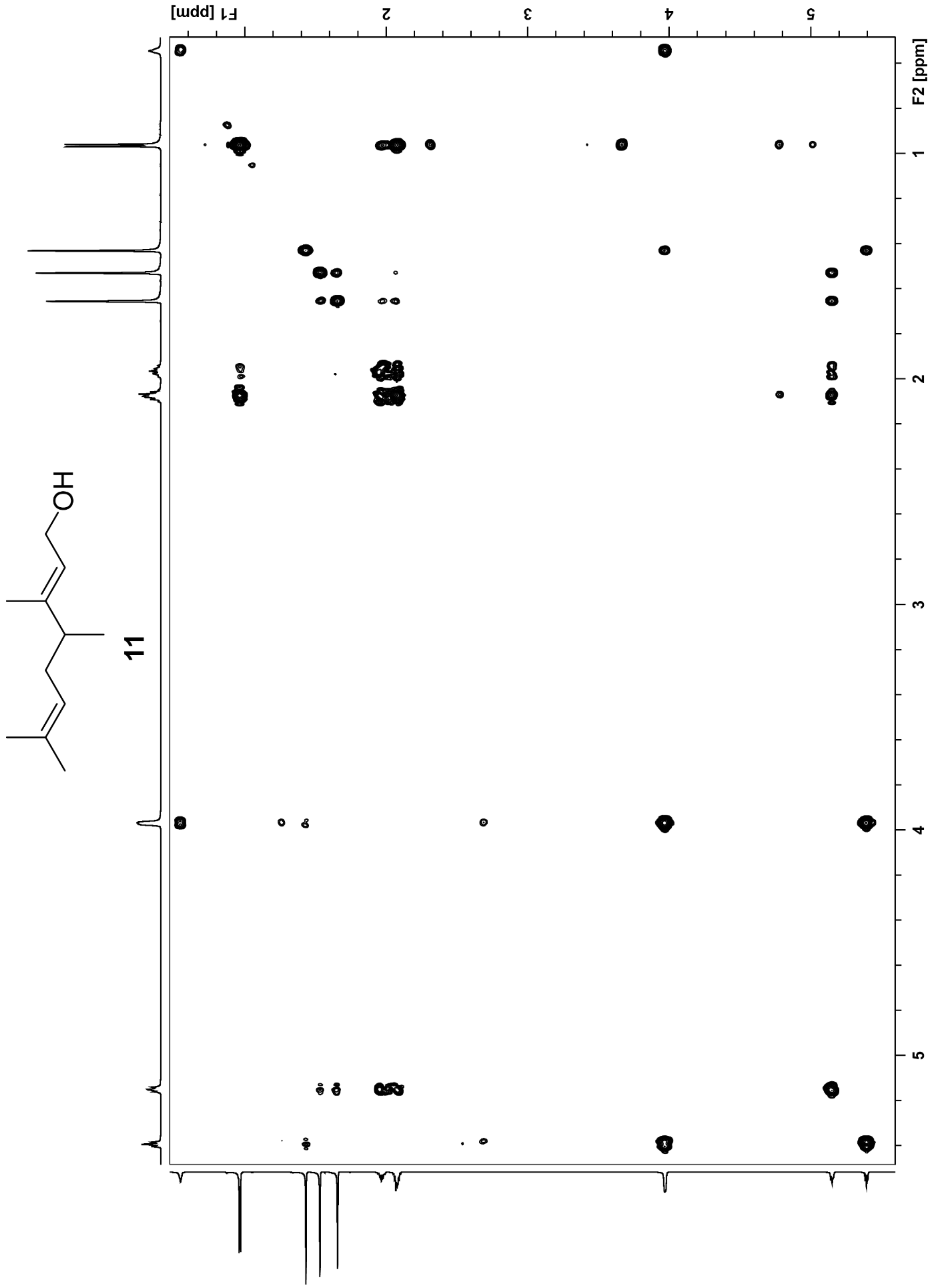

Figure S5. $^{1}H,^{1}H$-COSY spectrum of **11** (C_6D_6).

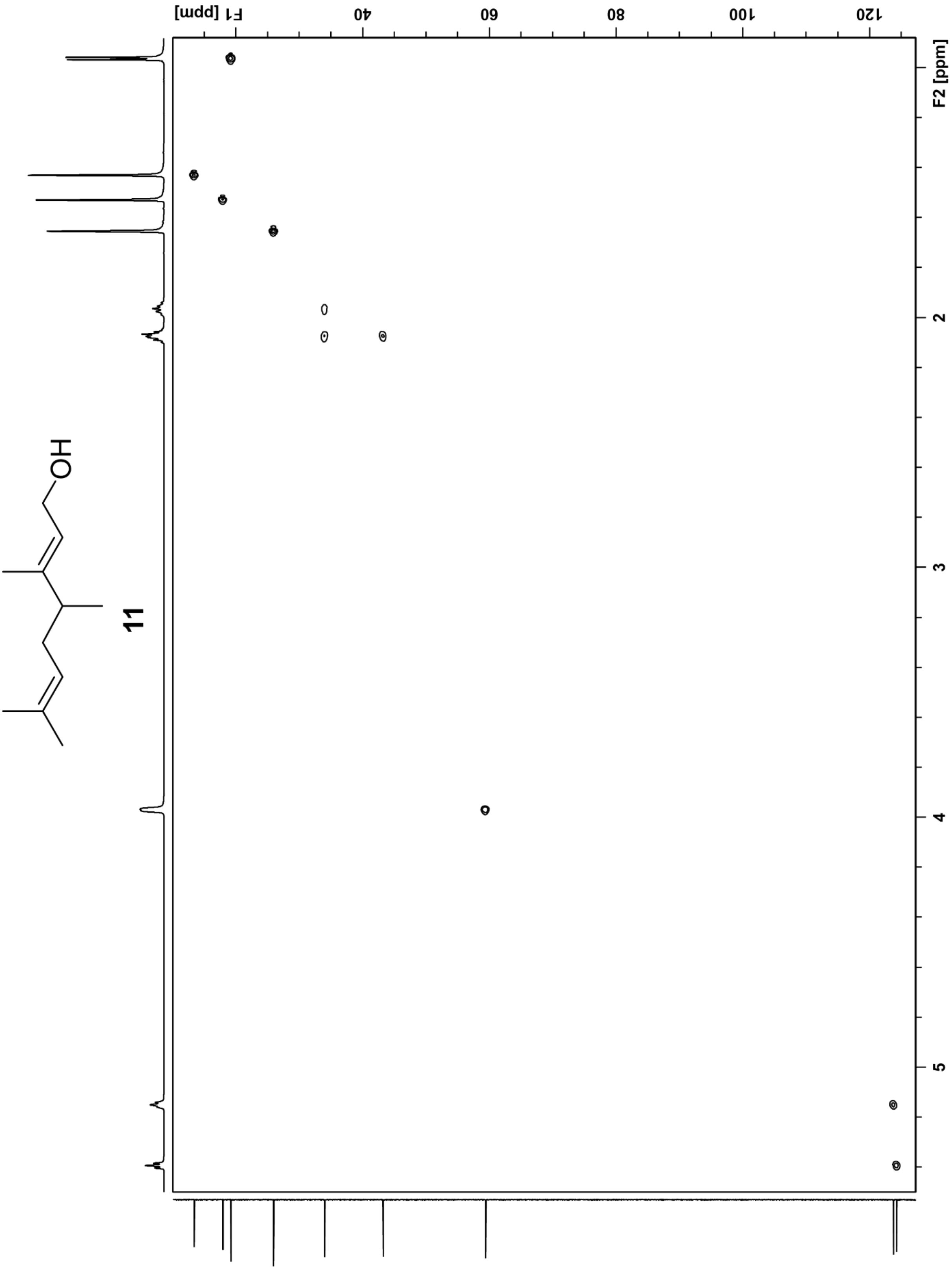

Figure S6. HSQC spectrum of **11** (C_6D_6).

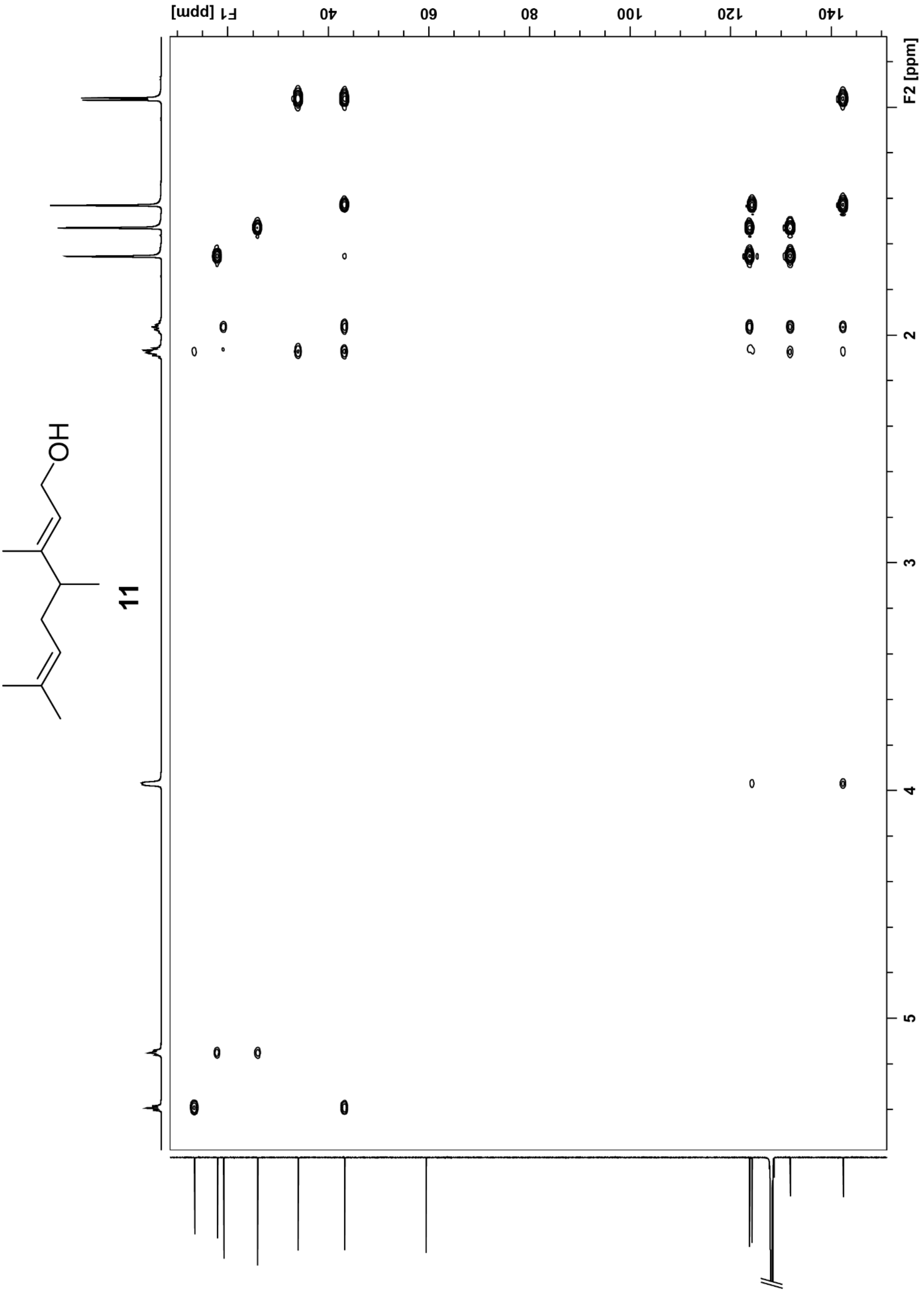

Figure S7. HMBC spectrum of **11** (C_6D_6).

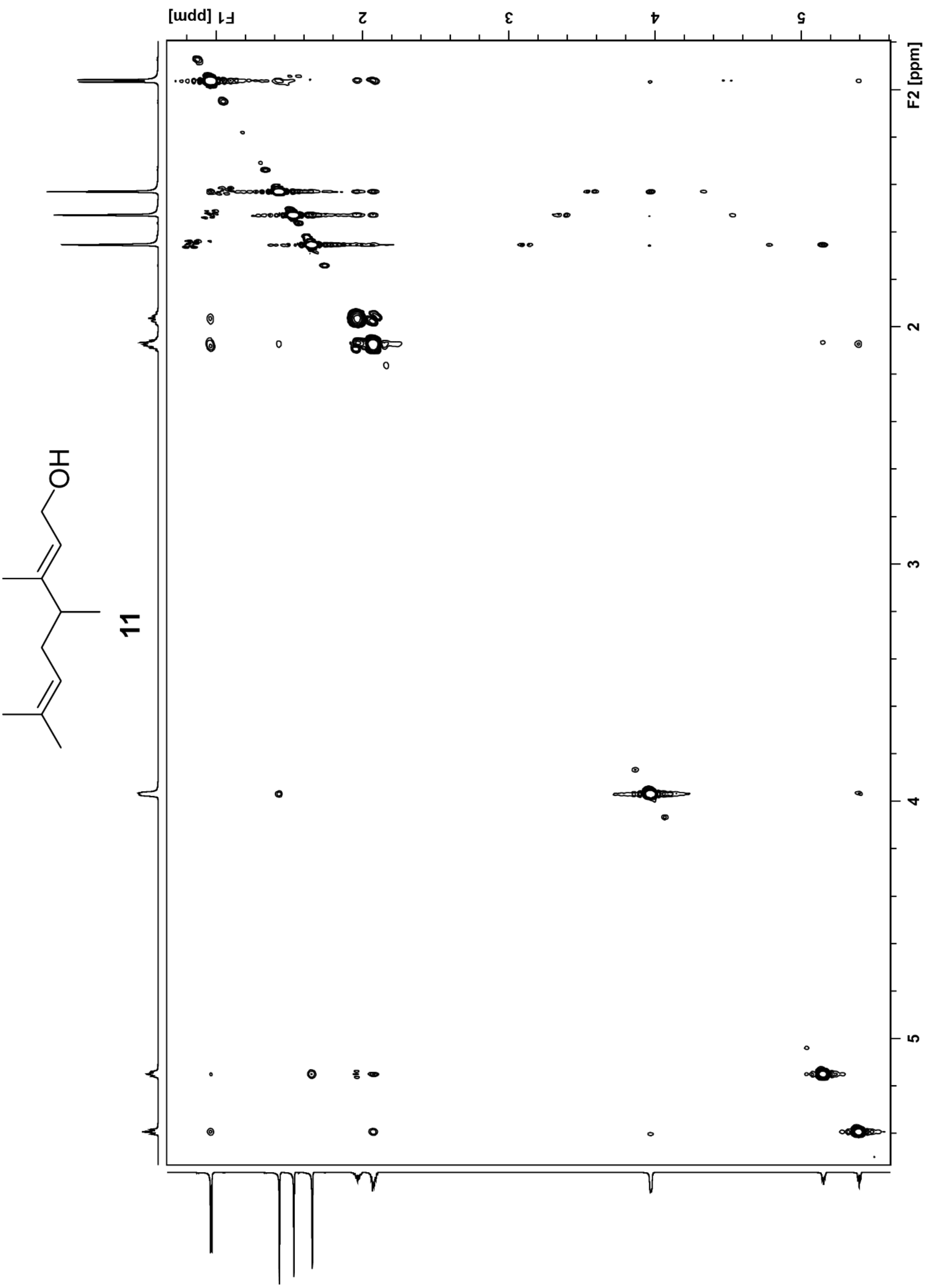

Figure S8. NOESY spectrum of **11** (C_6D_6).

GC analyses using a chiral stationary phase

Chiral GC analyses were performed on an Agilent 7820A GC system (Agilent, Santa Clara, CA, USA) equipped with an FID detector and an Agilent Cyclosil-B capillary column (30 m, 0.25 mm i. d., 0.25 μm film). For analysis of linalool, **11** and **14**, the GC program was: starting from 80 °C, increasing with 0.5 °C/min to 115 °C, followed by increasing with 30 °C/min to 245 °C, then hold for 5 min. For analysis of **12**, the GC program was: starting from 120 °C, increasing with 0.5 °C/min to 155 °C, followed by increasing with 30 °C/min to 245 °C, then hold for 5 min. For analysis of **15**, the GC program was: starting from 30 °C, increasing with 1 °C/min to 68 °C, followed by increasing with 30 °C/min to 220 °C, then hold for 5 min. Inlet temperature was 250 °C, inject volume was 1 μL, the carrier gas was H_2 at 2.3 mL/min.

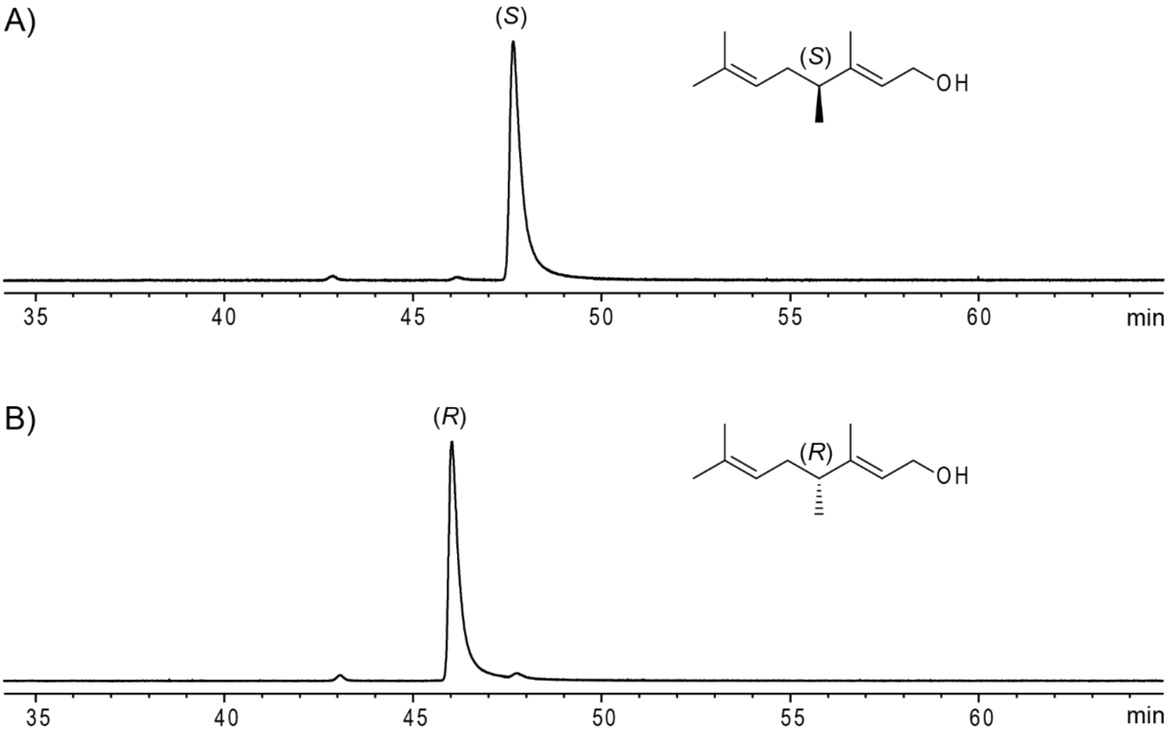

Figure S9. Gas chromatograms on a chiral stationary phase of A) (*S*)-**11a** (98% *ee*) obtained from (*E*)-4-methyl-IPP (**8a**) and B) (*R*)-**11b** (98% *ee*) obtained from (*Z*)-4-methyl-IPP (**8b**).

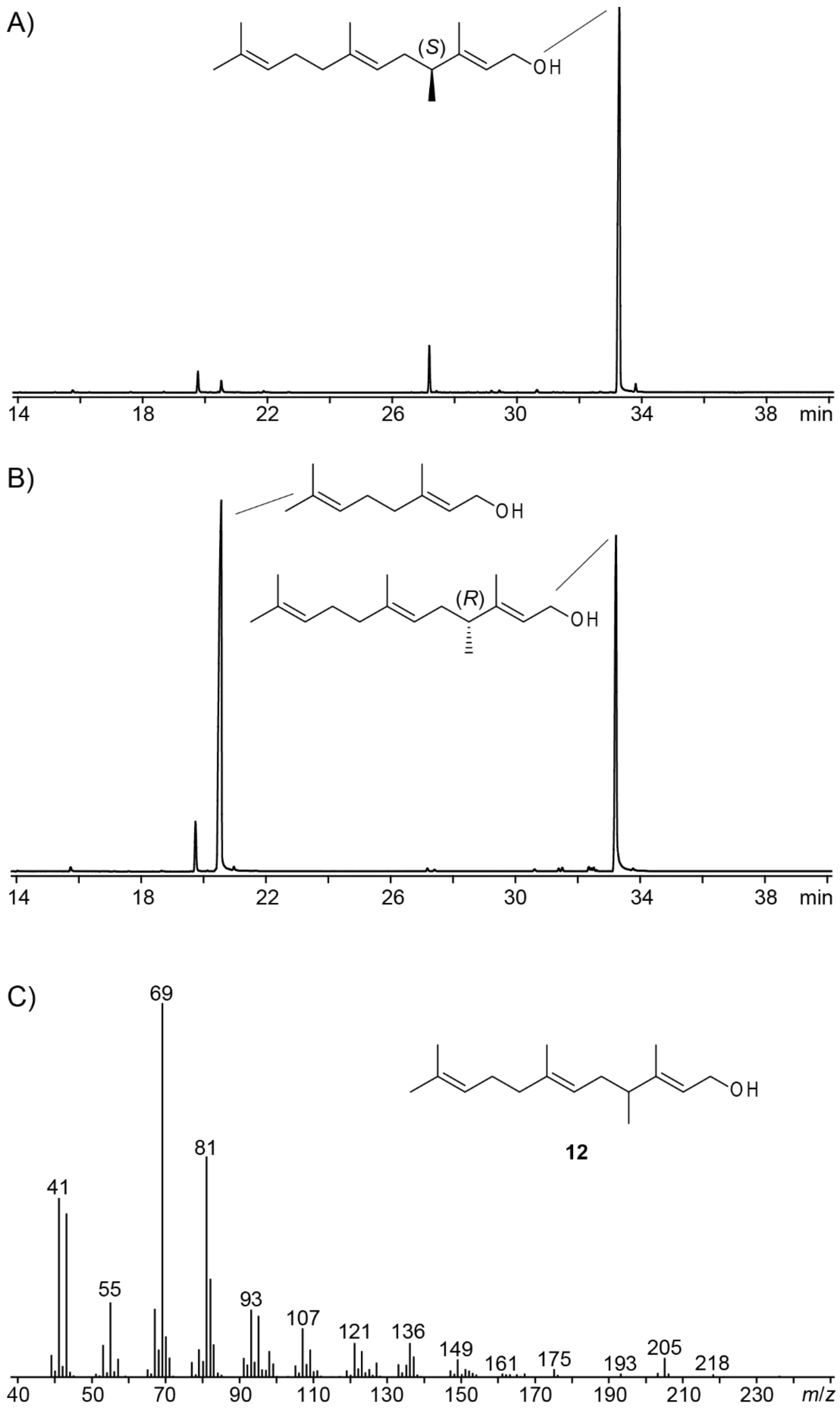

Figure S10. Total ion chromatograms of the products obtained with FPPS after dephosphorylation with CIP from A) GPP and (*E*)-4-methyl-IPP (**8a**) and B) GPP and (*Z*)-4-methyl-IPP (**8b**). C) EI mass spectrum of 4-methylfarnesol (**12**).

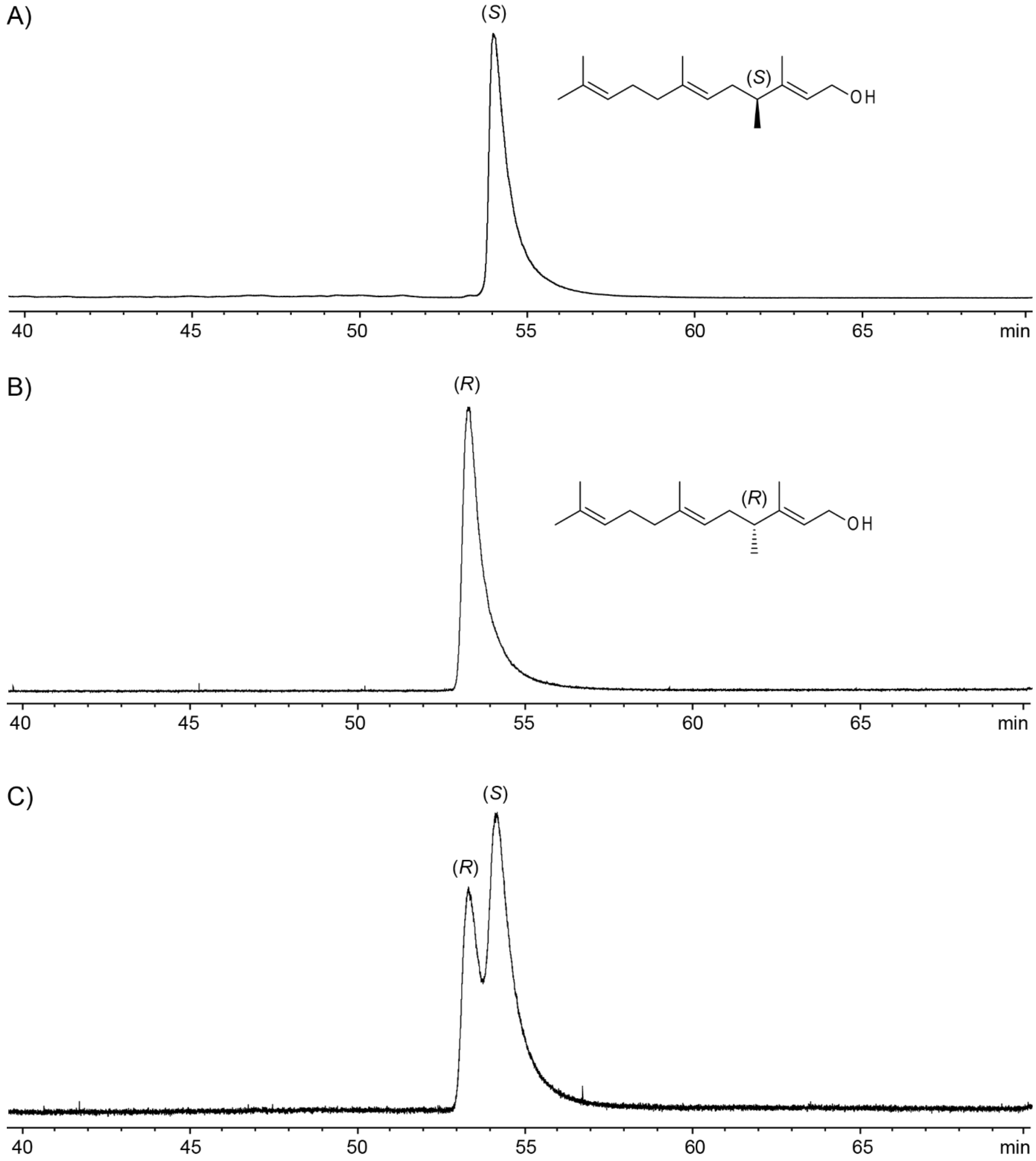

Figure S11. Gas chromatograms on a chiral stationary phase of A) (*S*)-**12a** (99% *ee*) obained from (*E*)-4-methyl-IPP (**8a**), B) (*R*)-**12b** (99% *ee*) obtained from (*Z*)-4-methyl-IPP (**8b**) and C) co-injection of **12ab**.

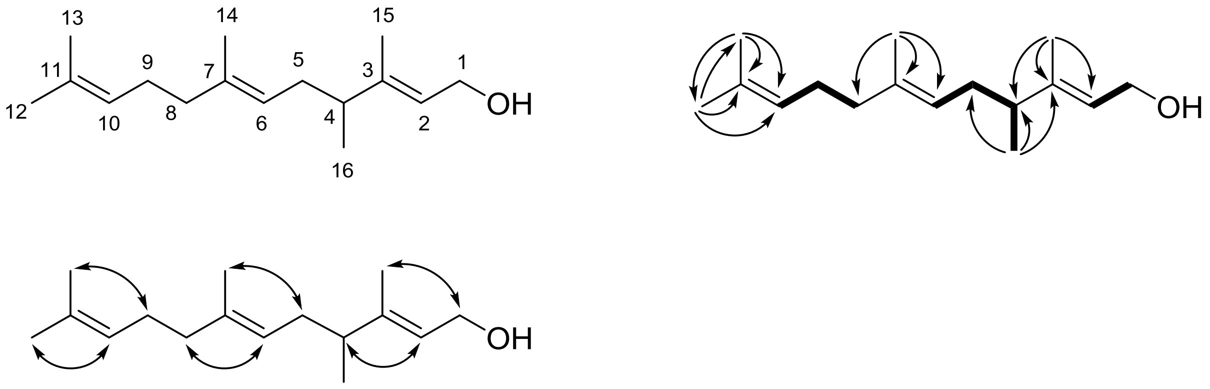

Table S2. NMR data of 4-methylfarnesol (**12**) in C_6D_6 recorded at 298 K.

C[a]		^{13}C[b]	^{1}H[b]
1	CH_2	59.37	3.98 (br t, *J* = 5.6, 2H)
2	CH	124.19	5.40 (br t, *J* = 6.5)
3	C_q	142.39	–
4	CH	43.21	2.09 (m)
5	CH_2	33.86	2.09 (m)
			1.99 (m)
6	CH	123.73	5.20 (m)
7	C_q	135.62	–
8	CH_2	40.27	2.08 (m, 2H)
9	CH_2	27.18	2.16 (m)
10	CH	124.91	5.21 (m)
11	C_q	131.19	–
12	CH_3	25.88	1.68 (br s)
13	CH_3	17.76	1.56 (br s)
14	CH_3	16.24	1.57 (br s)
15	CH_3	13.41	1.44 (br s)
16	CH_3	19.10	0.98 (d, *J* = 6.8)
–	OH	–	0.51 (t, *J* = 5.5)

[a] Carbon numbering as shown in the structure above the table (bold lines: $^{1}H,^{1}H$-COSY correlations, single headed arrows: HMBC correlations, double headed arrows: NOESY correlations). [b] Chemical shifts δ in ppm, multiplicity: s = singlet, d = doublet, m = multiplet, br = broad, coupling constants *J* are given in Hertz.

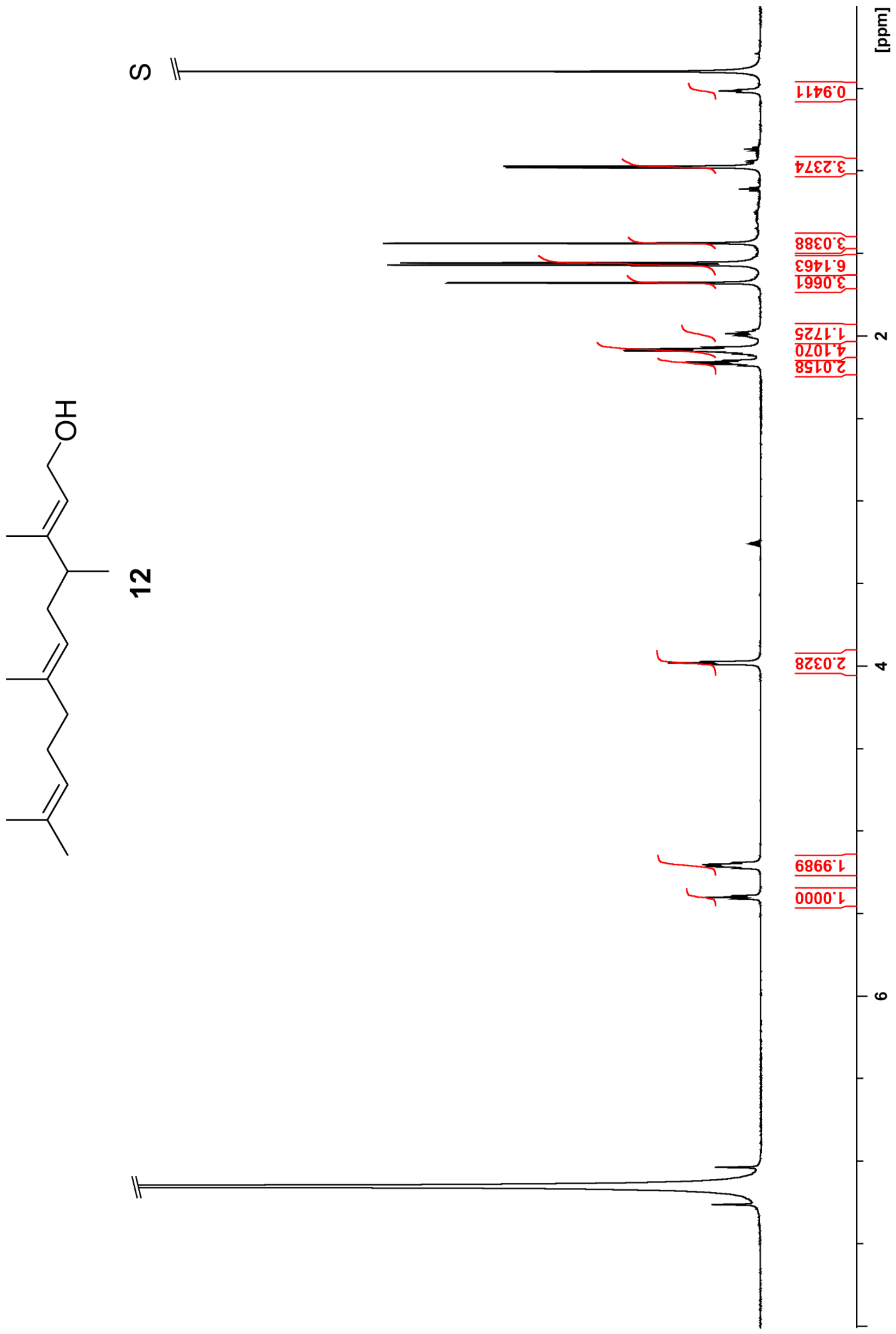

Figure S12. ^{1}H-NMR spectrum of **12** (700 MHz, C_6D_6).

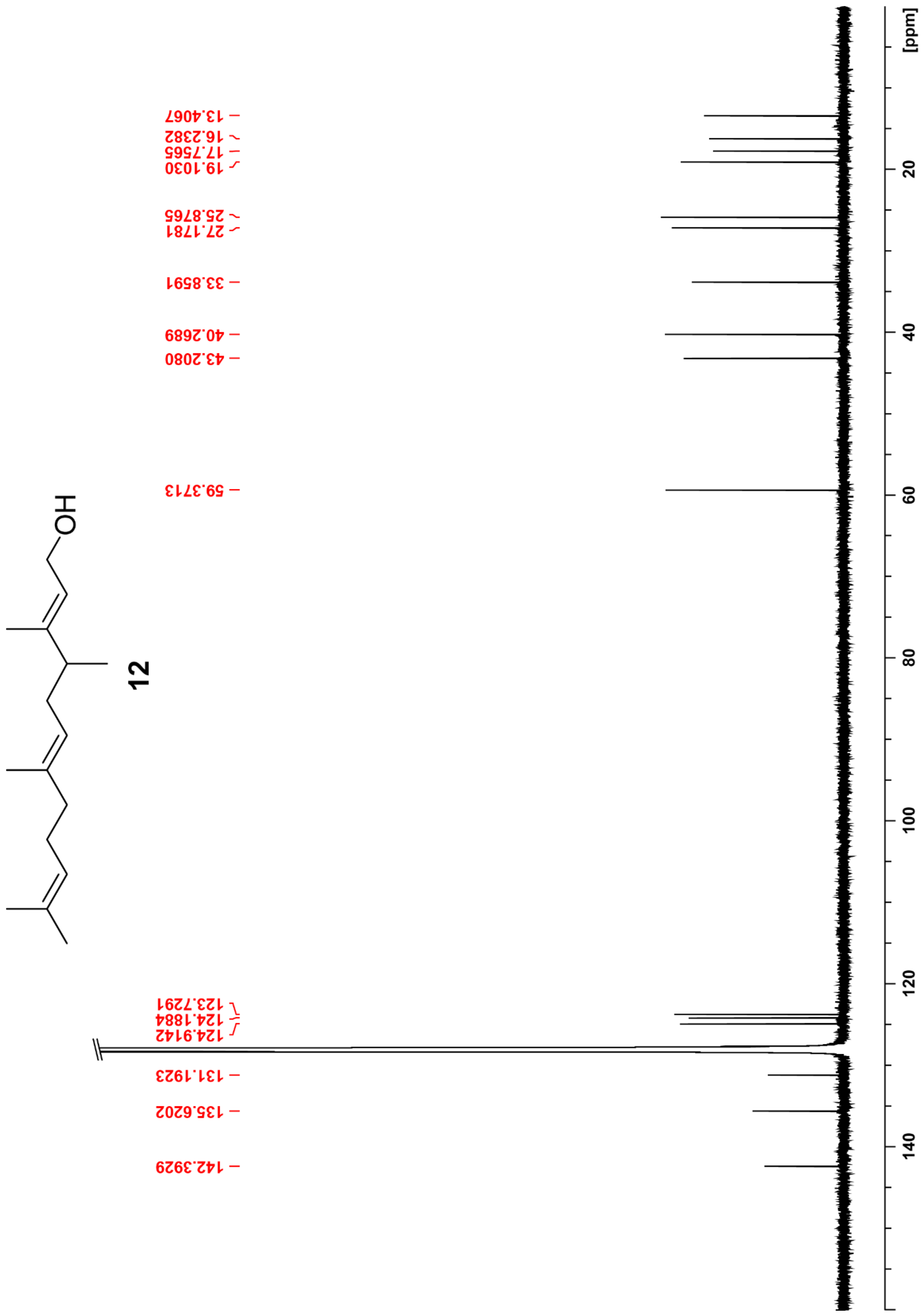

Figure S13. ^{13}C-NMR spectrum of **12** (175 MHz, C_6D_6).

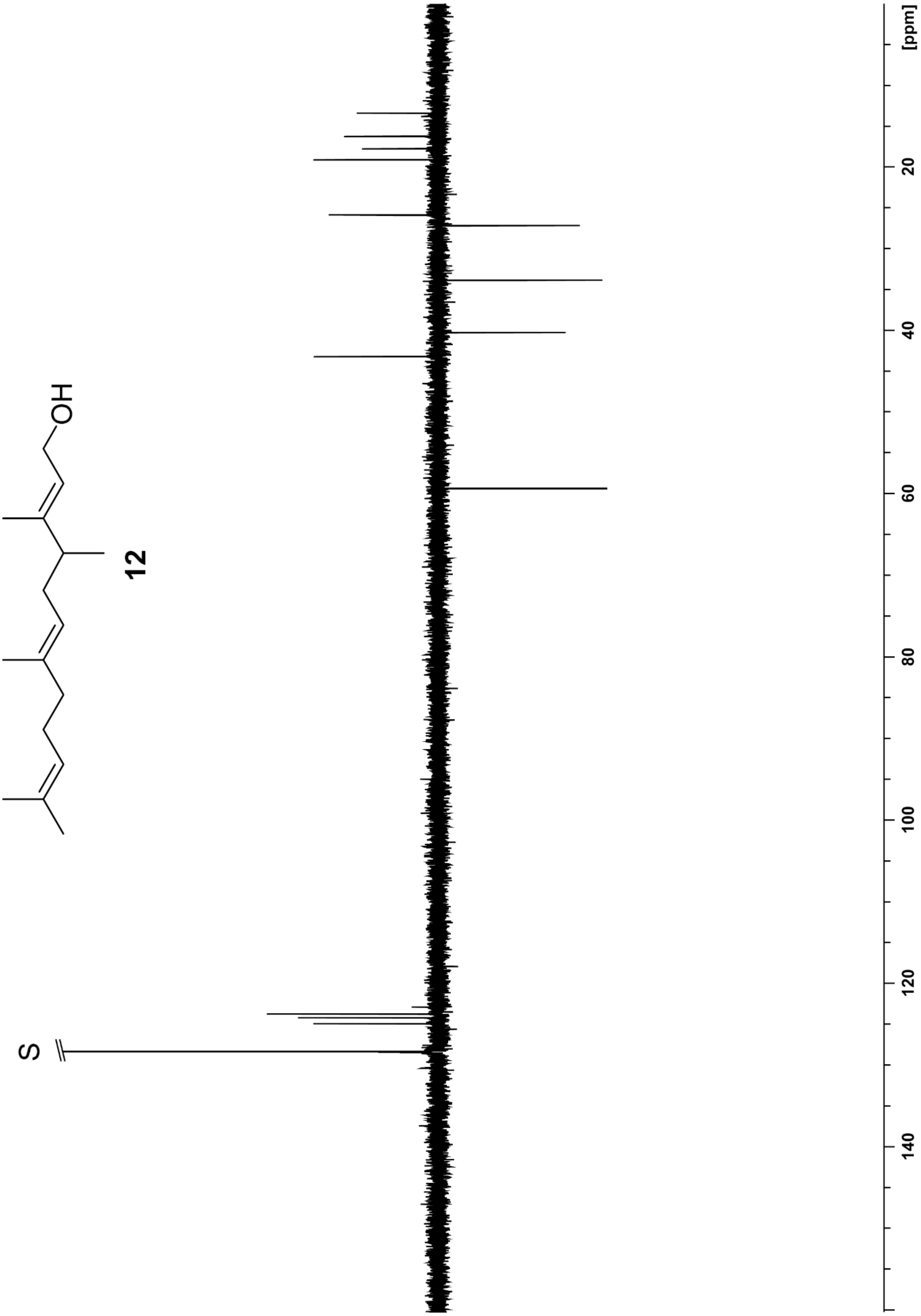

Figure S14. ¹³C-DEPT spectrum of **12** (175 MHz, C_6D_6).

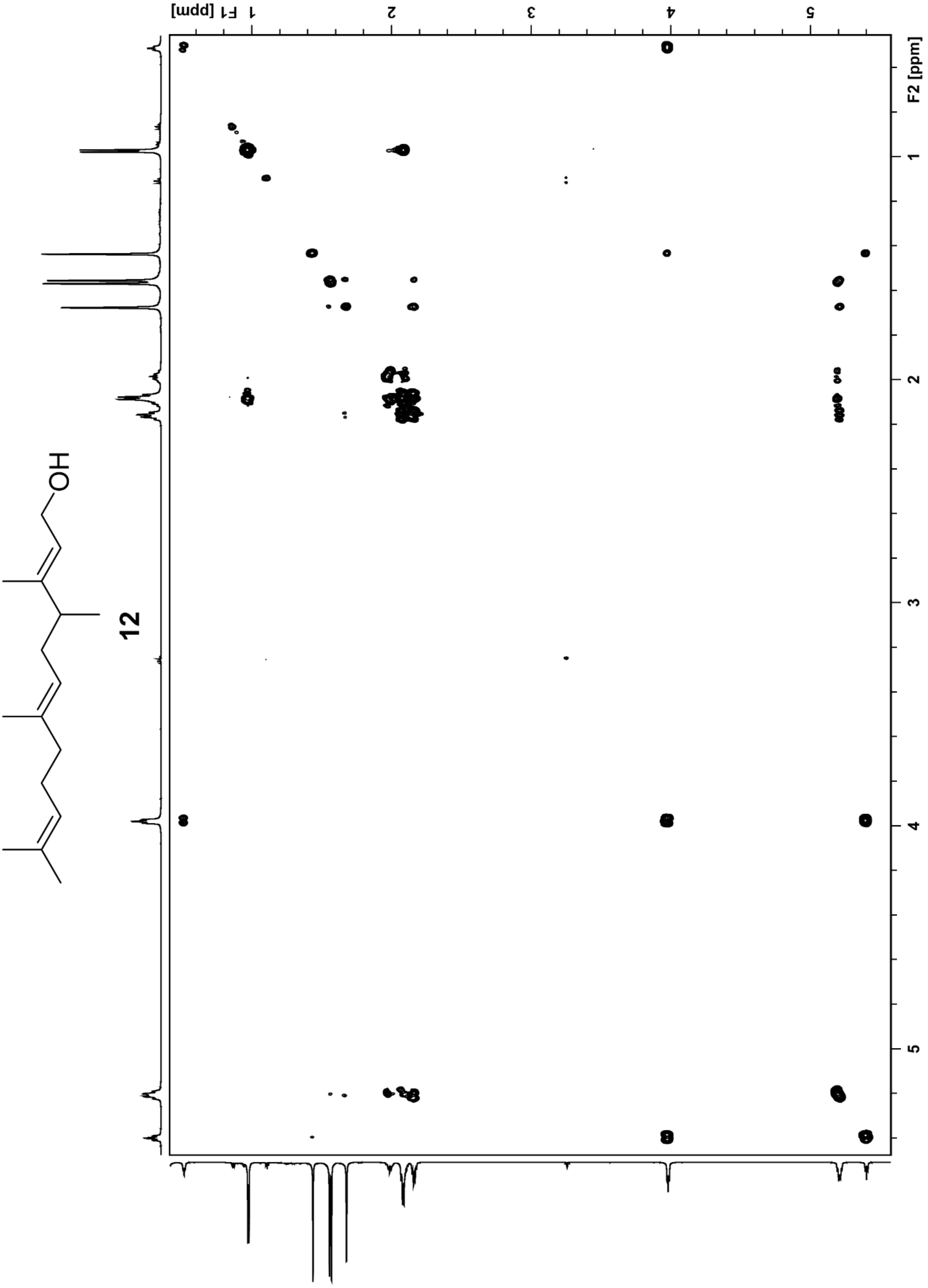

Figure S15. ^{1}H,^{1}H-COSY spectrum of **12** (C_6D_6).

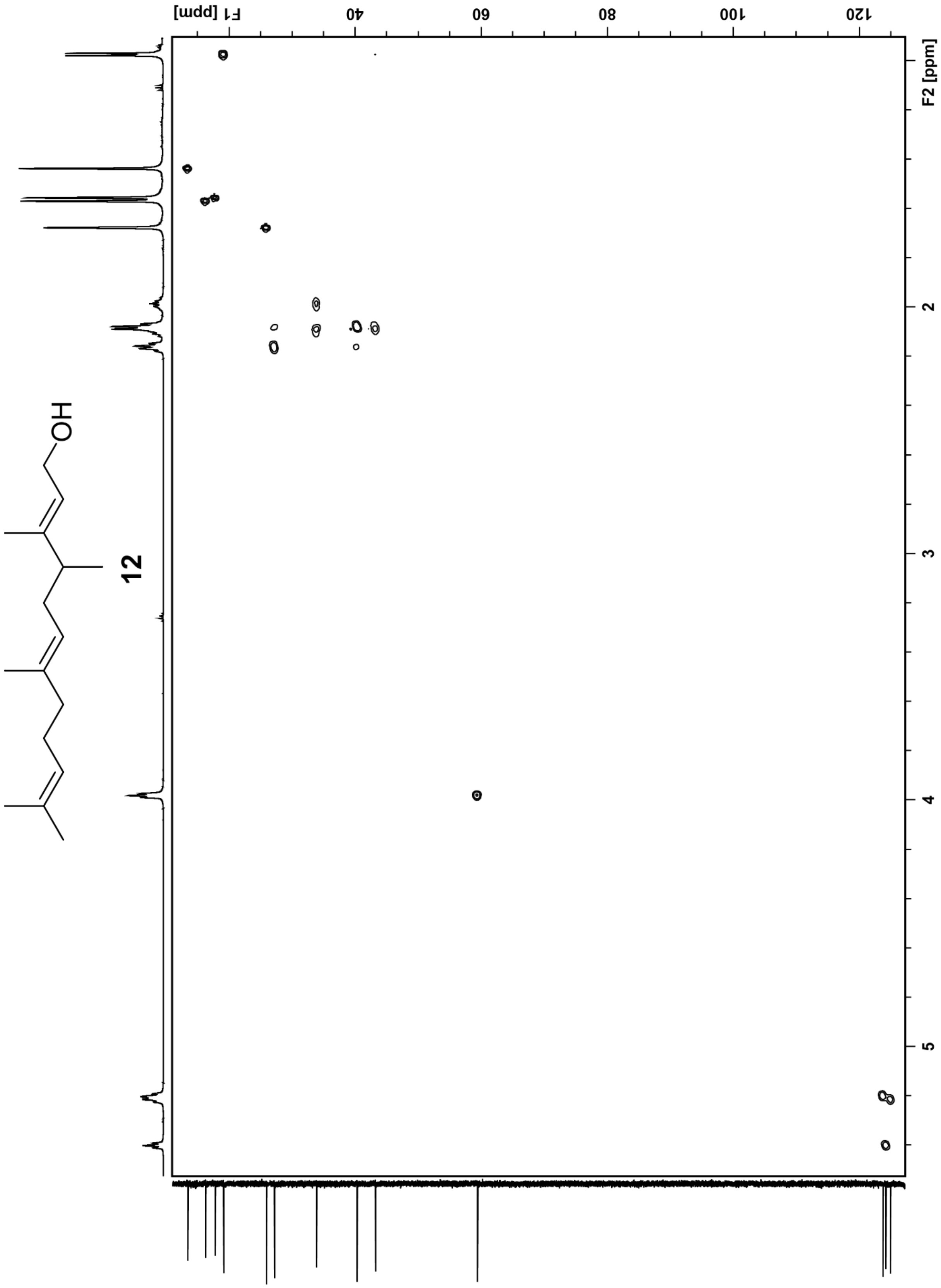

Figure S16. HSQC spectrum of **12** (C_6D_6).

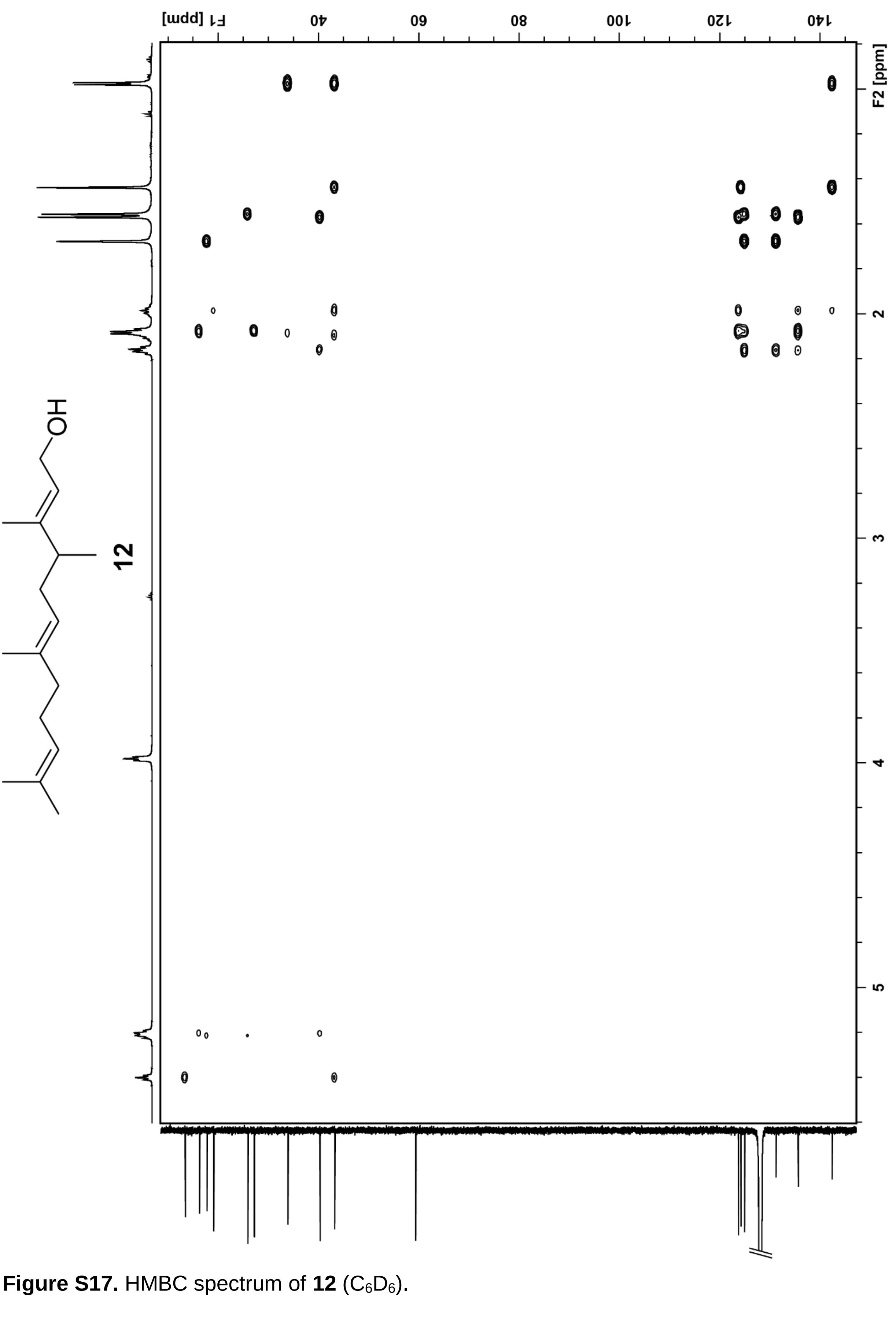

Figure S17. HMBC spectrum of **12** (C_6D_6).

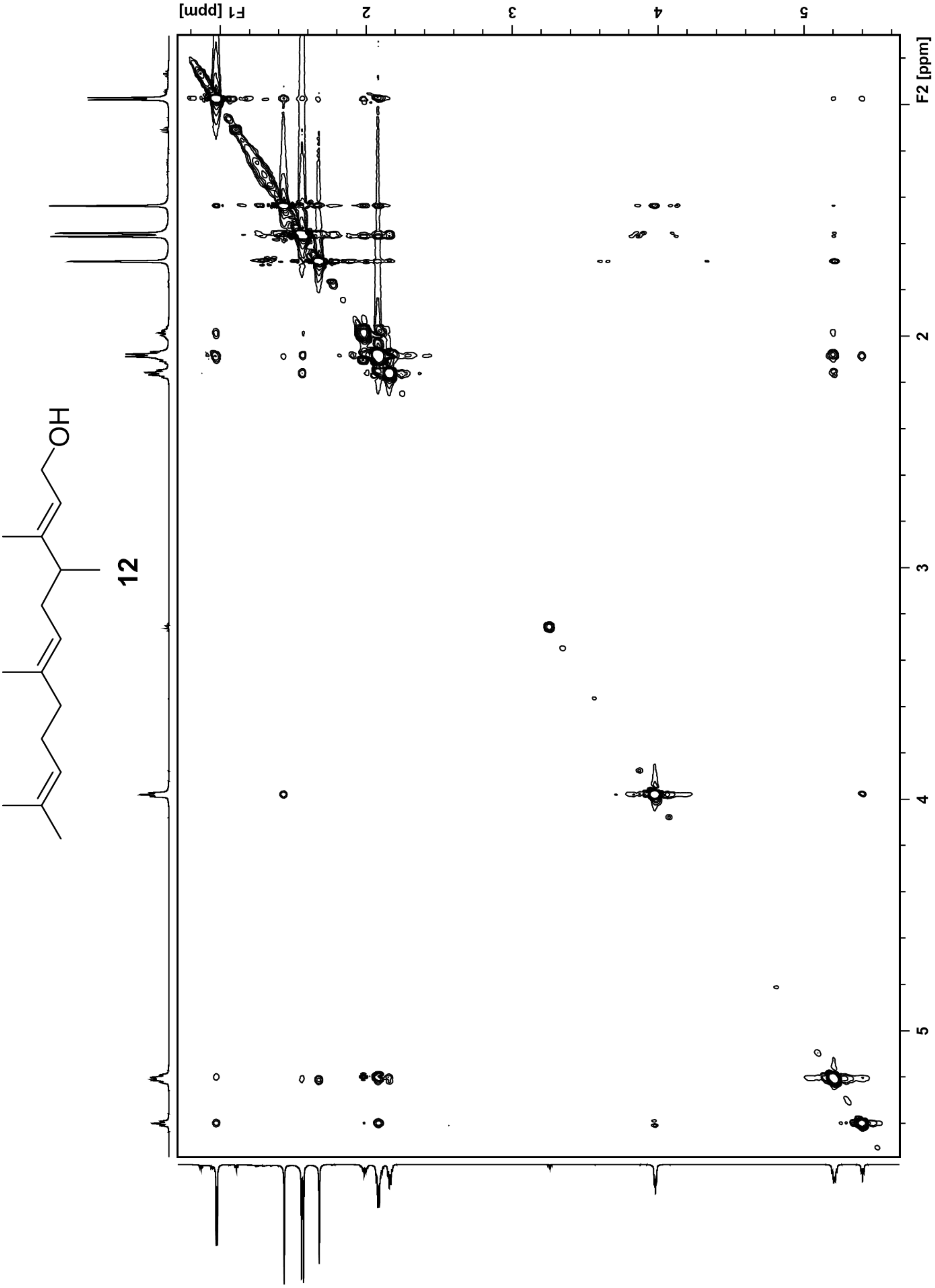

Figure S18. NOESY spectrum of **12** (C_6D_6).

Gene cloning and expression of *Chryseobacterium polytrichastri* Linalool Synthase (CpLS)

Chryseobacterium polytrichastri was grown in 1496 PGY medium (5.0 g tryptone, 3.0 g yeast extract, 1.0 g D-glucose, 1 L deionised water, pH 7.0) at 28 °C for 1 week. Genomic DNA was extracted from *C. polytrichastri* using a standard phenol/chloroform extraction protocol.[5] PCR was performed using the gDNA as template and the primers LL103f (ATGAAAACATCAATTTCAAACGAAGAATTCTATG) and LL103r (CTATTCTCCCTGAGTGATAAATTCAGC) together with Q5®-polymerase (NEB, Ipswich, MA, USA) following a standard protocol. The program used for PCR was initial denaturation at 98 °C for 5 min, followed by a 3-step cycle of denaturation at 98 °C for 15 sec, annealing for 30 sec at a gradient reaching from 55 °C – 57 °C, increasing by 0.5 °C per cycle, and elongation at 72 °C for 1 min, repeated 16 times, followed by another 3-step cycle, repeated 24 times, with the same conditions for denaturation and elongation as before, yet a fixed gradient for the annealing from 63 °C – 65 °C and a final elongation at 72 °C for 5 min. The reaction products were analysed by gel electrophoresis and purified using the Wizard® SV Gel and PCR Clean-Up System (Promega, Madison, WI, USA). The PCR product was used as a template in a second PCR under the same conditions with the primers LL104f (<u>GGCAGCCATATGGCTAGCATGACTGGTGGA</u>ATGAAAACATCAATTTCAAACGAAGAATTCTATG) and LL104r (<u>TCTCAGTGGTGGTGGTGGTGGTGCTCGAGT</u>CTATTCTCCCTGAGTGATAAATTCAGC, homology arms are underlined) to attach homology arms for homologous recombination in yeast with the pYE-Express Shuttle vector.[6] Homologous recombination was performed according to a standard PEG/LiOAc method.[7] The produced plasmid was isolated from yeast using the Zymoprep™ Yeast Plasmid Miniprep II kit (Zymoresearch, Irvine, CA, USA) and was introduced into *E. coli* BL21 (DE3) by electroporation. Cells were spread on LB agar plates (10.0 g tryptone, 5.0 g yeast extract, 5.0 g NaCl, 16.0 g agar, 1 L deionised water) with kanamycin (50 μg/mL) and cultivated at 37 °C overnight. Single colonies were selected and grown in liquid LB medium with kanamycin for 7 h. The plasmid DNA was isolated using PureYield™ Plasmid Miniprep System (Promega, Madison, WI, USA) and the correct size of the insert was verified by analytical digest with the restriction enzymes PvuII and XhoI (NEB, Ipswich, MA, USA). The correct insertion of the gene was also verified by DNA sequencing. An *E. coli* transformant carrying the verified plasmid pYE_CpLS was used for further experiments.

E. coli BL21 carrying the pYE_CpLS plasmid was used to inoculate liquid LB medium containing kanamycin. A preculture was grown overnight at 37 °C and used to inoculate an expression culture in LB Medium (1 mL/L) which was grown at 37 °C until an OD_{600} of 0.4 – 0.6 was reached. The culture was cooled to 18 °C and IPTG (400 mM, 1 mL/L) was added to induce protein expression. The cultures were shaken overnight at 18 °C and cells were harvested by centrifugation (8000 x g, 5 min). Cell pellets were resuspended in cooled binding buffer (10 mL/L culture, 4 °C) and lysed by ultra-sonication (5 x 1 min). The lysate was centrifuged (14.000 x g, 7 min) to remove the cell debris and the cleared lysate was filtered through a syringe filter with loading onto a Ni^{2+}-NTA affinity chromatography column (Super Ni-NTA, Generon, Slough, UK). The column was washed with binding buffer (10 mL/L culture) and the protein was eluted with cooled elution buffer (10 mL/L culture, 4 °C).

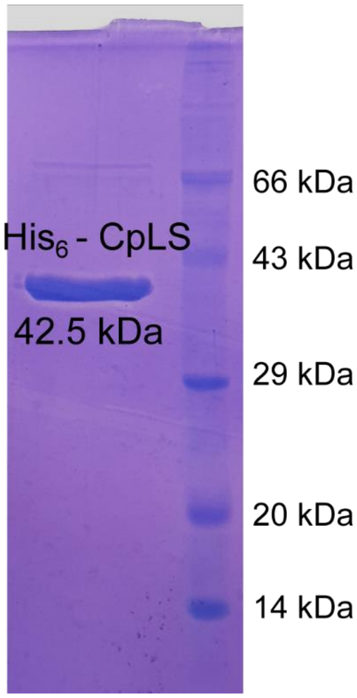

Figure S19. SDS-PAGE analysis of recombinant CpLS from *C. polytrichastri*.

Incubation experiments with recombinant FPPS and CpLS or TmS

TmS was purified by the procedure described before,[8] and the reaction system was similar to the experiments with recombinant FPPS and CIP described above. For small scale test reactions purified FPPS (0.2 mL in elution buffer), CpLS or TmS (0.2 mL in elution buffer), DMAPP or GPP (0.1 mL in 25 mᴍ NH_4HCO_3, 1 mg/mL), **8a** or **8b** (0.1 mL in 25 mᴍ NH_4HCO_3, 2 mg/mL) and incubation buffer (0.5 mL) were mixed and incubated at 28 °C overnight. The product was extracted with hexane (0.2 mL) and the extracts were dried with $MgSO_4$, followed by GC/MS analysis.

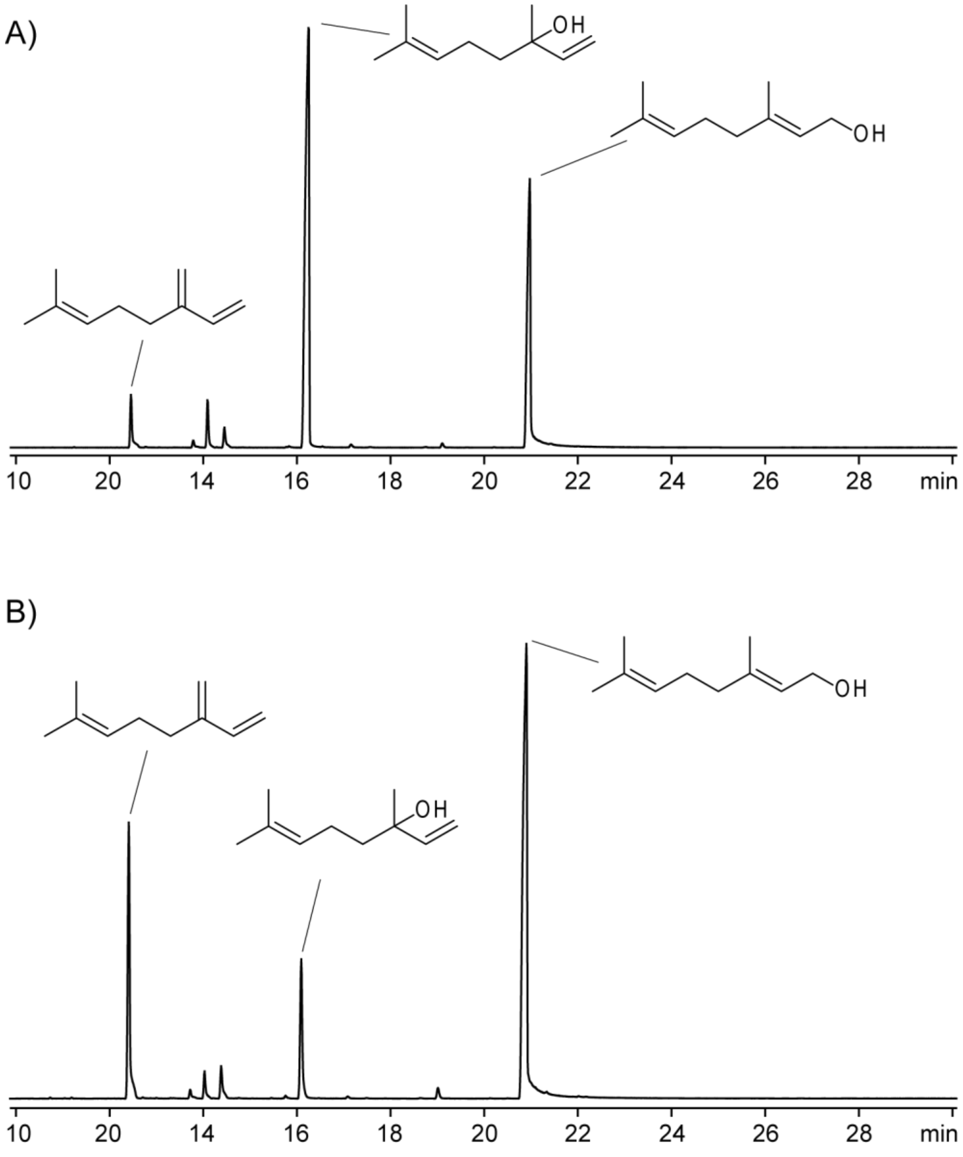

Figure S20. Gas chromatograms on a HP5-MS stationary phase of the products obtained from GPP A) with CpLS and B) with TmS.

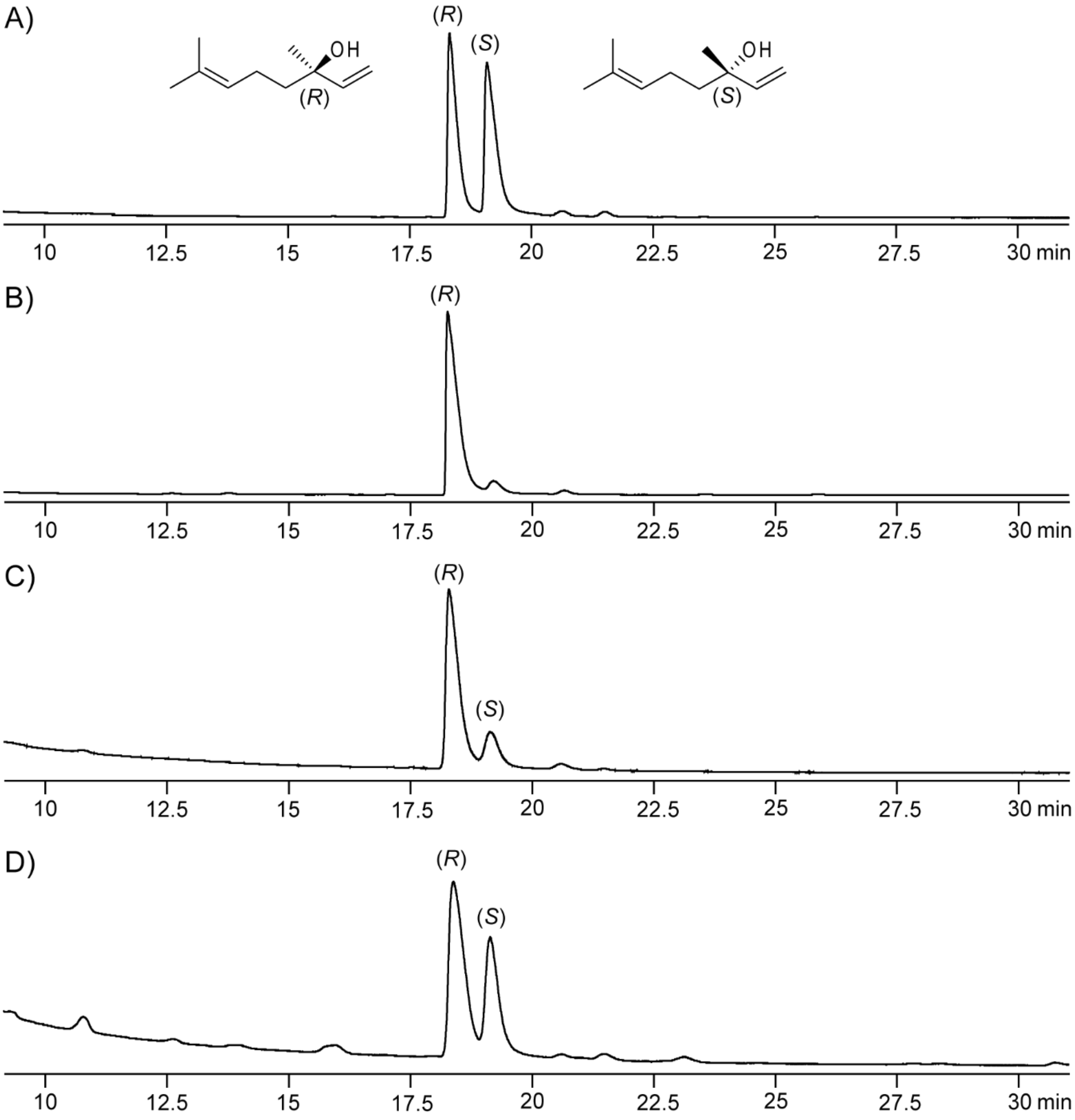

Figure S21. Gas chromatograms on a chiral stationary phase of A) (*rac*)-**13**, B) commercially available (*R*)-**13**, C) **13** obtained from GPP with CpLS, and D) **13** obtained from GPP with TmS.

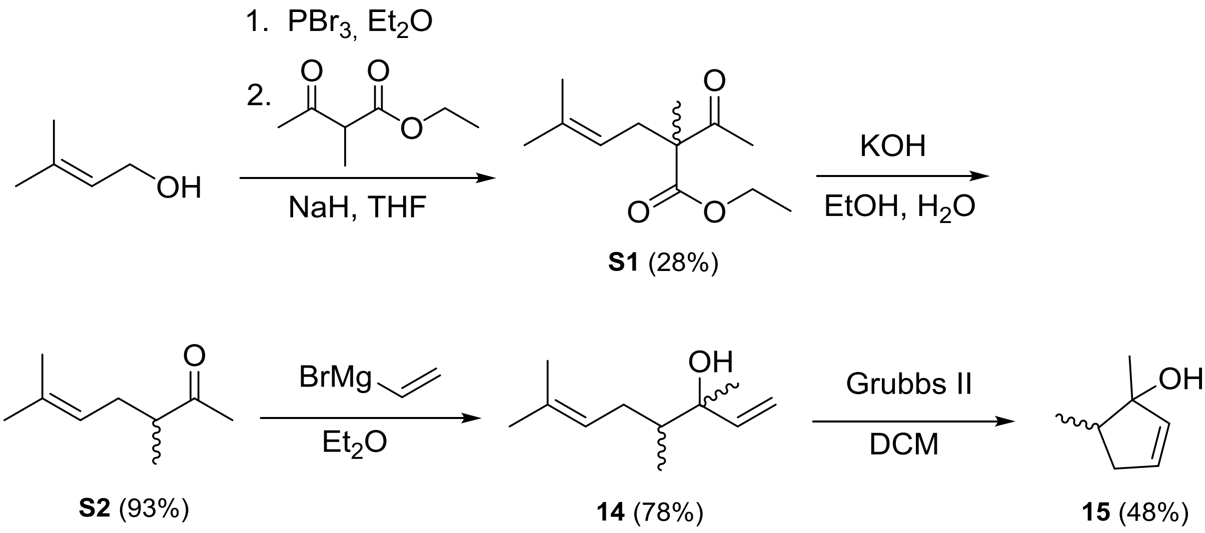

Scheme S1. Synthesis of the mixture of all four stereoisomers of **14** and cyclisation to (1*R**,5*S**)-**15ab** and (1*S**,5*S**)-**15a'b'** using the Grubbs II catalyst.

Synthetic procedures towards (1*R,5*S**)-15ab and (1*S**,5*S**)-15a'b'**

Ethyl 2-acetyl-2,5-dimethylhex-4-enoate (S1)

3-Methyl-2-buten-1-ol (2.07 g, 24.0 mmol) was dissolved in Et_2O (30 mL). After the solution was cooled to 0 °C, PBr_3 (2.8 g, 10.3 mmol, 0.4 eq.) was added dropwise. The mixture was stirred at 0 °C for 1 h and then quenched by pouring onto an ice-water mixture, followed by extraction with Et_2O (3x 50 mL). The combined organic layers were washed with brine and dried with $MgSO_4$. The solvent was evaporated under reduced pressure. The obtained prenyl bromide was of sufficiently quality to be used in the next step without purification.

NaH (60% in mineral oil, 1.15 g, 28.8 mmol, 1.1 eq.) was suspended in THF (200 mL) and the suspension was cooled to 0 °C. Ethyl 2-methylacetoacetate (3.8 g, 26.4 mmol, 1.1 eq.) was added dropwise followed by stirring at room temperature for 1 h. Prenyl bromide was added dropwise and the reaction mixture was stirred overnight at room temperature. The reaction was quenched by adding H_2O (200 mL). The product was extracted with Et_2O (3x 70 mL), the combined organic layers were washed with brine and dried with $MgSO_4$. The solvent was evaporated under reduced pressure. The residue was purified by silica gel chromatography [pentane/Et_2O(10/1), R_f=0.30] to afford **S1** (1.42 g, 6.69 mmol, 28%) as a colourless oil.

Ethyl 2-acetyl-2,5-dimethylhex-4-enoate (S1). ^1H NMR (500 MHz, $CDCl_3$): δ = 4.98 – 4.92 (m, 1H, CH), 4.18 (q, $^3J_{H,H}$= 7.1 Hz, 2H, CH_2), 2.62 – 2.45 (m, 2H, CH_2), 2.14 (s, 3H, CH_3), 1.68 (s, 3H, CH_3), 1.61 (s, 3H, CH_3), 1.30 (s, 3H, CH_3), 1.25 (t, $^3J_{H,H}$ = 7.1 Hz, 3H, CH_3) ppm. ^{13}C NMR (126 MHz, $CDCl_3$): δ = 205.80 (C_q), 173.04 (C_q), 135.67 (C_q), 118.15 (CH), 61.40 (CH_2), 59.97 (C_q), 33.51(CH_2), 26.38 (CH_3), 26.13 (CH_3), 18.97 (CH_3), 18.09 (CH_3), 14.20 (CH_3) ppm. GC: *I* = 1364 (HP-5MS). MS (EI, 70 eV): *m*/*z* (%) = 212 (2), 169 (61), 144 (14), 139 (14), 123 (100), 115 (15), 109 (6), 95 (34), 87 (18), 79 (7), 69 (42), 56 (15), 43 (62).

3,6-Dimethylhept-5-en-2-one (S2)

Compound **S1** (1.42 g, 6.69 mmol) was dissolved in EtOH (18 mL) and a solution of KOH (1.05 g, 18.7 mmol, 2.8 eq.) in H_2O (5.5 mL) was added. The mixture was heated to reflux for 4 h. After cooling to 0 °C, 6 M HCl was added, resulting in the development of CO_2. The product was extracted with Et_2O (3x 30 mL), the combined organic layers were washed with brine and dried with $MgSO_4$. The solvent was evaporated under reduced pressure. The residue was

purified by silica gel chromatography [pentane/Et_2O(10/1), R_f=0.33] to afford **S2** (0.87 g, 6.20 mmol, 93%) as a colourless oil.

3,6-Dimethylhept-5-en-2-one (S2). ^{1}H NMR (700 MHz, C_6D_6): δ = 5.06 – 5.02 (m, 1H, CH), 2.28 – 2.22 (m, 1H, 0.5 CH_2), 2.19 (sext, $^3J_{H,H}$ = 6.9 Hz, 1H, CH), 1.98 – 1.92 (m, 1H, 0.5 CH_2), 1.73 (s, 3H, CH_3), 1.60 (s, 3H, CH_3), 1.48 (s, 3H), 0.90 (d, $^3J_{H,H}$ = 6.9 Hz, 3H, CH_3) ppm. ^{13}C NMR (176 MHz, C_6D_6): δ = 209.62 (C_q), 133.17 (C_q), 122.27 (CH), 47.30 (C_q), 31.70 (CH_3), 27.98 (CH_2), 25.85 (CH_3), 17.78 (CH_3), 15.98 (CH_3) ppm. GC: *I* = 1034 (HP-5MS). MS (EI, 70 eV): *m*/*z* (%) = 140 (12), 125 (16), 122 (30), 107 (16), 85 (21), 72 (52), 69 (81), 55 (73), 43 (100), 41 (72).

3,4,7-Trimethylocta-1,6-dien-3-ol (14)

Vinylmagnesium bromide (1 M in THF, 12.4 mL, 12.4 mmol, 2.0 eq.) was added into Et_2O (50 mL), and the solution was cooled to 0 °C. Then a solution of **S2** (0.87 g, 6.20 mmol) in Et_2O (10 mL) was added dropwise, followed by stirring at room temperature for 4 h. The reaction was quenched by adding sat. aq. NH_4HCO_3 (100 mL) at 0 °C and the product was extracted with Et_2O (3x 80 mL). The combined organic layers were washed with brine and the solvent was evaporated under reduced pressure. The residue was purified by silica gel chromatography to afford **14** (0.82 g, 4.87 mmol, 79%) as a mixture of four stereoisomers. The pure stereoisomers **14a** and **14b'** were isolated by HPLC on a chiral stationary phase (Figure S37).

(3*S*,4*S*)-14a. $[\alpha]_D^{20}$ = –26.4 (CH_2Cl_2, *c* 0.74). NMR data are given in Table S5 and Figures S38–S44. HRMS (ToF): *m*/*z* = 150.1401 ($[M-H_2O]^+$, calc. for $[C_{11}H_{18}]^+$ 150.1403). GC (HP5-MS): *I* = 1184. MS (EI, 70 eV): *m*/*z* (%) = 153 (2), 150 (5), 135 (11), 123 (4), 107 (45), 97 (48), 94 (21), 81 (14), 71 (100), 55 (97), 43 (45). IR (diamond ATR): $\tilde{\nu}$ / cm^{-1} = 3457 (w), 2968 (m), 2916 (m), 2879 (m), 1452 (m), 1412 (m), 1375 (w), 1174 (w), 1120 (m), 995 (m), 917 (s), 871 (w), 831 (w), 691 (w), 555 (w), 444 (w).

(3*S*,4*R*)-14b'. $[\alpha]_D^{20}$ = –5.3 (CH_2Cl_2, *c* 0.62). NMR data are given in Table S6 and Figures S45–S51. HRMS (ToF): *m*/*z* = 150.1403 ($[M-H_2O]^+$, calc. for $[C_{11}H_{18}]^+$ 150.1403). GC (HP5-MS): *I* = 1184. MS (EI, 70 eV): *m*/*z* (%) = 153 (2), 150 (6), 135 (10), 123 (4), 107 (45), 97 (49), 94 (16), 81 (14), 71 (100), 55 (98), 43 (45). IR (diamond ATR): $\tilde{\nu}$ / cm^{-1} = 3449 (w), 2968 (m), 2916 (m), 2879 (m), 1452 (m), 1412 (m), 1375 (m), 1119 (m), 1070 (m), 996 (m), 917 (s), 869 (w), 831 (w), 776 (w), 688 (w), 445 (w).

1,5-Dimethylcyclopent-2-en-1-ol (15)

The mixture of four stereoisomers of **14** (220 mg, 1.30 mmol) and Grubbs Catalyst® (2nd generation, 20 mg, 0.023 mmol, 0.018 eq.) were dissolved in DCM (3 mL).[9] After stirring at room temperature for 2 h, the solvent was evaporated by reduced pressure and the residue was purified by the silica gel chromatography [pentane/Et_2O (2/1)] to afford the product **15ab** (50 mg, 0.44 mmol, 34% yield) and **15a'b'** (20 mg, 0.18 mmol, 14% yield) as colourless oils.

(1*R,5*S**)-15ab.** NMR data are given in Table S3 and Figures S23-S29. HRMS (ToF): *m*/*z* = 111.0808 ($[M-H]^+$, calc. for $[C_7H_{11}O]^+$ 111.0804). GC (HP5-MS): *I* = 841. MS (EI, 70 eV): *m*/*z* (%) = 112 (31), 97 (100), 91 (5), 79 (27), 69 (25), 58 (7), 55 (18), 43 (78). IR (diamond ATR): $\tilde{\nu}$ / cm^{-1} = 3352 (m), 3052 (w), 2961 (m), 2925 (m), 2873 (w), 2841 (w), 1463(w), 1445 (m), 1376 (m), 1362 (m), 1340 (m), 1185 (m), 1130 (s), 1082 (m), 1060 (m), 1010 (m), 960 (m), 925 (m), 905 (m), 728 (s), 688 (w), 578 (w), 539(w).

(1*S,5*S**)-15a'b'.** NMR data are given in Table S4 and Figures S30-S36. HRMS (ToF): *m*/*z* = 111.0807 ($[M-H]^+$, calc. for $[C_7H_{11}O]^+$ 111.0804). GC (HP5-MS): *I* = 821. MS (EI, 70 eV): *m*/*z* (%) = 112 (28), 97 (100), 91 (4), 79 (25), 69 (27), 58 (7), 55 (19), 43 (78). IR (diamond ATR): $\tilde{\nu}$ / cm^{-1} = 3367 (m), 3052 (w), 2964 (m), 2927 (m), 2908 (m), 2873 (w), 2846 (w), 1454 (m), 1366 (m), 1325 (w), 1294 (w), 1264 (w), 1181 (m), 1150 (m), 1124 (m), 1089 (s), 1058 (m), 1010 (m), 961 (m), 908 (s), 868 (m), 768 (m), 725 (s), 599 (w), 538 (w), 495 (w).

The purified (3*S*,4*S*)-**14a** and (3*S*,4*R*)-**14b'** from chiral HPLC (Figure S37) was converted to (1*R*,5*S*)-**15a** and (1*R*,5*R*)-**15b'** by the same mothed for analysis their specific rotation.
(1*R*,5*S*)-**15a**. $[\alpha]_D^{20}$ = +13.5 (CH_2Cl_2, *c* 0.6).
(1*R*,5*R*)-**15b'**. $[\alpha]_D^{20}$ = +5.2 (CH_2Cl_2, *c* 0.5).

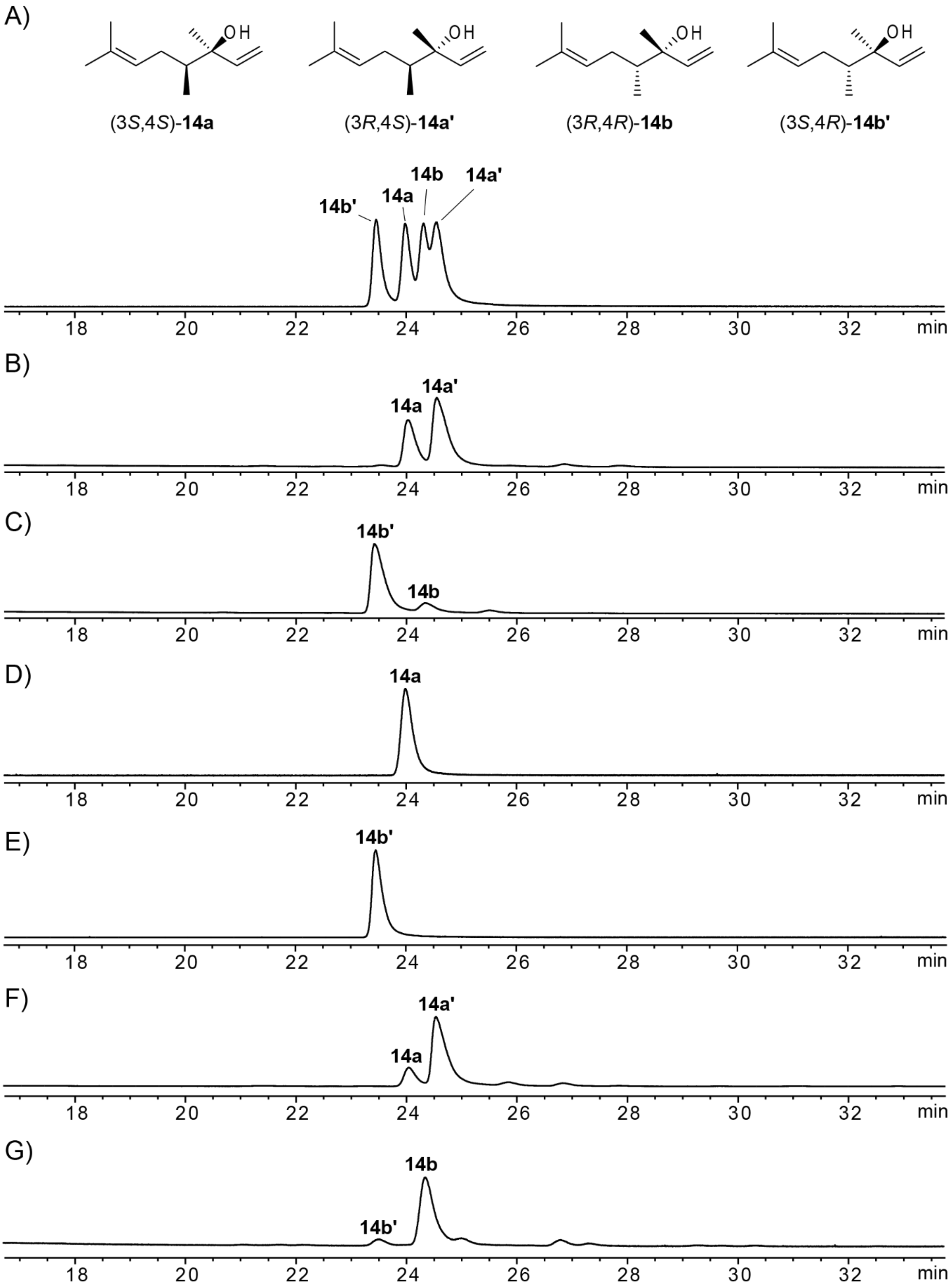

Figure S22. Gas chromatograms on a chiral stationary phase of A) synthetic mixture of all four stereoisomers of **14**, B) (3*S*,4*S*)-**14a** and (3*R*,4*S*)-**14a'** obtained from DMAPP and **8a** with FPPS and CpLS, C) (3*R*,4*R*)-**14b** and (3*S*,4*R*)-**14b'** obtained from DMAPP and **8b** with FPPS and CpLS, D) (3*S*,4*S*)-**14** and E) (3*S*,4*R*)-**14** both purified from the synthetic mixture of four stereoisomers by HPLC on a chiral stationary phase, F) (3*S*,4*S*)-**14a** and (3*R*,4*S*)-**14a'** obtained from DMAPP and **8a** with FPPS and TmS, and G) (3*R*,4*R*)-**14b** and (3*S*,4*R*)-**14b'** obtained from DMAPP and **8b** with FPPS and TmS.

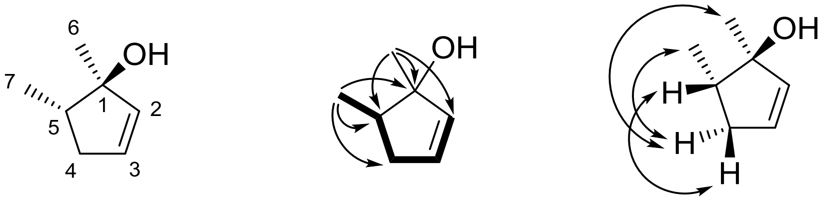

Table S3. NMR data of (1*R**,5*S**)-**15ab** in C_6D_6 recorded at 298 K.

C[a]		^{13}C[b]	^{1}H[b]
1	C_q	83.75	–
2	CH	140.25	5.58 (ddd, *J* = 5.7, 2.1, 1.6)
3	CH	130.15	5.53 (ddd, *J* = 5.7, 2.7, 2.1)
4	CH_2	39.45	2.28 (dddd, *J* = 16.1, 7.7, 2.7, 1.6)
			1.62 (dddd, *J* = 16.1, 7.9, 2.1, 2.1)
5	CH	45.92	1.91 (sext, *J* = 7.4)
6	CH_3	21.94	1.03 (s)
7	CH_3	14.35	0.89 (d, *J* = 7.1)
–	OH	–	0.86 (br s)

[a] Carbon numbering as shown in the structure above the table (bold lines: $^{1}H,^{1}H$-COSY correlations, single headed arrows: HMBC correlations, double headed arrows: NOESY correlations). [b] Chemical shifts δ in ppm, multiplicity: s = singlet, d = doublet, sext = sextet, br = broad, coupling constants *J* are given in Hertz.

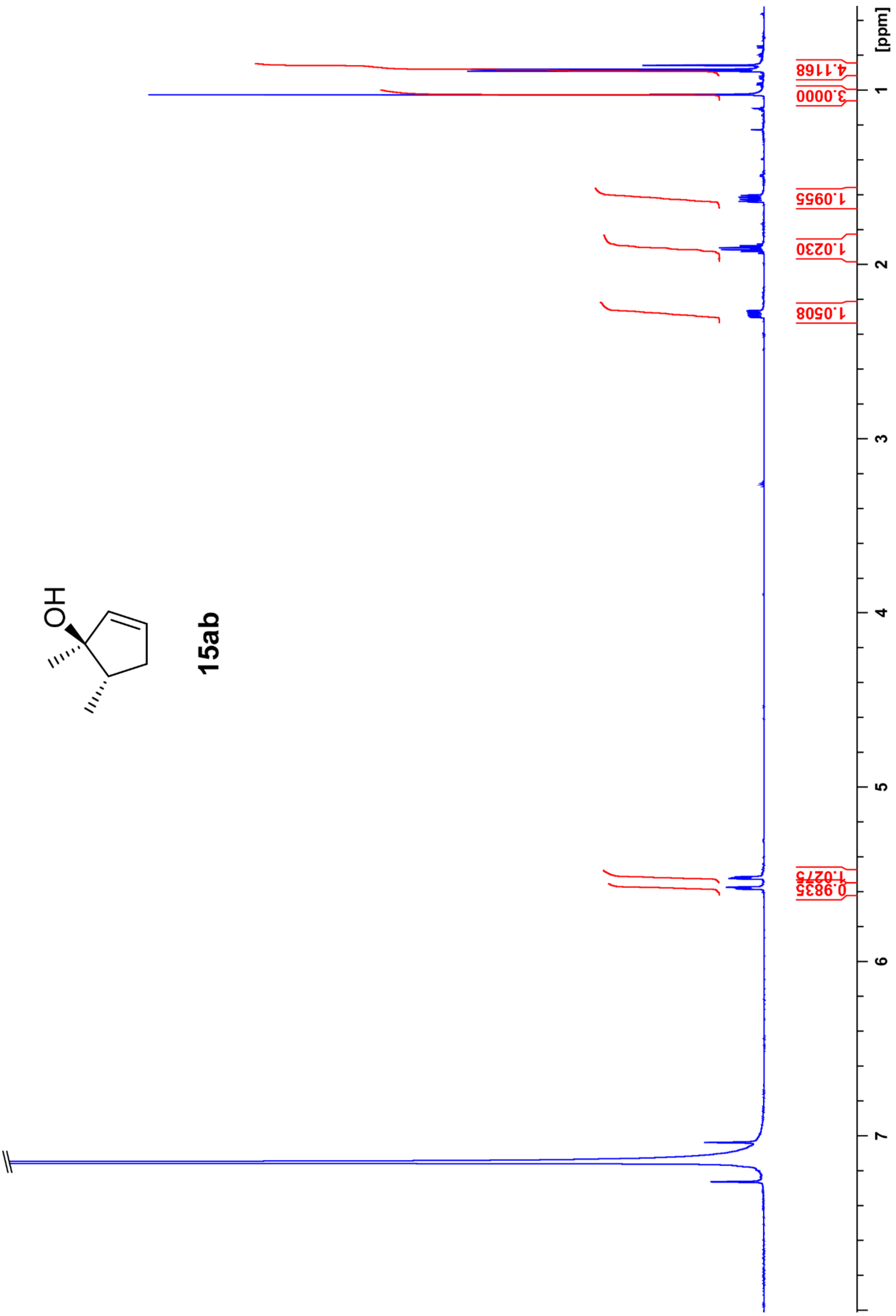

Figure S23. ^{1}H-NMR spectrum of **15ab** (700 MHz, C_6D_6).

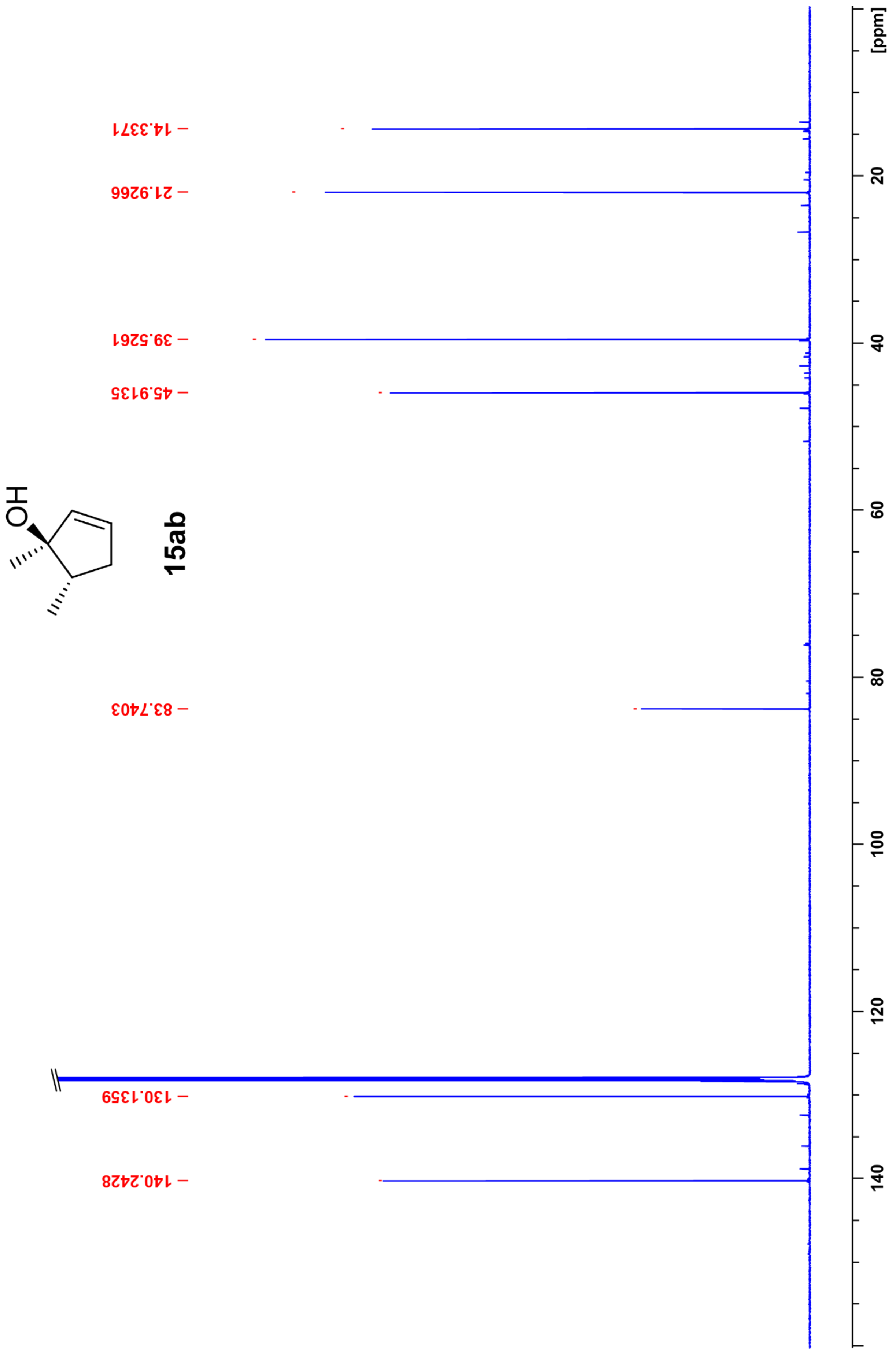

Figure S24. ^{13}C-NMR spectrum of **15ab** (175 MHz, C_6D_6).

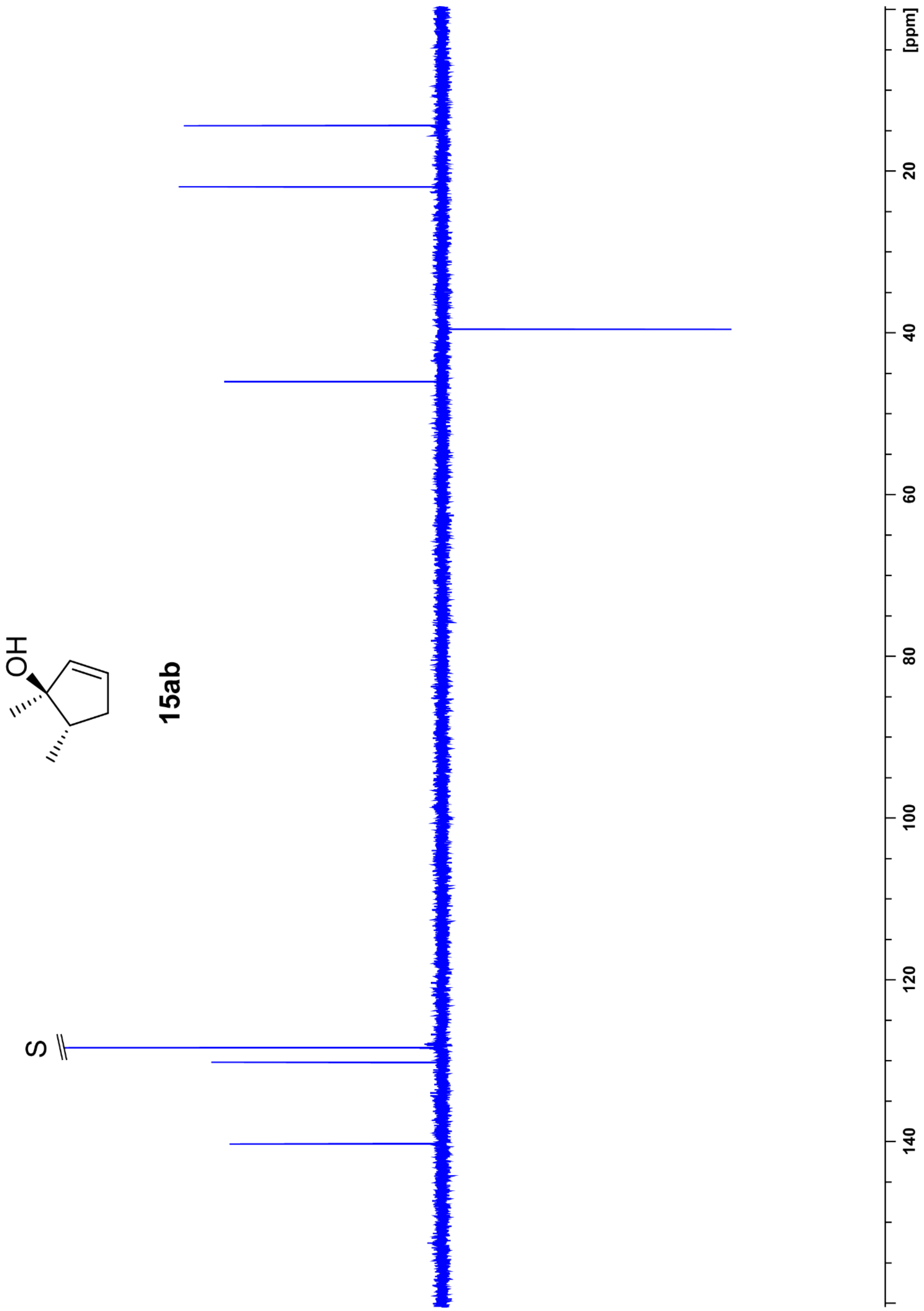

Figure S25. ^{13}C-DEPT spectrum of **15ab** (175 MHz, C_6D_6).

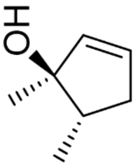

15ab

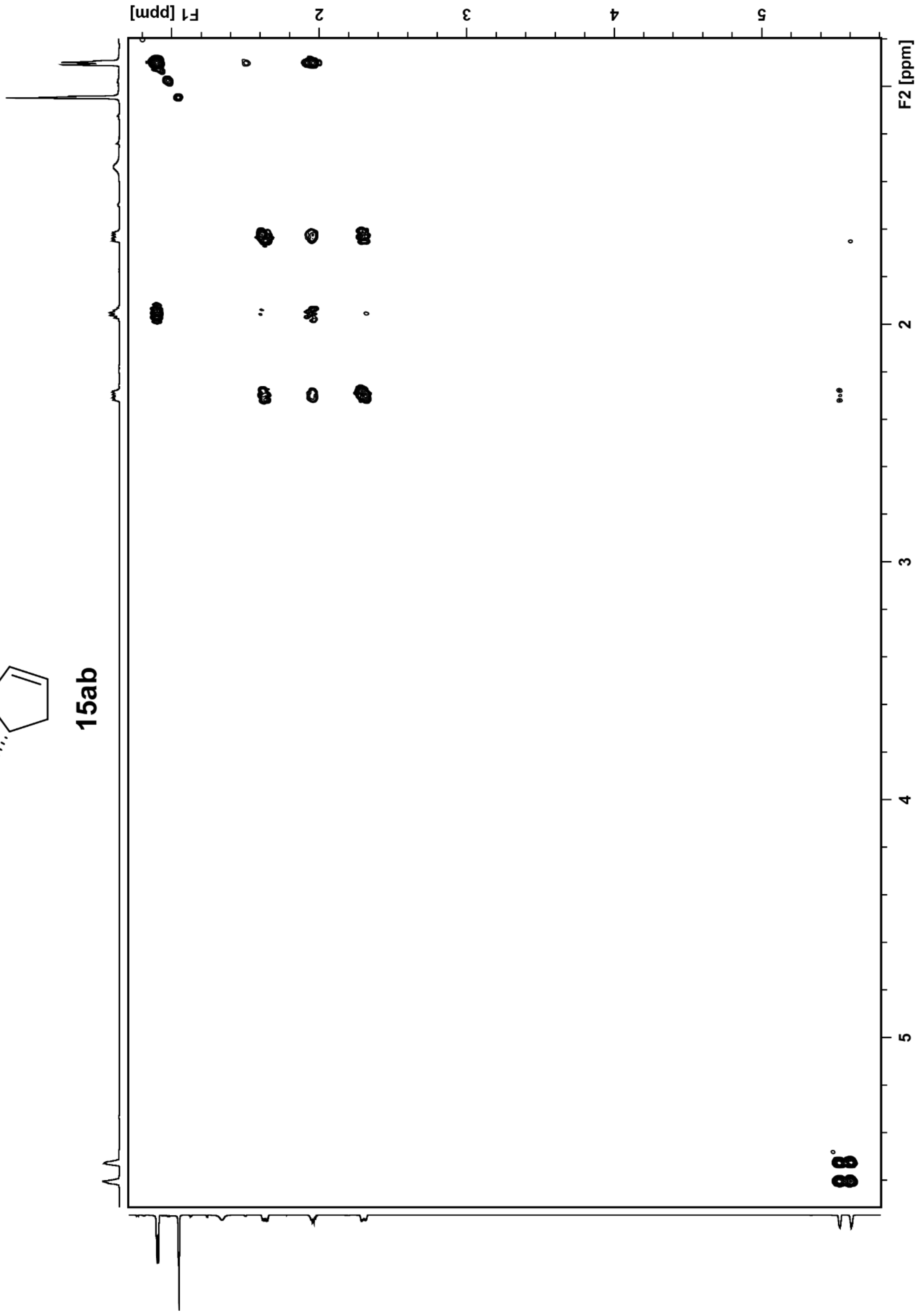

Figure S26. $^{1}H,^{1}H$-COSY spectrum of **15ab** (C_6D_6).

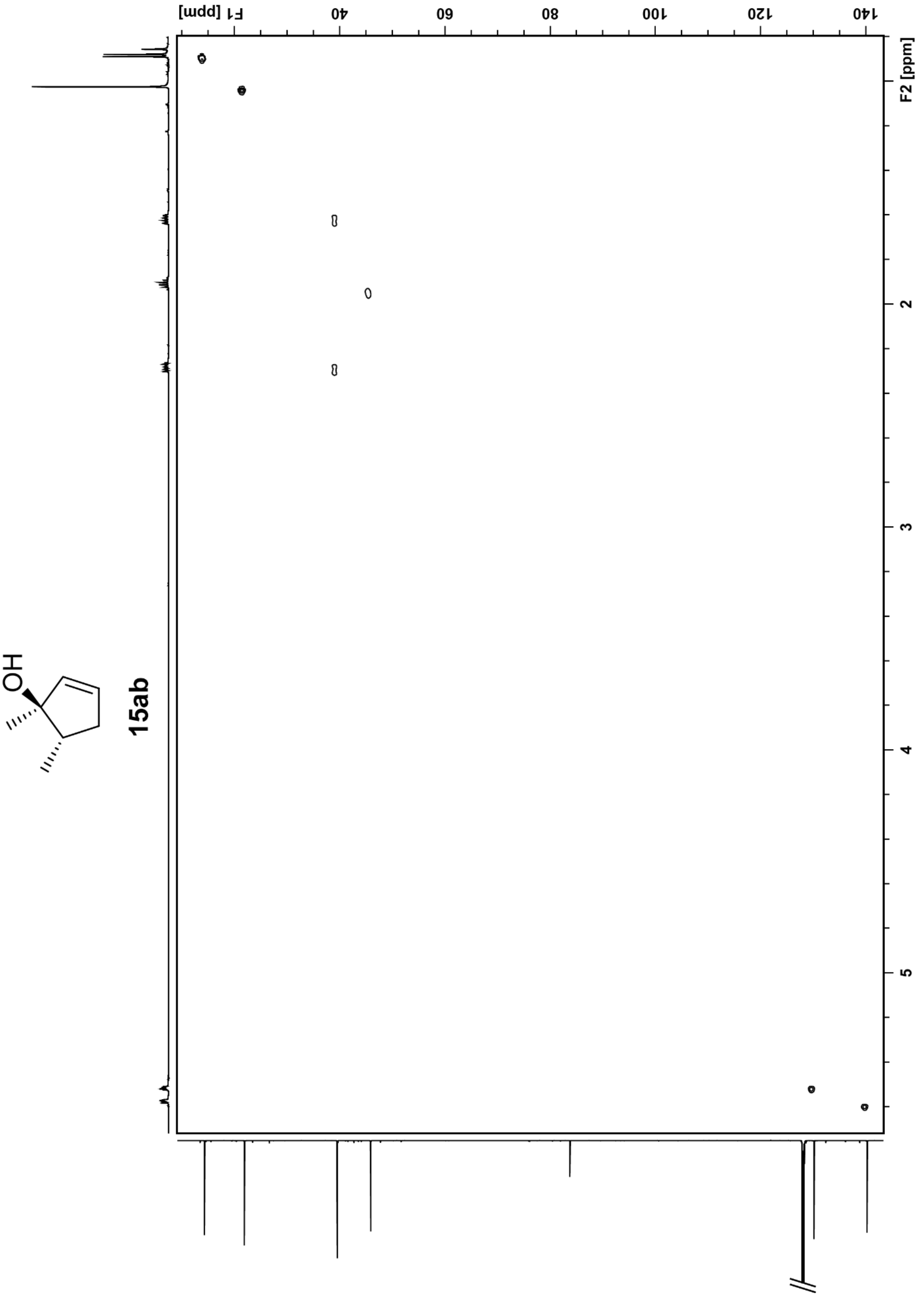

Figure S27. HSQC spectrum of **15ab** (C_6D_6).

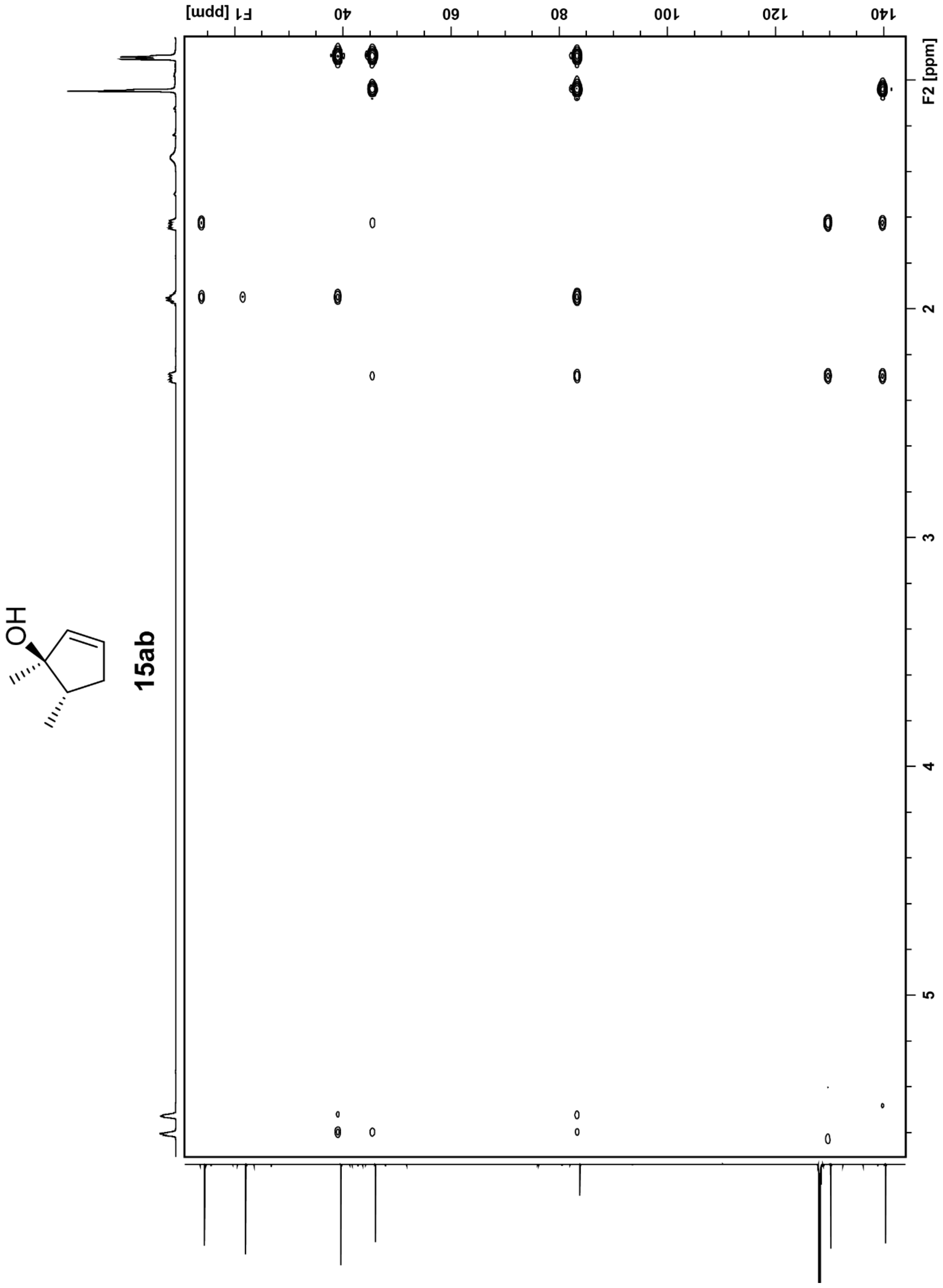

Figure S28. HMBC spectrum of **15ab** (C_6D_6).

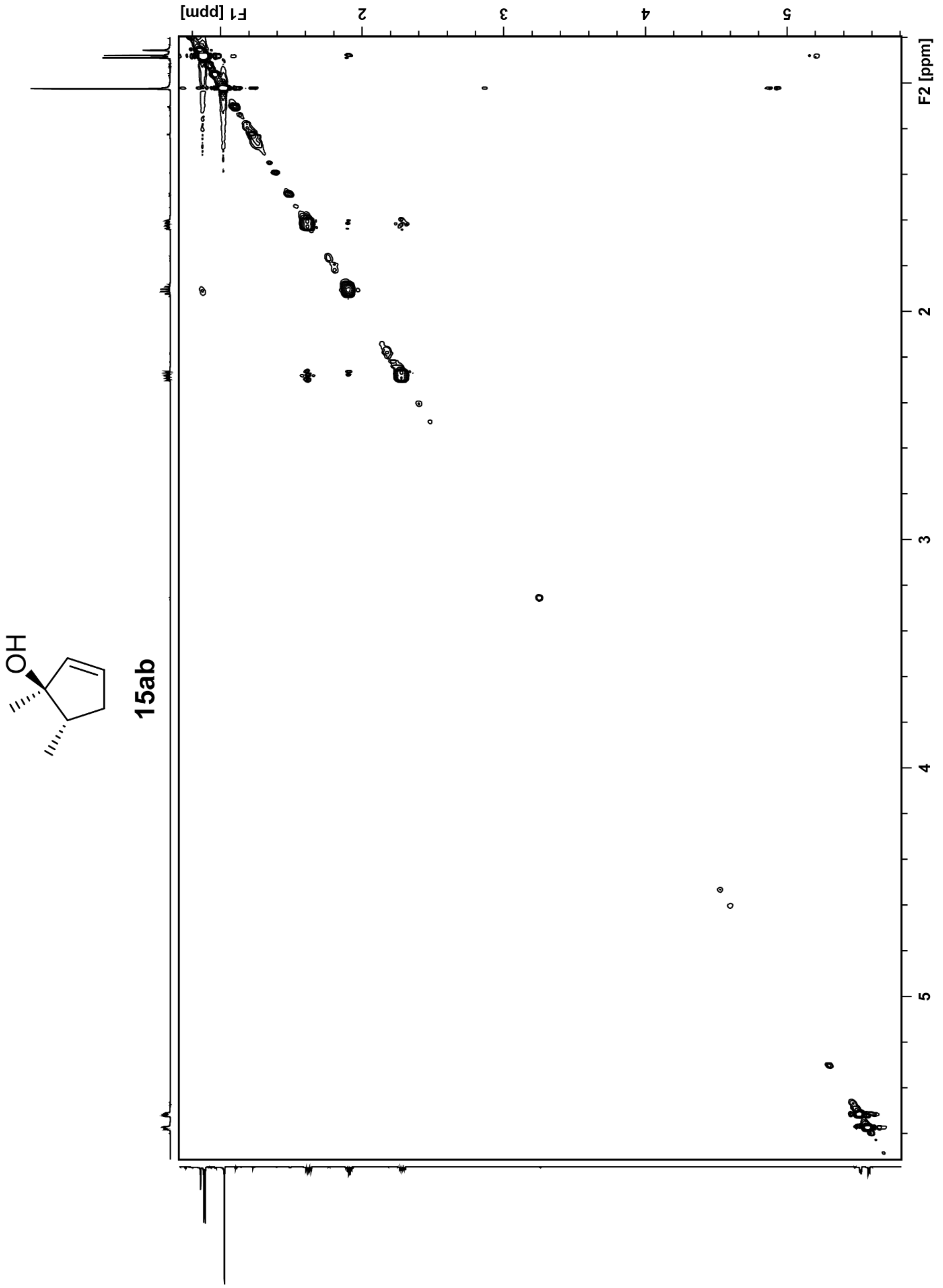

Figure S29. NOESY spectrum of **15ab** (C_6D_6).

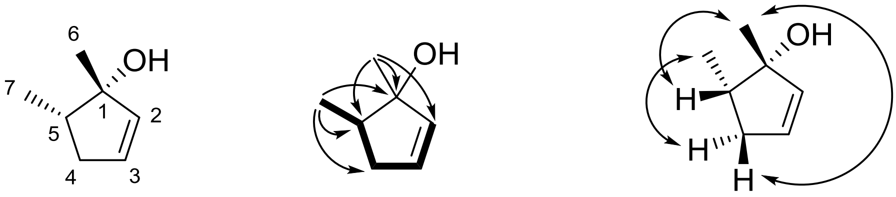

Table S4. NMR data of (1*S**,5*S**)-**15a'b'** in C_6D_6 recorded at 298 K.

C[a]		^{13}C[b]	^{1}H[b]
1	C_q	81.94	–
2	CH	138.83	5.60 (ddd, *J* = 5.7, 2.1, 1.7)
3	CH	132.37	5.58 (ddd, *J* = 5.7, 2.8, 2.0)
4	CH_2	39.72	2.19 (dddd, *J* = 16.4, 7.5, 2.5, 1.5)
			1.81 (dddd, *J* = 16.4, 7.3, 2.2, 1.9)
5	CH	42.74	1.76 (sext, *J* = 7.1)
6	CH_3	26.71	1.23 (s)
7	CH_3	13.54	0.96 (d, *J* = 6.9)
–	OH	–	0.56 (br s)

[a] Carbon numbering as shown in the structure above the table (bold lines: $^{1}H,^{1}H$-COSY correlations, single headed arrows: HMBC correlations, double headed arrows: NOESY correlations). [b] Chemical shifts δ in ppm, multiplicity: s = singlet, d = doublet, sext = sextet, br = broad, coupling constants *J* are given in Hertz.

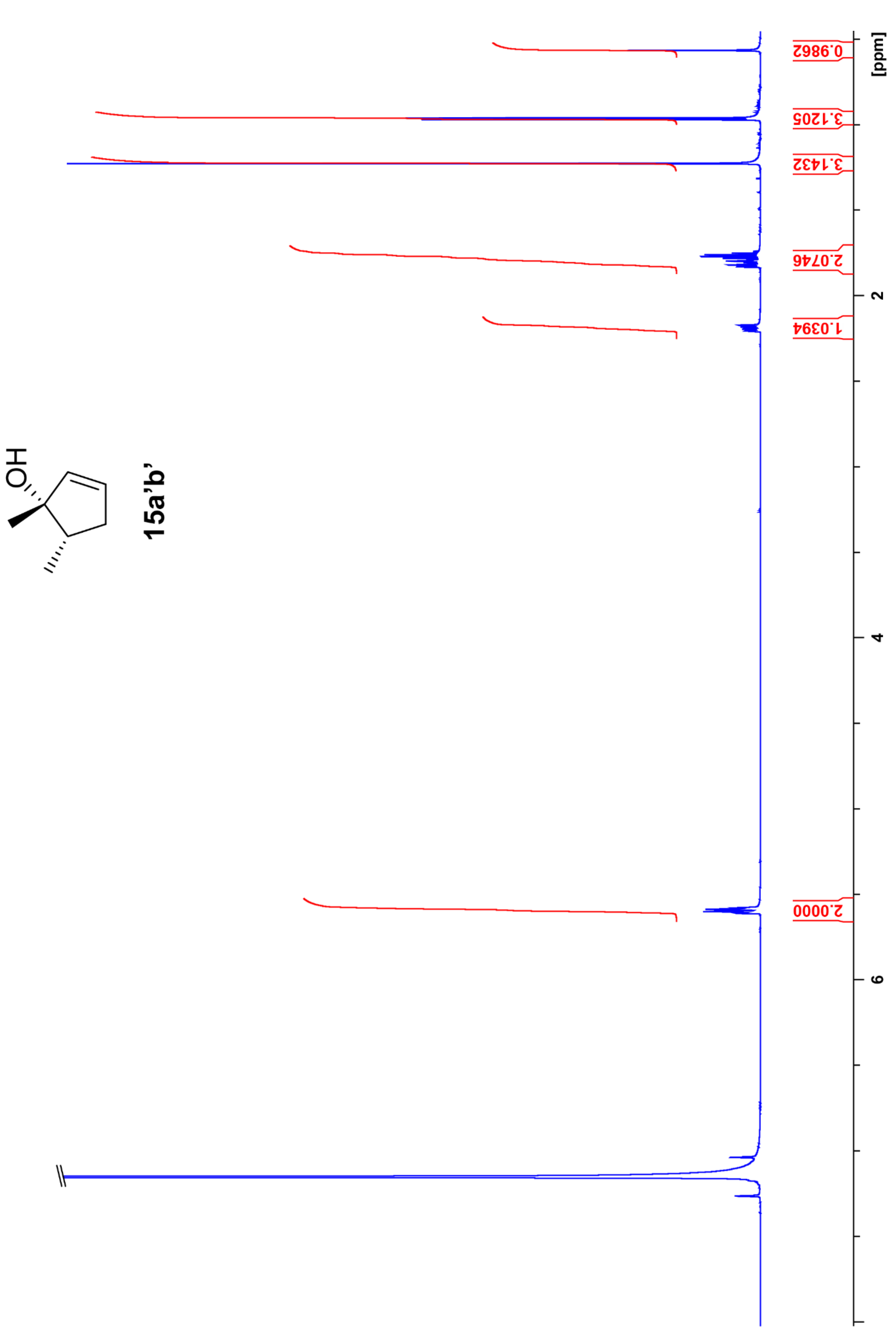

Figure S30. ^{1}H-NMR spectrum of **15a'b'** (700 MHz, C_6D_6).

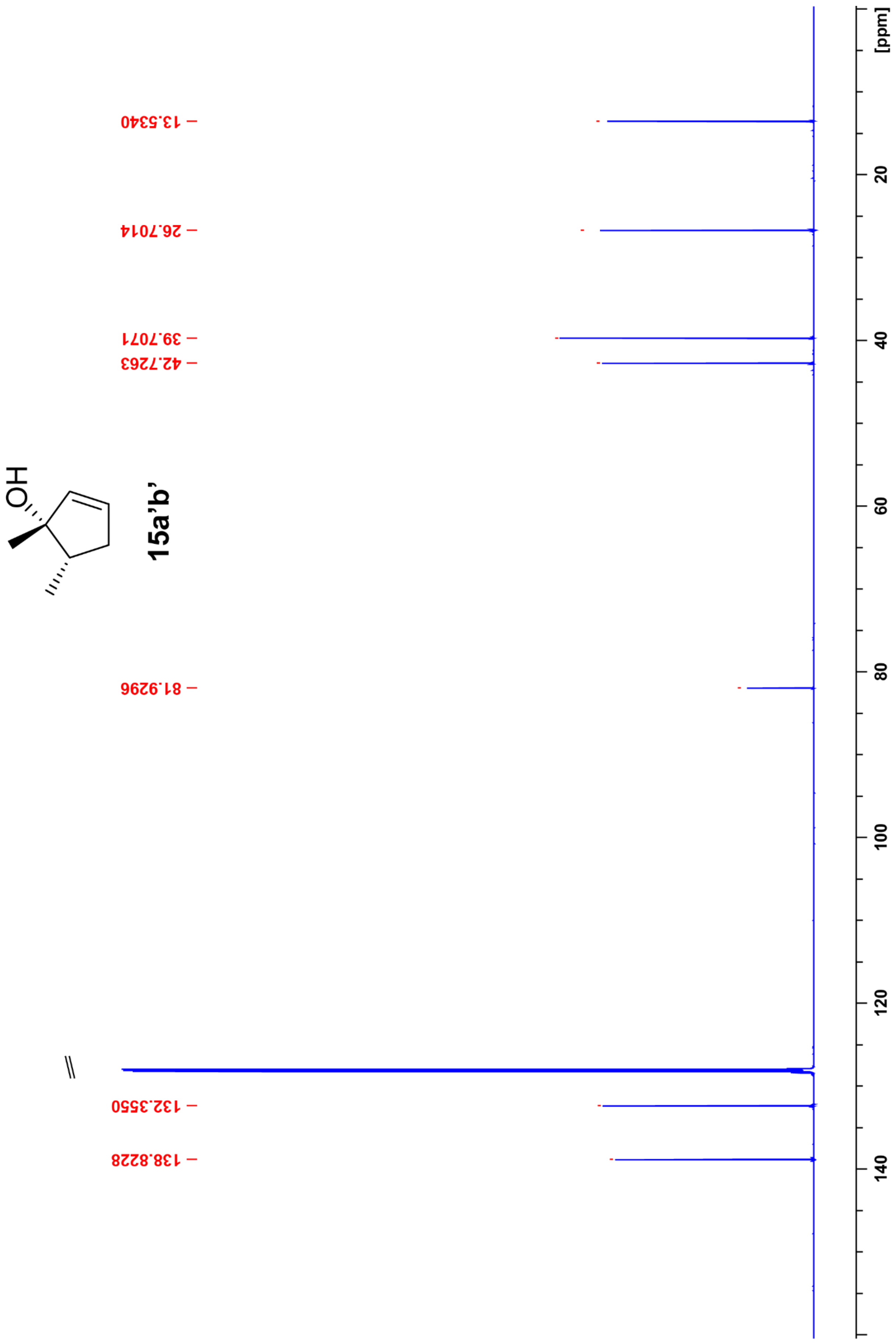

Figure S31. ^{13}C-NMR spectrum of **15a'b'** (175 MHz, C_6D_6).

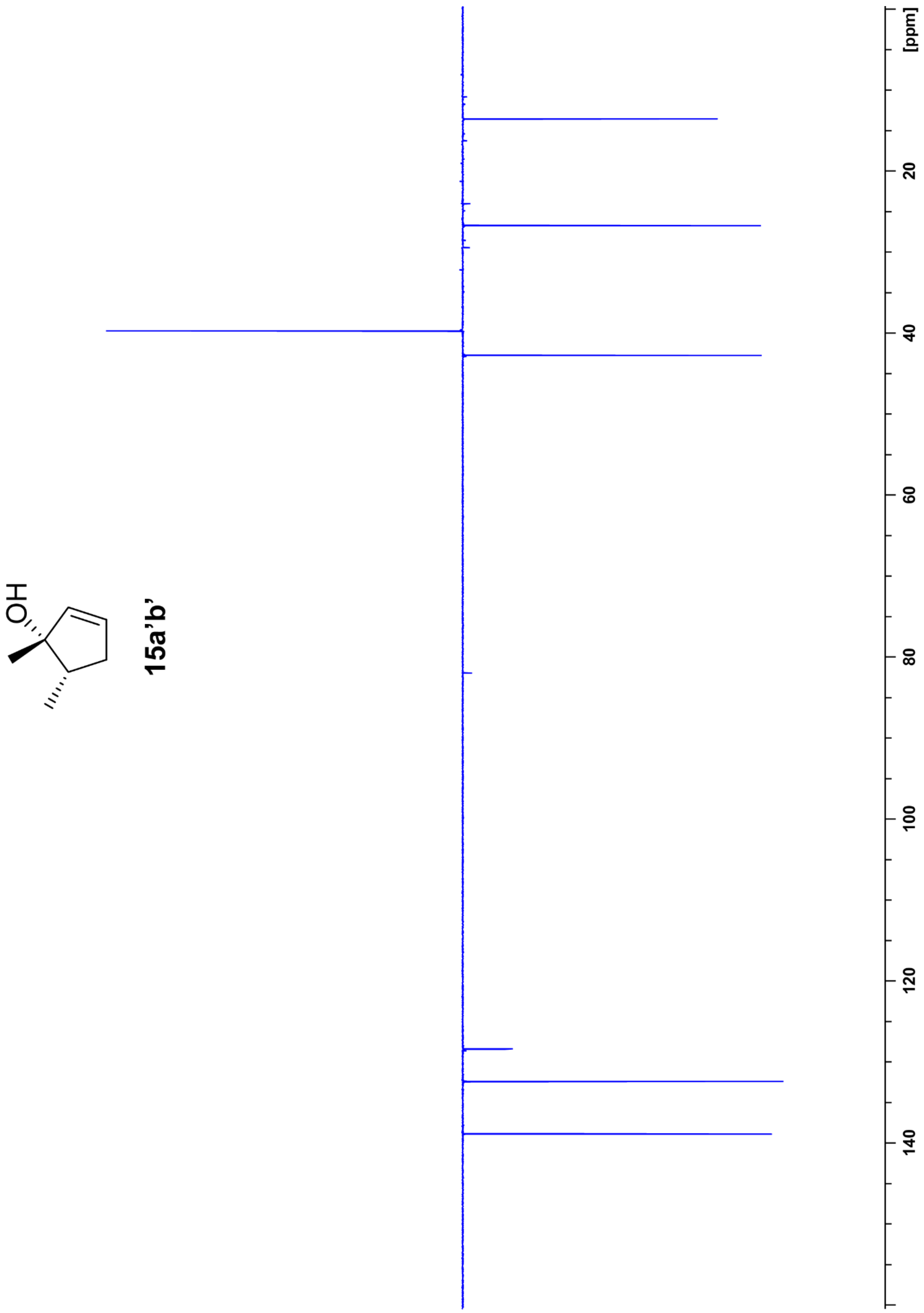

Figure S32. ^{13}C-DEPT spectrum of **15a'b'** (175 MHz, C_6D_6).

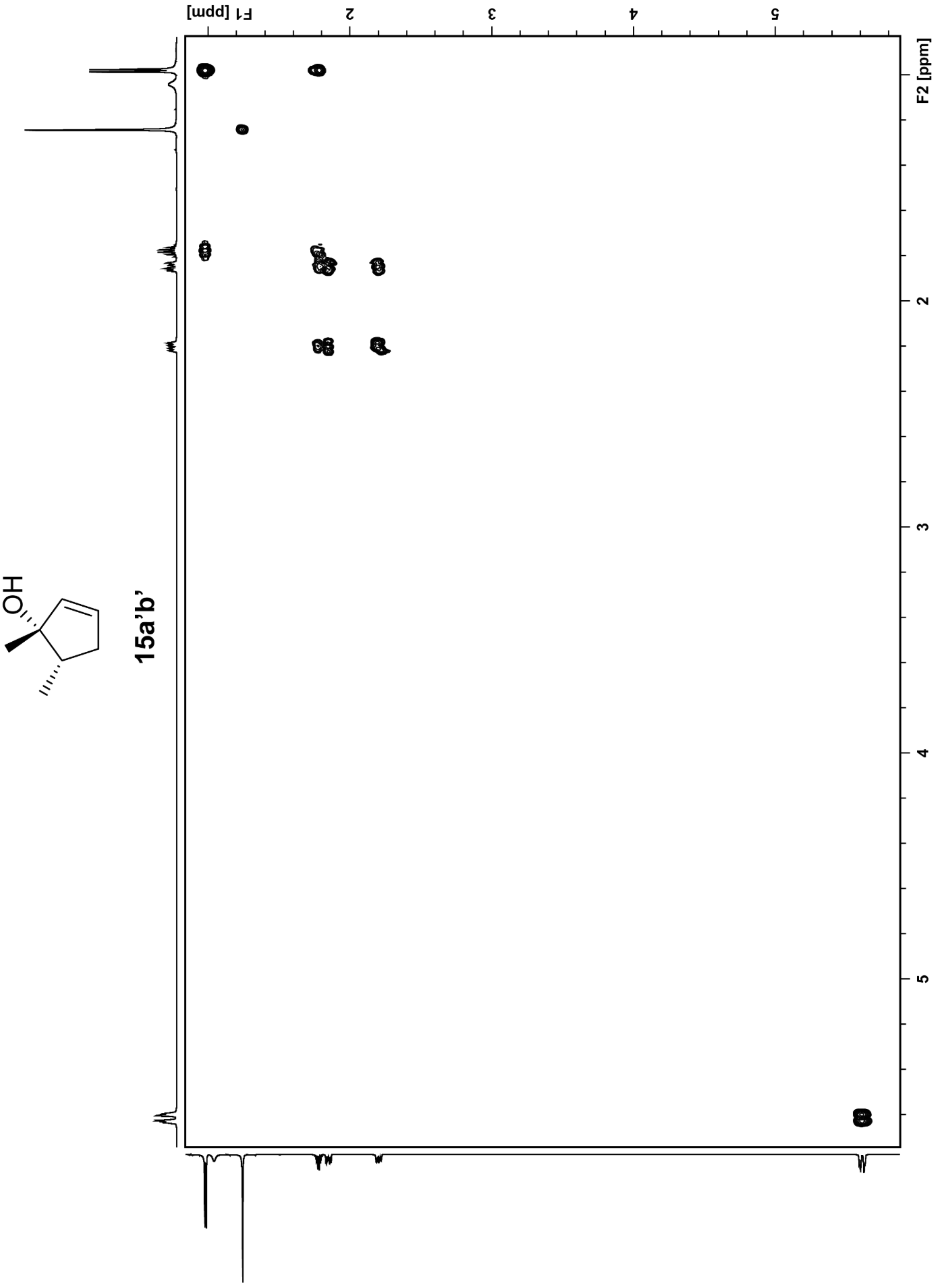

Figure S33. $^{1}H,^{1}H$-COSY spectrum of **15a'b'** (C_6D_6).

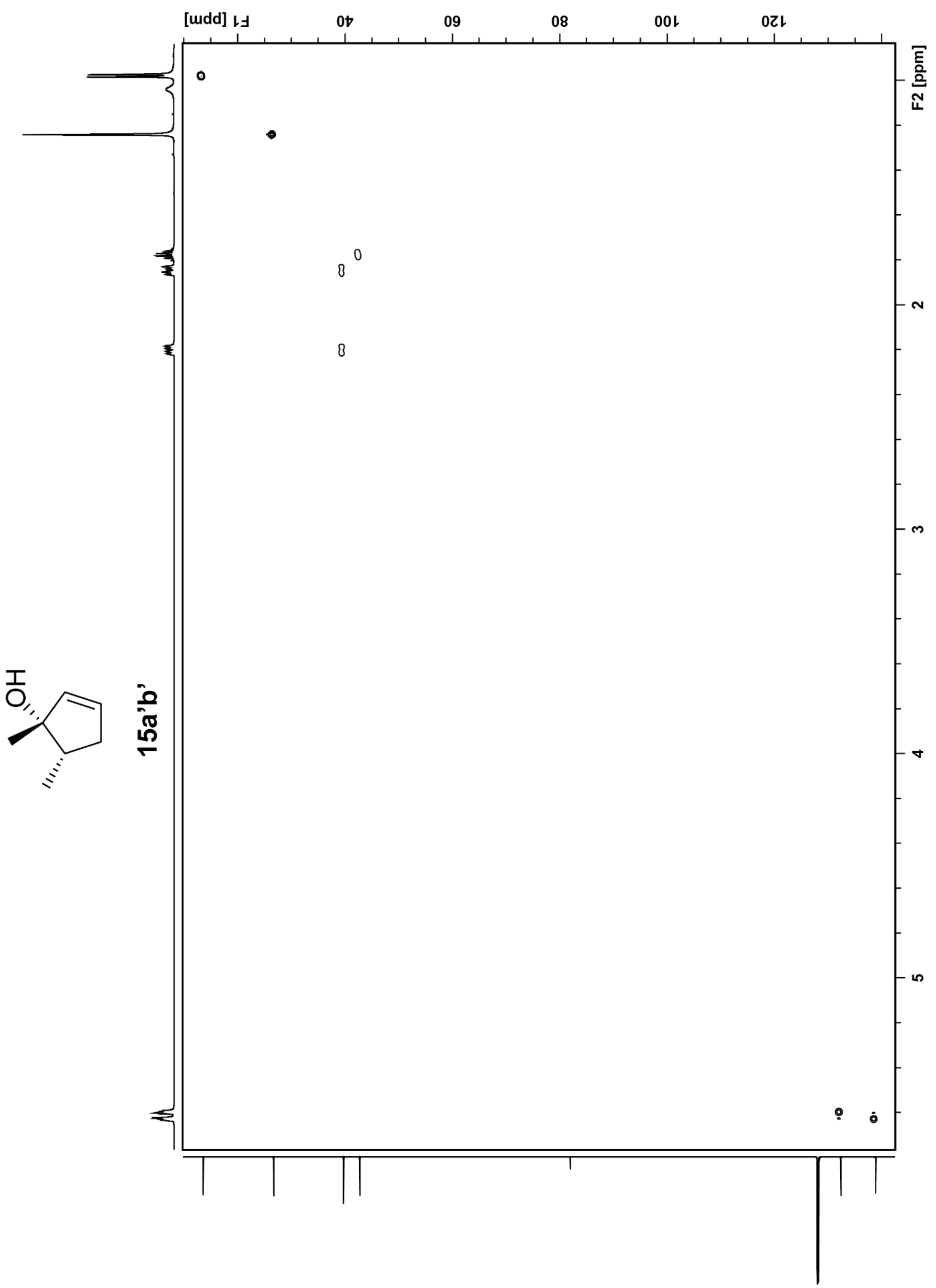

Figure S34. HSQC spectrum of **15a'b'** (C_6D_6).

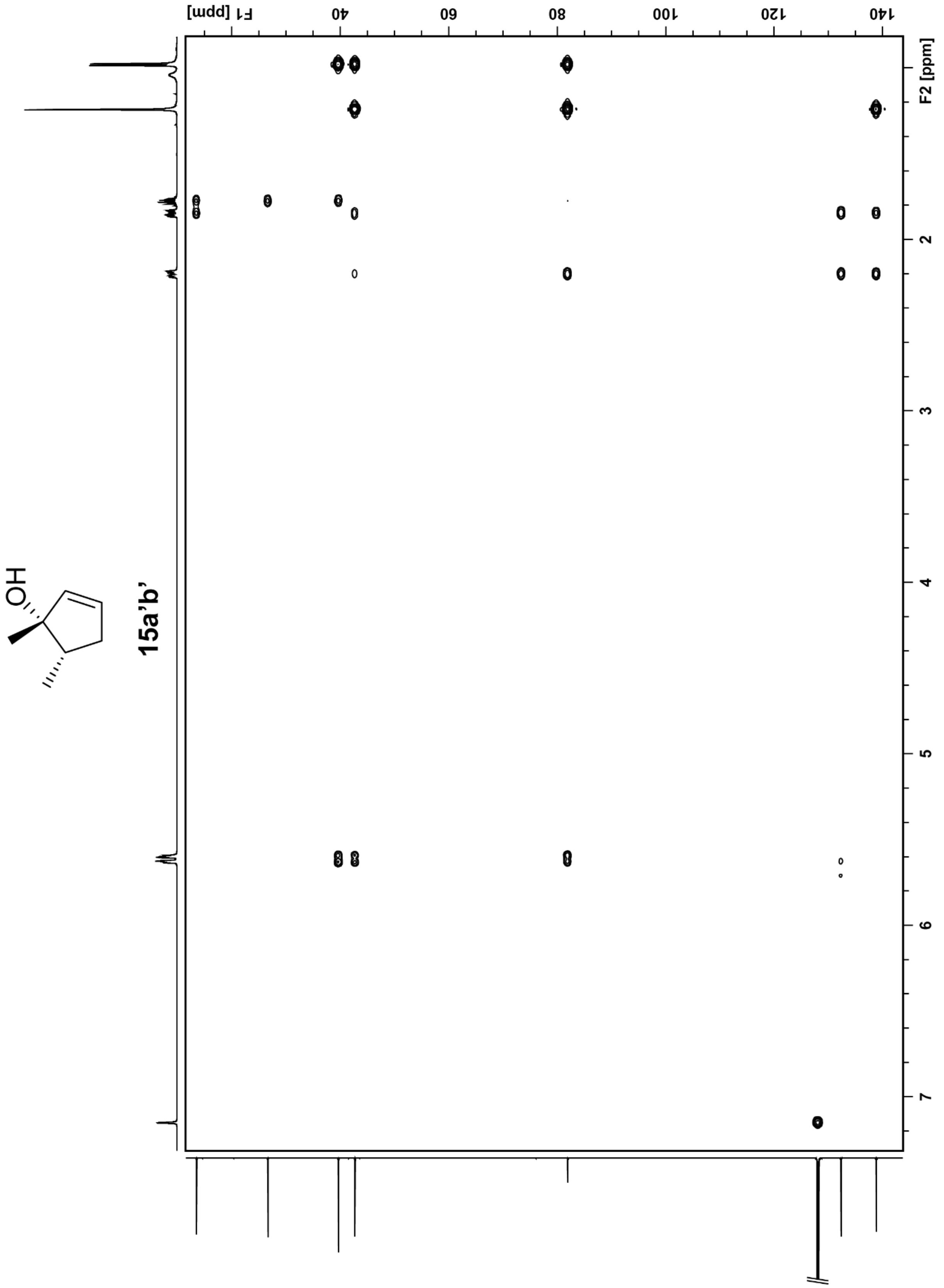

Figure S35. HMBC spectrum of **15a'b'** (C_6D_6).

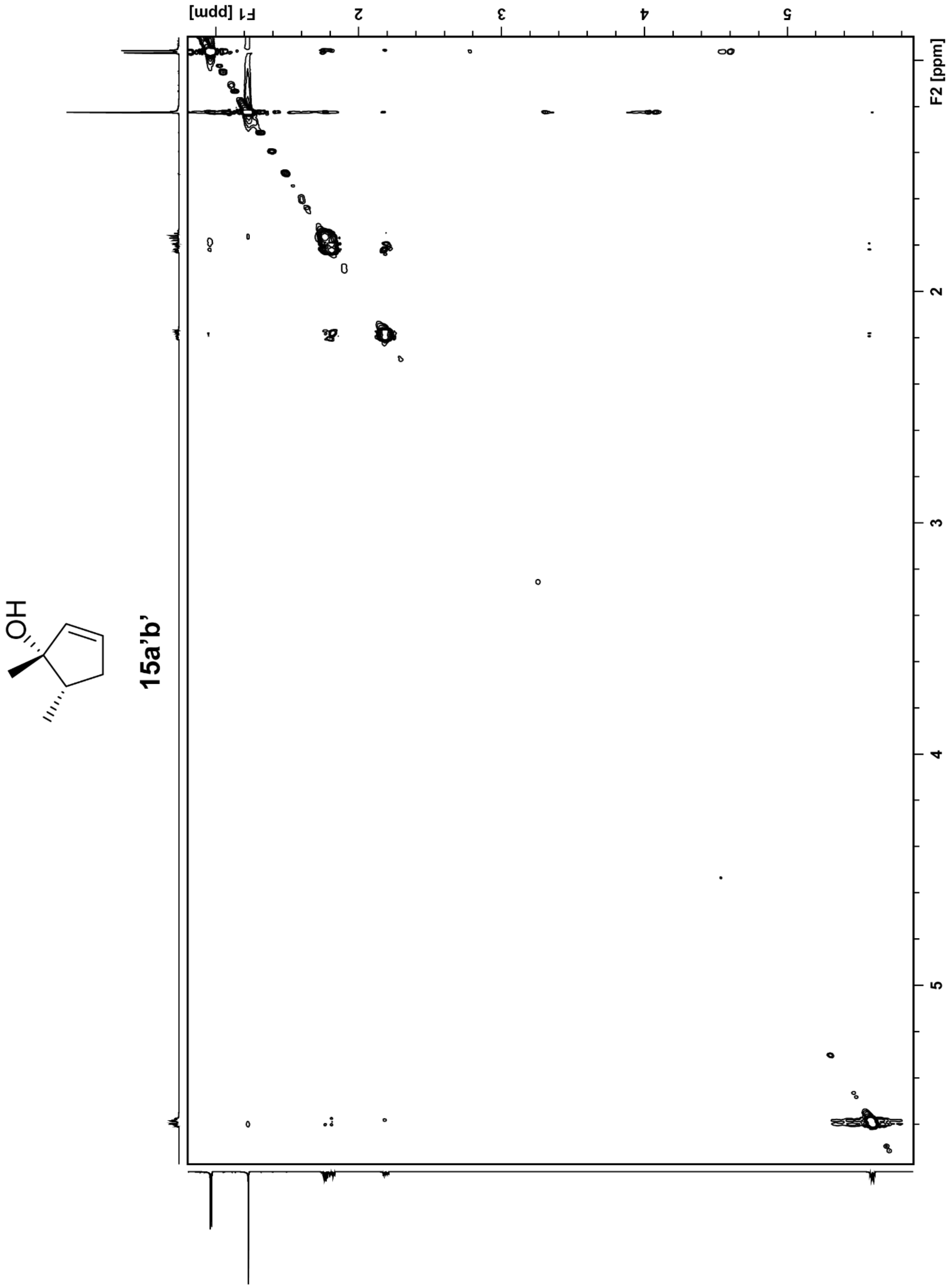

Figure S36. NOESY spectrum of **15a'b'** (C_6D_6).

HPLC conditions

HPLC anlysises were performed on Azura series HPLC system(Knauer, Berlin, Germany), equipped with a Photometric Diode Array detector PDA(190–1000 nm) and a Daicel (Tokyo, Japan) Chiralpak® IA column (5 μm; 4.6 × 250 mm). The elution was an isocratic solvent mixture of methanol/water (55/45) at 1.0 mL/min (124 bar). The UV–vis absorption was monitored at 205 nm.

Purification via HPLC were performed on an Azura series HPLC system with a multi wavelength detector MWL 2.1L (190–700 nm) and a Daicel Chiralpak® IA column (5 μm; 250 × 20 mm). The elution was an isocratic mixture of methanol/water (55/45) at 12 mL/min (158 bar). The UV–vis absorption was monitored at 205 nm.

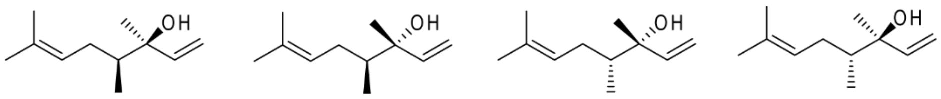

(3*S*, 4*S*)-**14a** (3*R*, 4*S*)-**14a**' (3*R*, 4*R*)-**14b** (3*S*, 4*R*)-**14b'**

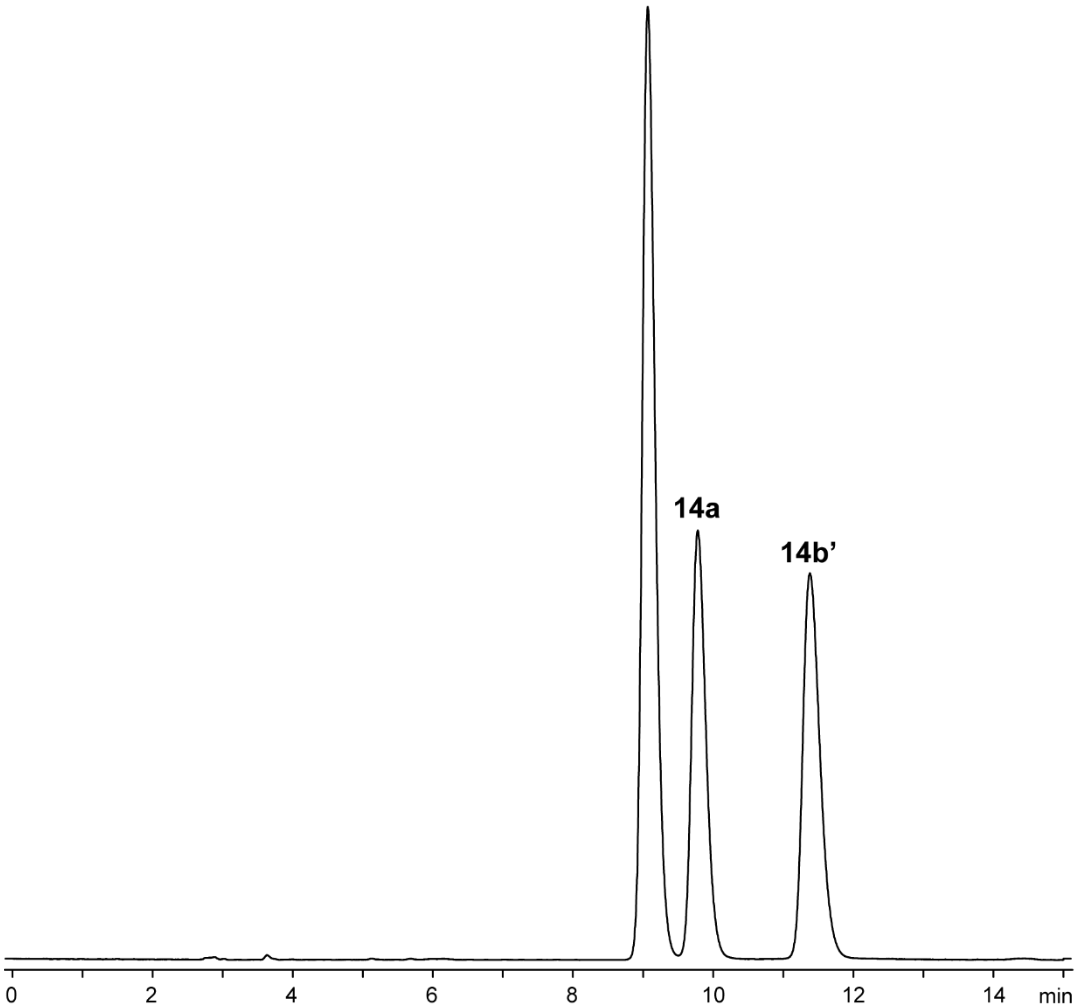

Figure S37. HPLC-UV chromatograms of **14** separated using a chiral stationary phase. Purified (3*S*,4*S*)-**14a**, also obtained as the minor product in the enzyme reaction with CpLS and FPPS from **8a**, purified (3*S*,4*R*)-**14b'**, also obtained as the major product in the enzyme reaction with CpLS and FPPS from **8b**. (3*R*,4*S*)-**14a'** and (3*R*,4*R*)-**14b** elute with same retention time.

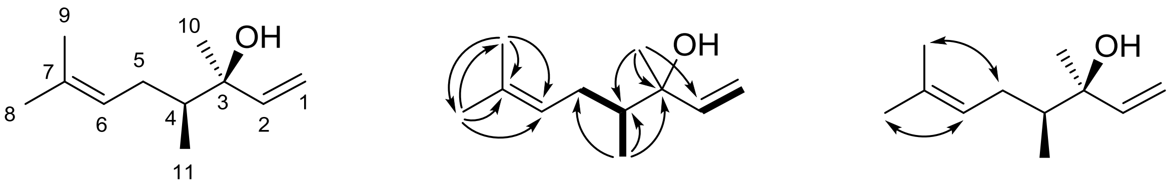

Table S5. NMR data of (3*S*,4*S*)-**14a** in C_6D_6 recorded at 298 K.

C[a]		^{13}C[b]	^{1}H[b]
1	CH_2	112.21	5.19 (dd, *J* = 17.3, 1.6, H_Z)
			4.98 (dd, *J* = 10.7, 1.6, H_E)
2	CH	144.22	5.79 (dd, *J* = 17.3, 10.7)
3	C_q	75.37	–
4	CH	44.40	1.49 (ddq, *J* = 10.5, 3.6, 6.9)
5	CH_2	30.35	2.35 (m)
			1.74 (ddd, *J* = 14.2, 9.2, 9.2)
6	CH	124.72	5.20 (m)
7	C_q	131.96	–
8	CH_3	25.97	1.66 (d, *J* = 0.8)
9	CH_3	17.93	1.55 (br s)
10	CH_3	25.91	1.10 (s)
11	CH_3	14.74	0.87 (d, *J* = 6.9)
–	OH	–	0.98 (br s)

[a] Carbon numbering as shown in the structure above the table (bold lines: ^{1}H,^{1}H-COSY correlations, single headed arrows: HMBC correlations, double headed arrows: NOESY correlations). [b] Chemical shifts δ in ppm, multiplicity: s = singlet, d = doublet, q = quartet, br = broad, coupling constants *J* are given in Hertz.

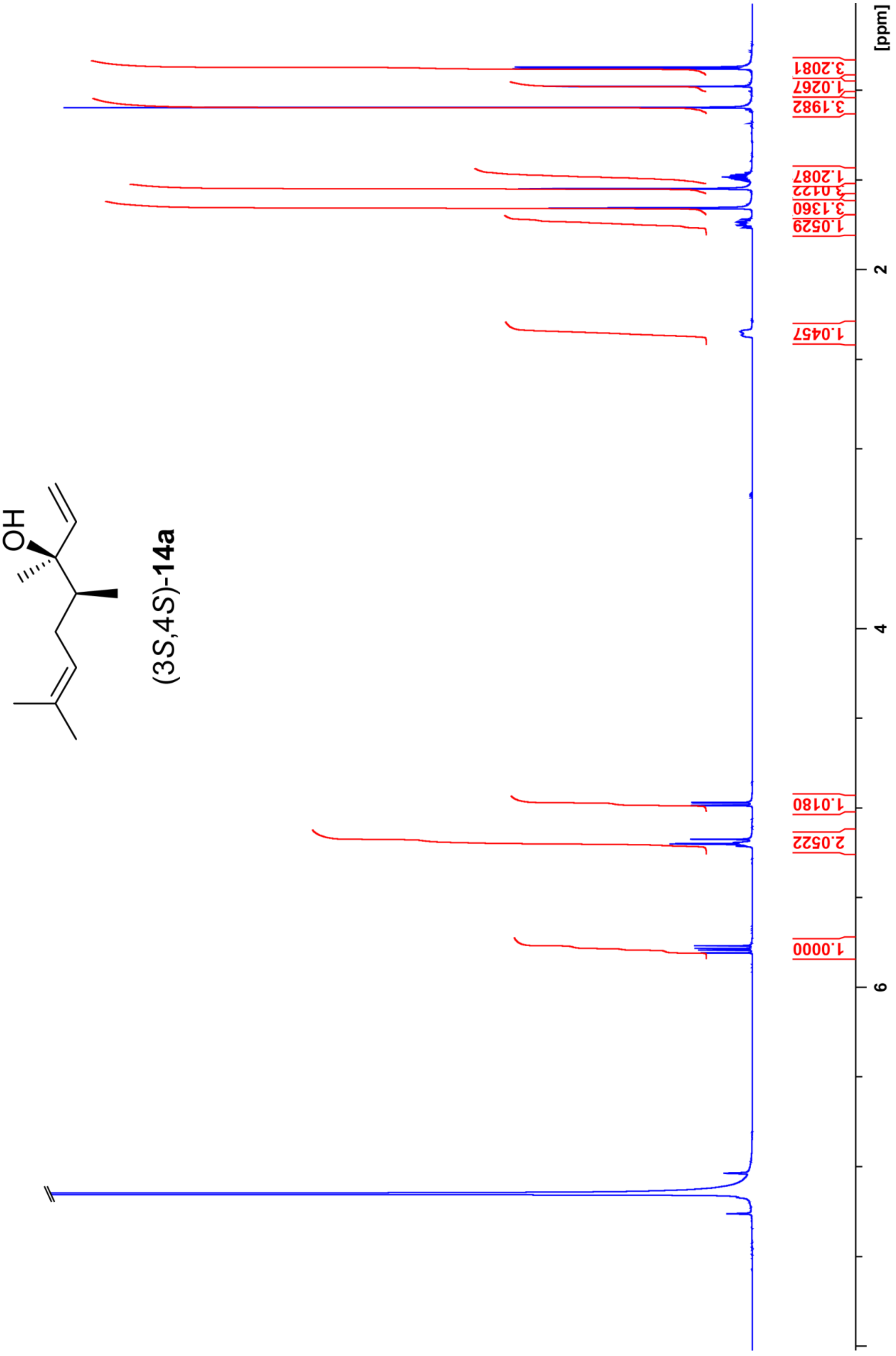

Figure S38. ^{1}H-NMR spectrum of (3*S*,4*S*)-**14a** (700 MHz, C_6D_6).

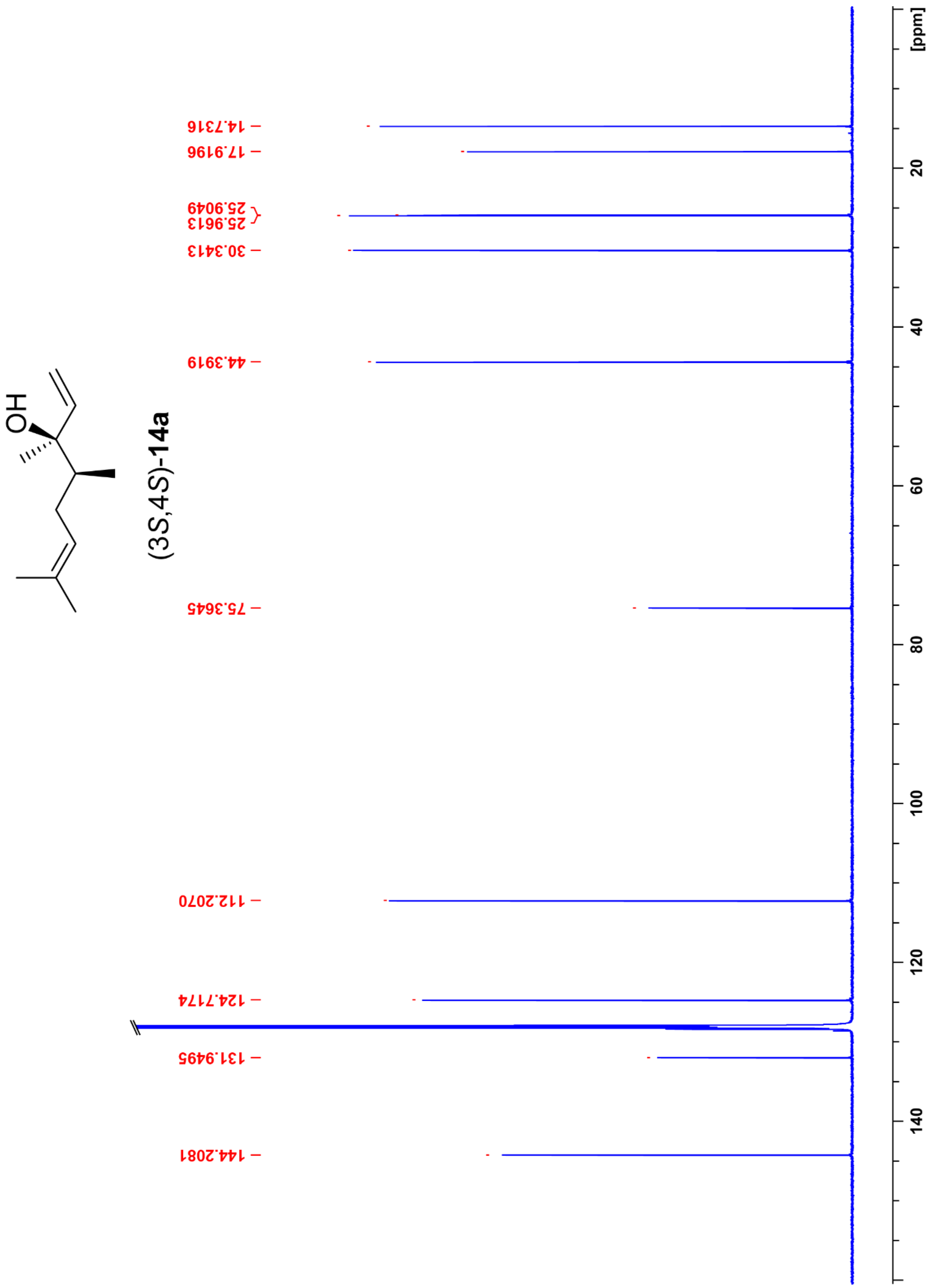

Figure S39. ^{13}C-NMR spectrum of (3*S*,4*S*)-**14a** (175 MHz, C_6D_6).

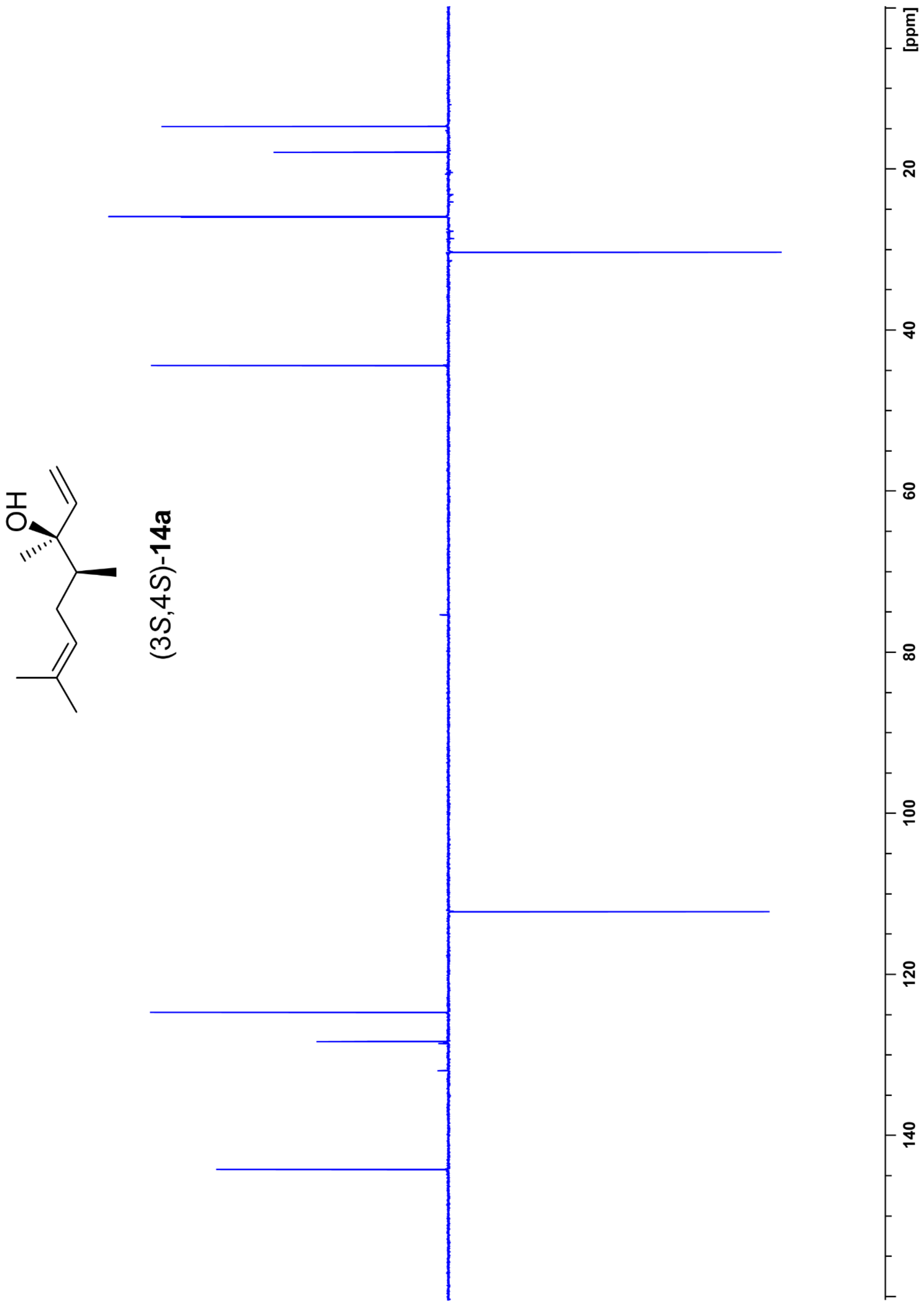

Figure S40. ^{13}C-DEPT spectrum of (3*S*,4*S*)-**14a** (175 MHz, C_6D_6).

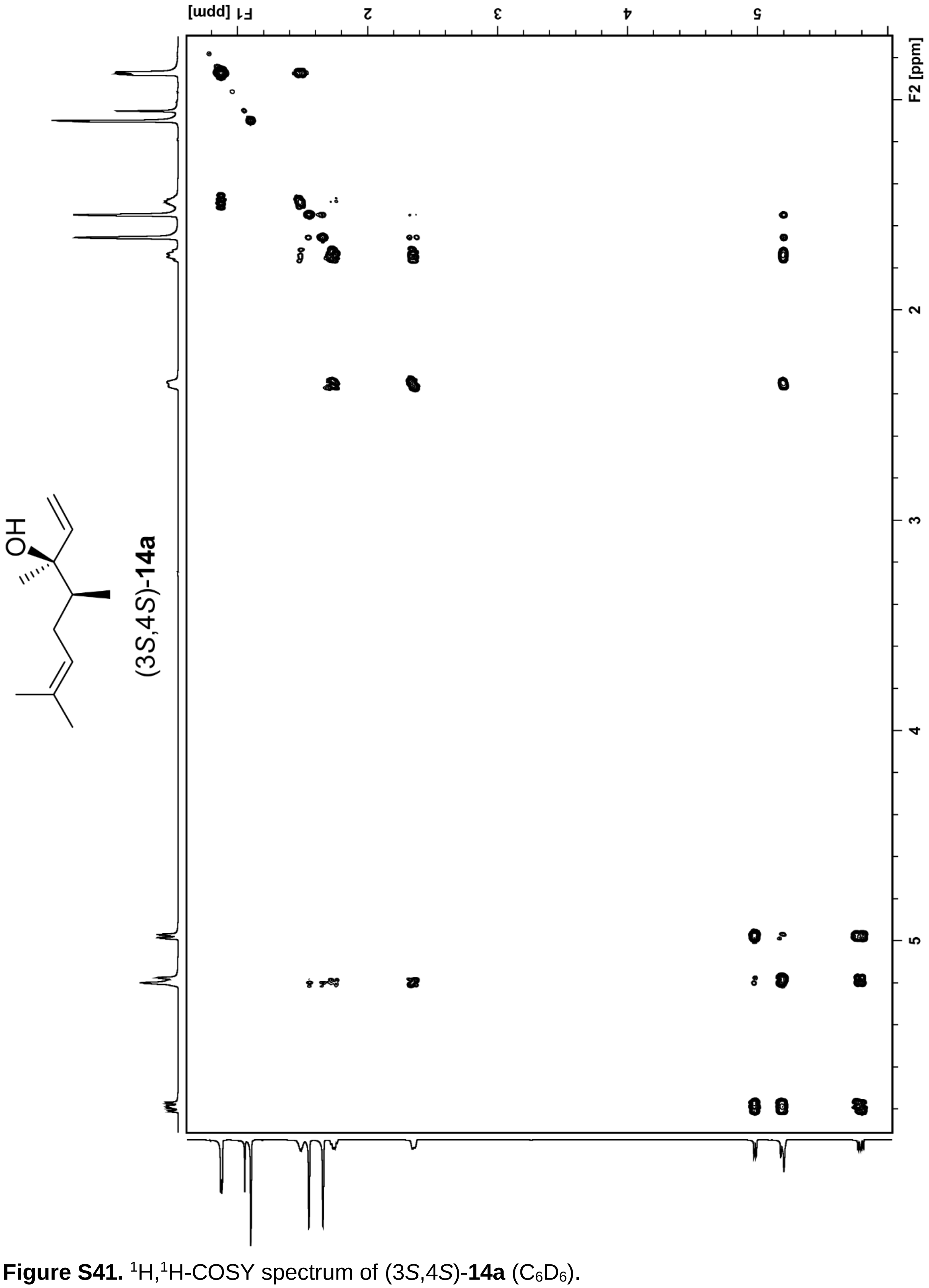

Figure S41. $^{1}H,^{1}H$-COSY spectrum of (3*S*,4*S*)-**14a** (C_6D_6).

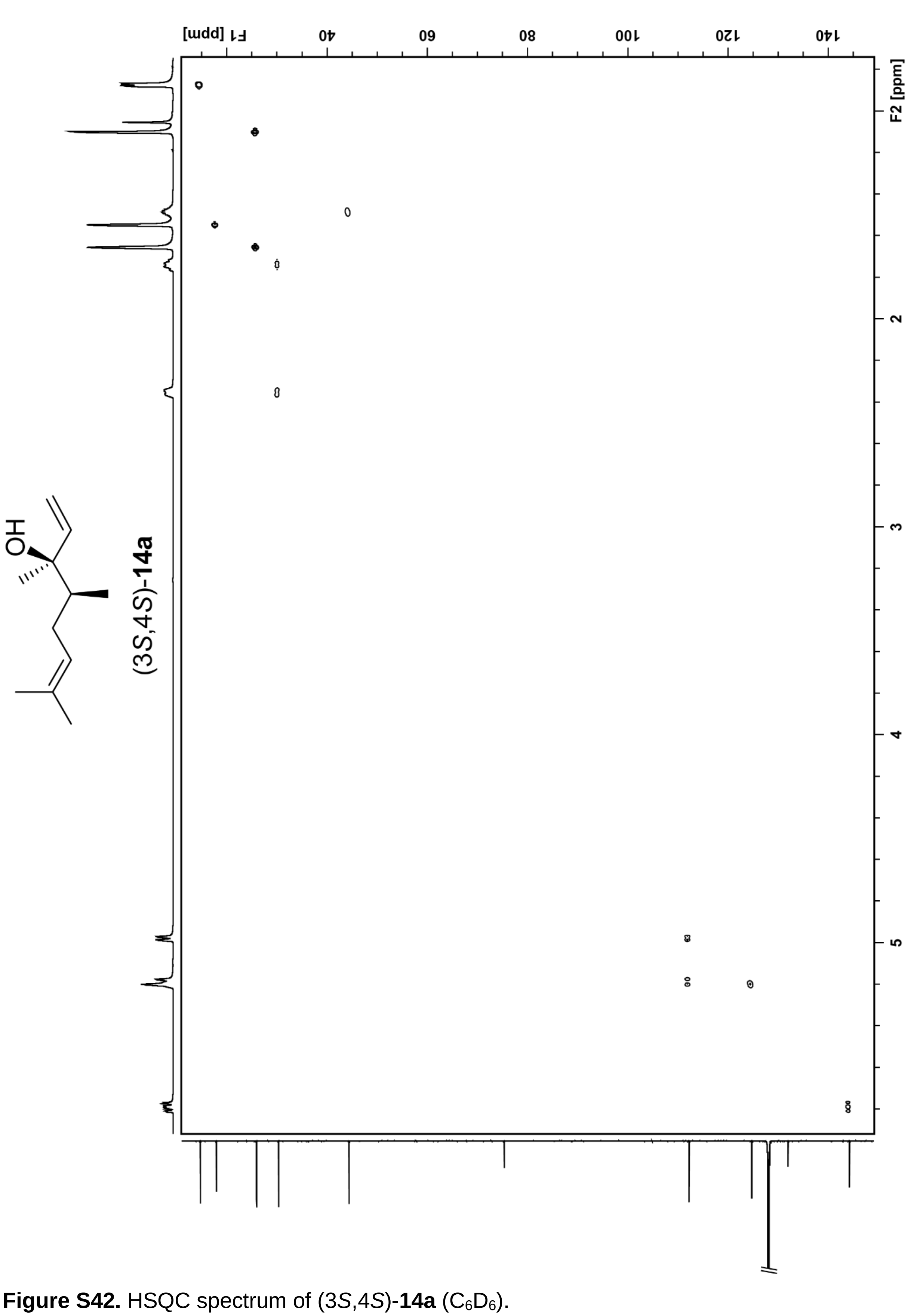

Figure S42. HSQC spectrum of (3*S*,4*S*)-**14a** (C_6D_6).

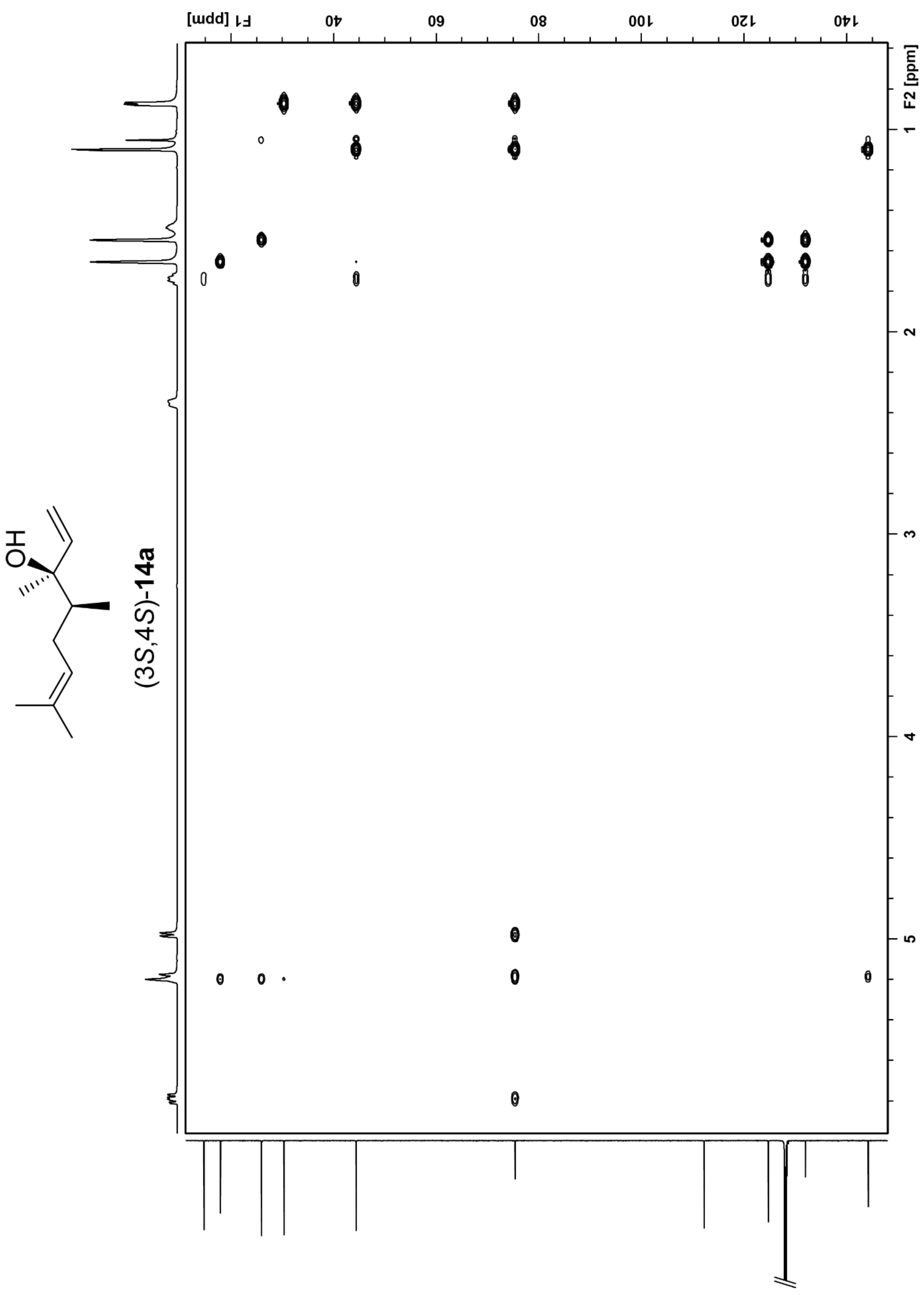

Figure S43. HMBC spectrum of (3*S*,4*S*)-**14a** (C_6D_6).

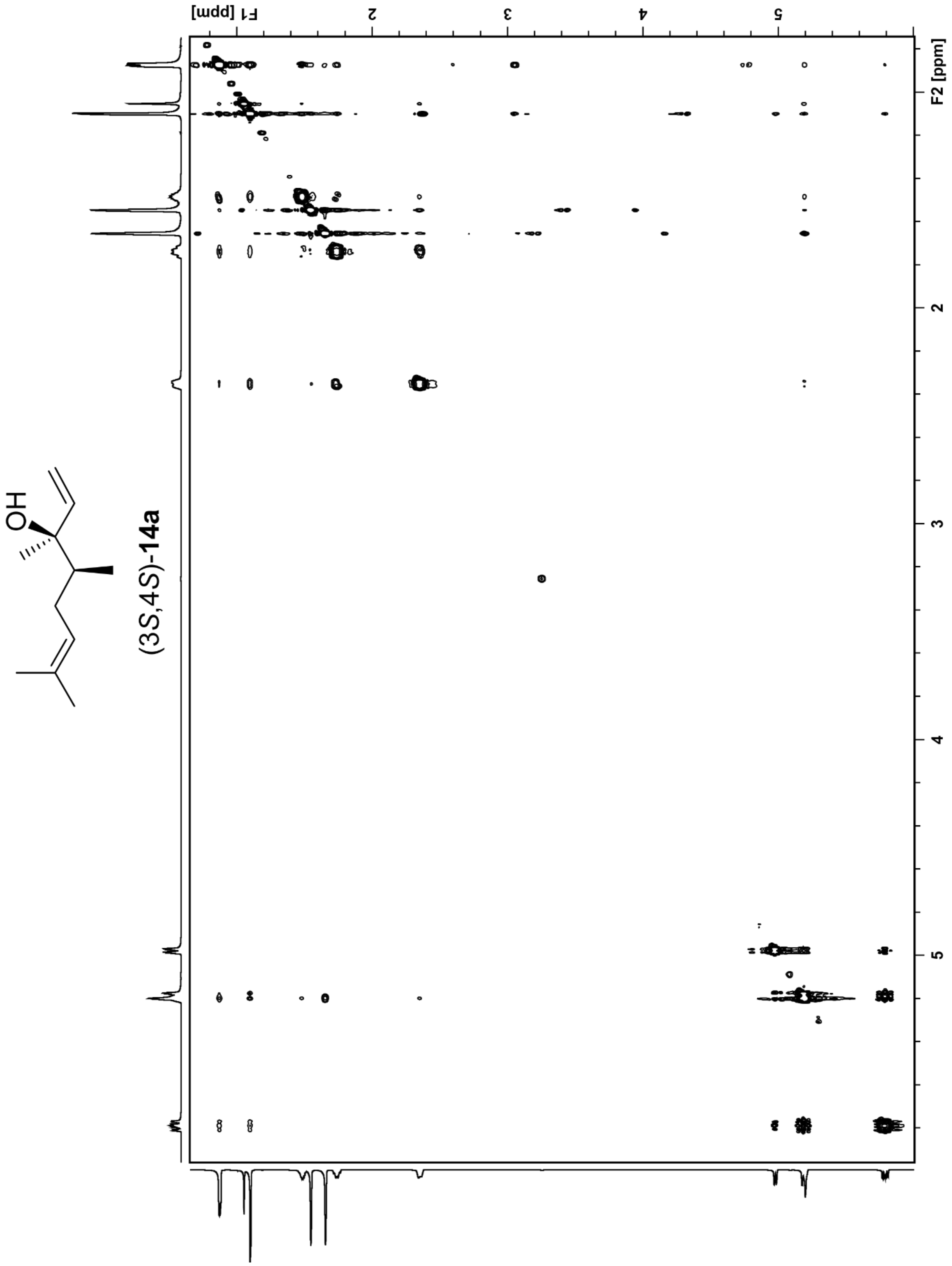

Figure S44. NOESY spectrum of (3*S*,4*S*)-**14a** (C_6D_6).

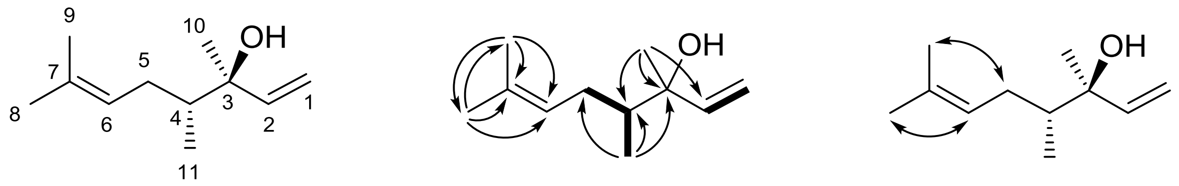

Table S6. NMR data of (3*S*,4*R*)-**14b'** in C_6D_6 recorded at 298 K.

C[a]		^{13}C[b]	^{1}H[b]
1	CH_2	111.89	5.97 (dd, *J* = 10.7, 1.7, H_E)
			5.185 (dd, *J* = 17.3, 1.7, H_Z)
2	CH	145.30	5.77 (dd, *J* = 17.3, 10.7)
3	C_q	75.33	–
4	CH	44.04	1.44 (ddq, *J* = 10.0, 3.1, 6.8)
5	CH_2	30.46	2.31 (m)
			1.78 (ddd, *J* = 14.0, 9.5, 9.5)
6	CH	124.61	5.190 (m)
7	C_q	131.95	–
8	CH_3	25.98	1.66 (br s)
9	CH_3	17.92	1.55 (br s)
10	CH_3	25.29	1.08 (s)
11	CH_3	13.99	0.91 (d, *J* = 6.9)
–	OH	–	0.89 (br s)

[a] Carbon numbering as shown in the structure above the table (bold lines: $^{1}H,^{1}H$-COSY correlations, single headed arrows: HMBC correlations, double headed arrows: NOESY correlations). [b] Chemical shifts δ in ppm, multiplicity: s = singlet, d = doublet, q = quartet, br = broad, coupling constants *J* are given in Hertz.

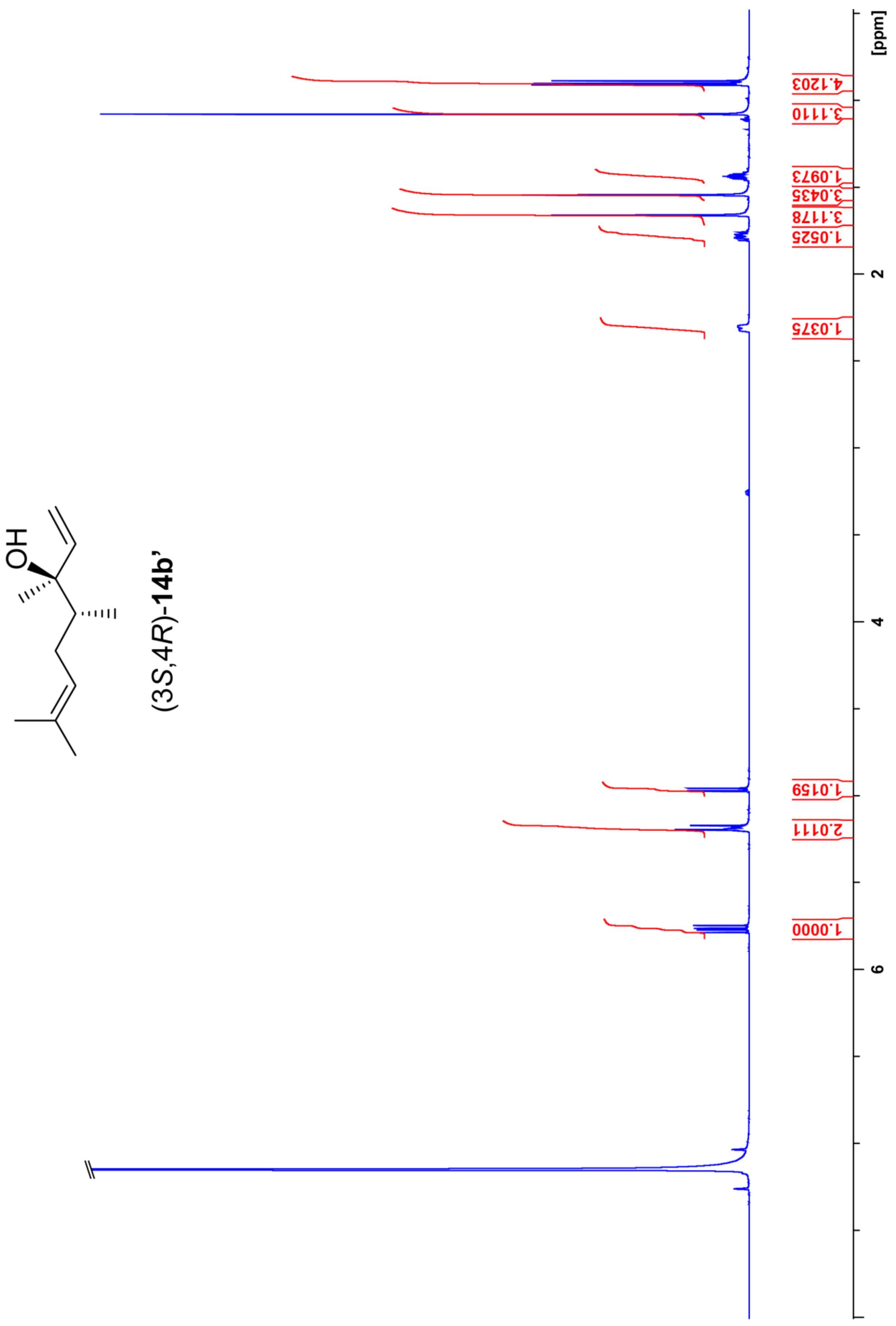

Figure S45. ^{1}H-NMR spectrum of (3*S*,4*R*)-**14b'** (700 MHz, C_6D_6).

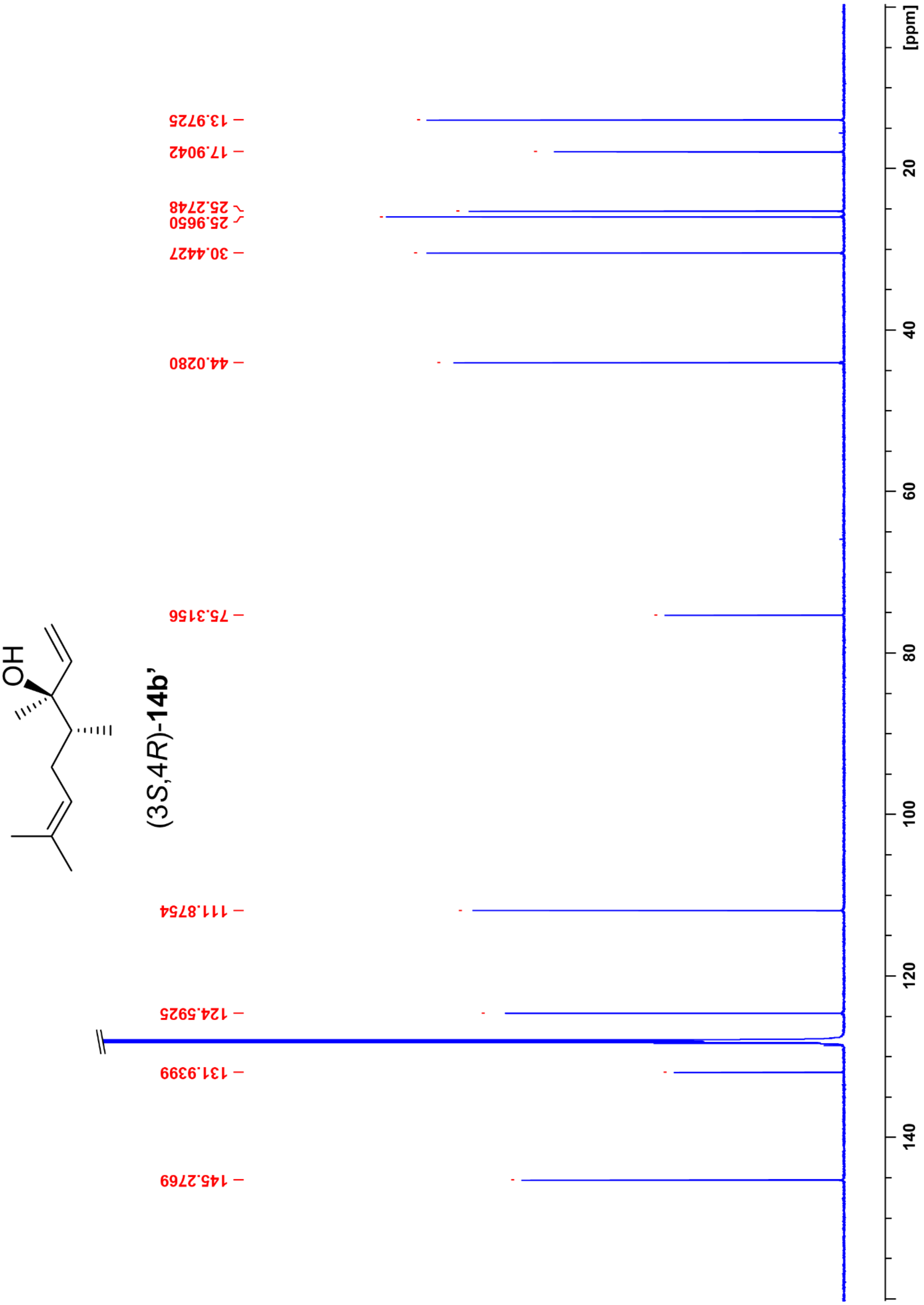

Figure S46. ^{13}C-NMR spectrum of (3*S*,4*R*)-**14b'** (175 MHz, C_6D_6).

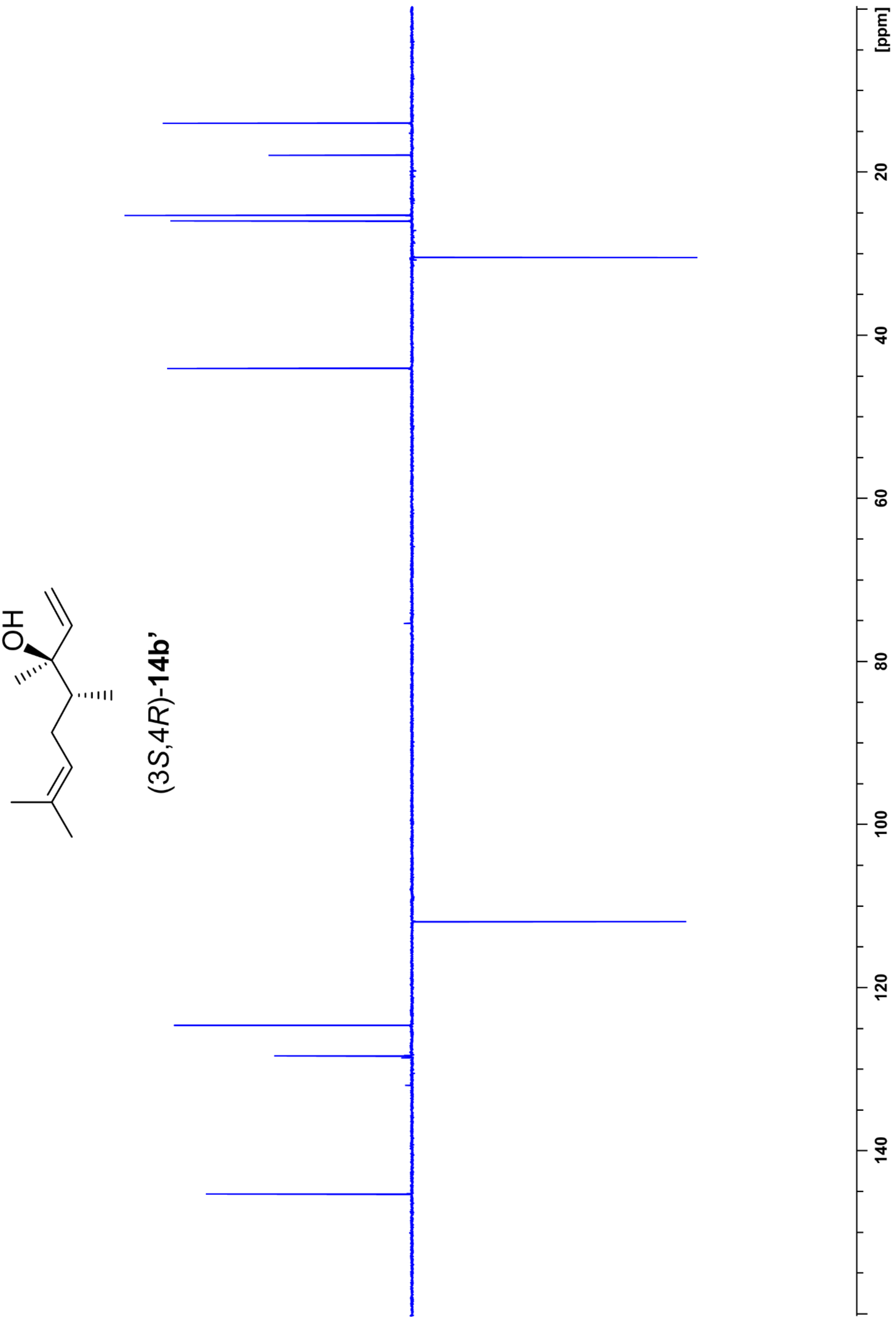

Figure S47. ^{13}C-DEPT spectrum of (3*S*,4*R*)-**14b’** (175 MHz, C_6D_6).

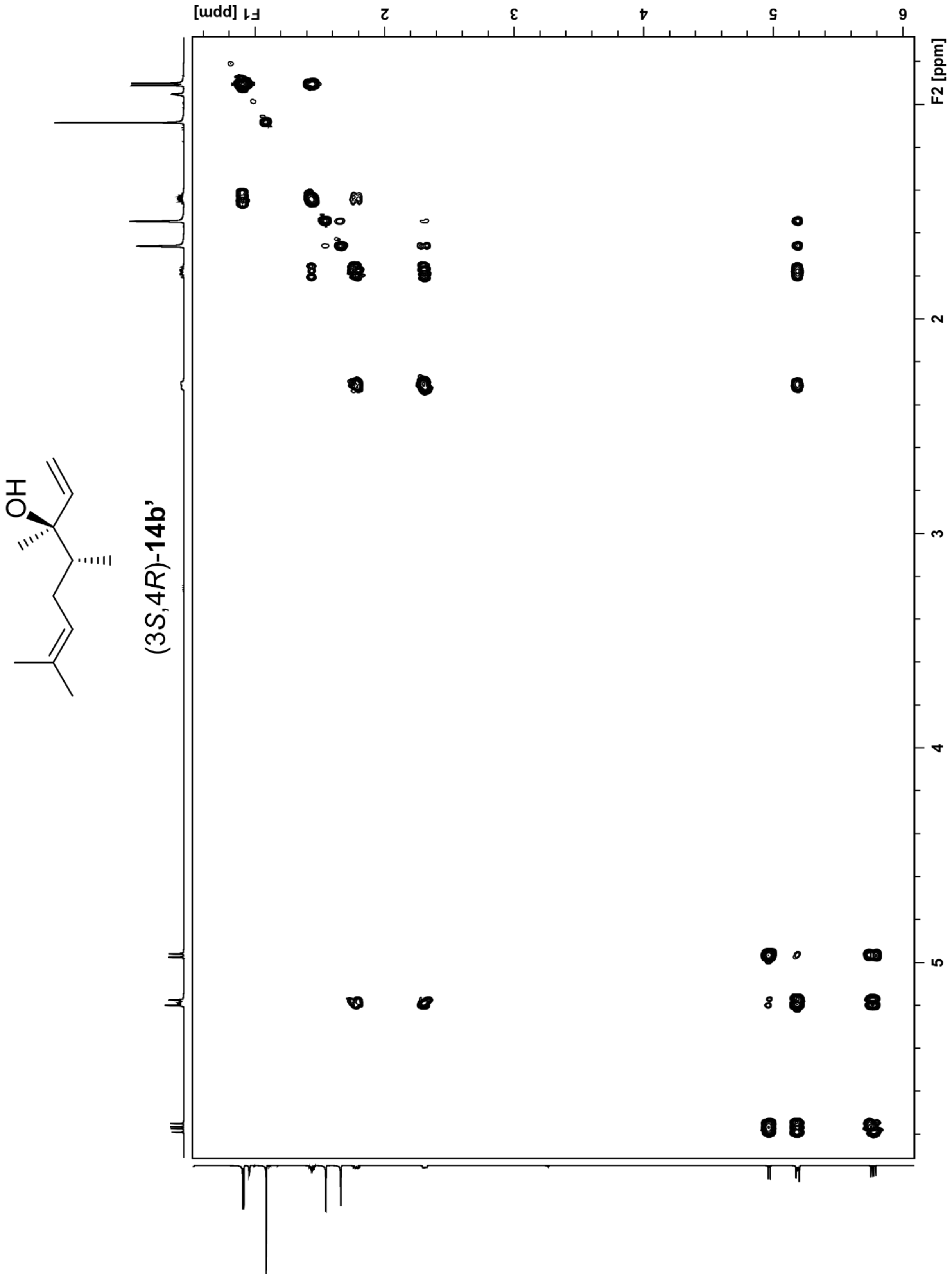

Figure S48. $^{1}H,^{1}H$-COSY spectrum of (3*S*,4*R*)-**14b'** (C_6D_6).

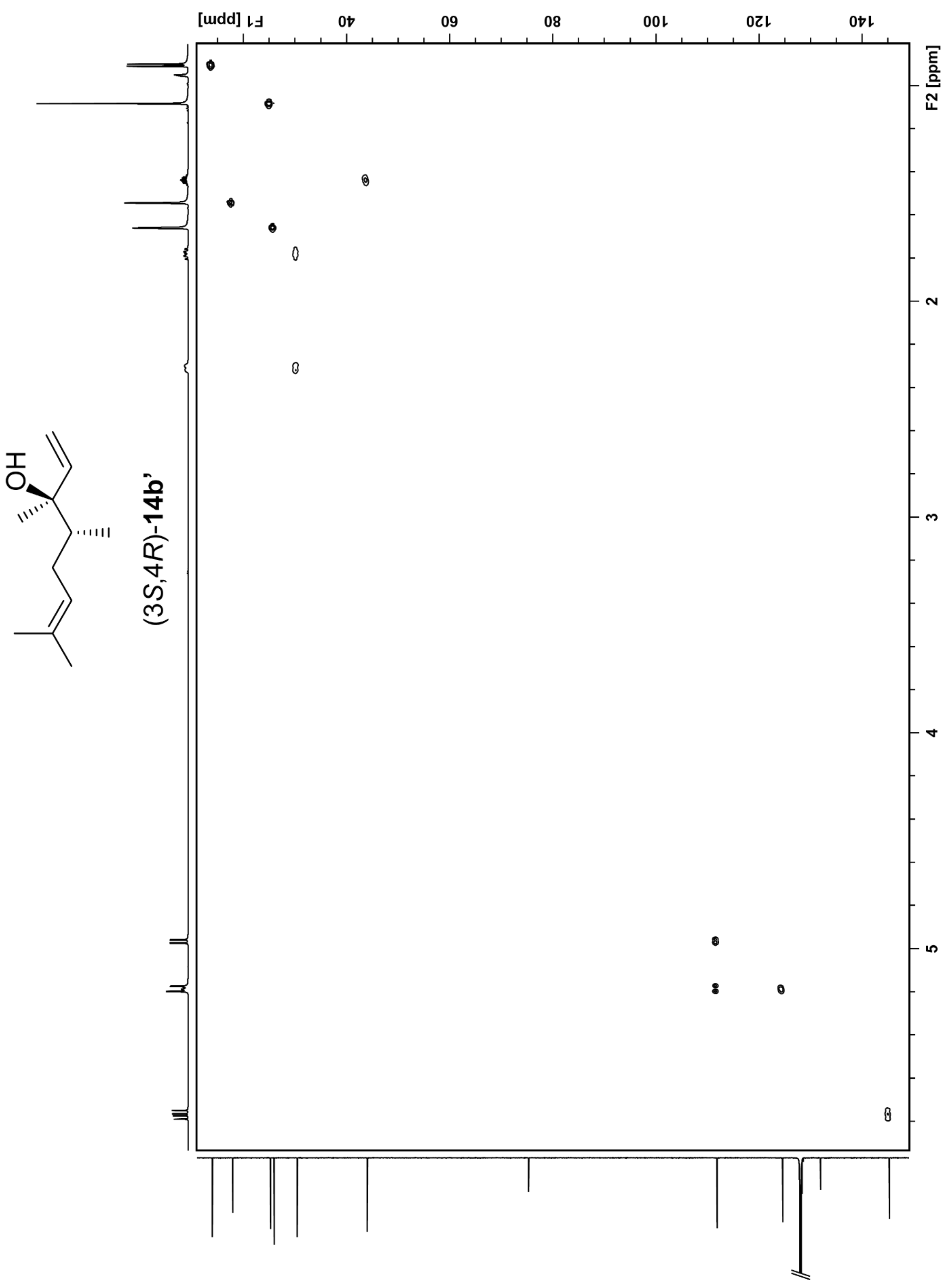

Figure S49. HSQC spectrum of (3*S*,4*R*)-**14b'** (C_6D_6).

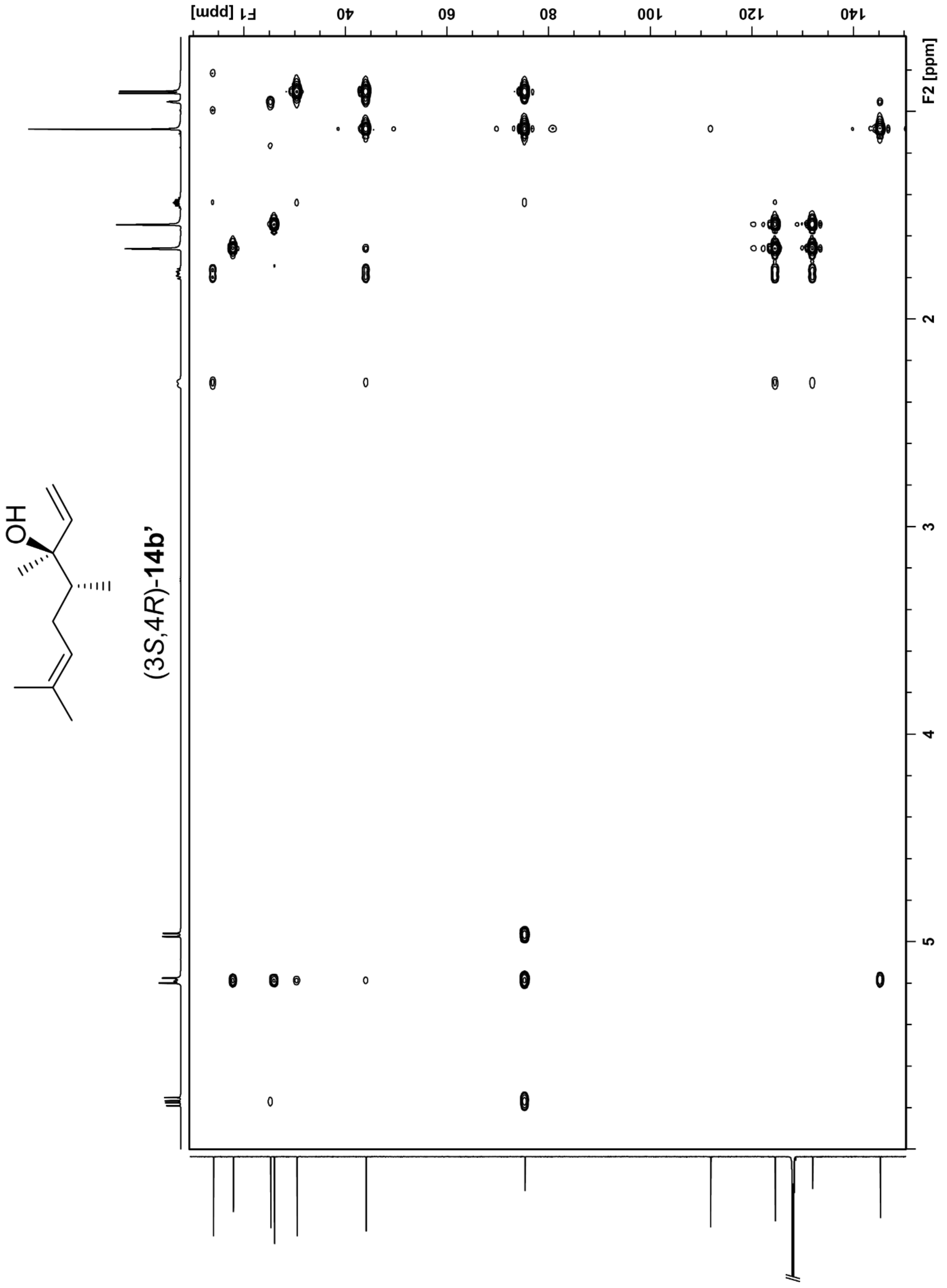

Figure S50. HMBC spectrum of (3*S*,4*R*)-**14b'** (C_6D_6).

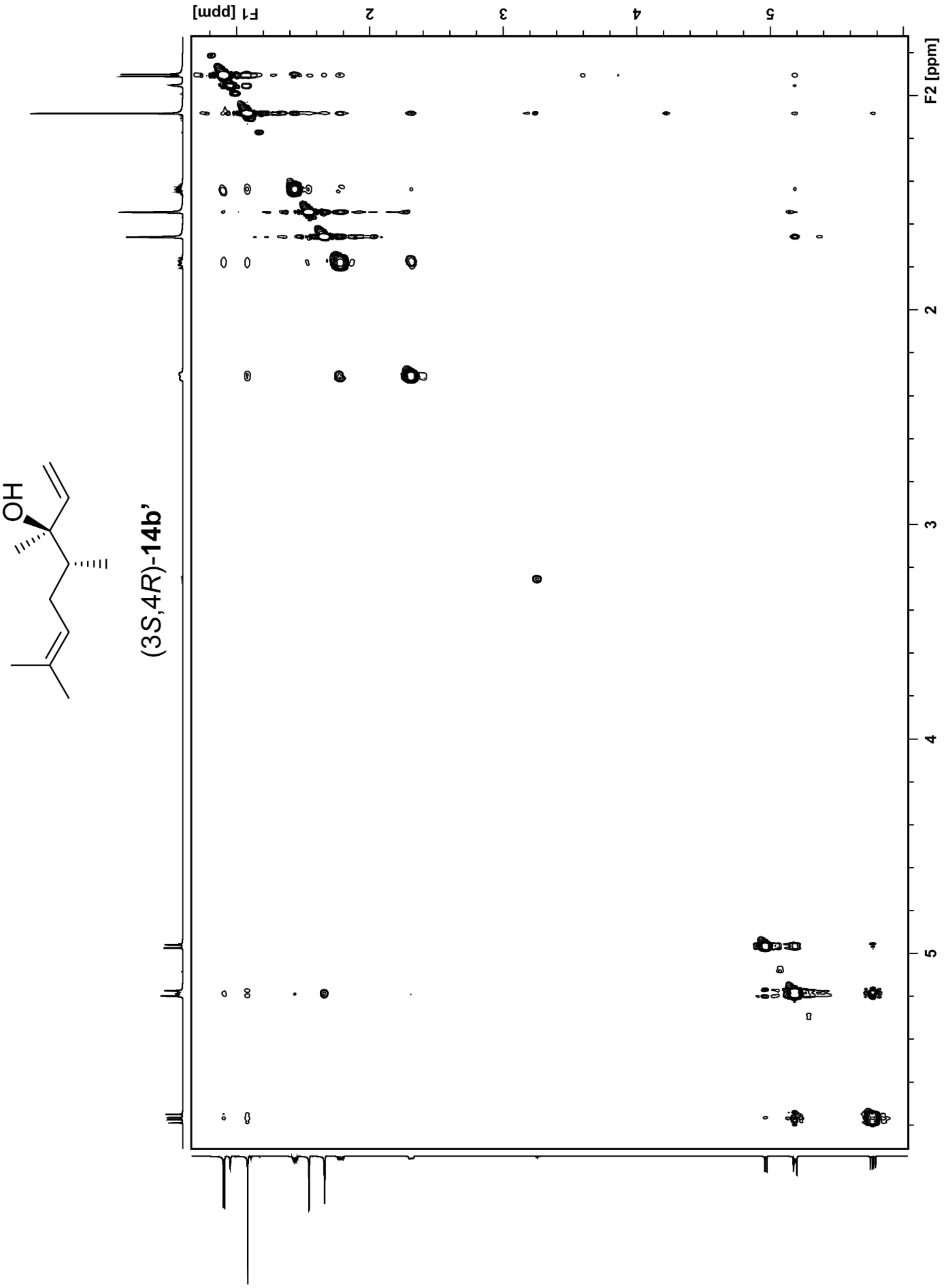

Figure S51. NOESY spectrum of (3*S*,4*R*)-**14b'** (C_6D_6).

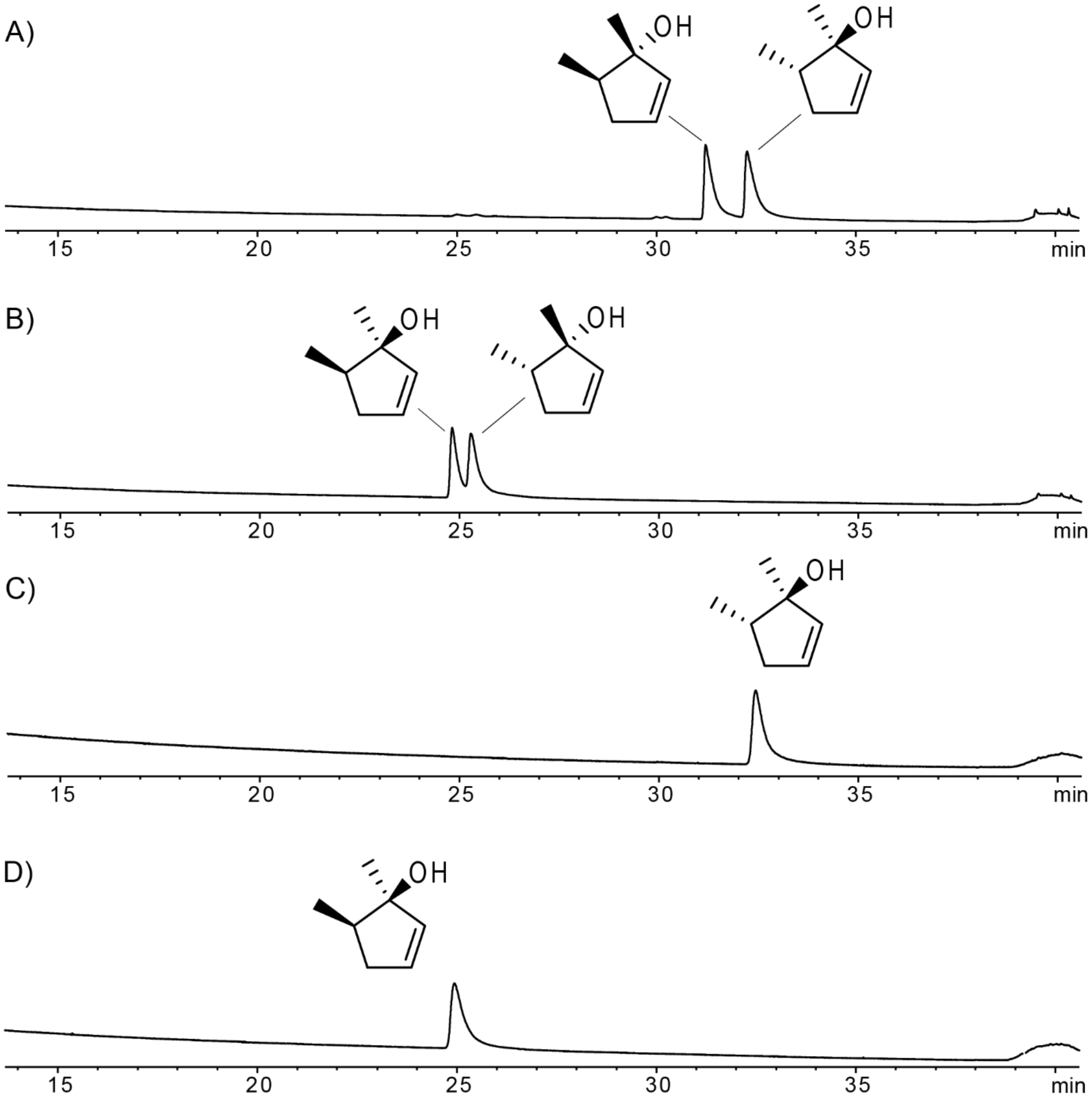

Figure S52. Total ion chromatograms of **15** separated by GC on a chiral Cyclosil-B stationary phase. A) Racemic (1*R**,5*S**)-**15ab**, obtained from the synthetic mixture of all four stereoisomers of **14** by cyclisation with Grubbs II catalyst and separation of the diastereomers by column chromatography, B) racemic (1*S**,5*S**)-**15a'b'** obtained in the same way, C) (1*R*,5*S*)-**15** obtained from HPLC purified (3*S*,4*S*)-**14** through cyclisation with Grubbs II catalyst, and D) (1*R*,5*R*)-**15** obtained from HPLC purified (3*S*,4*R*)-**14** in the same way.

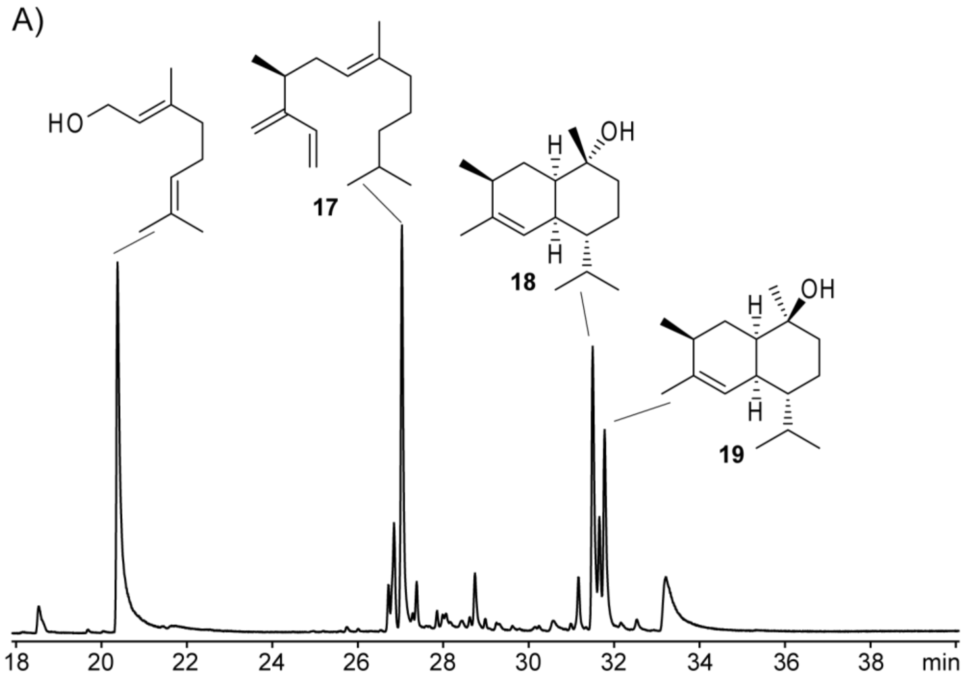

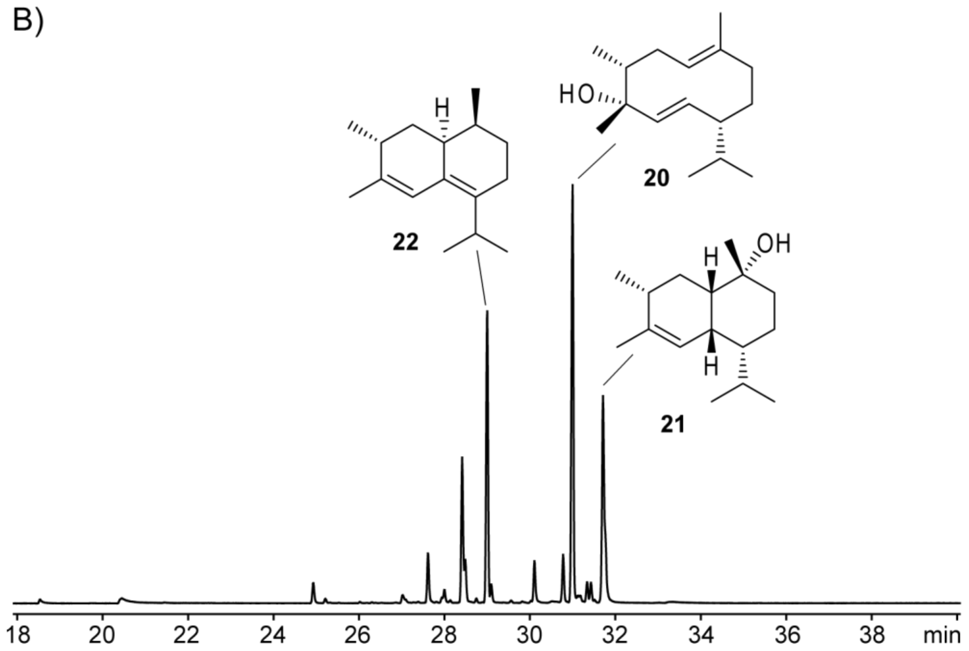

Figure S53. Total ion chromatograms of the products obtained with FPPS and TmS from GPP and A) **8a** and B) **8b**.

Large scale incubation experiments with recombinant FPPS and TmS

For large scale reactions purified protein fractions of FPPS (45 mL in elution buffer, obtained from 4 L of *E. coli* expression culture) and of TmS (45 mL in elution buffer, obtained from 4 L of *E. coli* expression culture), GPP (100 mg in 20 mL 25 mM NH_4HCO_3), **8a** or **8b** (100 mg in 20 mL 25 mM NH_4HCO_3), and incubation buffer (200 mL) were mixed and incubated at 28 °C overnight. The products were extracted with pentane (3x 100 mL) and the extracts were concentrated under reduced pressure. The products were obtained by purification through silica gel chromatography.

(*S*)-4-Methyl-(*E*)-β-farnesene (17). Yield: 1.0 mg (0.005 mmol, 1%). TLC: R_f [pentane] = 0.50. $[\alpha]_D^{20}$ = –6.0 (CH_2Cl_2, *c* 0.1). HRMS (ToF): *m*/*z* = 218.2030 ($[M]^+$, calc. for $[C_{16}H_{26}]^+$ 218.2029). GC (HP5-MS): *I* = 1501. MS (EI, 70 eV): *m*/*z* (%) = 218 (4), 203 (1), 175 (7), 161 (2), 147 (21), 134 (12), 119 (6), 107 (34), 93 (18), 79 (33), 69 (100), 53 (18), 41 (59). IR (diamond ATR): $\tilde{\nu}$ / cm^{-1} = 3089 (w), 2962 (m), 2925 (s), 2872 (m), 2854 (m), 1668 (m), 1594 (m), 1454 (m), 1376 (m), 1260 (w), 1103 (w), 1019 (w), 990 (m), 892 (s), 806 (m). NMR data are given in Table S7 and Figures S54–S60.

(3*S*)-3-Methyl-T-muurolol (18). Yield: 1.8 mg (0.008 mmol, 1%). TLC: R_f [pentane/Et_2O(1/1)] = 0.61. $[\alpha]_D^{20}$ = +18.3 (CH_2Cl_2, *c* 0.18). HRMS (ToF): *m*/*z* = 236.2134 ($[M]^+$, calc. for $[C_{16}H_{28}O]^+$ 236.2135). GC (HP5-MS): *I* = 1693. MS (EI, 70 eV): *m*/*z* (%) = 263 (4), 218 (28), 203 (4), 193 (4), 175 (48), 166 (6), 148 (8), 135 (56), 119 (42), 109 (100), 93 (52), 77 (22), 69 (28), 55 (25), 43 (83). IR (diamond ATR): $\tilde{\nu}$ / cm^{-1} = 3360 (w), 2956 (s), 2928 (s), 2869 (m), 1739 (w), 1722 (w), 1671 (w), 1462 (m), 1448 (m), 1369 (m), 1302 (w), 1260 (w), 1232 (w), 1195 (w), 1146 (w), 1100 (w), 1083 (w), 1057 (m), 1042 (m), 1032 (m), 1016 (w), 975 (w), 928 (w), 910 (w), 884 (w), 855 (w), 800 (m). NMR data are given in Table S8 and Figures S61–S67.

(3*S*)-3-Methyl-10-*epi*-T-muurolol (19). Yield: 1.2 mg (0.005 mmol, 1%). TLC: R_f [pentane/Et_2O(1/1)] = 0.52. $[\alpha]_D^{20}$ = +4.2 (CH_2Cl_2, *c* 0.12). HRMS (ToF): *m*/*z* = 236.2137 ($[M]^+$, calc. for $[C_{16}H_{28}O]^+$ 236.2135). GC (HP5-MS): *I* = 1706. MS (EI, 70 eV): *m*/*z* (%) = 236 (4), 218 (47), 203 (8), 193 (4), 175 (51), 166 (7), 151 (10), 135 (58), 119 (37), 109 (100), 93 (36), 79 (15), 67 (19), 55 (17), 43 (46). IR (diamond ATR): $\tilde{\nu}$ / cm^{-1} = 3390 (w), 2959 (s), 2928 (s), 2869 (s), 2855 (s), 1716 (w), 1673 (m),1623 (w), 1455 (m), 1378 (m), 1260 (m), 1117 (m), 1041 (m), 1017 (m), 958 (w), 920 (w), 845 (w), 802 (m). NMR data are given in Table S9 and Figures S68–S74.

(3*R*,4*R*,7*R*)-3-Methyl-4-hydroxygermacra-1(10),5-diene (20). Yield: 0.8 mg (0.003 mmol, 1%). TLC: R_f [pentane/Et_2O(5/1)] = 0.57. $[\alpha]_D^{20}$ = +26.2 (CH_2Cl_2, *c* 0.08). HRMS (ToF): *m*/*z* = 218.2029 ($[M-H_2O]^+$, calc. for $[C_{16}H_{26}]^+$ 218.2029). GC (HP5-MS): *I* = 1667. MS (EI, 70 eV): *m*/*z* (%) = 236 (1), 218 (11), 203 (5), 193 (3), 175 (15), 161 (3), 149 (5), 135 (11), 123 (21), 109 (21), 95 (29), 81 (100), 69 (15), 55 (17), 43 (48). IR (diamond ATR): $\tilde{\nu}$ / cm^{-1} = 3459 (w), 2958 (s), 2925 (s), 2872 (m), 2854 (m), 1738 (m), 1675 (w), 1451 (w), 1367 (m), 1229 (m), 1217 (m), 1205 (m), 1084 (w), 1034 (w), 1016 (w), 1005 (w), 984 (w), 799 (w), 528 (w), 515 (w). NMR data are given in Table S10 and Figures S75–S81.

(3*R*)-3-Methyl-1,6-*diepi*-T-muurolol (21). Yield: 1.1 mg (0.005 mmol, 1%). TLC: R_f [pentane /Et_2O(1/1)] = 0.52. $[\alpha]_D^{20}$ = +28.2 (CH_2Cl_2, *c* 0.11). HRMS (ToF): *m*/*z* = 218.2032 ($[M-H_2O]^+$, calc. for $[C_{16}H_{26}]^+$ 218.2029). GC (HP5-MS): *I* = 1701. MS (EI, 70 eV): *m*/*z* (%) = 218 (18), 203 (4), 175 (100), 161 (3), 147 (5), 133 (66), 119 (46), 109 (18), 93 (27), 81 (10), 69 (13), 55 (12), 43 (33). IR (diamond ATR): $\tilde{\nu}$ / cm^{-1} = 3470 (w), 2958 (s), 2931 (s), 2872 (s), 1738 (s), 1676 (w), 1458 (w), 1368 (s), 1229 (m), 1205 (m), 1120 (m), 1037 (w), 1016 (w), 858 (w), 830 (w), 529 (w), 511 (w), 417 (w). NMR data are given in Table S11 and Figures S82–S88.

(1*S*,3*R*,10*S*)-3-Methylzonarene (22). Yield: 1.4 mg (0.006 mmol, 1%). R_f [pentane] = 0.71. $[\alpha]_D^{20}$ = +37.1 (CH_2Cl_2, *c* 0.14). HRMS (ToF): *m*/*z* = 218.2026 ($[M-H_2O]^+$, calc. for $[C_{16}H_{26}]^+$ 218.2029). GC (HP5-MS): *I* = 1581. MS (EI, 70 eV): *m*/*z* (%) = 218 (47), 203 (18), 175 (100), 161 (13), 147 (17), 133 (42), 119 (49), 105 (34), 95 (36), 81 (12), 69 (13), 55 (16), 41 (23). IR (diamond ATR): $\tilde{\nu}$ / cm^{-1} = 2959 (s), 2923 (s), 2871 (m), 2854 (m), 1739 (m), 1658 (w), 1632

(w), 1458 (m), 1378 (m), 1366 (m), 1228 (w), 1207 (w), 1094 (w), 1017 (w), 857 (w), 805 (w). NMR data are given in Table S12 and Figures S89–S95.

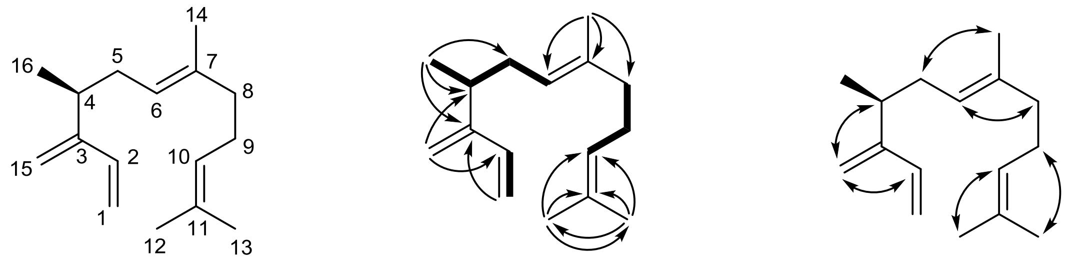

Table S7. NMR data of (*S*)-4-methyl-(*E*)-β-farnesene (**17**) in C_6D_6 recorded at 298 K.

C[a]		^{13}C[b]	^{1}H[b]
1	CH_2	112.79	5.26 (d, *J* = 17.6, H_Z)
			4.96 (d, *J* = 10.7, H_E)
2	CH	139.30	6.33 (dd, *J* = 17.6, 11.1)
3	C_q	151.87	–
4	CH	35.07	2.51 (sext, *J* = 6.8)
5	CH_2	34.84	2.28 (m, H_β)
			2.09 (m, H_α)
6	CH	123.65	5.24 (tm, *J* = 7.2)
7	C_q	135.87	–
8	CH_2	40.29	2.07 (m, 2H)
9	CH_2	27.16	2.15 (m, 2H)
10	CH	124.97	5.21 (tm, *J* = 6.9)
11	C_q	131.12	–
12	CH_3	25.87	1.67 (s)
13	CH_3	17.75	1.55 (s)
14	CH_3	16.28	1.57 (s)
15	CH_2	113.58	5.04 (br s, H_E)
			4.96 (br s, H_Z)
16	CH_3	19.72	1.09 (d, *J* = 6.9)

[a] Carbon numbering as shown in the structure above the table (bold lines: ^{1}H,^{1}H-COSY correlations, single headed arrows: HMBC correlations, double headed arrows: NOESY correlations). [b] Chemical shifts δ in ppm, multiplicity: s = singlet, d = doublet, t = triplet, sext = sextet, m = multiplet, br = broad, coupling constants *J* are given in Hertz.

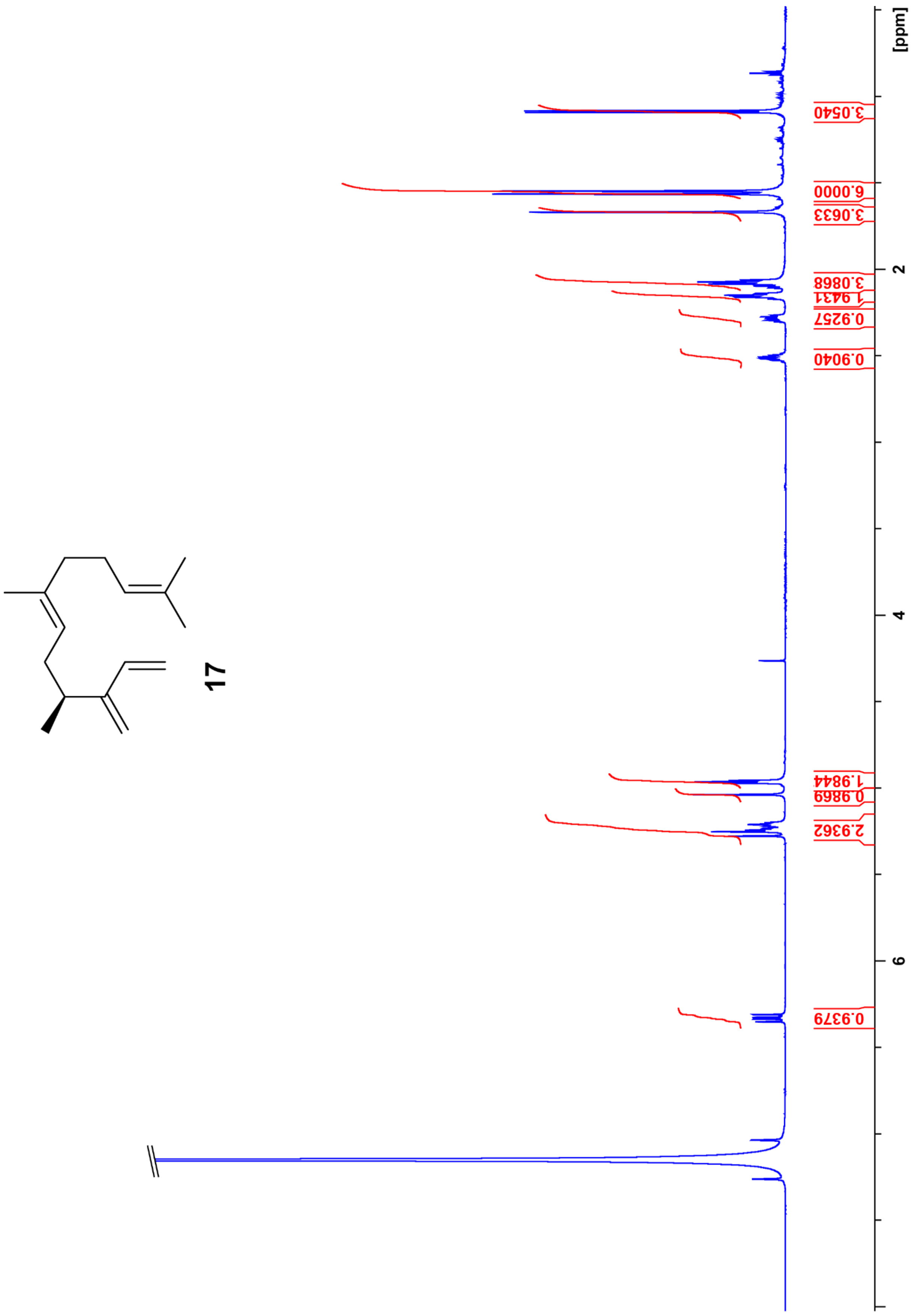

Figure S54. ^{1}H-NMR spectrum of **17** (700 MHz, C_6D_6).

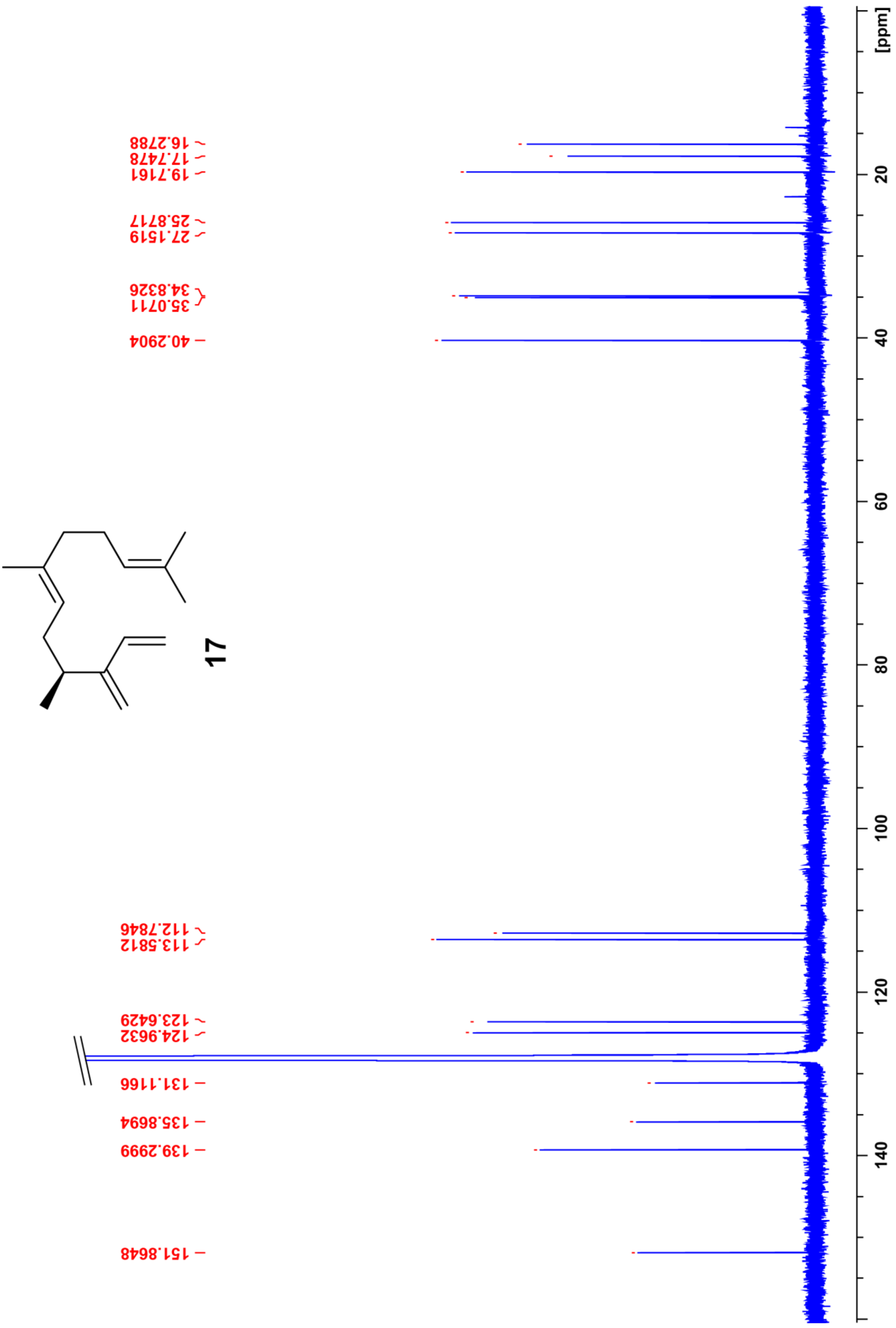

Figure S55. ^{13}C-NMR spectrum of **17** (175 MHz, C_6D_6).

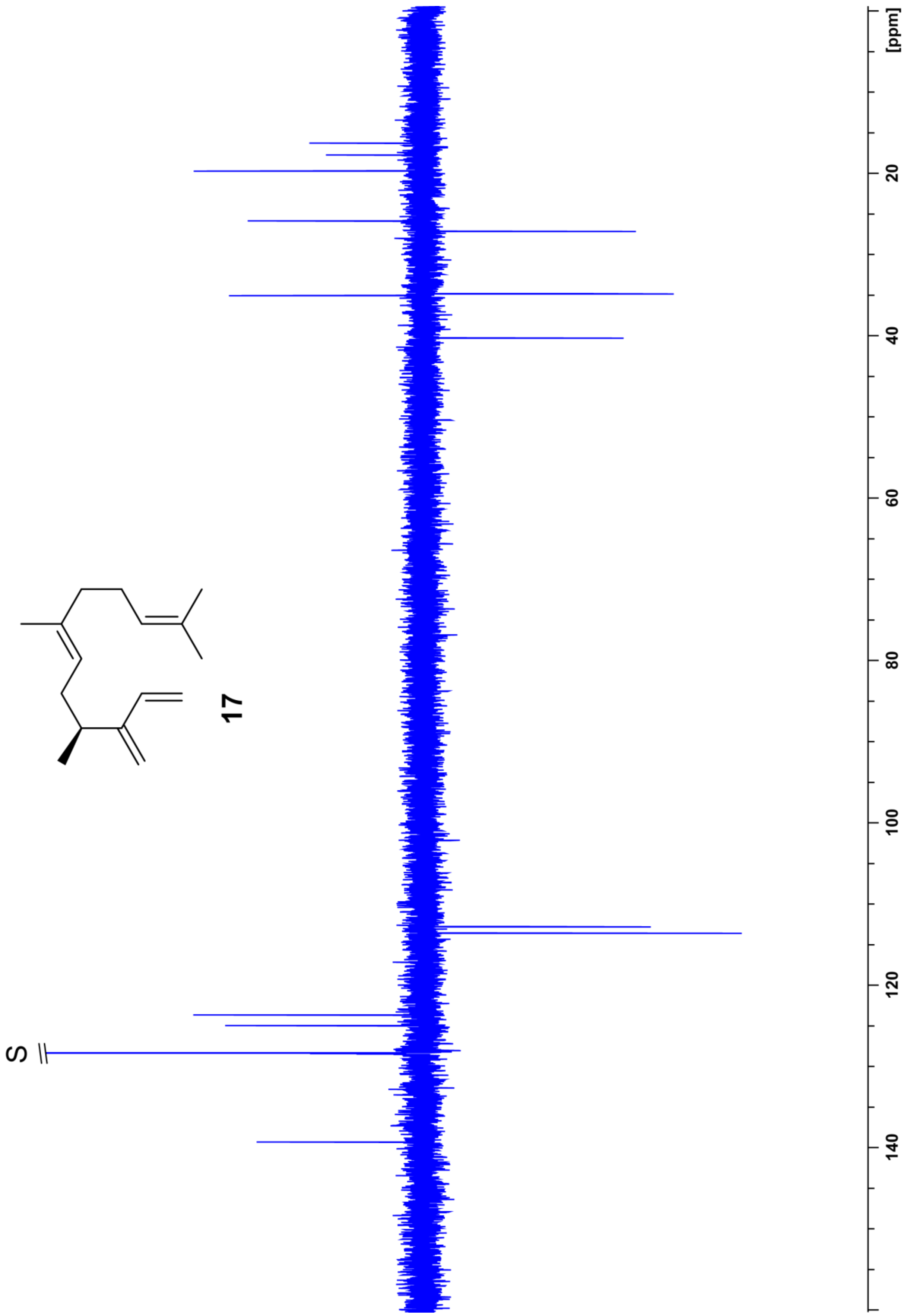

Figure S56. ^{13}C-DEPT spectrum of **17** (175 MHz, C_6D_6).

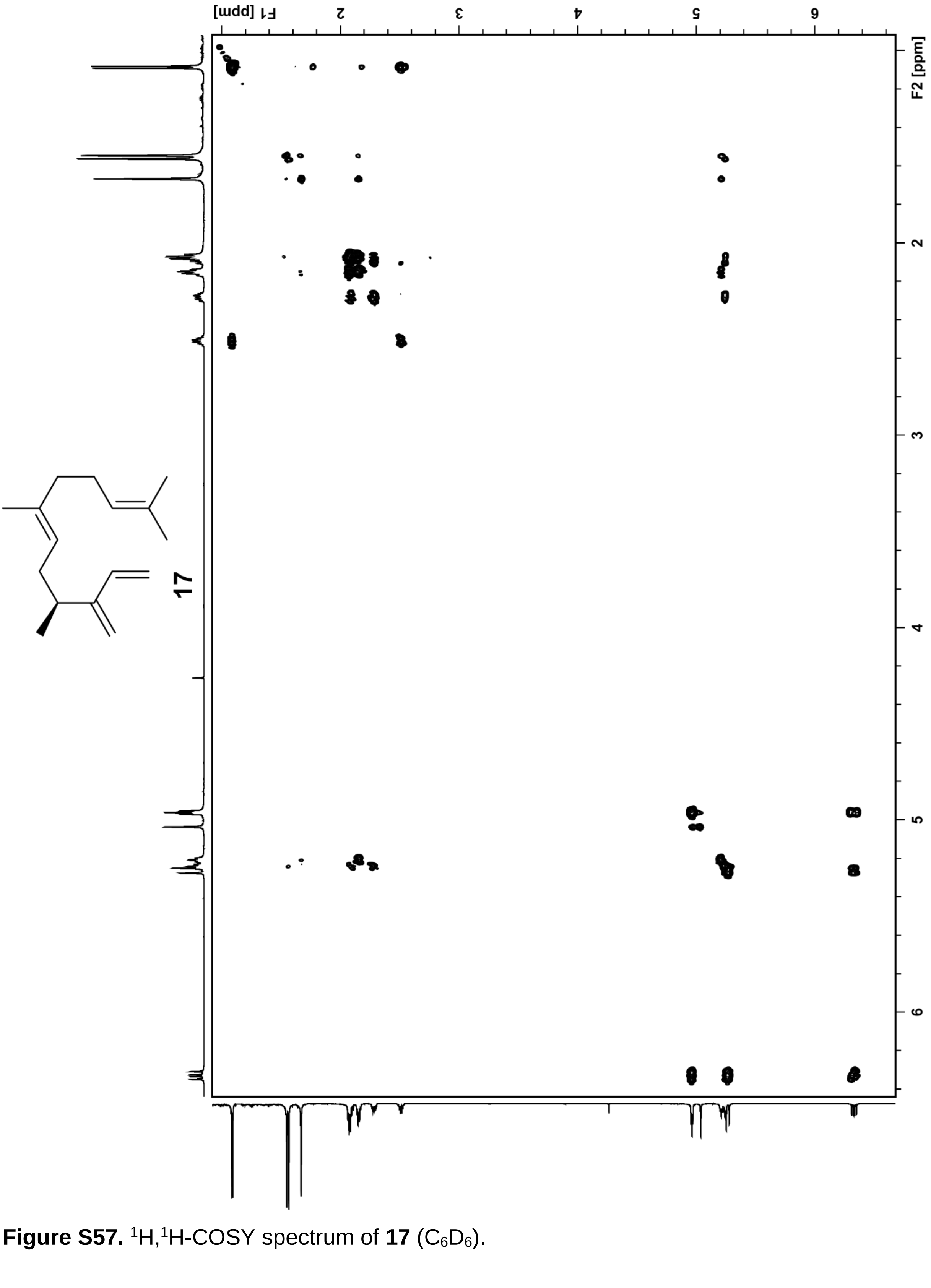

Figure S57. ^{1}H,^{1}H-COSY spectrum of **17** (C_6D_6).

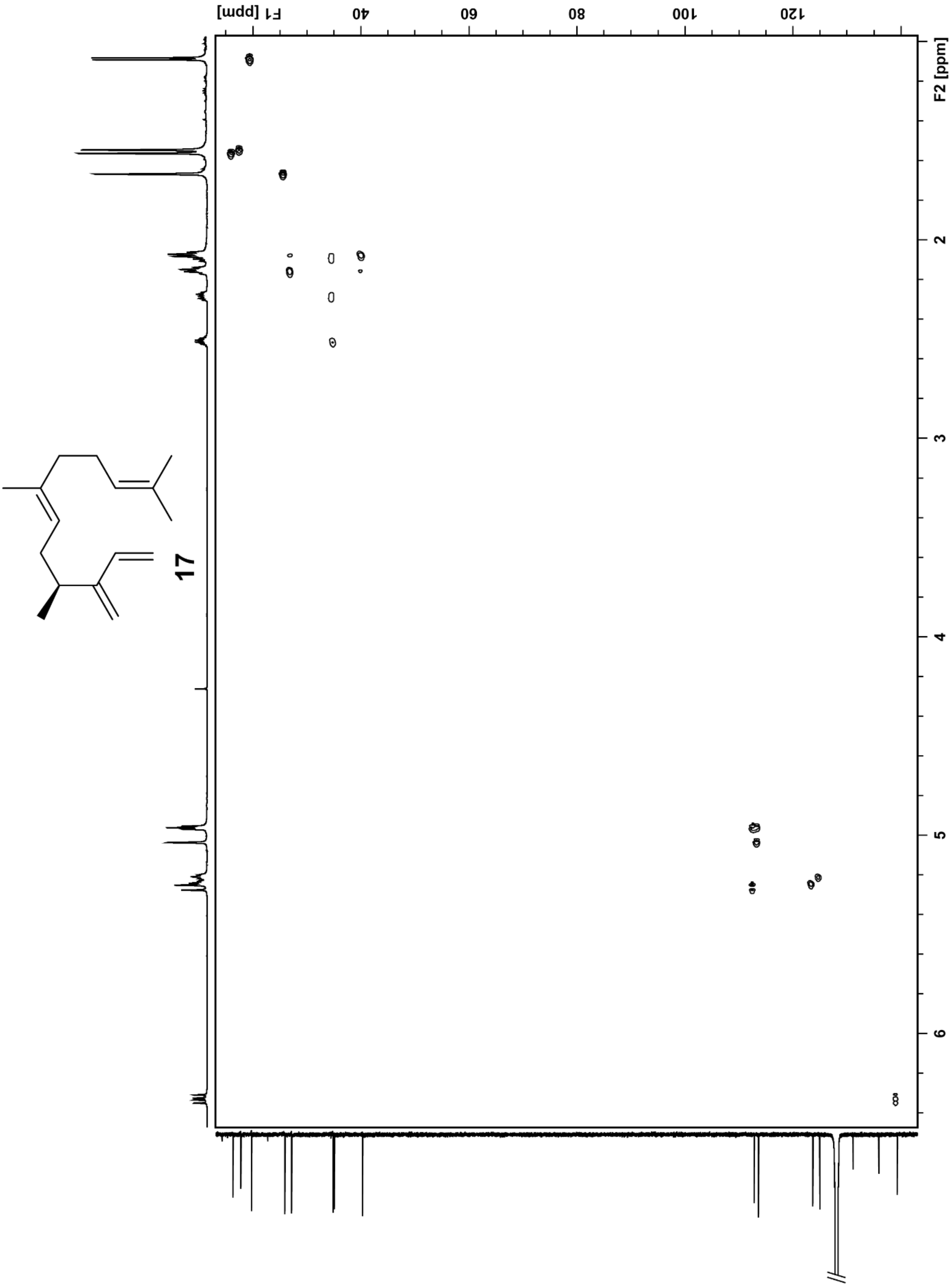

Figure S58. HSQC spectrum of **17** (C_6D_6).

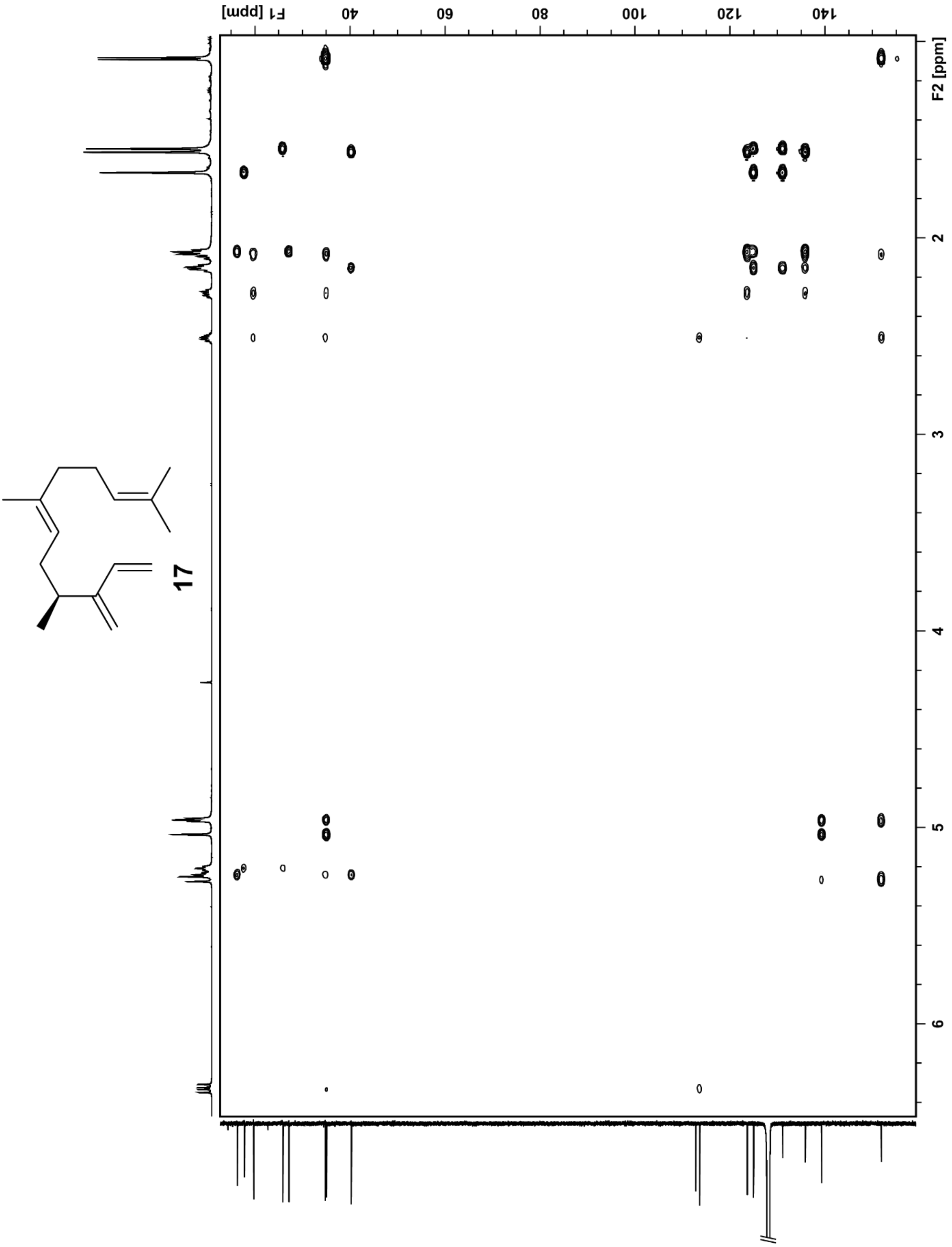

Figure S59. HMBC spectrum of **17** (C_6D_6).

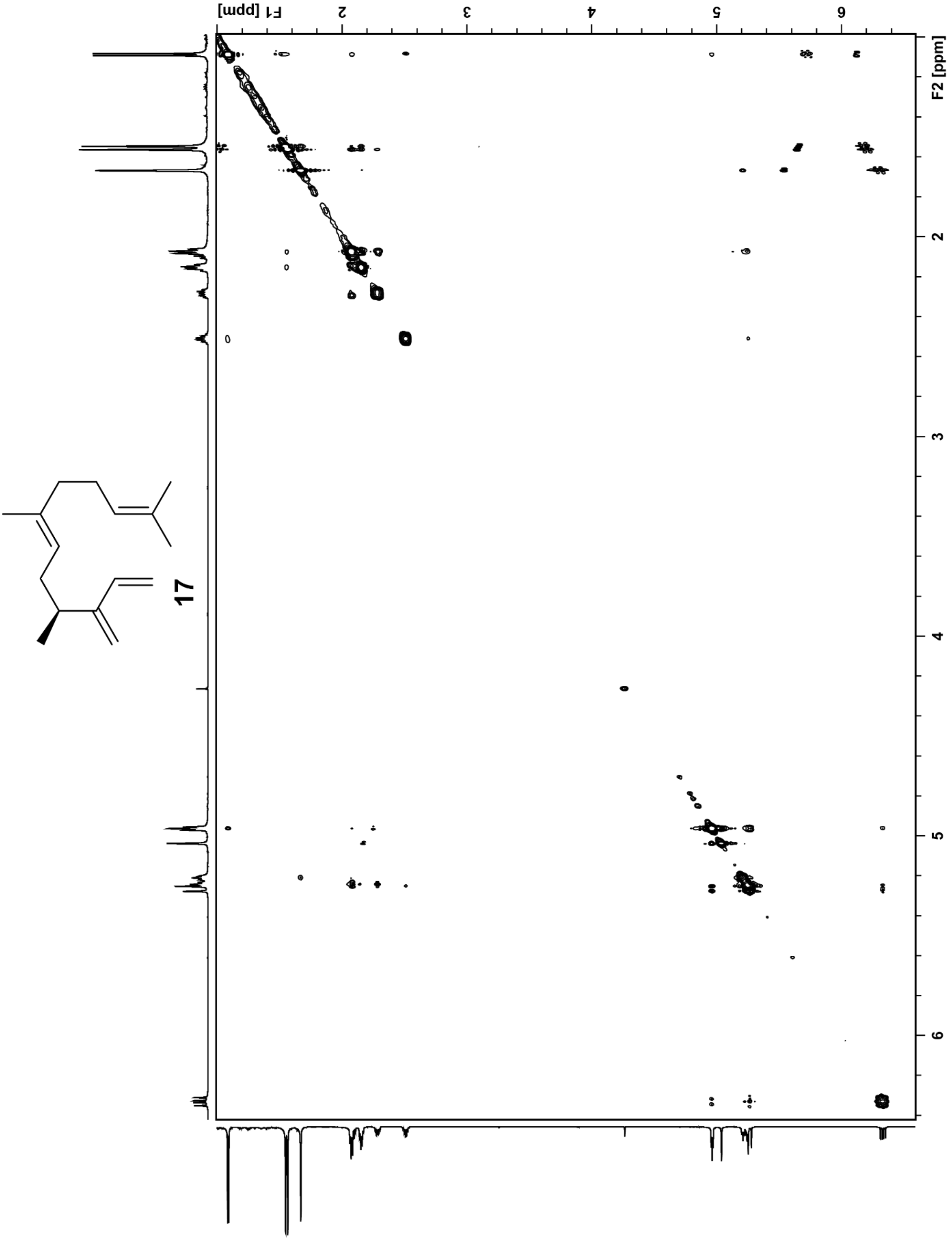

Figure S60. NOESY spectrum of **17** (C_6D_6).

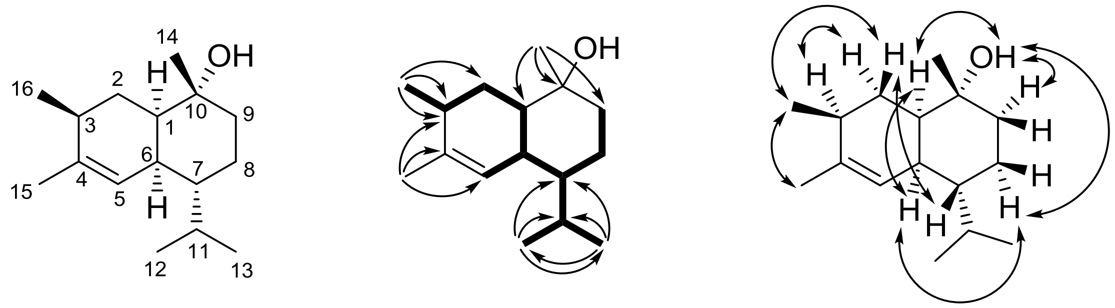

Table S8. NMR data of (3*S*)-3-methyl-T-muurolol (**18**) in C_6D_6 recorded at 298 K.

C[a]		^{13}C[b]	^{1}H[b]
1	CH	46.69	1.55 (m)
2	CH$_2$	31.46	1.49 (m, H$_\alpha$)
			1.18 (ddd, J = 13.1, 13.1, 10.8, H$_\beta$)
3	CH	36.08	1.98 (m)
4	C$_q$	137.53	–
5	CH	126.68	5.67 (d, J = 5.8)
6	CH	35.40	2.44 (m)
7	CH	44.97	1.27 (m)
8	CH$_2$	19.80	1.50 (m, H$_\alpha$)
			1.29 (m, H$_\beta$)
9	CH$_2$	34.92	1.41 (m, H$_\alpha$)
			1.31 (m, H$_\beta$)
10	C$_q$	71.41	–
11	CH	27.26	2.07 (m)
12	CH$_3$	15.77	0.89 (d, J = 6.9)
13	CH$_3$	21.88	0.90 (d, J = 6.9)
14	CH$_3$	29.58	1.05 (s)
15	CH$_3$	21.60	1.65 (m)
16	CH$_3$	20.31	0.94 (d, J = 7.0)
–	OH	–	0.61

[a] Carbon numbering as shown in the structure above the table (bold lines: ^{1}H,^{1}H-COSY correlations, single headed arrows: HMBC correlations, double headed arrows: NOESY correlations). [b] Chemical shifts δ in ppm, multiplicity: s = singlet, d = doublet, sept = septet, m = multiplet, br = broad, coupling constants J are given in Hertz.

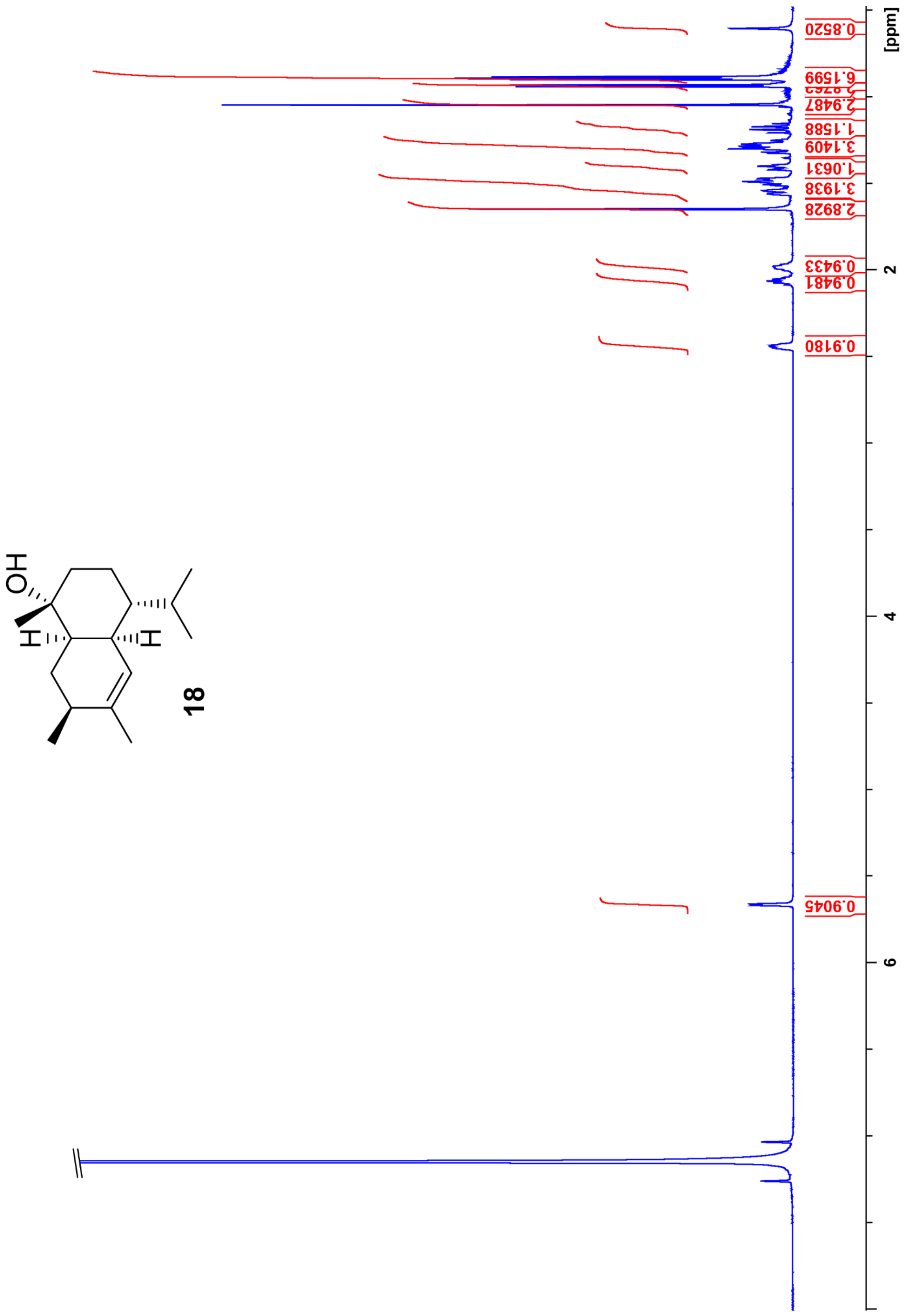

Figure S61. ^{1}H-NMR spectrum of **18** (700 MHz, C_6D_6).

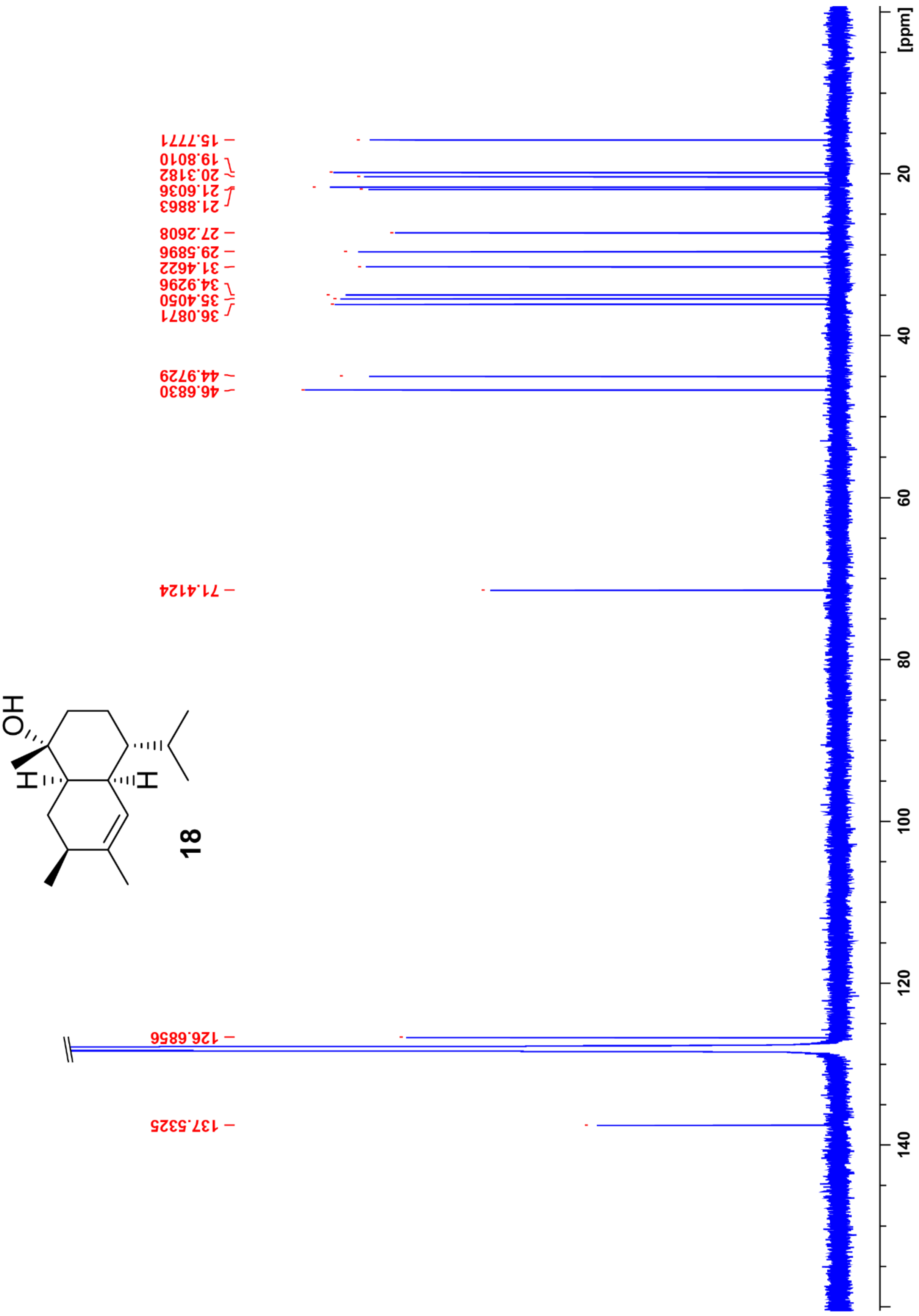

Figure S62. ^{13}C-NMR spectrum of **18** (175 MHz, C_6D_6).

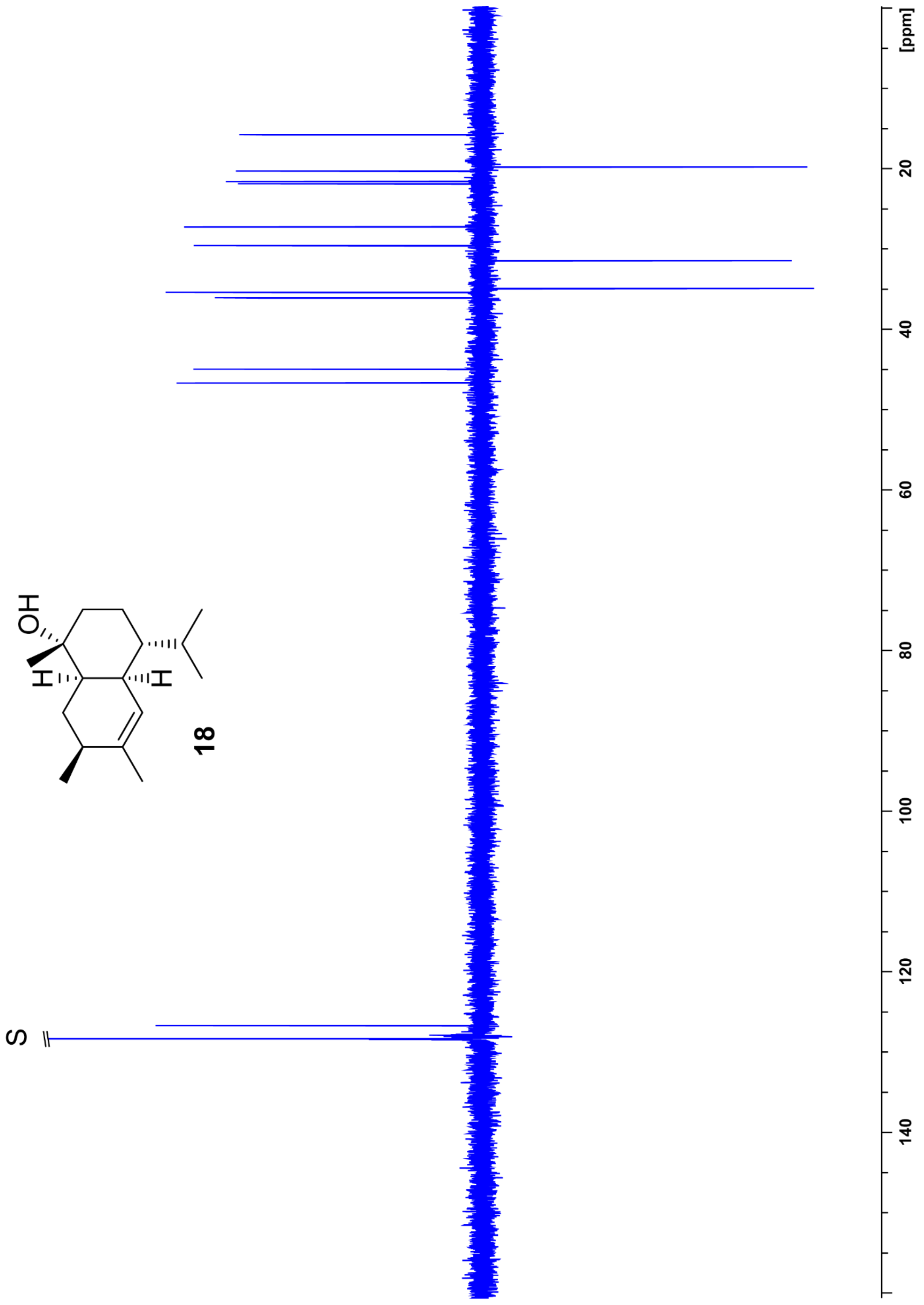

Figure S63. ^{13}C-DEPT spectrum of **18** (175 MHz, C_6D_6).

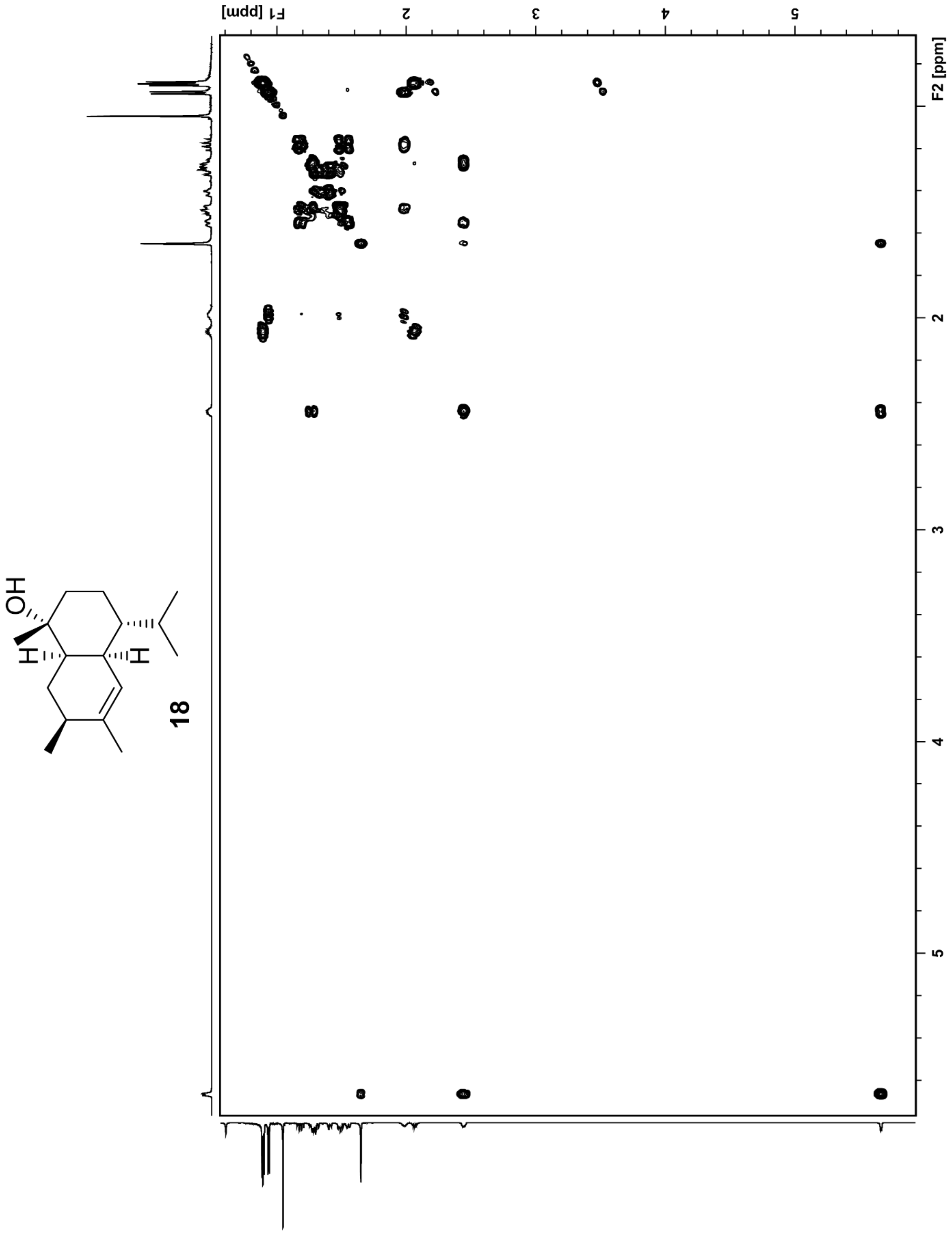

Figure S64. $^{1}H,^{1}H$-COSY spectrum of **18** (C_6D_6).

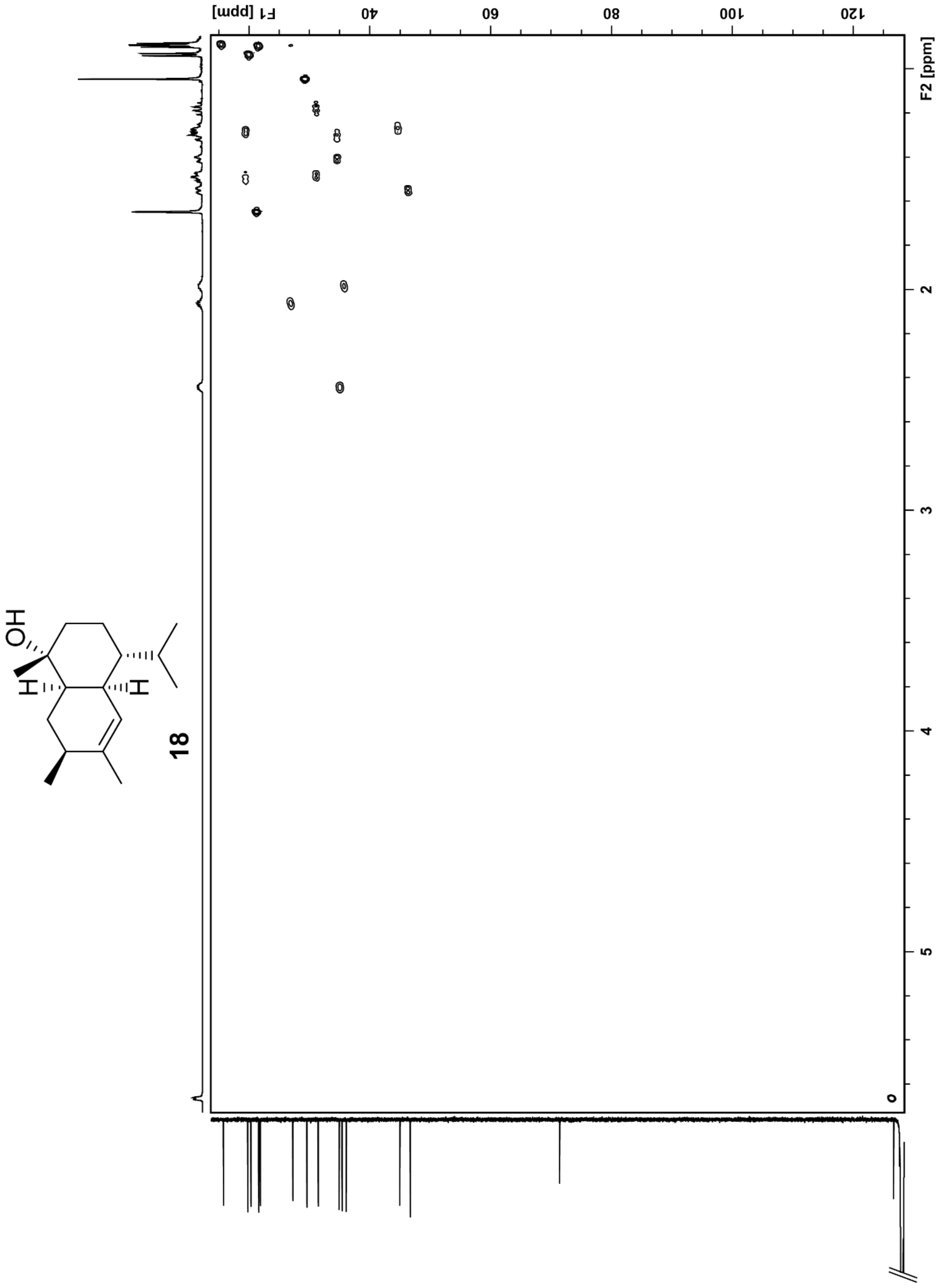

Figure S65. HSQC spectrum of **18** (C_6D_6).

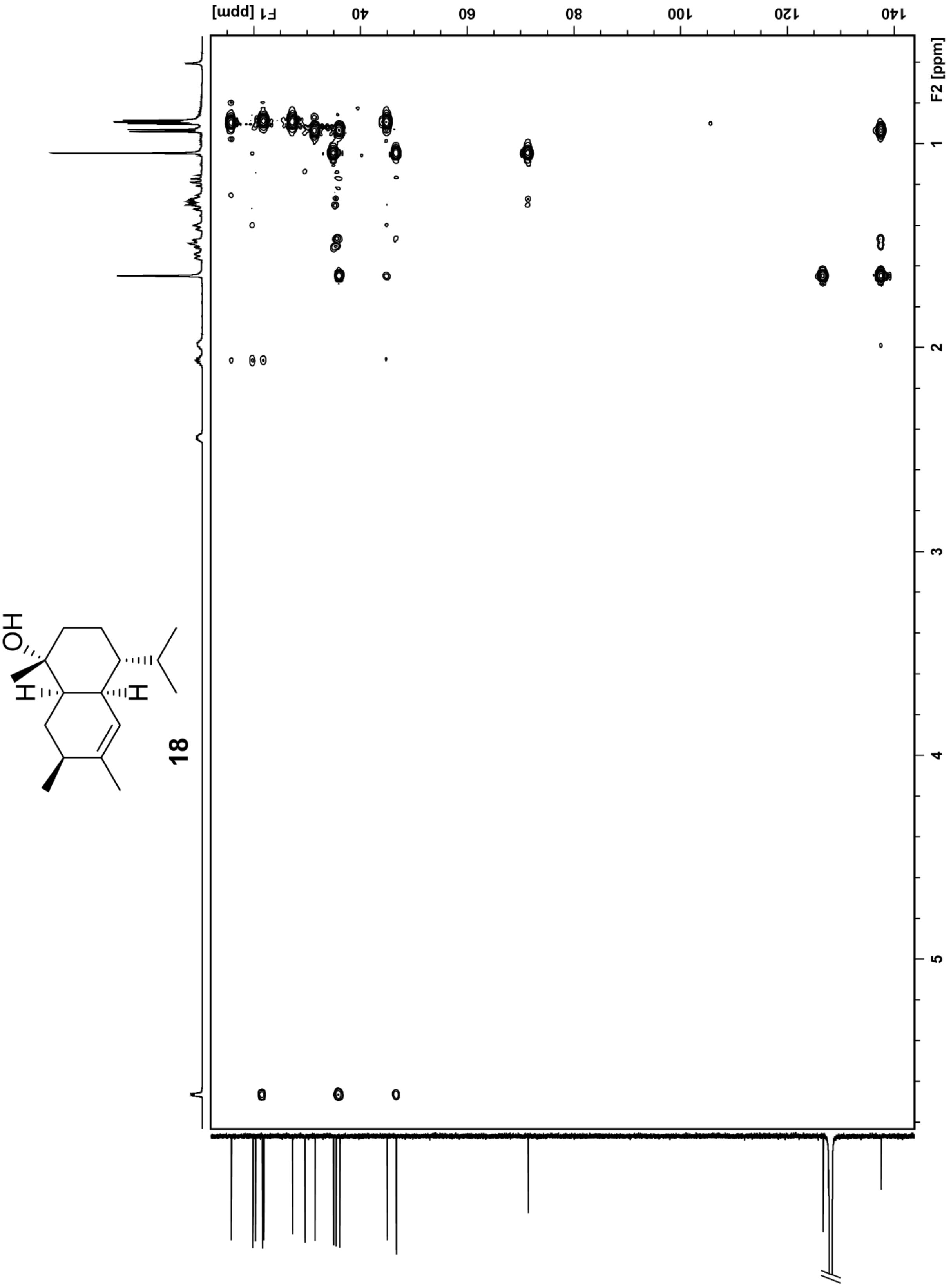

18

Figure S66. HMBC spectrum of **18** (C_6D_6).

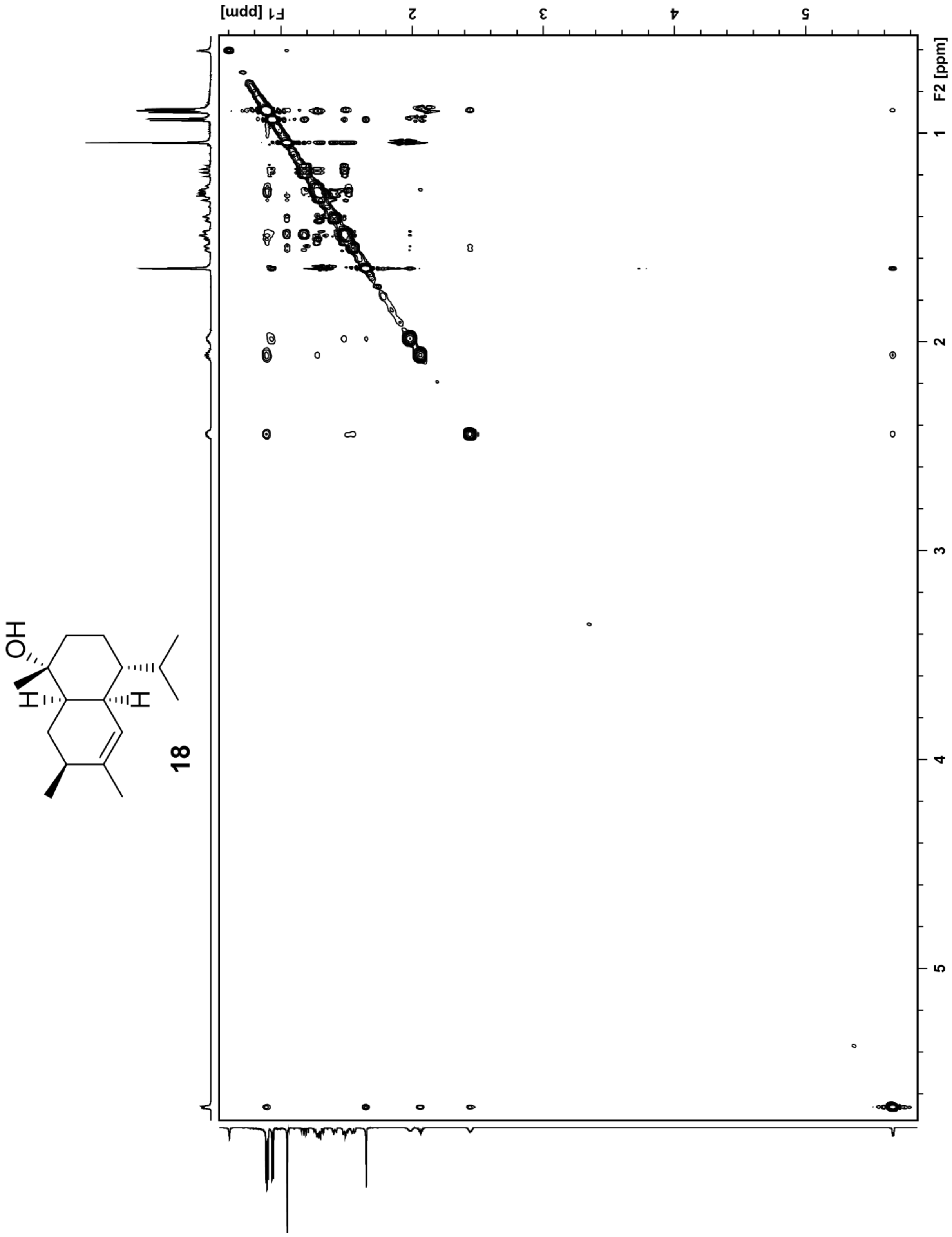

Figure S67. NOESY spectrum of **18** (C_6D_6).

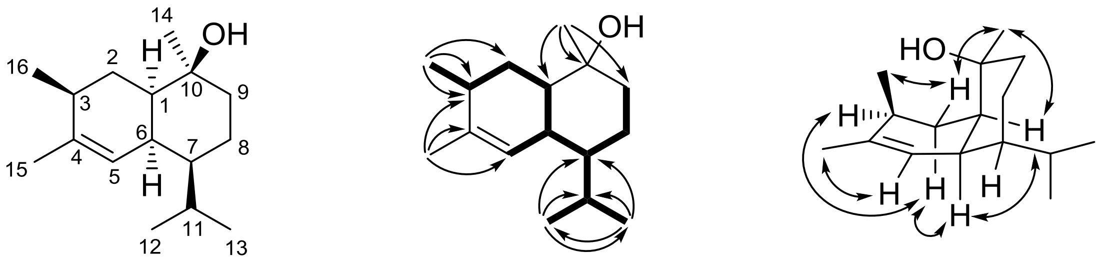

Table S9. NMR data of (3*S*)-3-methyl-10-*epi*-T-muurolol (**19**) in C_6D_6 recorded at 298 K.

C[a]		^{13}C[b]	^{1}H[b]
1	CH	44.92	1.34 (m)
2	CH_2	30.17	1.79 (m, H_β)
			1.34 (m, H_α)
3	CH	34.13	2.02 (m)
4	C_q	138.96	–
5	CH	122.96	5.54 (br s)
6	CH	40.80	1.63 (m)
7	CH	47.02	0.98 (m)
8	CH_2	22.36	1.43 (m)
			0.98 (m)
9	CH_2	42.75	1.66 (m)
			1.30 (m)
10	C_q	71.28	–
11	CH	26.37	2.11 (m)
12	CH_3	21.68	0.85 (d, J = 7.0)
13	CH_3	15.27	0.73 (d, J = 7.0)
14	CH_3	20.97	0.97 (s)
15	CH_3	22.64	1.67 (br s)
16	CH_3	19.69	1.07 (d, J = 7.3)
–	OH	–	0.70 (br s)

[a] Carbon numbering as shown in the structure above the table (bold lines: $^{1}H,^{1}H$-COSY correlations, single headed arrows: HMBC correlations, double headed arrows: NOESY correlations). [b] Chemical shifts δ in ppm, multiplicity: s = singlet, d = doublet, sept = septet, m = multiplet, br = broad, coupling constants J are given in Hertz.

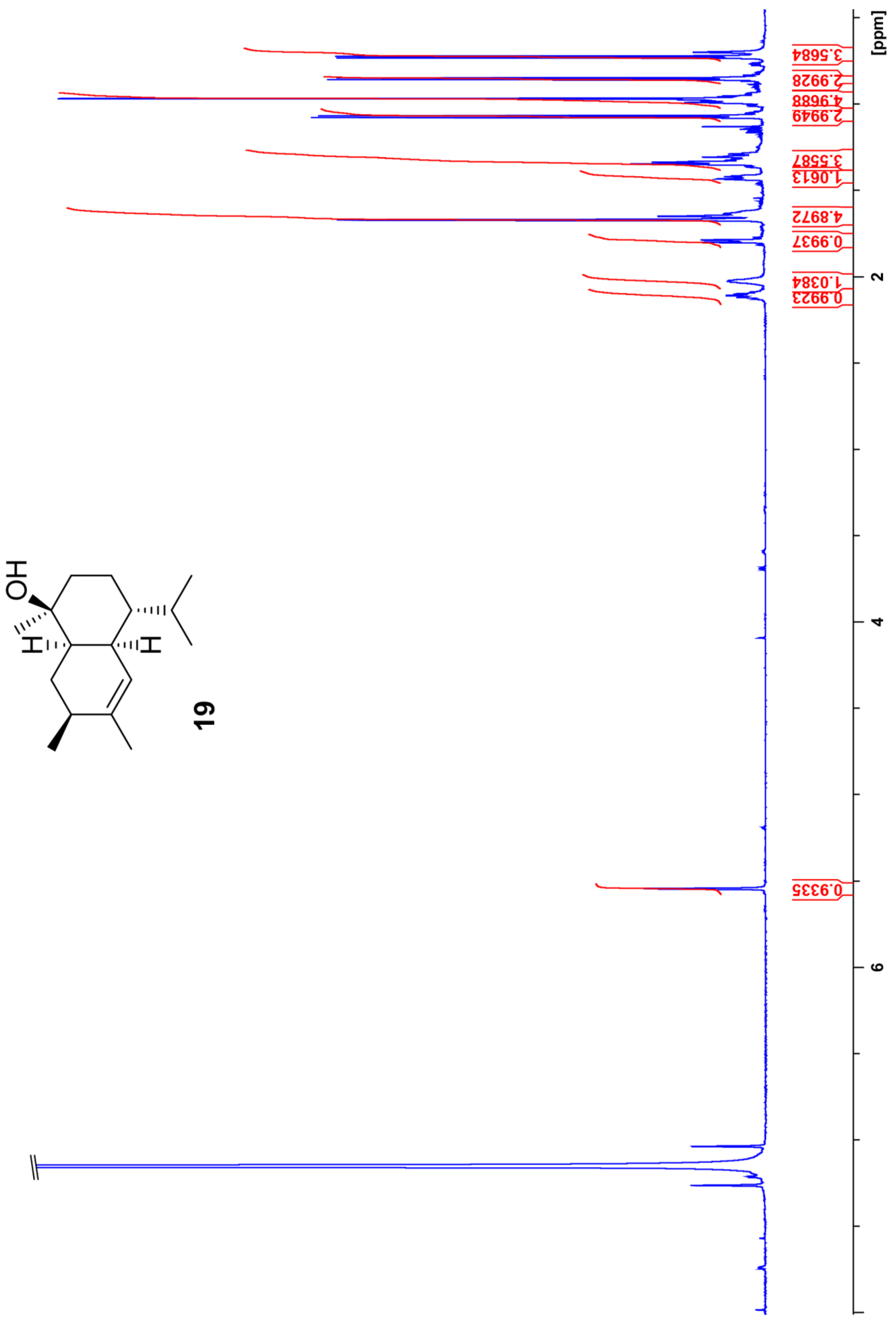

Figure S68. ^{1}H-NMR spectrum of **19** (700 MHz, C_6D_6).

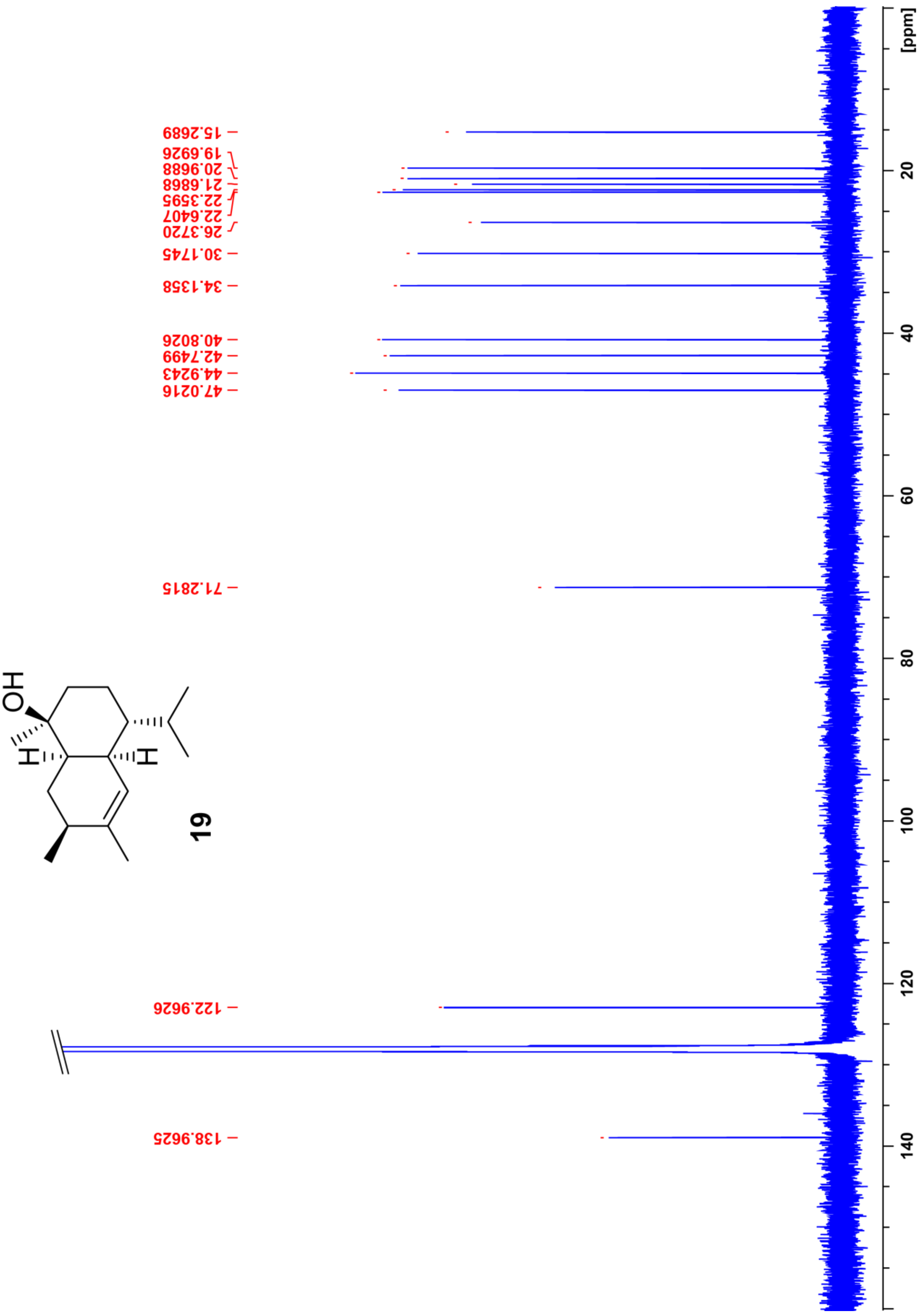

Figure S69. ^{13}C-NMR spectrum of **19** (175 MHz, C_6D_6).

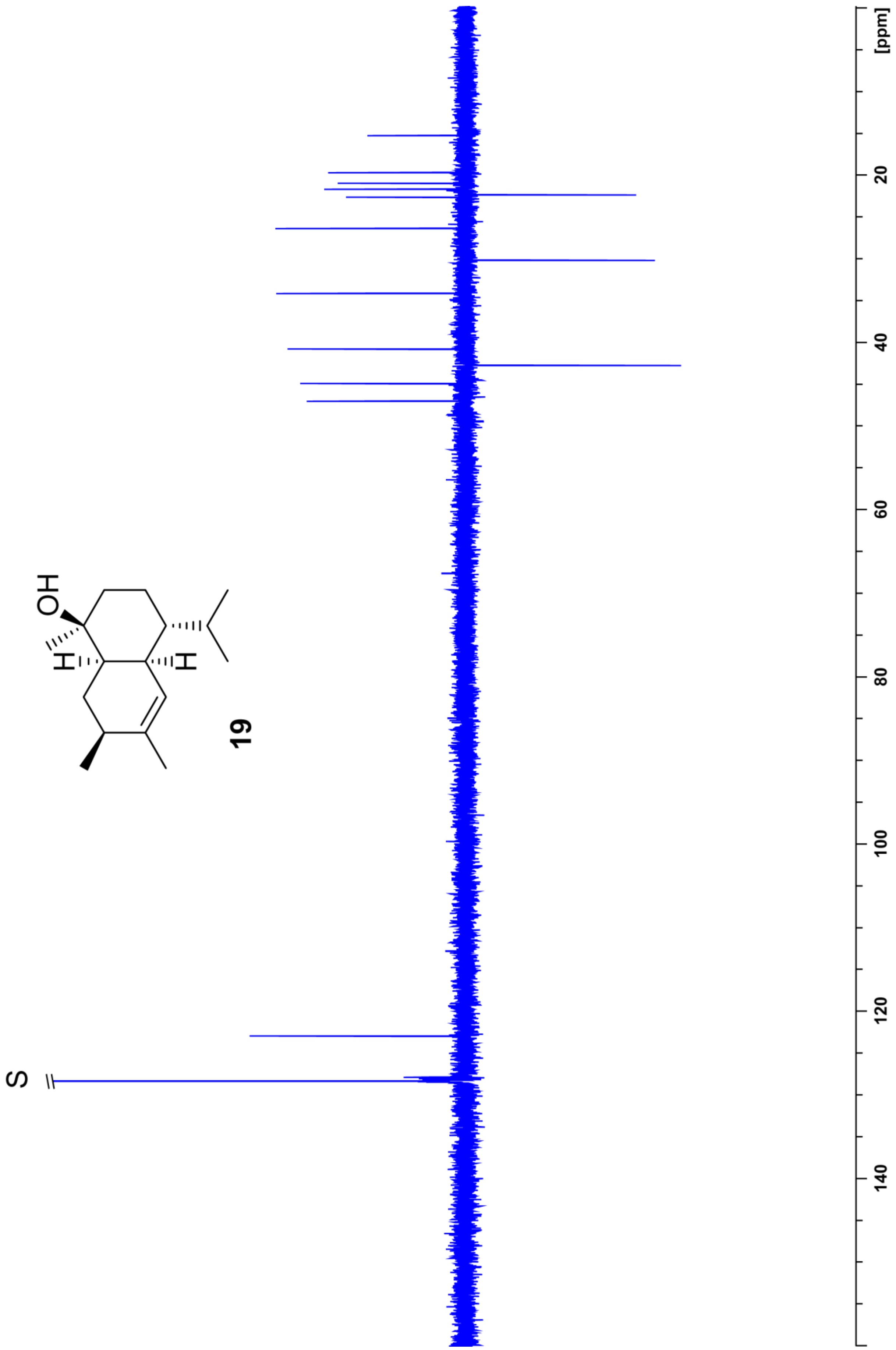

Figure S70. ^{13}C-DEPT spectrum of **19** (175 MHz, C_6D_6).

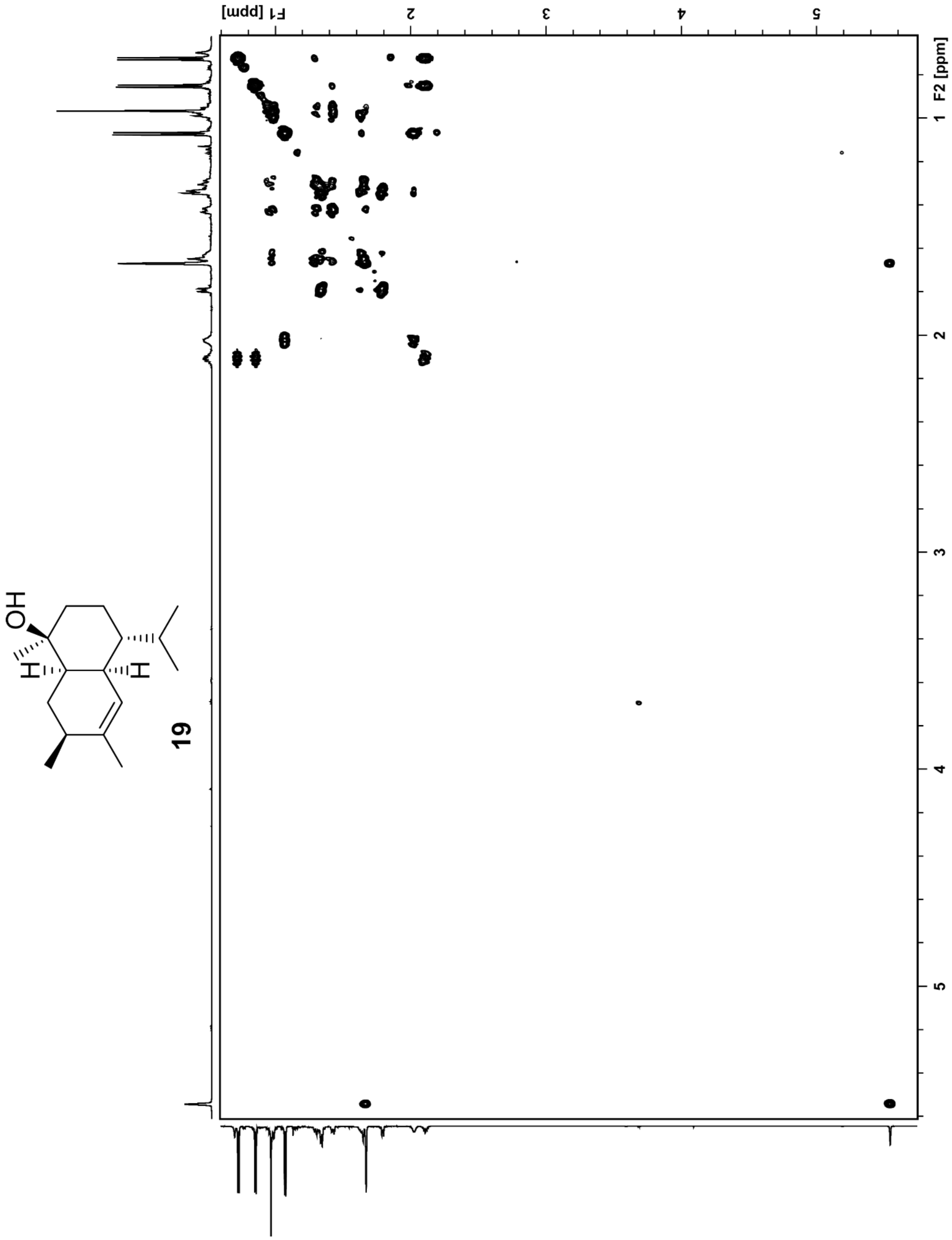

Figure S71. ^{1}H,^{1}H-COSY spectrum of **19** (C_6D_6).

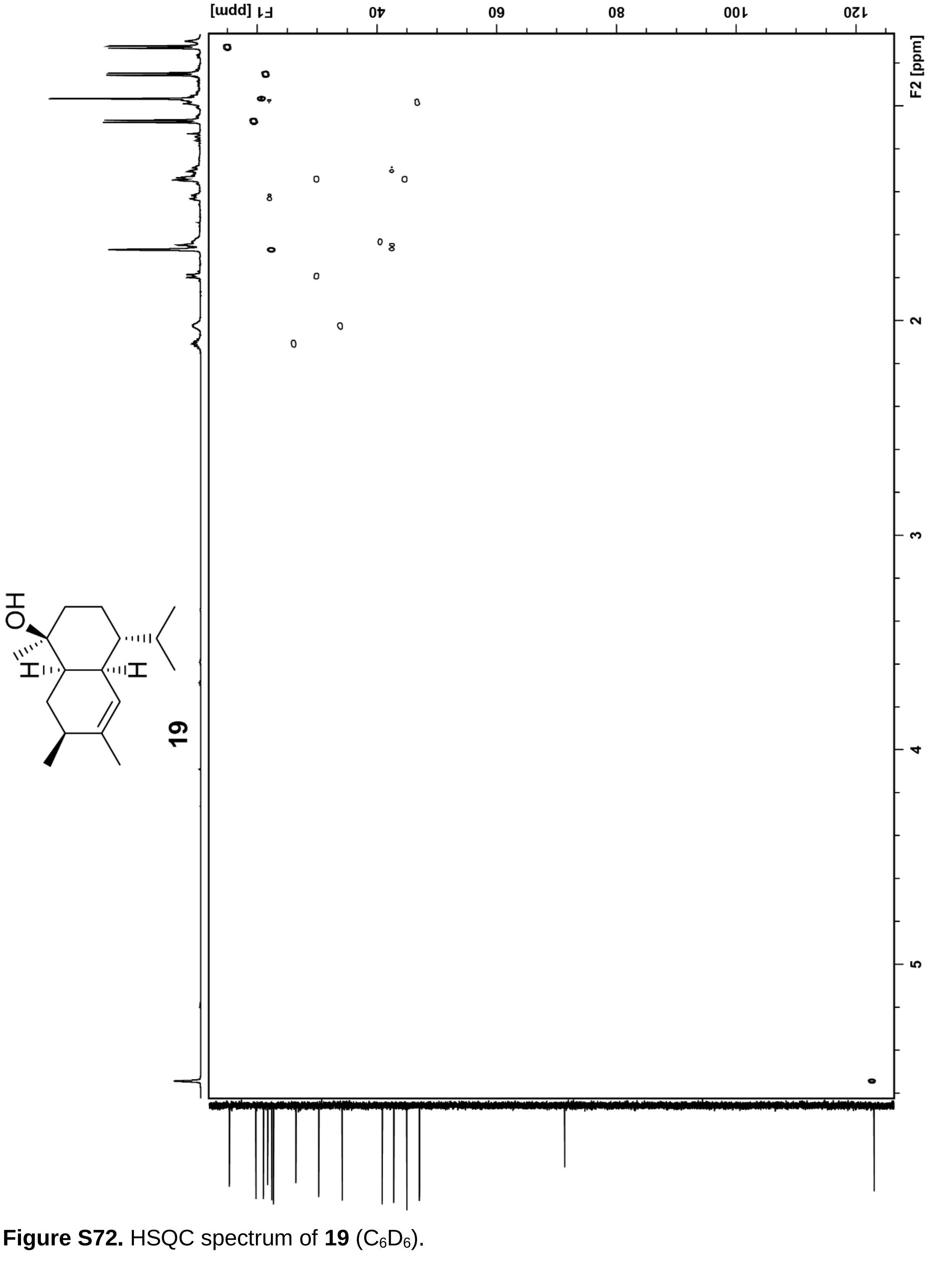

Figure S72. HSQC spectrum of **19** (C_6D_6).

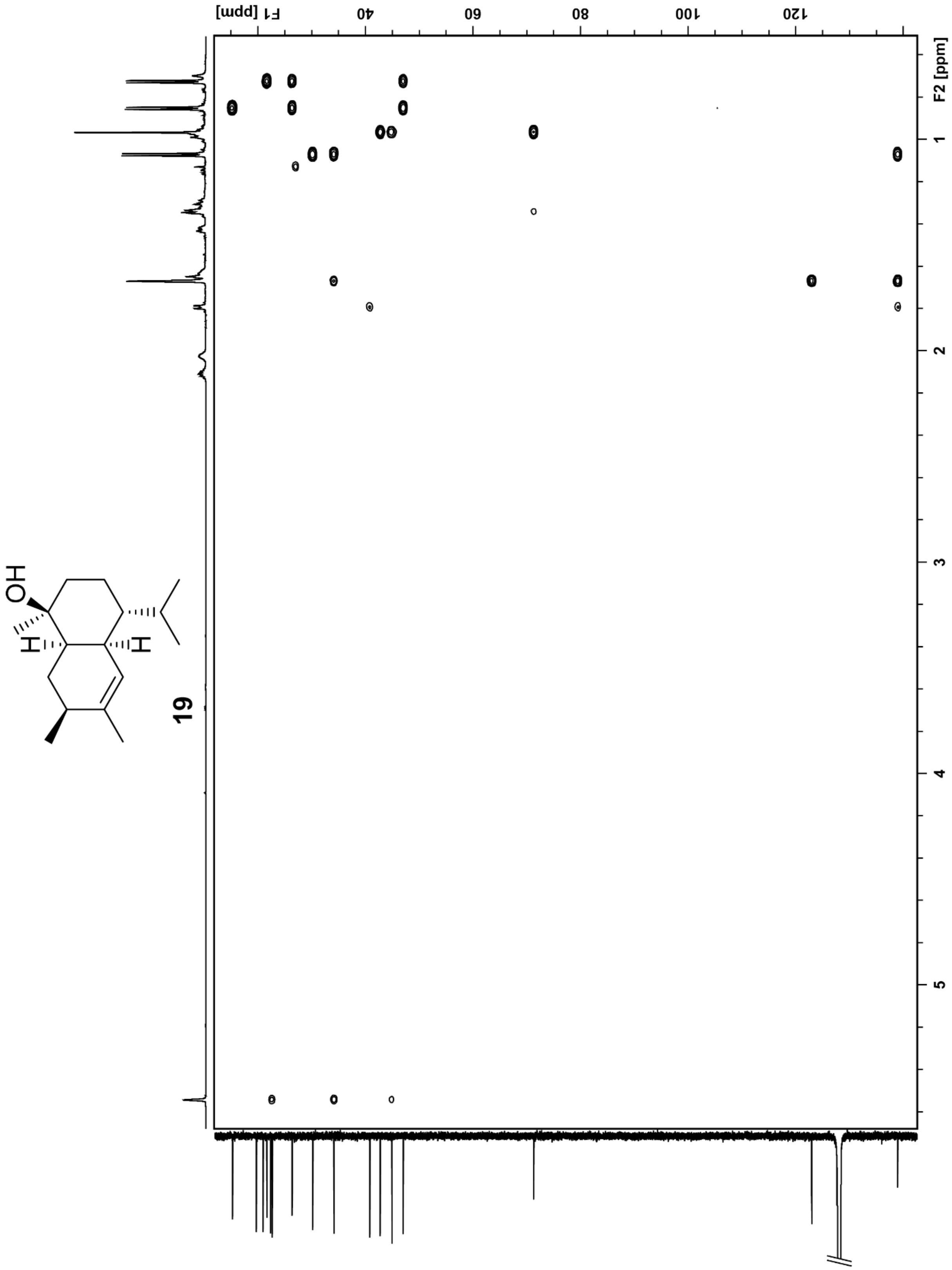

Figure S73. HMBC spectrum of **19** (C_6D_6).

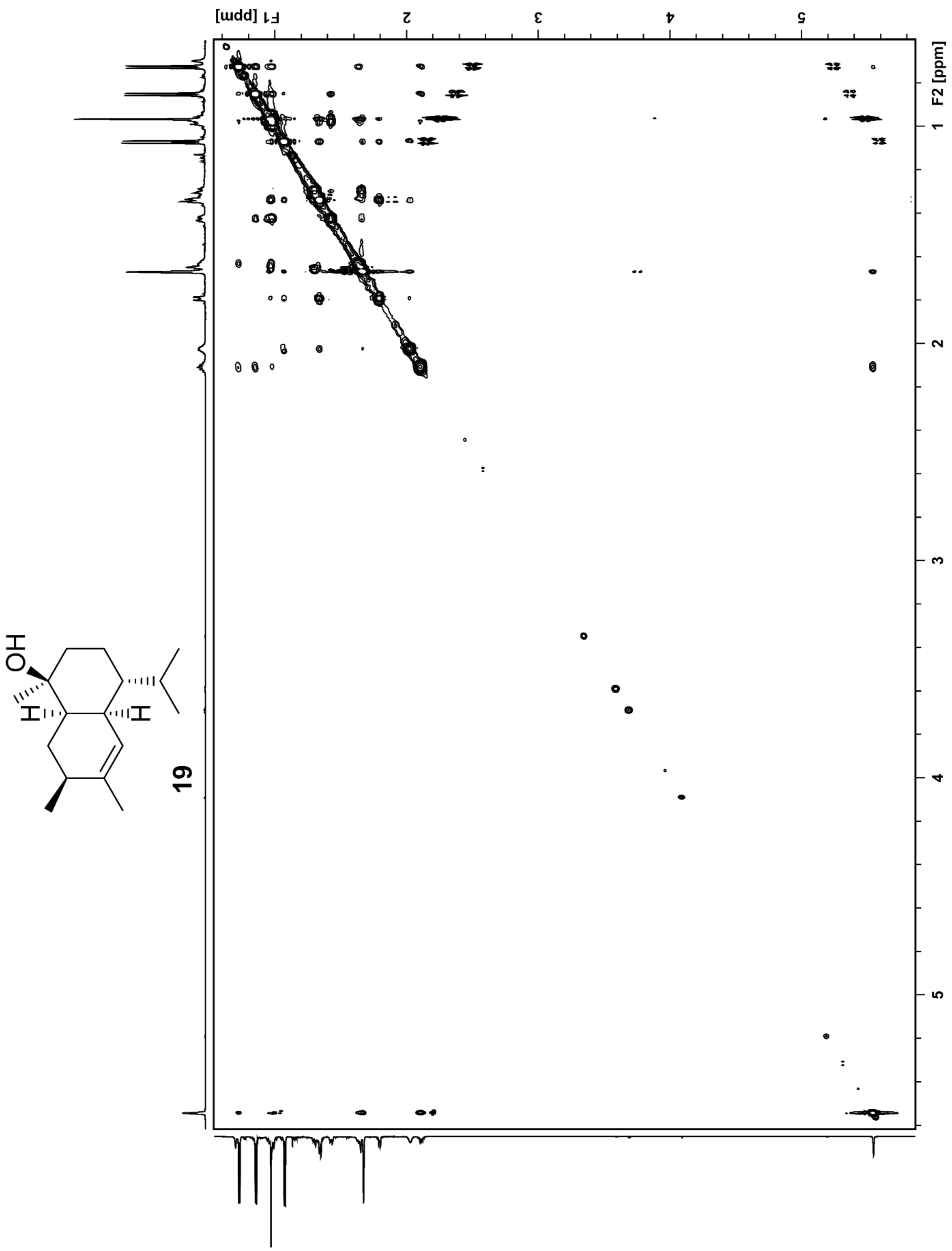

Figure S74. NOESY spectrum of **19** (C_6D_6).

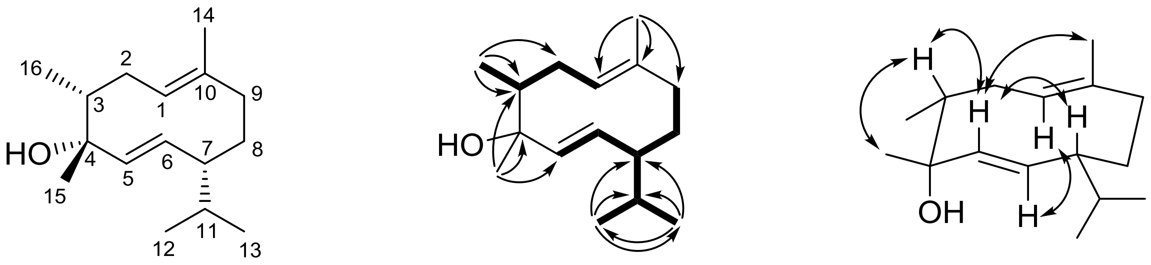

Table S10. NMR data of (3*R*,4*R*,7*R*)-3-methyl-4-hydroxygermacra-1(10),5-diene (**20**) in C_6D_6 recorded at 298 K.

C[a]		^{13}C[b]	^{1}H[b]
1	CH	130.55	5.05 (br d, *J* = 12.0)
2	CH_2	33.67	2.41 (dt, *J* = 14.0, 11.7, H_α)
			1.80 (br d, *J* = 14.0, H_β)
3	CH	41.25	1.30 (m)
4	C_q	74.14	–
5	CH	142.21	5.06 (d, *J* = 15.6)
6	CH	123.88	5.18 (dd, *J* = 15.6, 9.8)
7	CH	53.24	1.91 (m)
8	CH_2	25.42	1.20 (m, 2H)
9	CH_2	41.73	2.23 (m, 2H)
10	C_q	131.22	–
11	CH	33.52	1.38 (m)
12	CH_3	20.93	0.899 (d, *J* = 6.7)
13	CH_3	19.27	0.86 (d, *J* = 6.9)
14	CH_3	16.90	1.48 (s)
15	CH_3	28.46	1.05 (s)
16	CH_3	16.24	0.904 (d, *J* = 6.8)
–	OH	–	0.87 (br s)

[a] Carbon numbering as shown in the structure above the table (bold lines: $^{1}H,^{1}H$-COSY correlations, single headed arrows: HMBC correlations, double headed arrows: NOESY correlations). [b] Chemical shifts δ in ppm, multiplicity: s = singlet, d = doublet, m = multiplet, br = broad, coupling constants *J* are given in Hertz.

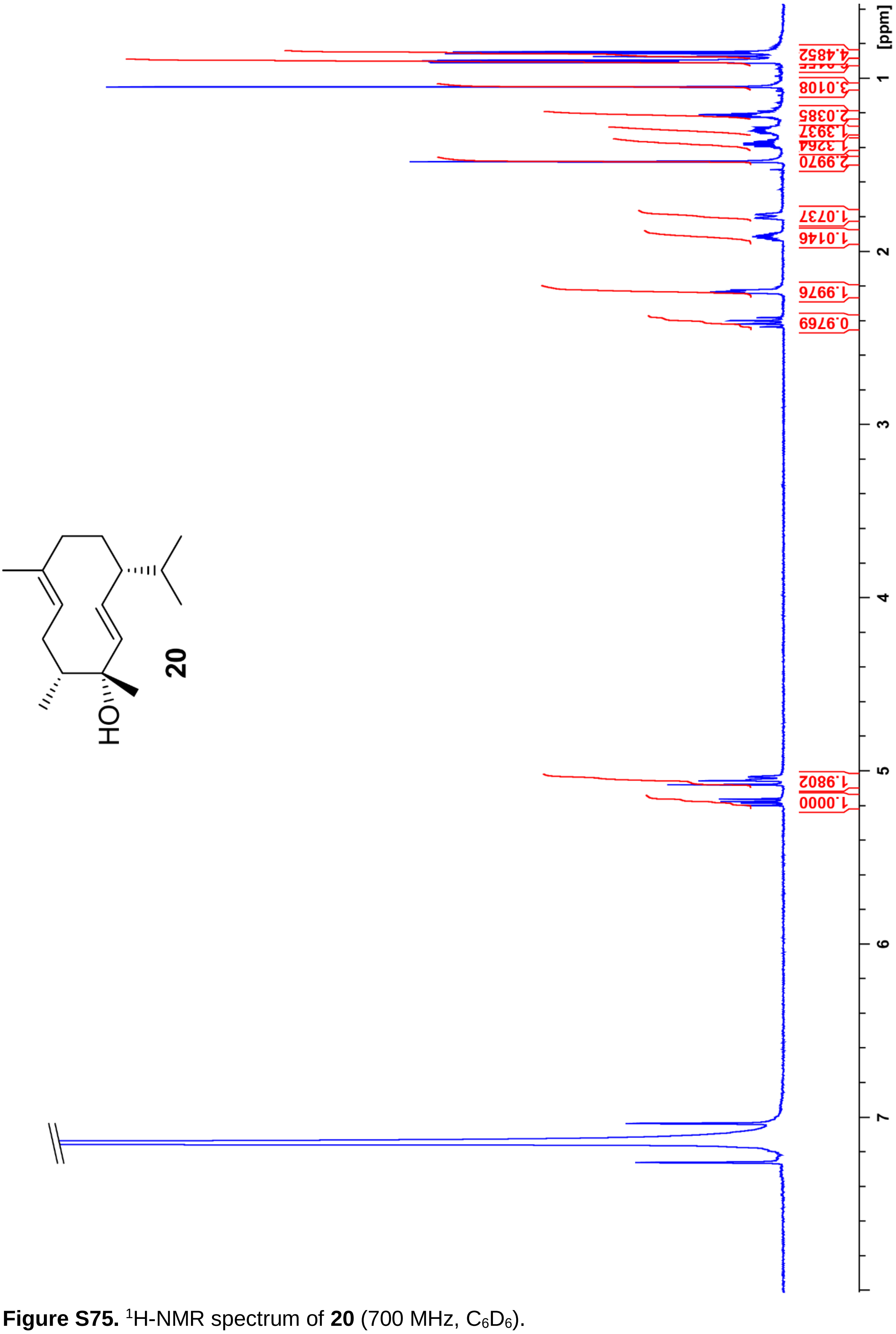

Figure S75. ^{1}H-NMR spectrum of **20** (700 MHz, C_6D_6).

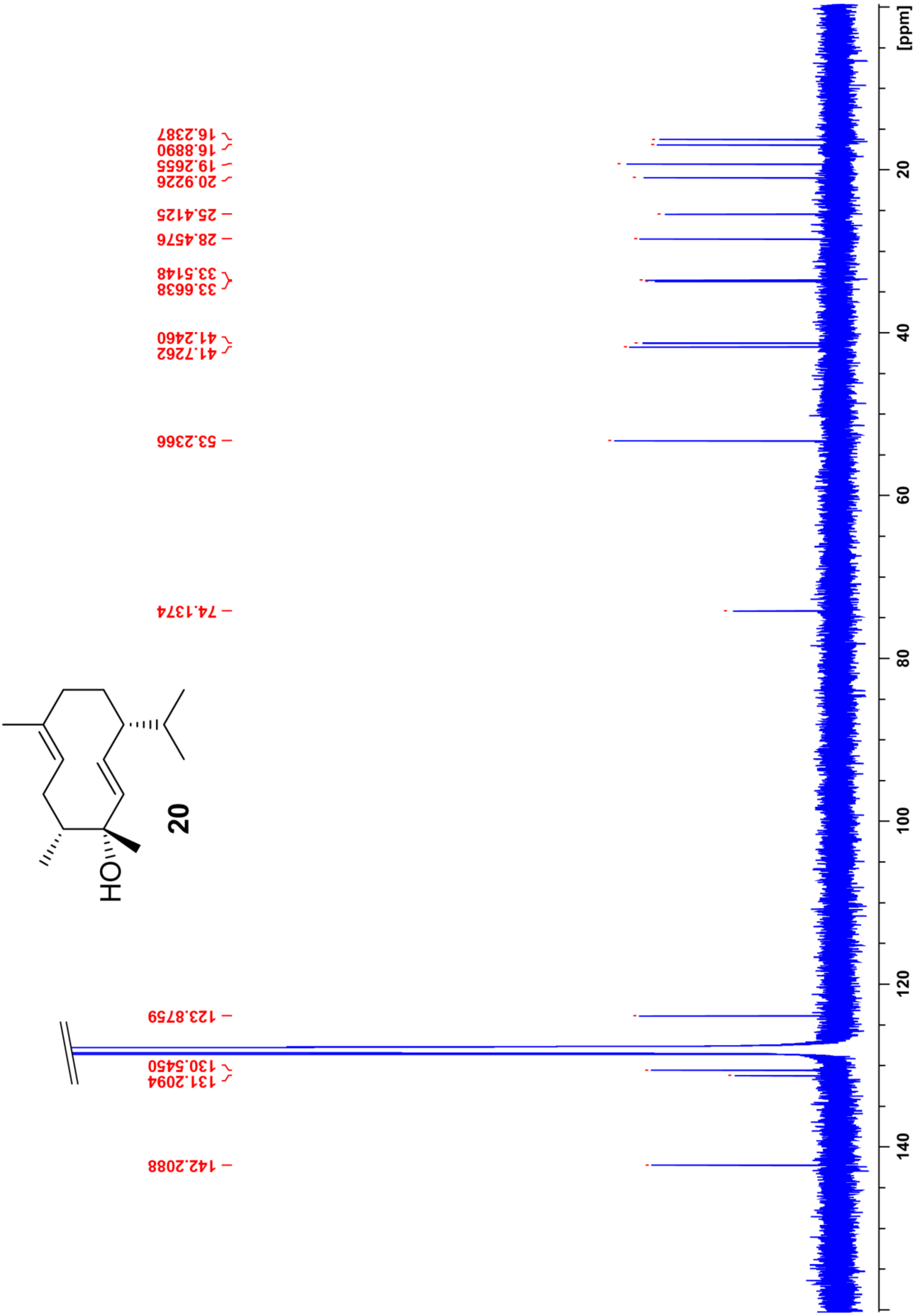

Figure S76. ^{13}C-NMR spectrum of **20** (175 MHz, C_6D_6).

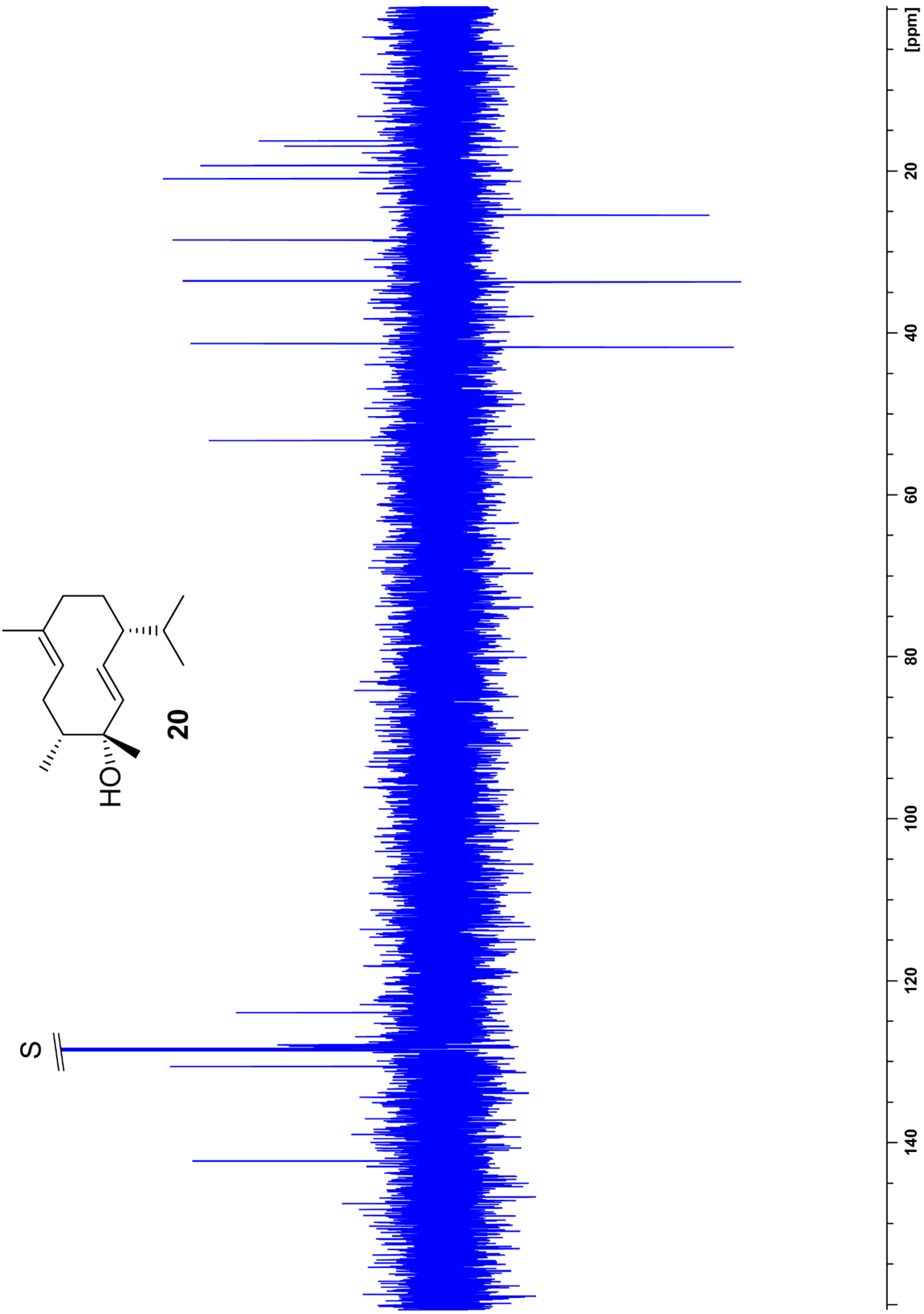

Figure S77. ^{13}C-DEPT spectrum of **20** (175 MHz, C_6D_6).

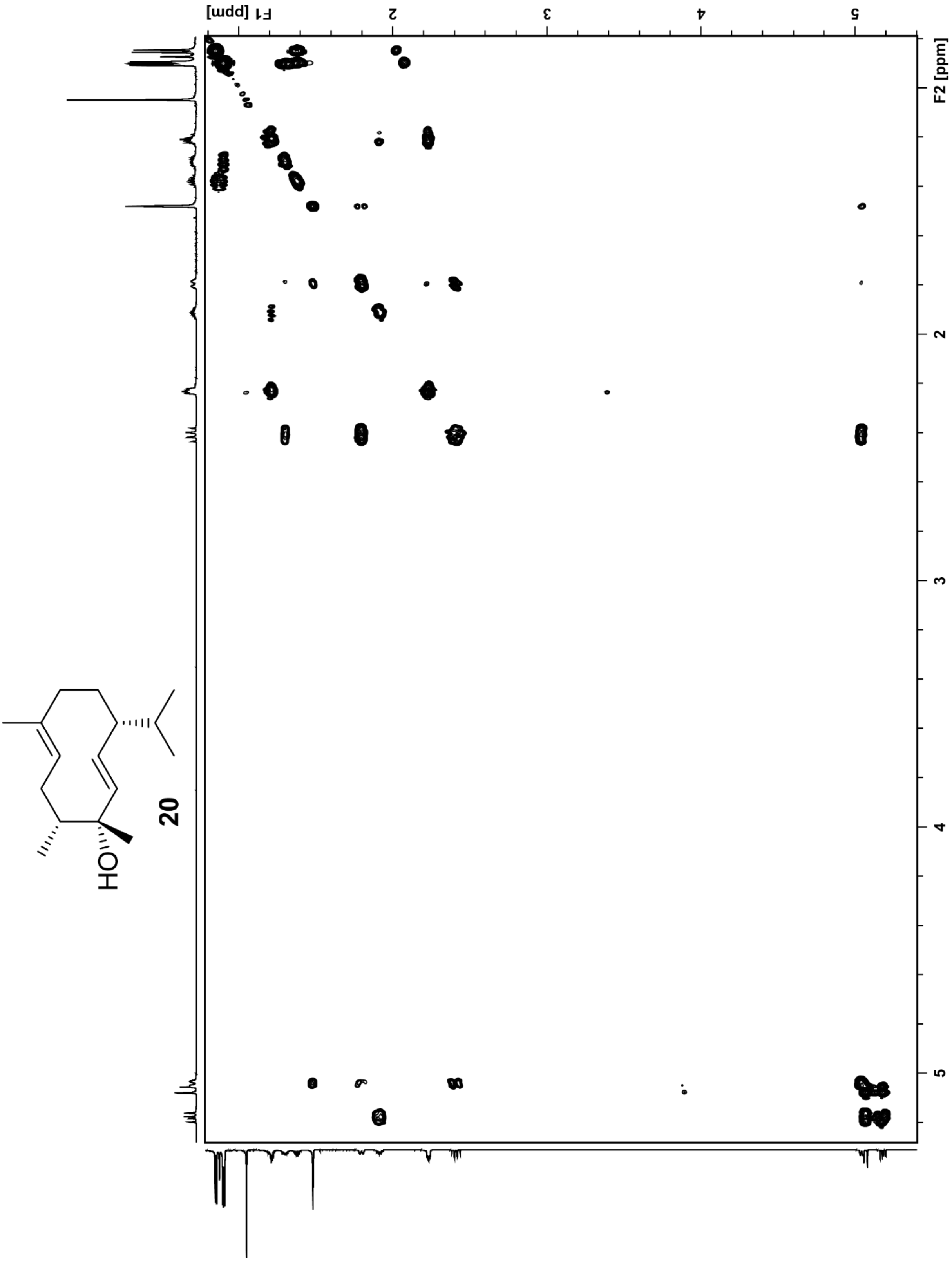

Figure S78. $^{1}H,^{1}H$-COSY spectrum of **20** (C_6D_6).

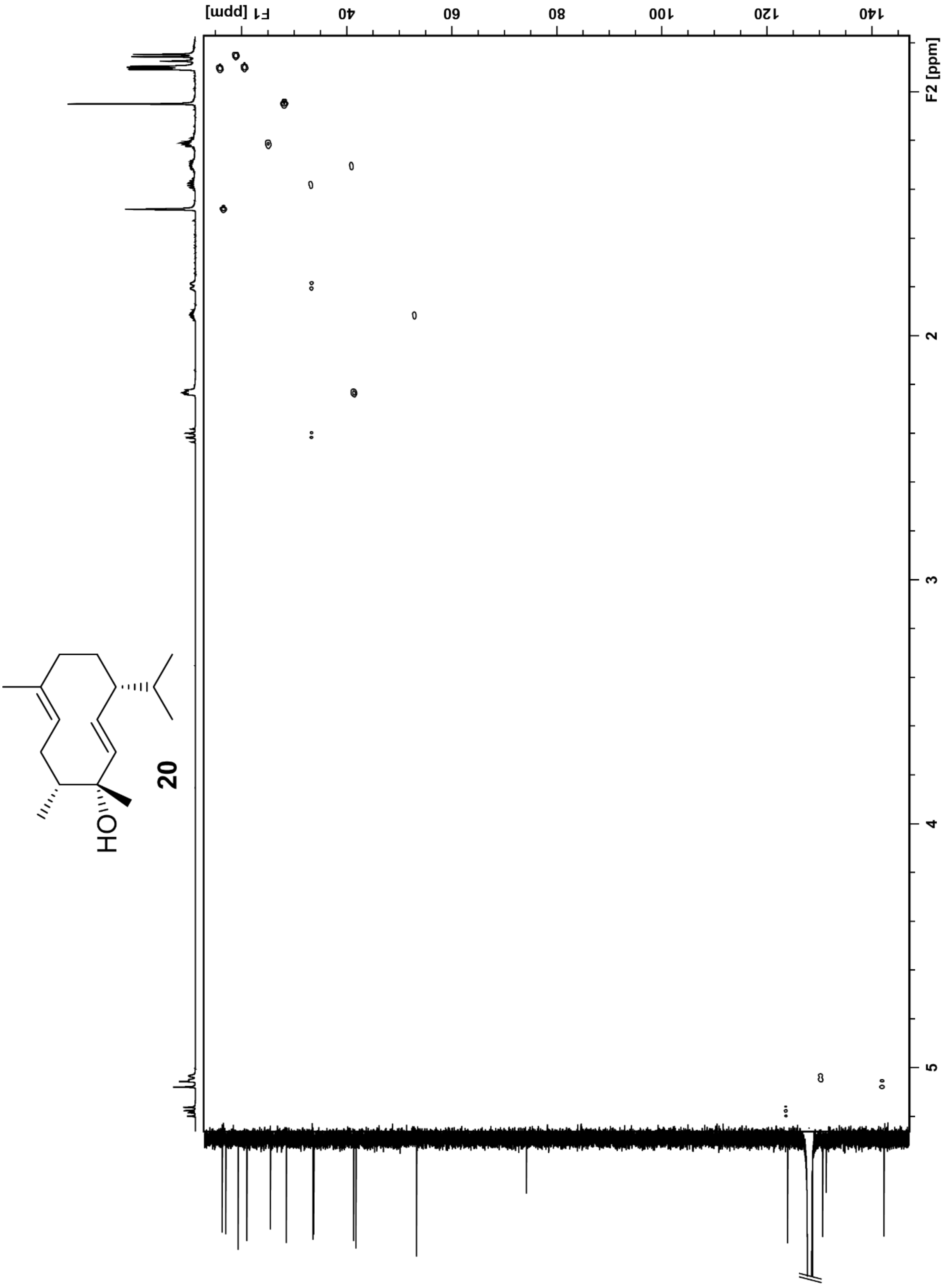

Figure S79. HSQC spectrum of **20** (C_6D_6).

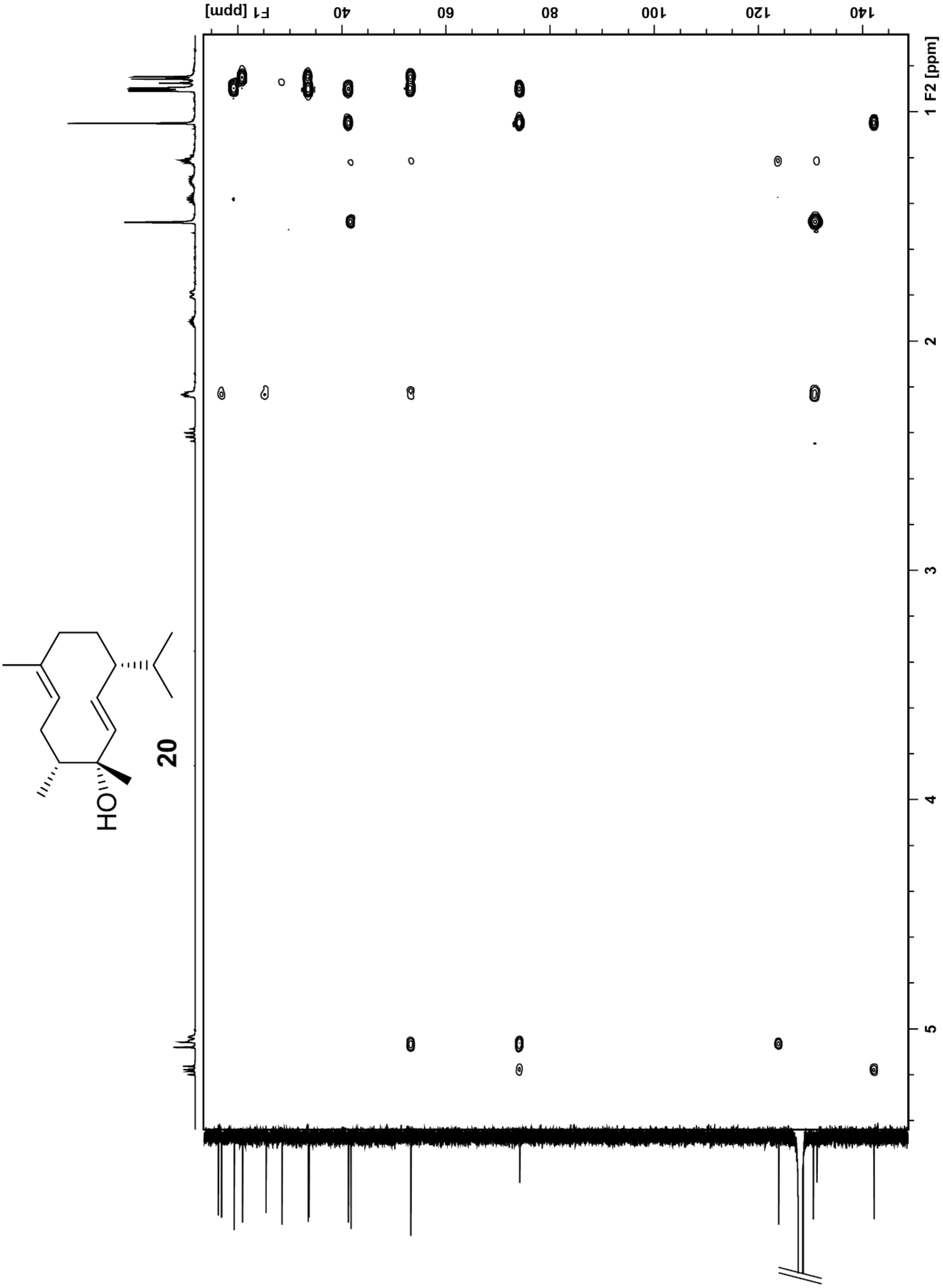

Figure S80. HMBC spectrum of **20** (C_6D_6).

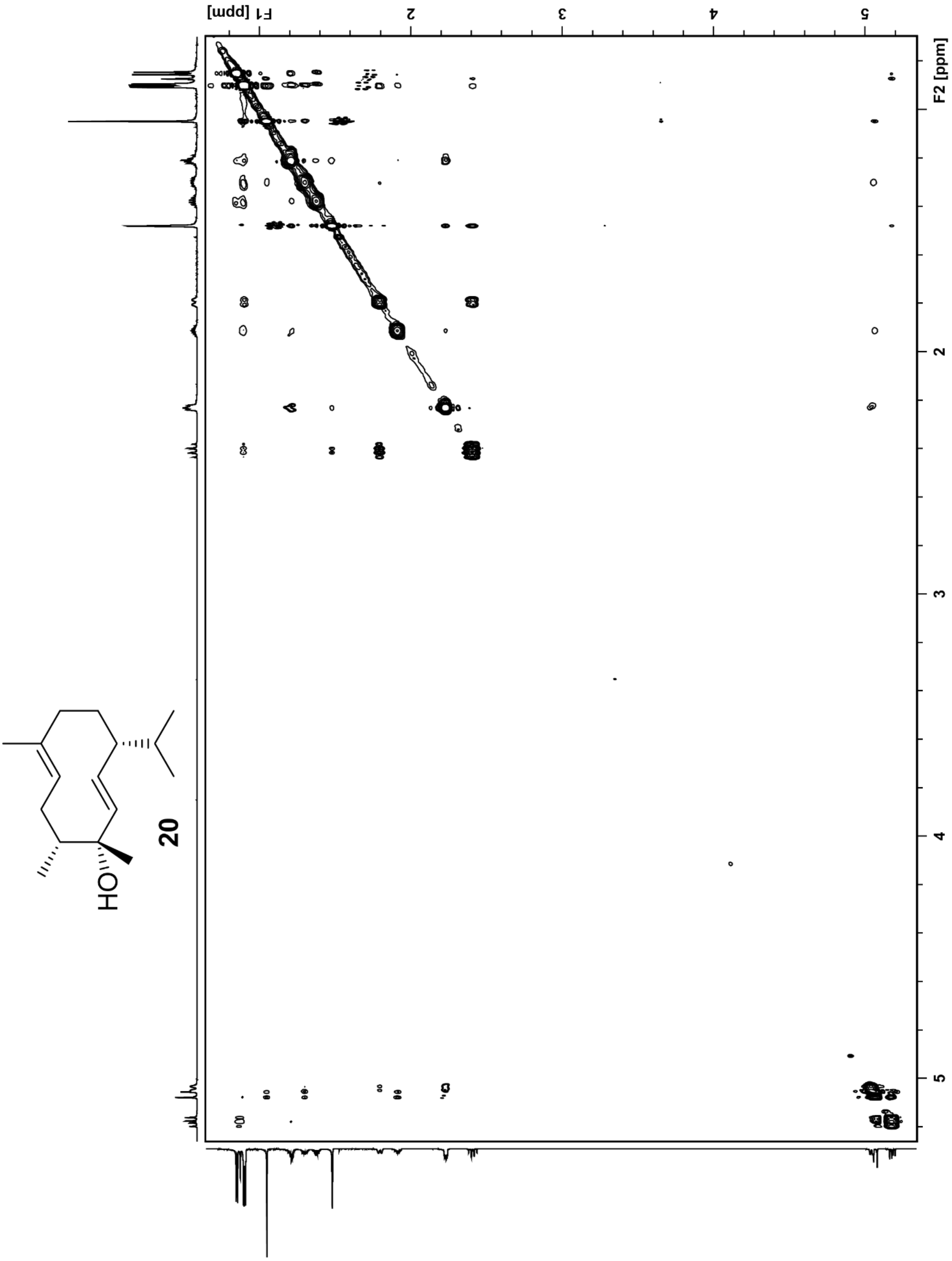

Figure S81. NOESY spectrum of **20** (C_6D_6).

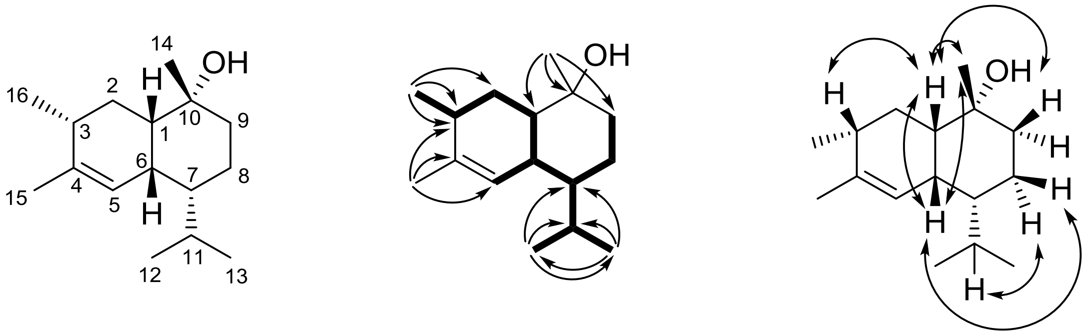

Table S11. NMR data of (3*R*)-3-methyl-1,6-*diepi*-T-muurolol (**21**) in C_6D_6 recorded at 298 K.

C[a]		^{13}C[b]	^{1}H[b]
1	CH	40.72	1.71 (m)
2	CH_2	26.32	1.75 (m, H_α)
			1.72 (m, H_β)
3	CH	34.34	2.08 (m)
4	C_q	138.38	–
5	CH	125.47	5.54 (m)
6	CH	37.37	1.92 (m)
7	CH	44.12	1.30 (m)
8	CH_2	21.83	1.33 (m, H_α)
			0.95 (m, H_β)
9	CH_2	35.69	1.48 (m, H_β)
			1.35 (m, H_α)
10	C_q	71.32	–
11	CH	26.81	2.00 (dsept, *J* = 3.1, 7.0)
12	CH_3	21.86	0.84 (d, *J* = 7.0)
13	CH_3	15.55	0.77 (d, *J* = 7.0)
14	CH_3	28.36	1.15 (s)
15	CH_3	22.44	1.65 (s)
16	CH_3	19.86	1.05 (d, *J* = 7.1)
–	OH	–	0.74 (br s)

[a] Carbon numbering as shown in the structure above the table (bold lines: ^{1}H,^{1}H-COSY correlations, single headed arrows: HMBC correlations, double headed arrows: NOESY correlations). [b] Chemical shifts δ in ppm, multiplicity: s = singlet, d = doublet, sept = septet, m = multiplet, br = broad, coupling constants *J* are given in Hertz.

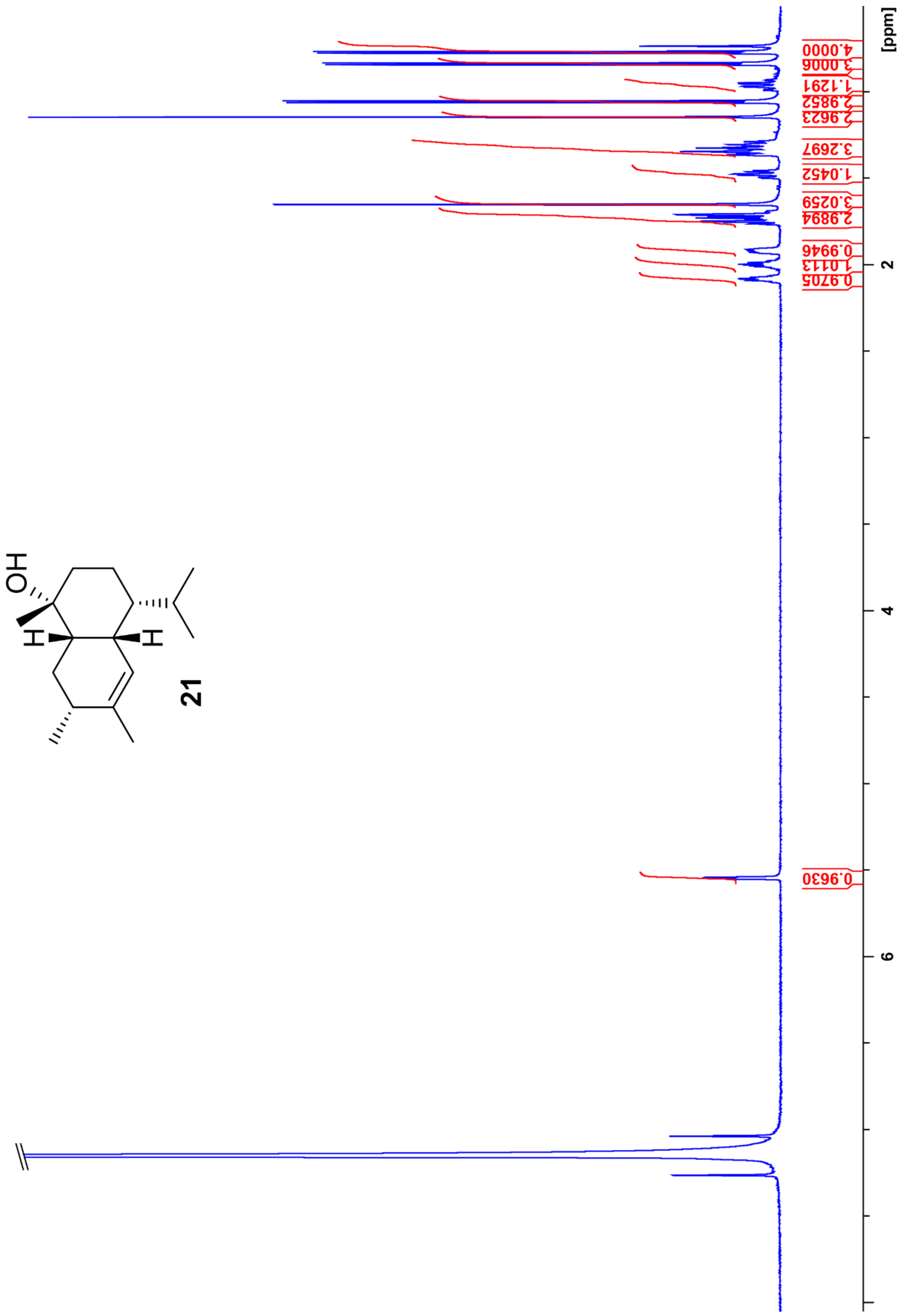

Figure S82. ^{1}H-NMR spectrum of **21** (700 MHz, C_6D_6).

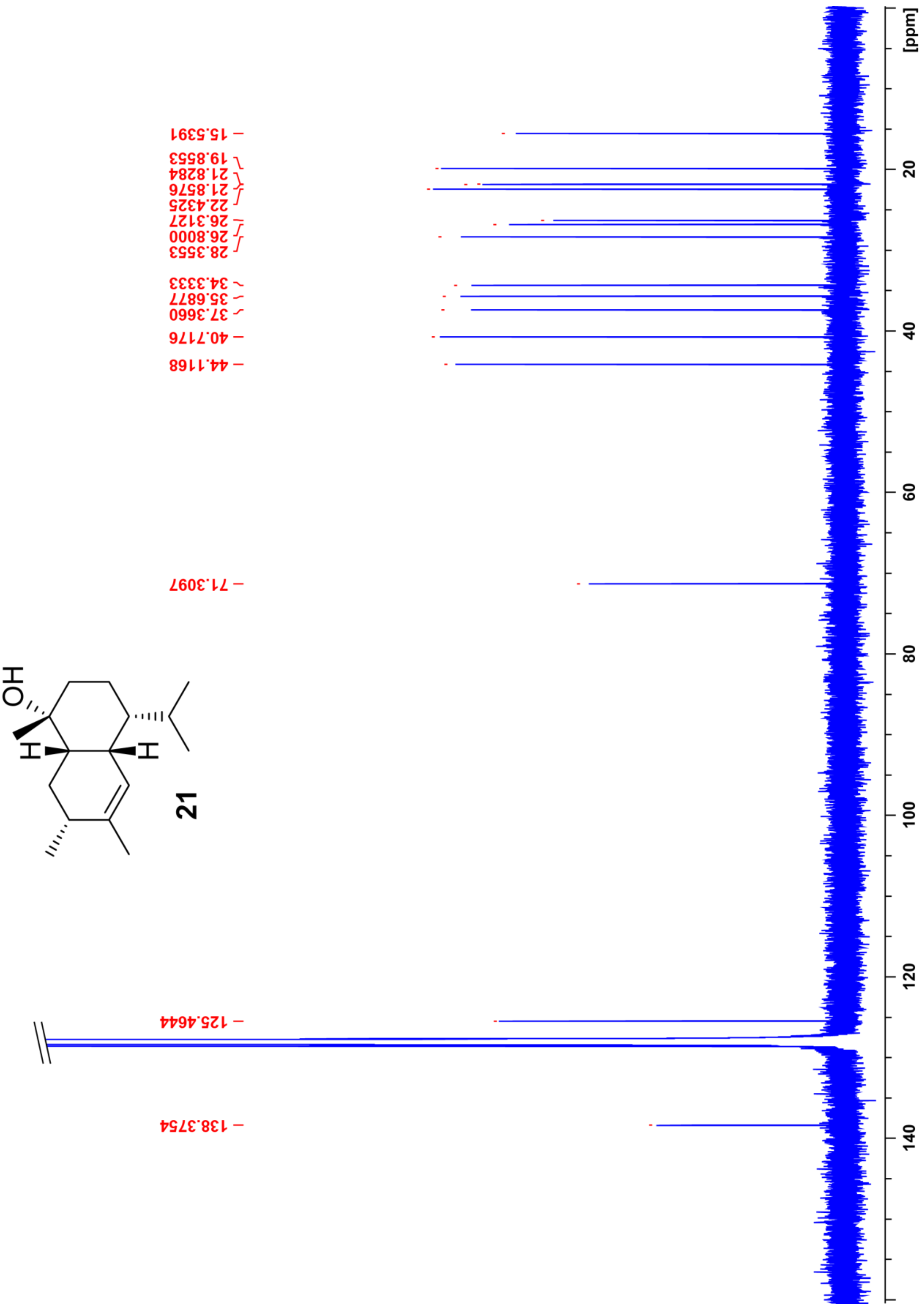

Figure S83. ^{13}C-NMR spectrum of **21** (175 MHz, C_6D_6).

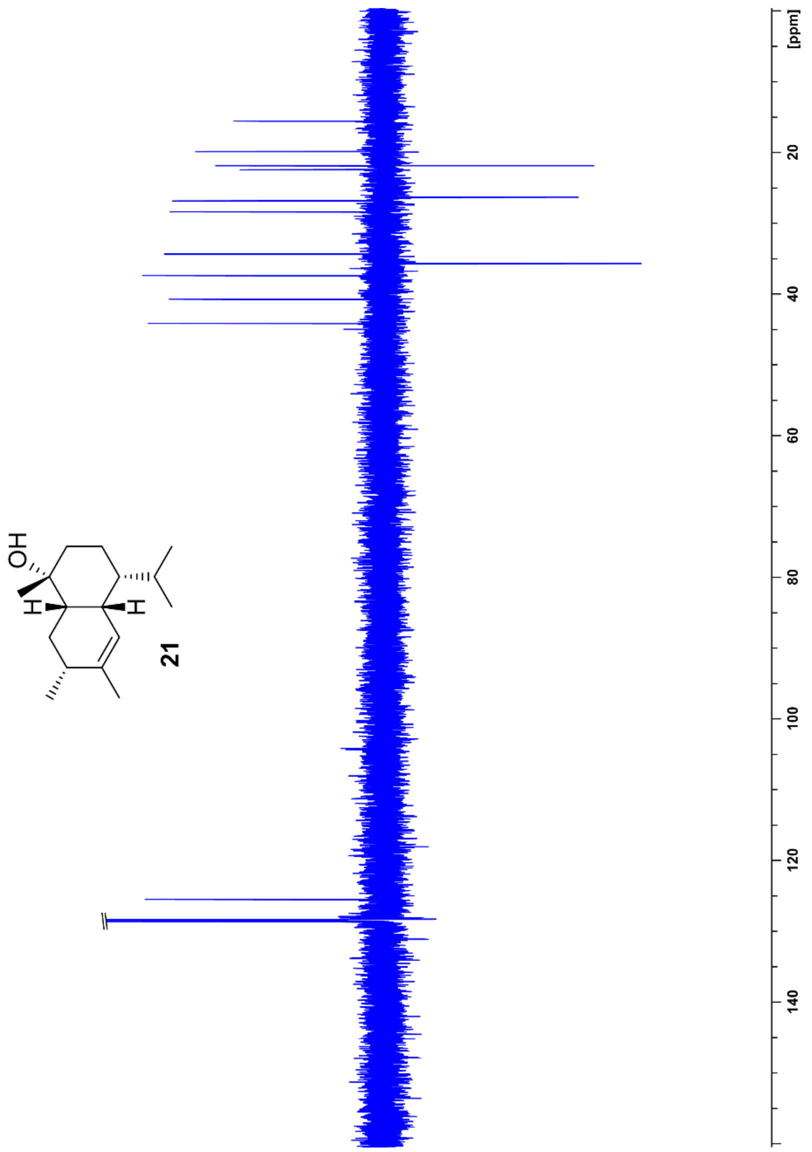

Figure S84. ^{13}C-DEPT spectrum of **21** (175 MHz, C_6D_6).

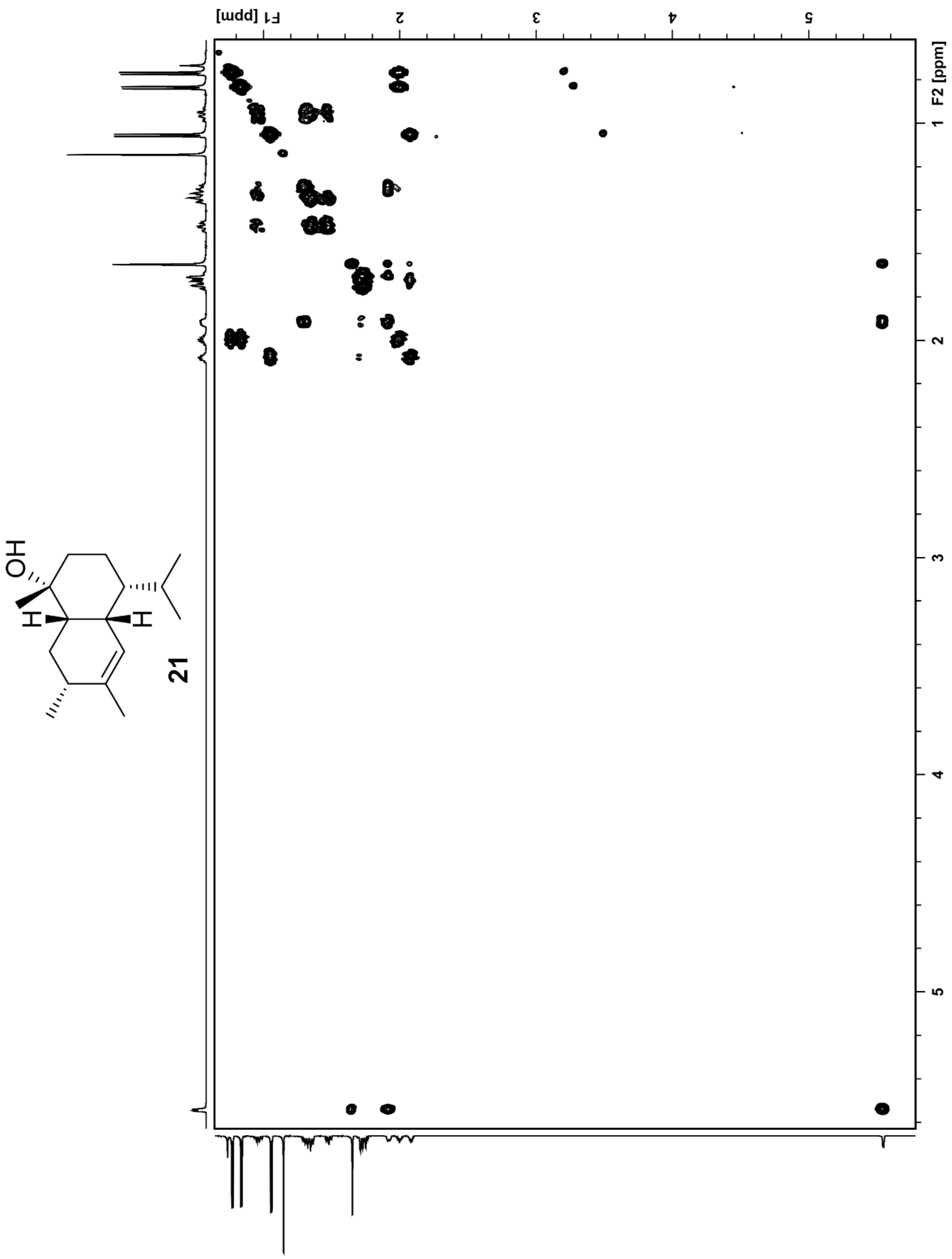

Figure S85. $^{1}H,^{1}H$-COSY spectrum of **21** (C_6D_6).

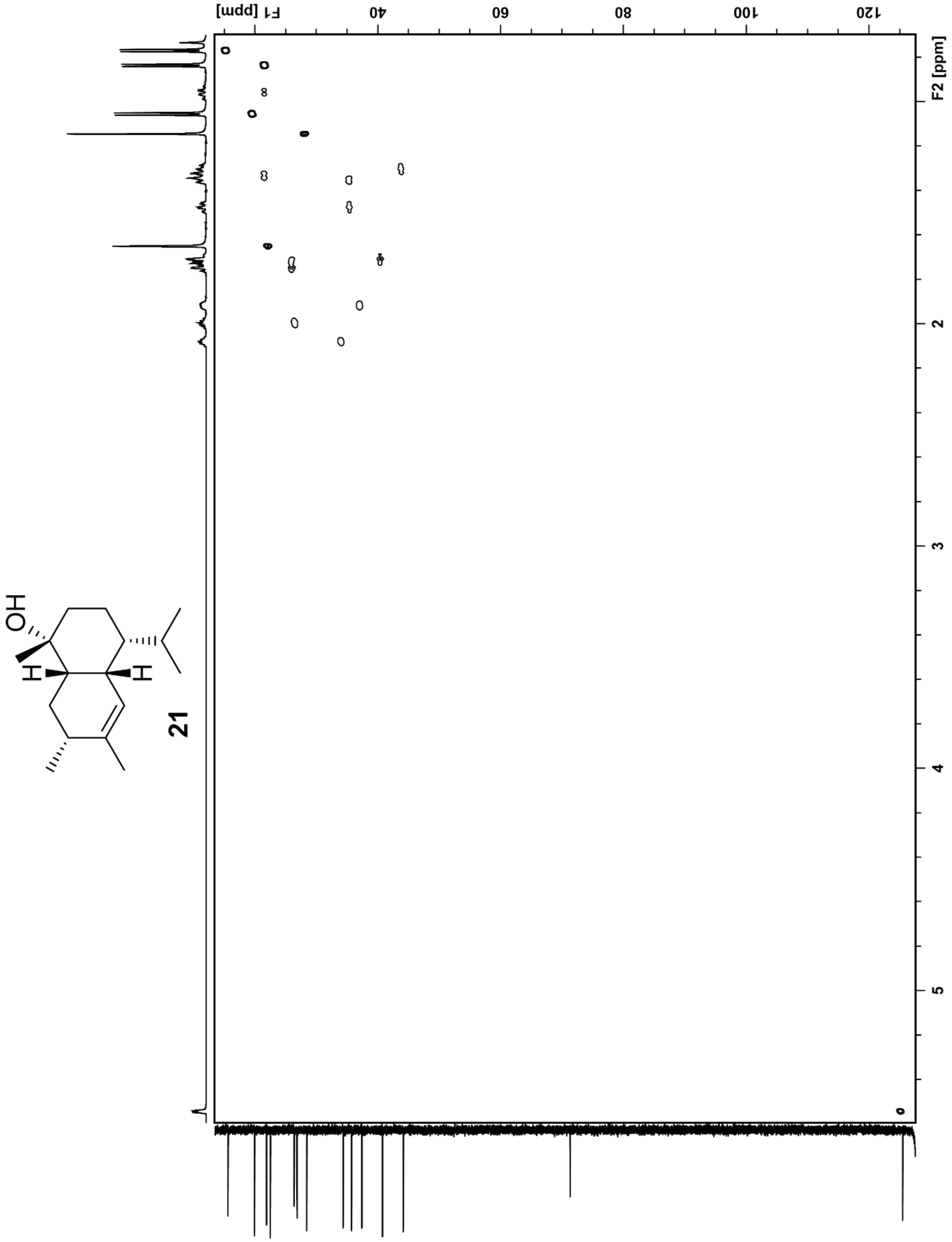

Figure S86. HSQC spectrum of **21** (C_6D_6).

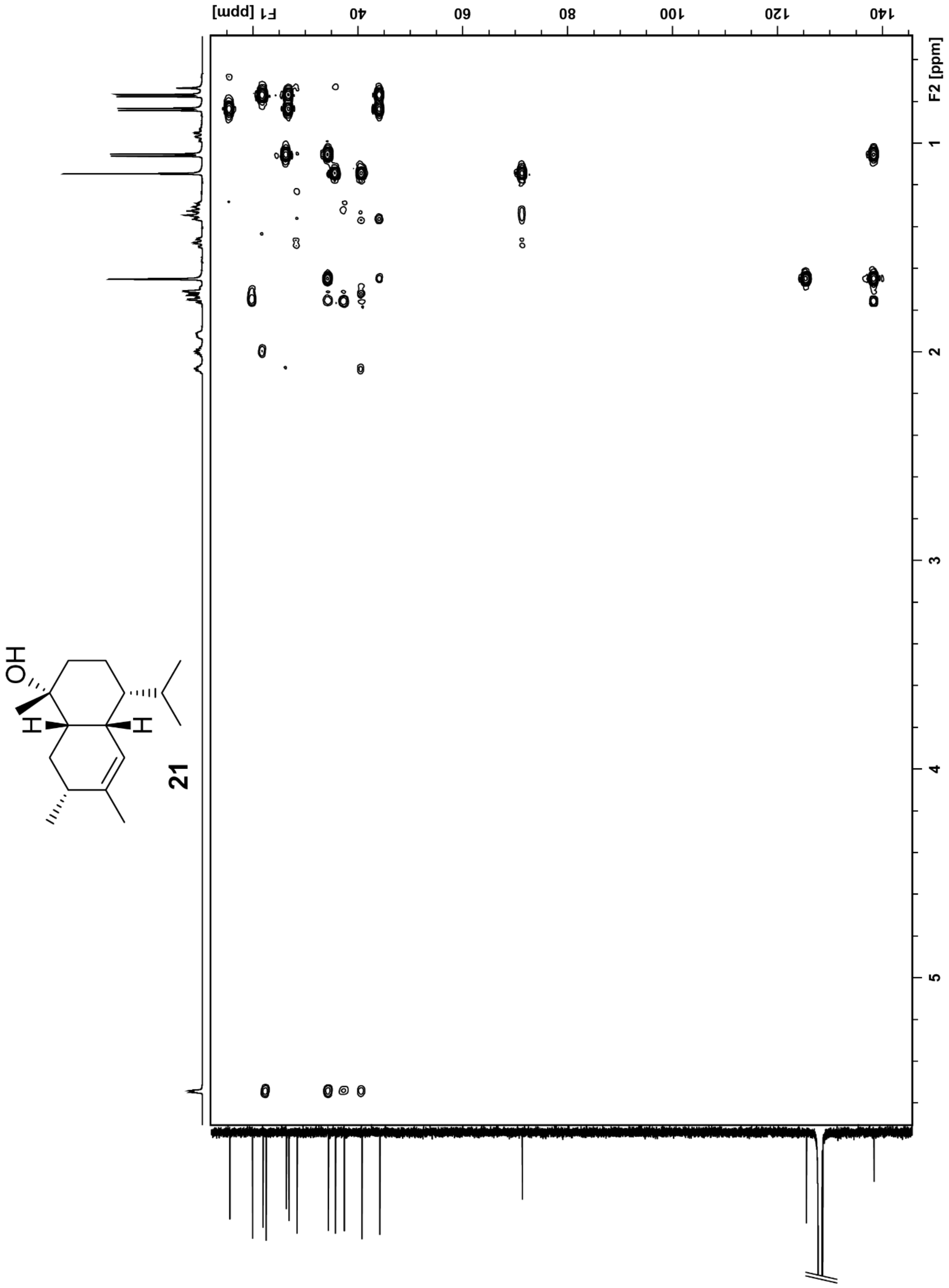

Figure S87. HMBC spectrum of **21** (C_6D_6).

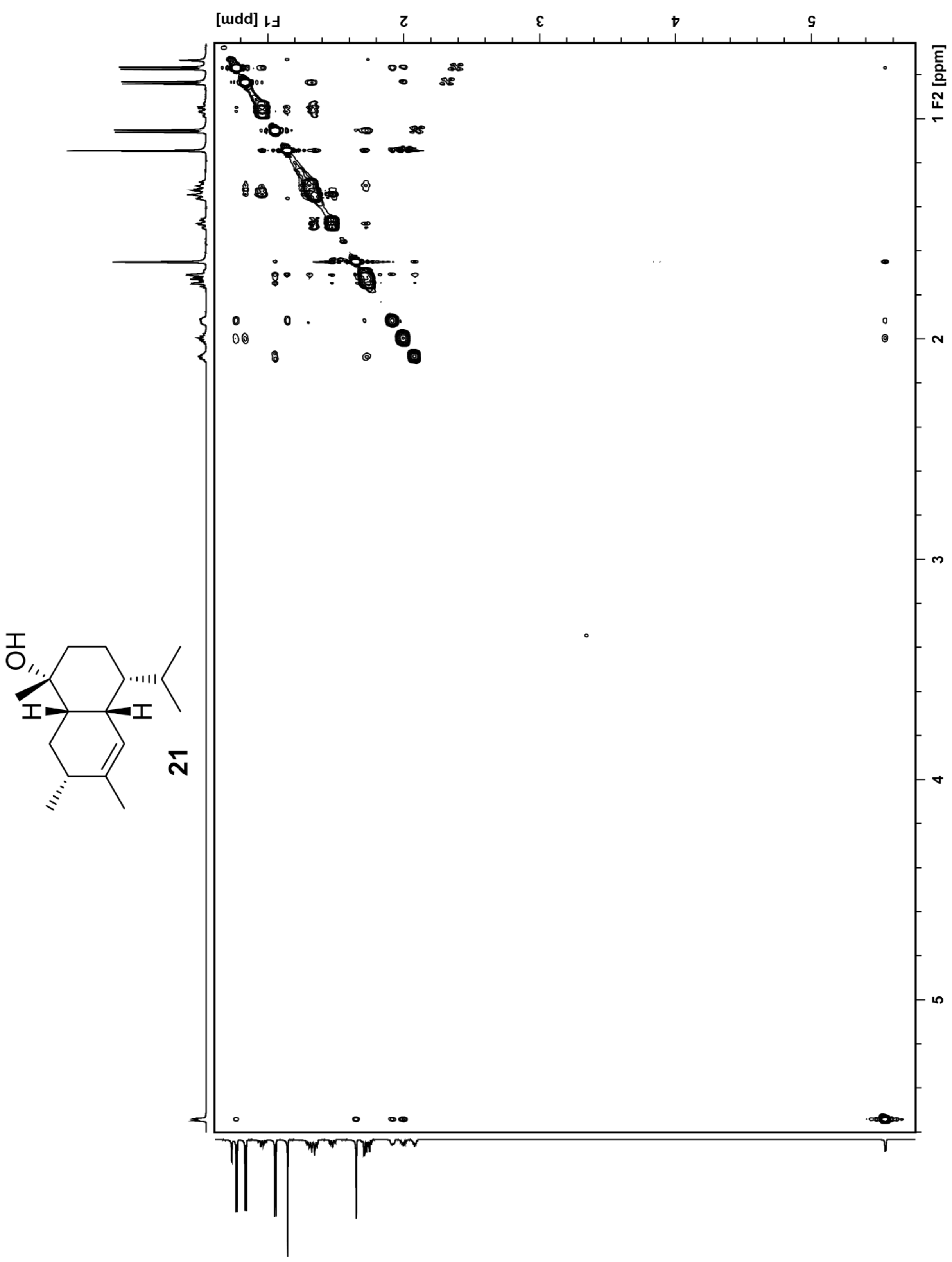

Figure S88. NOESY spectrum of **21** (C_6D_6).

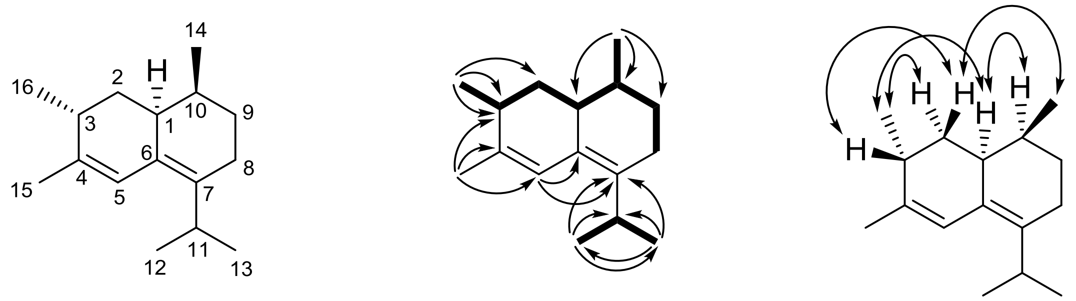

Table S12. NMR data of (1*S*,3*R*,10*S*)-3-methylzonarene (**22**) in C_6D_6 recorded at 298 K.

C[a]		^{13}C[b]	^{1}H[b]
1	CH	33.15	2.49 (m)
2	CH_2	35.05	1.65 (m, H_β)
			1.28 (ddd, *J* = 12.5, 3.5, 1.4, H_α)
3	CH	34.33	2.08 (m)
4	C_q	139.14	–
5	CH	120.88	6.39 (s)
6	C_q	126.66	–
7	C_q	133.89	–
8	CH_2	19.79	2.08 (m)
			1.94 (dd, *J* = 18.4, 5.8)
9	CH_2	30.23	1.67 (m)
			1.61 (m)
10	CH	30.95	–
11	CH	28.37	3.13 (sept, *J* = 6.9)
12	CH_3	21.29	1.00 (d, *J* = 7.0)
13	CH_3	20.90	0.97 (d, *J* = 6.9)
14	CH_3	12.71	0.86 (d, *J* = 7.0)
15	CH_3	23.01	1.75 (s)
16	CH_3	18.91	1.04 (d, *J* = 7.1)

[a] Carbon numbering as shown in the structure above the table (bold lines: ^{1}H,^{1}H-COSY correlations, single headed arrows: HMBC correlations, double headed arrows: NOESY correlations). [b] Chemical shifts δ in ppm, multiplicity: s = singlet, d = doublet, sept = septet, m = multiplet, coupling constants *J* are given in Hertz.

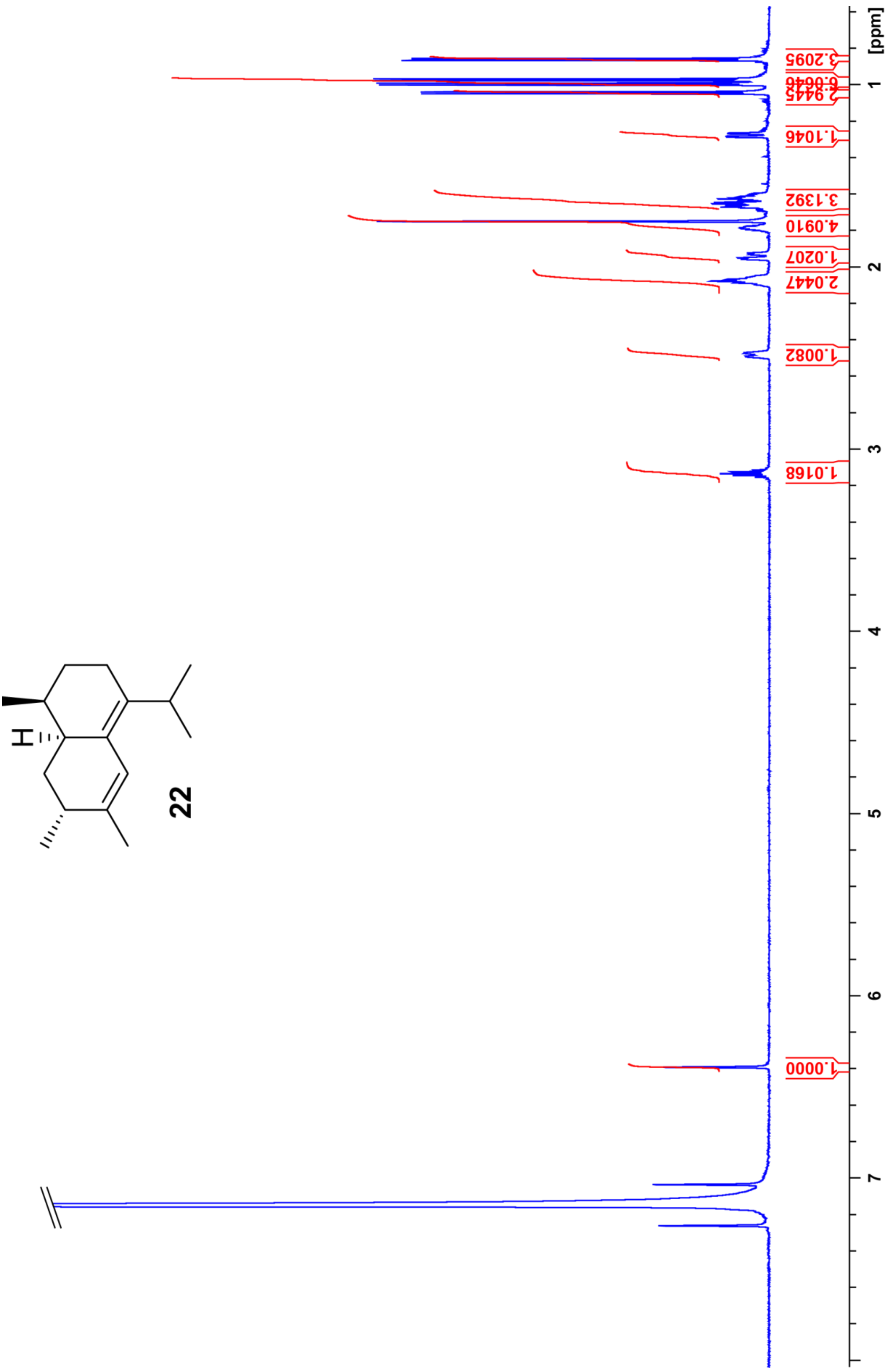

Figure S89. ^{1}H-NMR spectrum of **22** (700 MHz, C_6D_6).

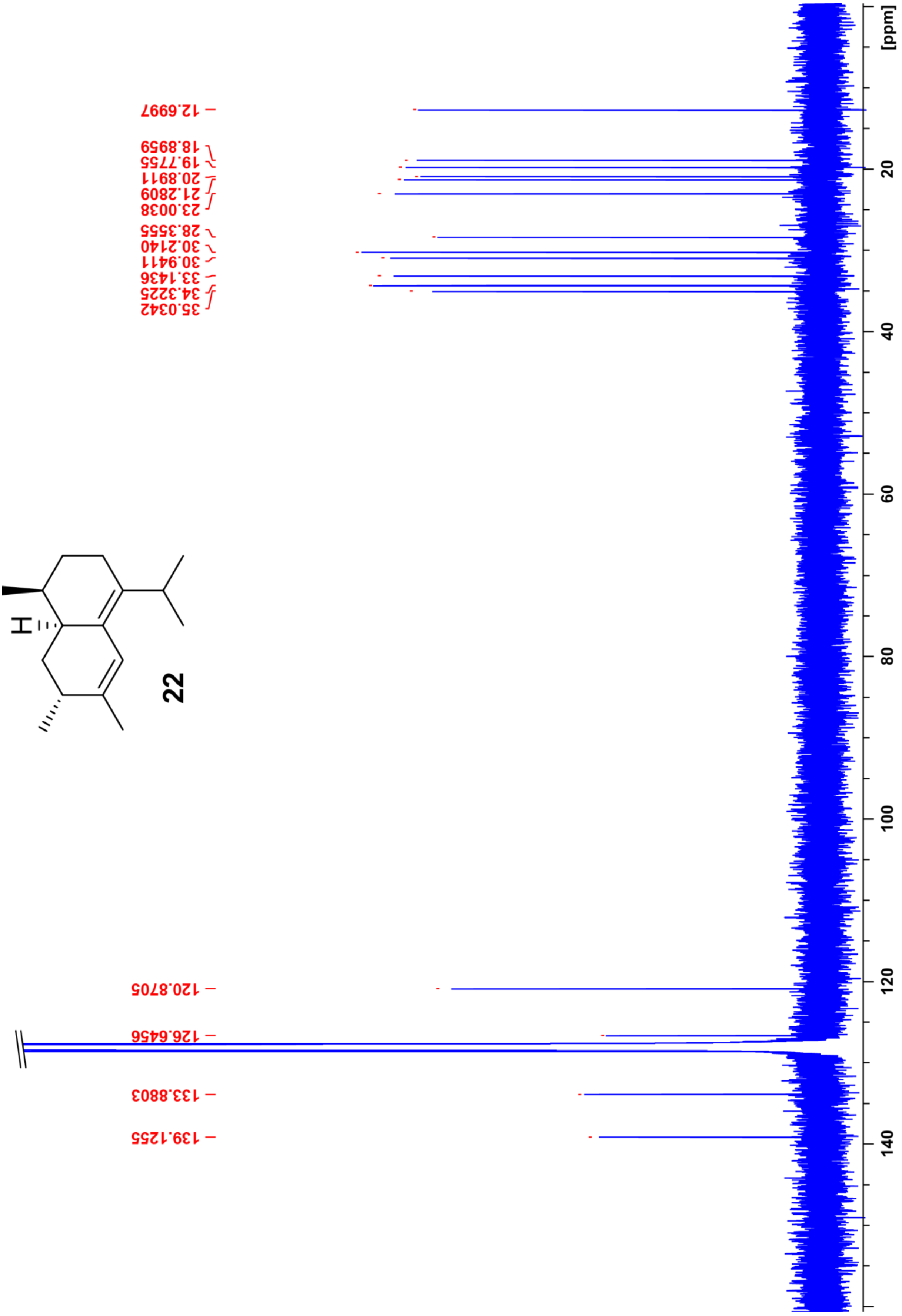

Figure S90. ^{13}C-NMR spectrum of **22** (175 MHz, C_6D_6).

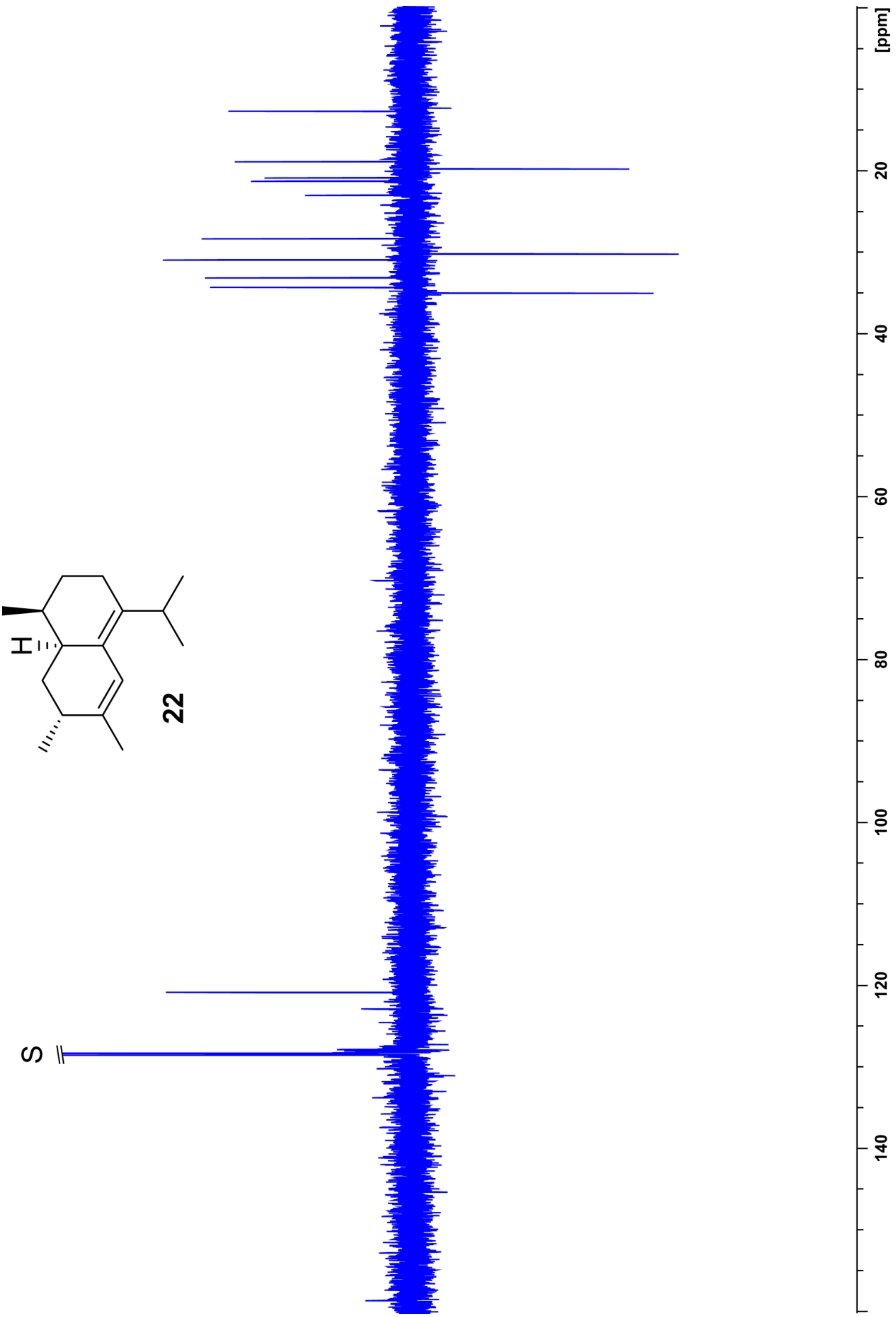

Figure S91. ^{13}C-DEPT spectrum of **22** (175 MHz, C_6D_6).

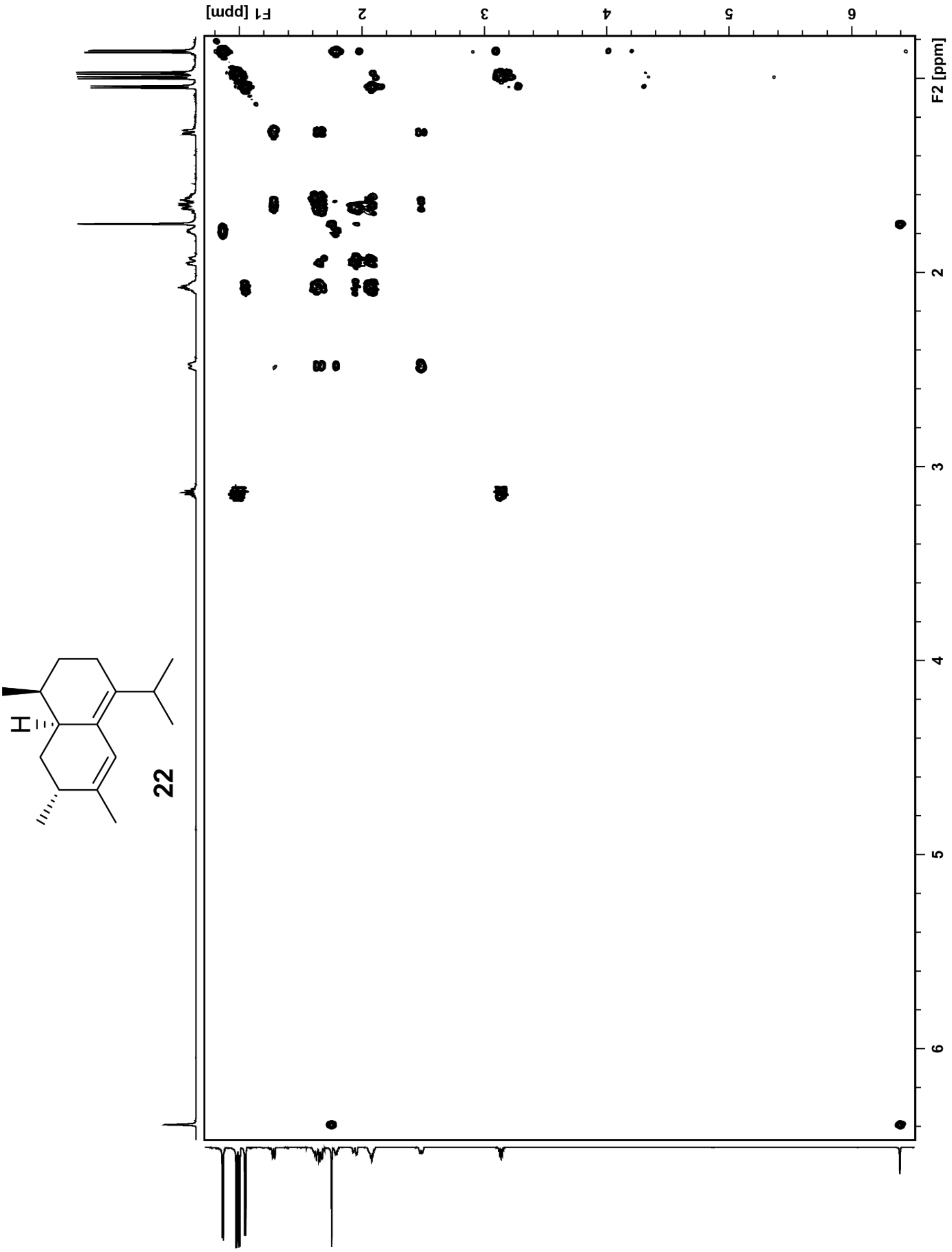

Figure S92. $^{1}H,^{1}H$-COSY spectrum of **22** (C_6D_6).

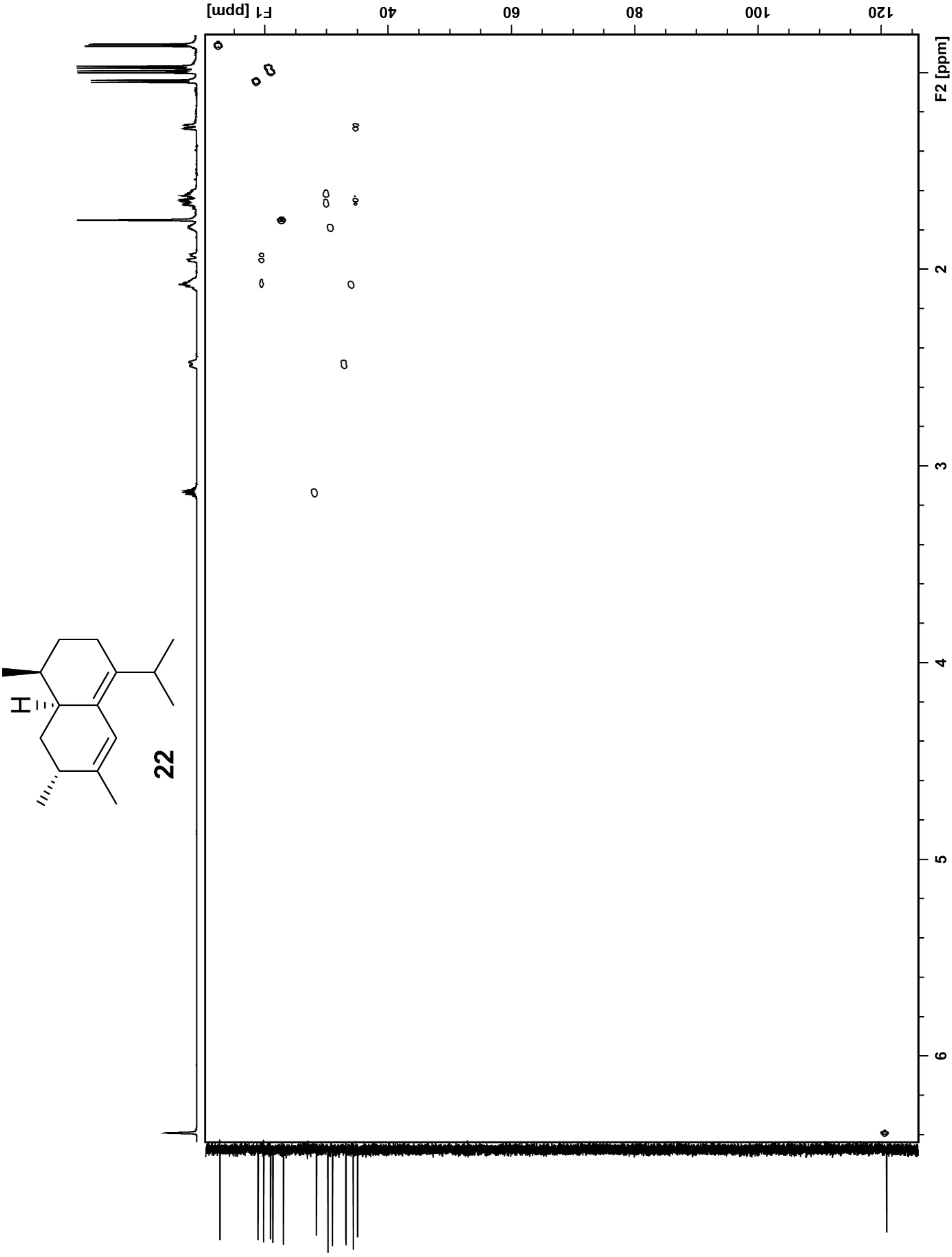

Figure S93. HSQC spectrum of **22** (C_6D_6).

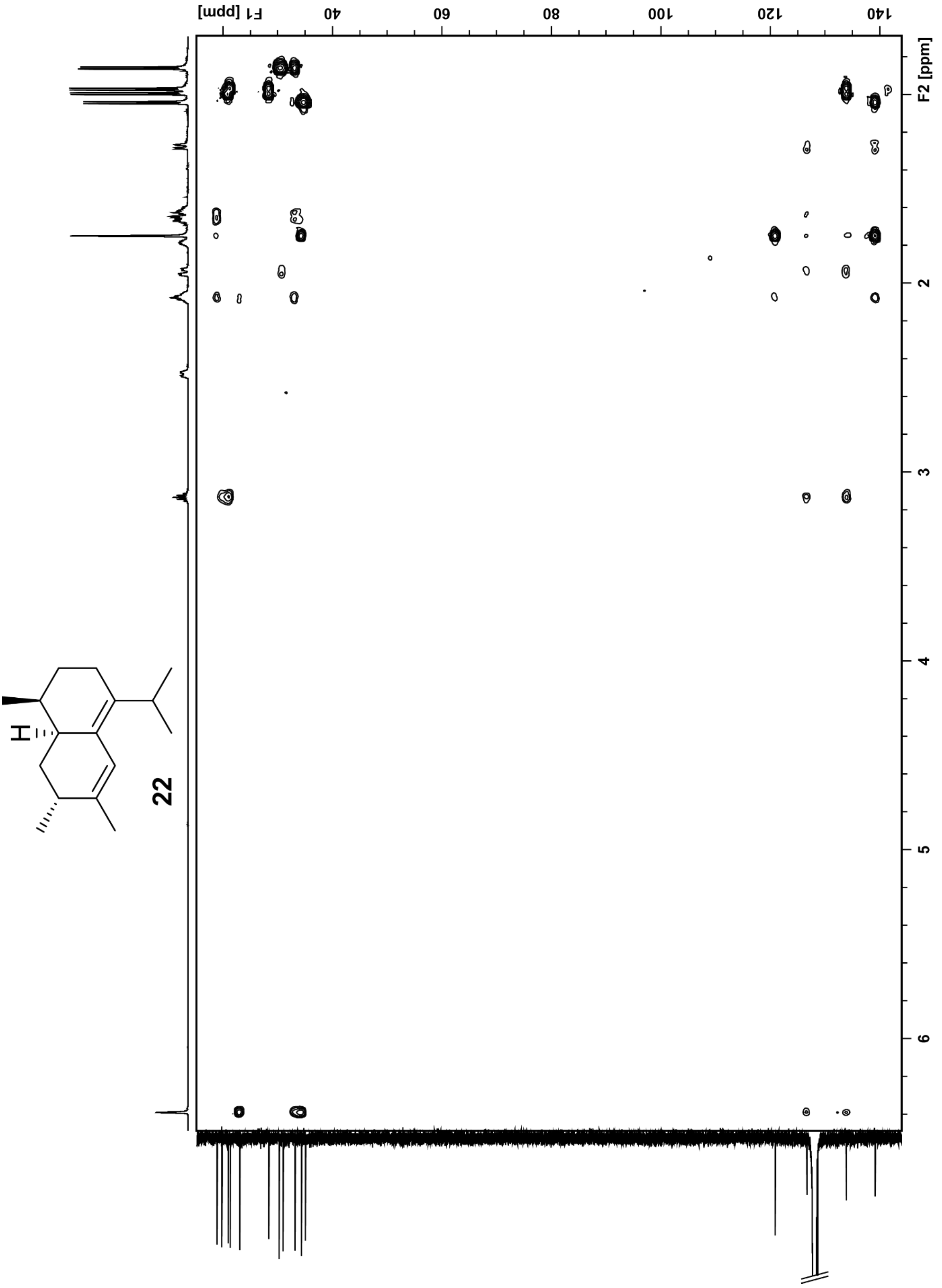

Figure S94. HMBC spectrum of **22** (C_6D_6).

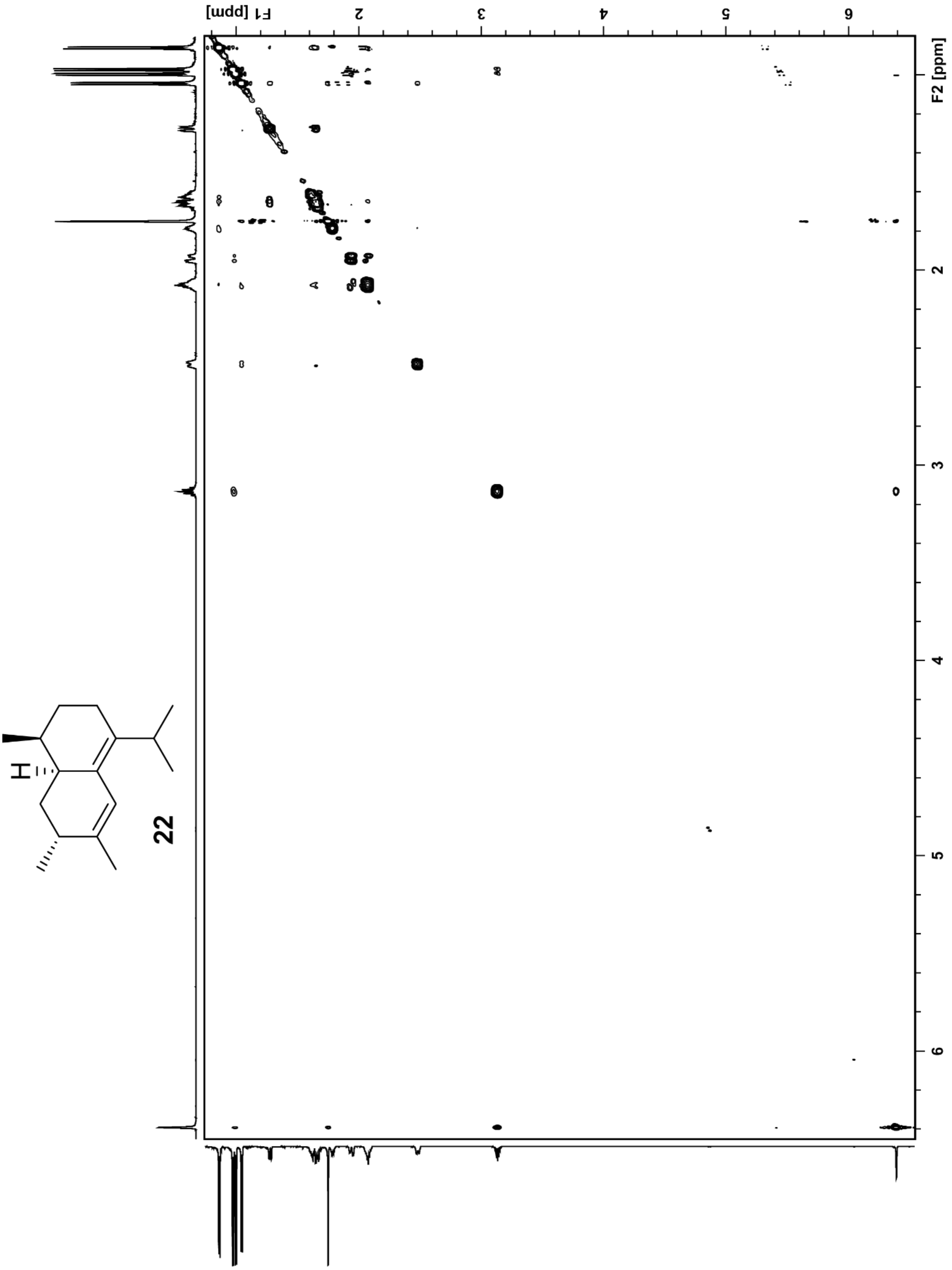

Figure S95. NOESY spectrum of **22** (C_6D_6).

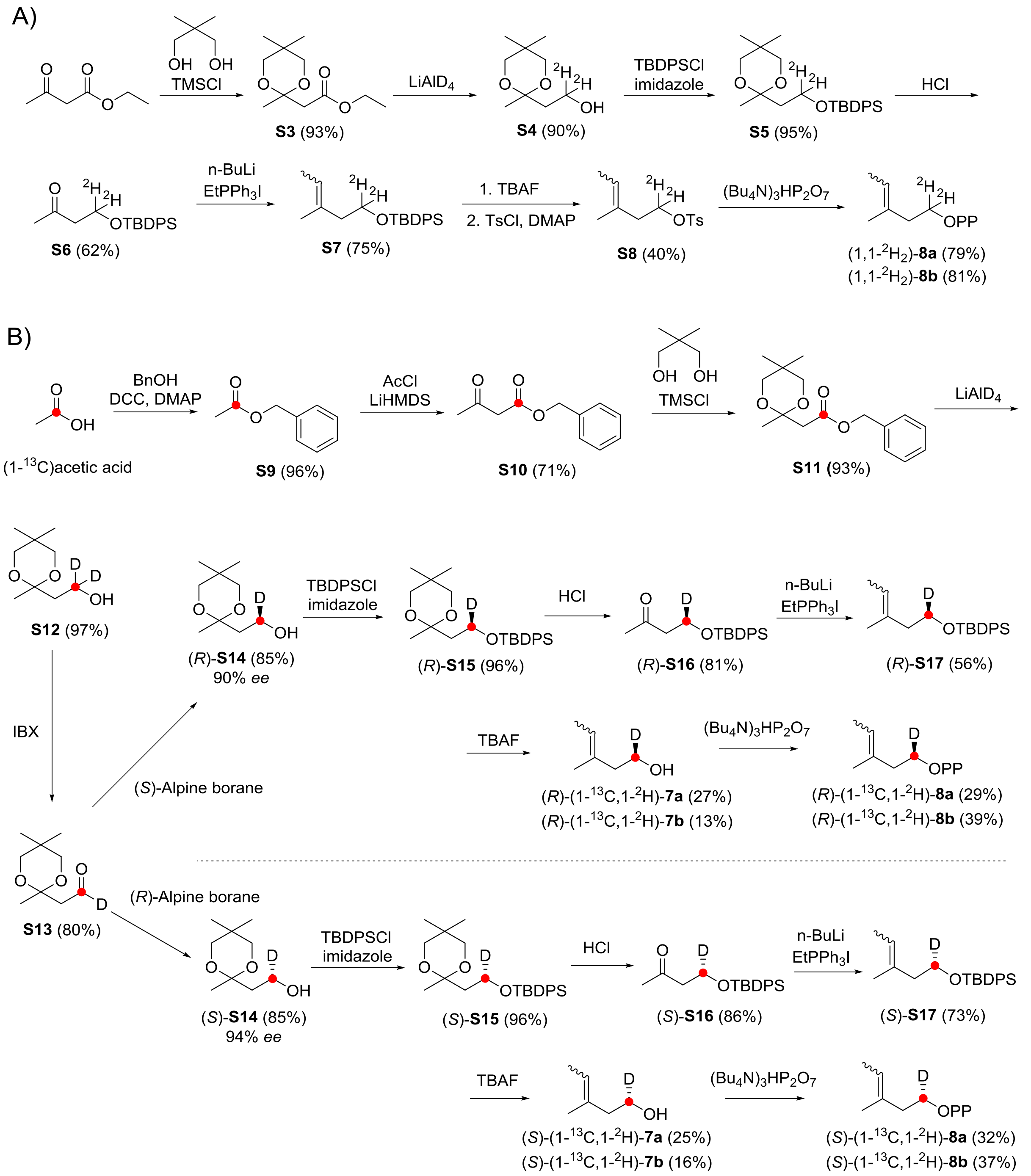

Scheme S2. Synthesis of isotopically labelled **8a** and **8b**. A) Synthesis of (1,1-$^2H_2$)-**8a** and (1,1-$^2H_2$)-**8b**, B) synthesis of (*R*)- and (*S*)-(1-^{13}C,1-^{2}H)-**8a**, and (*R*)- and (*S*)-(1-^{13}C,1-^{2}H)-**8b**.

Synthesis of (1,1-$^2H_2$)-8ab

Ethyl 2-(2,5,5-trimethyl-1,3-dioxan-2-yl)acetate (S3)

Ethyl acetoacetate (3.93 g, 30.2 mmol) and neopentyl glycol (6.9 g, 66.3 mmol, 2.2 eq.) were dissolved in DCM (50 mL). Then TMSCl was added dropwise. After the solution was heated to reflux for 12 h, the reaction was quenched by adding 5% aq. $NaHCO_3$, followed by extraction with Et_2O (3x 100 mL). The combined organic layers were dried with $MgSO_4$ and the solvent was removed under reduced pressure. The residue was purified by silica gel chromatography [cyclohexane/EA (7/1), R_f = 0.28] to afford **S3** (6.1 g, 28.20 mmol, 93%) as a colourless oil.

Ethyl 2-(2,5,5-trimethyl-1,3-dioxan-2-yl)acetate (S3). ^{1}H NMR (500 MHz, $CDCl_3$): δ = 4.16 (q, $^{3}J_{H,H}$ = 7.1 Hz, 2H, CH_2), 3.58 – 3.48 (m, 4H, 2x CH_2), 2.78 (s, 2H, CH_2), 1.54 (s, 3H, CH_3), 1.26 (d, $^{3}J_{H,H}$= 7.1 Hz, 3H, CH_3), 0.98 (s, 3H, CH_3), 0.94 (s, 3H, CH_3) ppm. ^{13}C NMR (126 MHz, $CDCl_3$): δ = 169.68 (Cq), 97.49 (Cq), 70.78 (2x CH_2), 60.69 (CH_2), 41.77 (CH_2), 30.03 (C_q), 22.96 (CH_3), 22.75 (CH_3), 22.64 (CH_3), 14.33 (CH_3) ppm. GC (HP-5MS): *I* = 1345. MS (EI, 70 eV): *m*/*z* (%) = 201 (23), 129 (100), 115 (18), 103 (13), 85 (32), 69 (54), 56 (25), 43 (61).

(1,1-^{2}H$_2$)-2-(2,5,5-Trimethyl-1,3-dioxan-2-yl)ethan-1-ol (S4)

$LiAlD_4$ (1.19 g, 28.4 mmol, 1.0 eq.) was suspended in THF (200 mL). After the mixture was cooled to 0 °C, **S3** (6.1 g, 28.2 mmol) was added dropwise, followed by stirring at 0 °C for 3 h. The reaction was quenched by addition of H_2O (8 mL) at 0 °C and then allowed to warm to room temperature and dried with $MgSO_4$. The mixture was filtrated and rinsed with THF, the filtrate was then evaporated under reduced pressure to afford the crude product which was purified by silica gel chromatography [cyclohexane/EA (1/1), R_f = 0.40] to afford **S4** (4.46 g, 25.3 mmol, 90%) as a colourless oil.

(1,1-^{2}H$_2$)-2-(2,5,5-Trimethyl-1,3-dioxan-2-yl)ethan-1-ol (S4). ^{1}H NMR (500 MHz, C_6D_6): δ = 3.31 – 3.08 (m, 4H, 2x CH_2), 2.75 (s, 1H, OH), 1.85 (s, 2H, CH_2), 1.16 (s, 3H, CH_3), 0.96 (s, 3H, CH_3), 0.39 (s, 3H, CH_3) ppm. ^{13}C NMR (126 MHz, C_6D_6): δ = 100.10 (C_q), 70.23 (2x CH_2), 42.32 (CH_2), 29.74 (C_q), 22.81 (CH_3), 22.03 (CH_3), 19.07 (CH_3) ppm. GC (HP-5MS): *I* = 1239. MS (EI, 70 eV): *m*/*z* (%) = 161 (82), 143 (4), 129 (100), 91 (38), 75 (43), 69 (66), 55 (15), 43 (75).

***tert*-Butyldiphenyl((1,1-^{2}H$_2$)-2-(2,5,5-trimethyl-1,3-dioxan-2-yl)ethoxy)silane (S5)**

To a solution of **S4** (4.46 g, 25.3 mmol) and imidazole (1.90 g, 27.9 mmol, 1.1 eq.) in DCM (150 mL) was added TBDPSCl (7.6 g, 27.7 mmol, 1.1 eq.). The reaction mixture was stirred over night at room temperature and then quenched by the addition of H_2O (150 mL), followed by extraction with DCM (3x 100 mL). The combined organic layers were washed with brine and dried with $MgSO_4$. The solvent was evaporated under reduced pressure. The residue was purified by silica gel chromatography [cyclohexane/EA (20/1), R_f = 0.25] to afford **S5** (9.93 g, 24.0 mmol, 95%) as a colourless oil.

***tert*-Butyldiphenyl((1,1-^{2}H$_2$)-2-(2,5,5-trimethyl-1,3-dioxan-2-yl)ethoxy)silane (S5).** ^{1}H NMR (499 MHz, C_6D_6): δ = 7.83 – 7.78 (m, 4H, 4x CH), 7.23 – 7.19 (m, 6H, 6x CH), 3.26 – 3.15 (m, 4H, 2x CH_2), 2.22 (s, 2H, CH_2), 1.33 (s, 3H, CH_3), 1.19 (s, 9H, 3x CH_3), 0.72 (s, 3H, CH_3), 0.64 (s, 3H, CH_3) ppm. ^{13}C NMR (126 MHz, C_6D_6): δ = 136.06 (4x CH) , 134.48 (2x C_q), 129.92 (2x CH), 128.09 (4x CH), 98.24 (C_q), 70.20 (2x CH_2), 40.39 (CH_2), 29.79 (C_q), 27.18 (3x CH_3), 22.69 (CH_3), 22.53 (CH_3), 21.82 (CH_3), 19.46 (C_q) ppm. GC (HP-5MS): *I* = 2585. MS (EI, 70 eV): *m*/*z* (%) = 399 (1), 283 (2), 271 (100), 253 (4), 239 (37), 227 (2), 221 (3), 213 (6), 199 (49), 193 (76), 181 (8), 175 (4), 166 (8), 161 (11), 139 (19), 129 (38), 121 (5), 115 (3), 105 (5), 93 (4), 77 (11), 69 (17), 57 (6), 43 (21).

(4,4-^{2}H$_2$)-4-((*tert*-Butyldiphenylsilyl)oxy)butan-2-one (S6)

To a solution of **S5** (9.93 g, 24.0 mmol) in methanol (150 mL) was added aq. HCl (1 M, 14.3 mL, 14.3 mmol, 0.48 eq.) dropwise. After the solution was stirred at room temperature for 30 min, the reaction was quenched by adding aq. $NaHCO_3$ (5%, 250 mL), followed by extraction with Et_2O (3x 100 mL). The combined organic layers were dried with $MgSO_4$ and concentrated under reduced pressure. Purification by silica gel chromatography [cyclohexane/EA (10/1), R_f = 0.35] afforded **S6** (4.9 g, 14.92 mmol, 62%) as a colourless oil.

(4,4-^{2}H$_2$)-4-((*tert*-Butyldiphenylsilyl)oxy)butan-2-one (S6). ^{1}H NMR (500 MHz, $CDCl_3$): δ = 7.69 – 7.61 (m, 4H, 4x CH), 7.45 – 7.35 (m, 6H, 6x CH), 2.63 (s, 2H, CH_2), 2.19 (s, 3H, CH_3), 1.04 (s, 9H, 3x CH_3) ppm. ^{13}C NMR (126 MHz, $CDCl_3$): δ = 208.05 (C_q), 135.68 (4x CH), 133.59 (2x C_q), 129.85 (2x CH), 127.85 (4x CH), 46.32 (CH_2), 30.87 (CH_3), 26.92 (3x CH_3), 19.29 (C_q)

ppm. GC (HP-5MS): *I* = 2206. MS (EI, 70 eV): *m*/*z* (%) = 271 (99), 239 (71), 221 (6), 213 (10), 199 (95), 193 (100), 181 (19), 161 (22), 139 (25), 121 (11), 115 (7), 105 (10), 93 (6), 77 (26), 57 (7), 51 (3), 45 (11).

Ethyltriphenylphosphonium iodide (ETPPI)
To a PhMe solution (200 mL) of Ph_3P (9.0 g, 34.3 mmol) was added CH_3CH_2I (6.86 g, 44.0 mmol, 1.28 eq.), and the mixture was heated to reflux for 5 h. Then the mixture was allowed to cool to room temperature, followed by filtration to remove the solvent. The solid residue was washed several times with PhMe, and dried under high vacuum at 50 °C to afford the desired product (10.7 g, 25.58 mmol, 75%) as a colourless powder.
ETPPI. ^{1}H NMR (500 MHz, $CDCl_3$): δ = 7.81 – 7.75 (m, 8H, 8x CH), 7.72 – 7.67 (m, 7H, 7x CH), 3.68 (dq, $^2J_{P,H}$ = 12.3 Hz, $^3J_{H,H}$ = 7.4 Hz, 2H, CH_2), 1.37 (dt, $^3J_{P,H}$ = 20.0 Hz, $^3J_{H,H}$ = 7.4 Hz, 3H) ppm. ^{13}C NMR (126 MHz, $CDCl_3$): δ = 135.23 (d, $^4J_{P,C}$ = 3.0 Hz, 3x CH), 133.74 (d, $^3J_{P,C}$ = 9.9 Hz, 6x CH), 130.67 (d, $^2J_{P,C}$ = 12.5 Hz, 6x CH), 117.95 (d, $^1J_{P,C}$ = 85.9 Hz, 3x C_q), 17.58 (d, $^1J_{P,C}$ = 51.9 Hz, CH_2), 7.02 (d, $^2J_{P,C}$ = 5.2 Hz, CH_3) ppm.

(*E*)- and (Z)-*tert*-Butyl(((1,1-$^2H_2$)-3-methylpent-3-en-1-yl)oxy)diphenylsilane (S7)
ETPPI (10.7 g, 25.6 mmol, 1.7 eq.) was suspended in THF (120 mL), and the mixture was cooled to 0 °C. n-BuLi (1.6 M in hexane, 16 mL, 25.6 mmol, 1.7 eq.) was added dropwise and the mixture was stirred at 0 °C for 1 h. Then the solution was cooled to –78 °C and **S6** (4.9 g, 14.9 mmol, in 4 mL THF) was added. The reaction mixture was allowed to slowly warm to room temperature over night. The reaction was quenched by pouring into an ice-water mixture (200 mL) and the product was extracted with Et_2O (3x 80 mL). The combined organic layers were washed with brine and dried with $MgSO_4$ followed by concentration under reduced pressure. The crude product was purified by silica gel chromatography [cyclohexane/EA (40/1), R_f = 0.42] to afford **S7** (*E*:*Z* = 2:3, 3.83 g, 11.25 mmol, 75%) as a colourless oil. The mixture of *E* and *Z* stereoisomers was only partially separated to obtain spectroscopic data for the pure stereoisomers. The synthesis was continued with the mixture of (*E*)- and (*Z*)-**S7**.
(*E*)-*tert*-Butyl(((1,1-$^2H_2$)-3-methylpent-3-en-1-yl)oxy)diphenylsilane (*E*)-S7. ^{1}H NMR (500 MHz, C_6D_6): δ = 7.80 – 7.78 (m, 4H, 4x CH), 7.24 – 7.21 (m, 6H, 6x CH), 5.28-5.21 (m, 1H, CH), 2.27 (s, 2H, CH_2), 1.50 (dq, $^3J_{H,H}$ = 6.7 Hz, $^5J_{H,H}$ =1.1 Hz, 3H, CH_3), 1.46 (p, $^4J_{H,H}$ = 1.1 Hz, $^5J_{H,H}$= 1.1 Hz, 3H, CH_3), 1.19 (s, 9H, 3x CH_3) ppm. ^{13}C NMR (126 MHz, C_6D_6): δ = 136.06 (4x CH), 134.54 (2x C_q), 132.90 (C_q), 129.92 (2x CH), 128.06 (4x CH), 120.83 (CH), 43.03 (CH_2), 27.14 (3x CH_3), 19.51 (C_q), 16.02 (CH_3), 13.56 (CH_3) ppm. GC (HP-5MS): *I* = 2196. MS (EI, 70 eV): *m*/*z* (%) = 283 (100), 251 (3), 241 (2), 226 (5), 213 (5), 205 (39), 199 (65), 183 (23), 173 (10), 163 (2), 155 (2), 145 (4), 135 (11), 127 (3), 121 (6), 105 (6), 93 (1), 77 (6), 69 (1), 57 (2), 41 (2).
(*Z*)-*tert*-Butyl(((1,1-$^2H_2$)-3-methylpent-3-en-1-yl)oxy)diphenylsilane (*Z*)-S7. ^{1}H NMR (500 MHz, C_6D_6): δ = 7.80 – 7.78 (m, 4H, 4x CH), 7.24 – 7.22 (m, 6H, 6x CH), 5.32 – 5.20 (m, 1H, CH), 2.31 (s, 2H, CH_2), 1.61 (p, $^4J_{H,H}$ = 1.5 Hz, $^5J_{H,H}$ =1.5 Hz, 3H, CH_3), 1.51 – 1.47 (m, 3H, CH_3), 1.19 (s, 9H, 3x CH_3) ppm. ^{13}C NMR (126 MHz, C_6D_6): δ = 136.06 (4x CH), 134.41 (2x C_q), 132.89 (C_q), 129.95 (2x CH), 128.06 (4x CH), 121.22 (CH), 35.07 (CH_2), 27.13 (3x CH_3), 24.15 (CH_3), 19.45 (C_q), 13.54 (CH_3) ppm. GC (HP-5MS): *I* = 2187. MS (EI, 70 eV): *m*/*z* (%) = 283 (100), 251 (2), 241 (2), 226 (6), 213 (6), 205 (45), 199 (77), 183 (31), 173 (13), 163 (4), 155 (3), 145 (7), 135 (20), 127 (6), 121 (10), 105 (13), 93 (3), 77 (14), 69 (2), 57 (5), 41 (5).

(*E*)- and (*Z*)-(1,1-$^2H_2$)-3-Methylpent-3-en-1-yl 4-methylbenzenesulfonate (S8)
To a solution of **S7** (2.04 g, 5.99 mmol) in THF (30 mL) was added TBAF (1 M in THF, 7.2 mL, 7.20 mmol, 1.2 eq.) dropwise at 0 °C. After the solution was stirred at room temperature for 2.5 h, the reaction was quenched by pouring into ice-water (150 mL), followed by extraction with Et_2O (3x 50 mL). The combined organic layers were washed with brine and concentrated

under reduced pressure to afford the crude product. Purification by silica gel chromatography [pentane/Et_2O (1/1), R_f = 0.40] gave (1,1-$^2H_2$)-**7ab** as a colourless oil. The diastereomeric mixture of (1,1-$^2H_2$)-**7ab** was used for the next step directly.

The diastereomeric mixture of **(1,1-^{2}H$_2$)7ab** and DMAP (2.43 g, 19.9 mmol, 3.3 eq.) were dissolved in DCM (30 mL) and the solution was cooled to 0 °C. TsCl (2.86 g, 15.0 mmol, 2.5 eq., in 5 mL DCM) was added dropwise and the mixture was stirred at room temperature overnight. The reaction was then quenched by adding sat. aq. NH_4Cl, followed by extraction with Et_2O (3x 50 mL). The combined organic layers were dried with $MgSO_4$ and concentrated under reduced pressure. Purification via flash column chromatography [cyclohexane/EA (10/1), R_f = 0.34] provided pure (*E*)- and (*Z*)-**S8** (0.61 g, 2.38 mmol, 40%) as a colourless oil.

Separation of **S8** (200 mg) was performed by HPLC (column: DAICEL Chiralpak IC-U, < 2,0 µm; 3,0 mm x 100 mm) with hexane/*i*PrOH (95:5) at 0.85 mL/min to provide (*E*)-**S8** (110 mg) and (Z)-**S8** (70 mg).

(*E*)-(1,1-^{2}H$_2$)-3-Methylpent-3-en-1-yl 4-methylbenzenesulfonate (*E*)-S8. ^{1}H NMR (700 MHz, $CDCl_3$): δ = 7.80 – 7.75 (m, 2H, 2x CH), 7.35 – 7.32 (m, 2H, 2x CH), 5.23-5.18 (m, 1H, CH), 2.45 (s, 3H, CH_3), 2.29 (s, 2H, CH_2), 1.53 (dq, $^3J_{H,H}$ = 6.7 Hz, $^5J_{H,H}$ =1.0 Hz, 3H, CH_3), 1.51 (s, 3H) ppm. ^{13}C NMR (176 MHz, $CDCl_3$): δ = 144.74 (C_q), 133.47 (C_q), 130.20 (C_q), 129.90 (2x CH_2), 128.04 (2x CH_2), 122.35 (CH), 38.62 (CH_2), 21.79 (CH_3), 15.72 (CH_3), 13.57 (CH_3) ppm. GC (HP-5MS): *I* = 1953. MS (EI, 70 eV): *m*/*z* (%) = 173 (2), 155 (12), 107 (1), 91 (40), 84 (100), 69 (70), 56 (4), 41 (14).

(*Z*)-(1,1-^{2}H$_2$)-3-Methylpent-3-en-1-yl 4-methylbenzenesulfonate (*Z*)-S8. ^{1}H NMR (700 MHz, $CDCl_3$): δ = 7.80 – 7.77 (m, 2H, 2x CH), 7.36 – 7.32 (m, 2H, 2x CH), 5.33-5.29 (m, 1H, CH), 2.45 (s, 3H, CH_3), 2.37 (s, 2H, CH_2), 1.60 (p, $^4J_{H,H}$ = 1.5 Hz, $^5J_{H,H}$ =1.5 Hz, 3H, CH_3), 1.50 (d, $^3J_{H,H}$ = 6.8 Hz, 3H) ppm. ^{13}C NMR (176 MHz, $CDCl_3$): δ = 144.78 (C_q), 133.46 (C_q), 130.03 (C_q), 129.91 (2x CH_2), 128.00 (2x CH_2), 123.07 (CH), 31.04 (CH_2), 23.50 (CH_3), 21.78 (CH_3), 13.45 (CH_3) ppm. GC (HP-5MS): *I* = 1931. MS (EI, 70 eV): *m*/*z* (%) = 173 (2), 155 (10), 107 (1), 91 (41), 84 (100), 69 (81), 56 (5), 41 (16).

Trisammonium (*E*)- and (*Z*)-(1,1-^{2}H$_2$)-4-methylisopentenyl diphosphate ((1,1-^{2}H$_2$)-8ab)

Following the same procedure as for the preparation of unlabelled **8a** and **8b**, (*E*)-**S8** (110 mg, 0.43 mmol) and (*Z*)-**S8** (70 mg, 0.27 mmol) were converted into (1,1-$^2H_2$)-**8a** (106 mg, 0.34 mmol, 79%) and (1,1-$^2H_2$)-**8b** (68 mg, 0.22 mmol, 81%) that were obtained as colourless powders.

Trisammonium (*E*)-(1,1-^{2}H$_2$)-4-methylisopentenyl diphosphate (1,1-^{2}H$_2$)-8a. ^{1}H NMR (500 MHz, D_2O): δ = 5.42 – 5.34 (m, 1H, CH), 2.33 (s, 2H, CH_2), 1.68 – 1.66-1.65 (m, 3H, CH_3), 1.58 (dq, $^3J_{H,H}$ = 6.7 Hz, $^4J_{H,H}$ =1.2 Hz, 3H, CH_3) ppm. ^{13}C NMR (126 MHz, D_2O): δ = 133.16 (C_q), 121.21 (CH), 39.46 (d, $^3J_{P,C}$ = 7.2 Hz, CH_2), 14.85 (CH_3), 12.67 (CH_3) ppm. ^{31}P NMR (202 MHz, D_2O): δ = –8.77(br s, 1P), –10.56 (br s, 1P).

Trisammonium (*Z*)-(1,1-^{2}H$_2$)-4-methylisopentenyl diphosphate (1,1-^{2}H$_2$)-8b. ^{1}H NMR (500 MHz, D_2O): δ = 5.46 – 5.40 (m, 1H, CH), 2.43 (s, 2H, CH_2), 1.74 (p, $^4J_{H,H}$ = 1.5 Hz, $^5J_{H,H}$ =1.5 Hz, 3H, CH_3), 1.61 (dq, $^3J_{H,H}$ = 7.0 Hz, $^5J_{H,H}$ =1.5 Hz, 3H, CH_3) ppm. ^{13}C NMR (126 MHz, D_2O): δ = 133.22 (C_q), 121.90 (CH), 31.84 (d, $^3J_{P,C}$ = 7.0 Hz, CH_2), 22.69 (CH_3), 12.69 (CH_3) ppm. ^{31}P NMR (202 MHz, D_2O): δ = –8.98 (br s, 1P), –10.68 (br s, 1P) ppm.

Synthesis of (*R*)- and (*S*)-(1-^{13}C,1-^{2}H)-8ab

Benzyl (1-^{13}C)acetate (S9)

(1-^{13}C)Acetic acid (1.00 g, 16.4 mmol), DMAP (0.2 g, 1.64 mmol, 0.1 eq.) and benzyl alcohol (3.55 g, 32.8 mmol, 2.0 eq.) were dissolved in DCM (10 mL), and the solution was cooled to 0 °C. A solution of DCC (4.06 g, 19.7 mmol, 1.2 eq.) in DCM (5 mL) was added dropwise. The

reaction mixture was then allowed to warm to room temperature and stirred for 4 h. The mixture was filtrated and the residual solid was washed several times with DCM. The combined filtrate was concentrated under reduced pressure to afford the crude product. Purification by silica gel chromatography [pentane/Et_2O (5/1), R_f = 0.47] provided **S9** (2.37 g, 15.7 mmol, 96%) as a colourless oil.

Benzyl (1-^{13}C)acetate (S9). ^{1}H NMR (300 MHz, $CDCl_3$): δ = 7.40 – 7.31 (m, 5H, 5x CH), 5.11 (d, $^3J_{C,H}$ = 3.1 Hz, 2H, CH_2), 2.11 (d, $^2J_{C,H}$= 6.8 Hz, 3H, CH_3) ppm. ^{13}C NMR (75 MHz, $CDCl_3$): δ = 171.02 ($^{13}C_q$), 136.07 (d, $^3J_{C,C}$ = 1.8 Hz, C_q), 128.70 (2x CH), 128.39 (3x CH), 66.45 (d, $^2J_{C,C}$ = 2.5 Hz, CH_2), 21.14 (d, $^1J_{C,C}$ = 59.4 Hz, CH_3) ppm. GC (HP-5MS): *I* = 1169. MS (EI, 70 eV): *m*/*z* (%) = 151(28), 108 (100), 91 (52), 79 (23), 65 (11), 51 (6), 44 (18).

Benzyl (1-^{13}C)-3-oxobutanoate (S10)

The reaction was followed to a pubilshed procedure.[10] To a solution of LiHMDS (1 M in THF, 31.4 mL, 31.4 mmol, 2.0 eq.) was added a solution of **S9** (2.37 g, 15.7 mmol) in THF (5 mL) dropwise at –78 °C. The reaction mixture was then stirred at –78 °C for 45 min. Acetyl chloride (1.23 g, 15.67 mmol, 1.0 eq.) was added dropwise and stirring was continued at the same temperature for 2.5 h. The reaction was quenched by the addition of HCl (6 M, 2.4 mL) at –78 °C, followed by the addition of H_2O (100 mL) and extraction with Et_2O (3x 50 mL). The combined organic layers were washed with HCl (3 M, 30 mL), water, sat. $NaHCO_3$ (40 mL) and brine and dried with $MgSO_4$, followed by concentration under reduced pressure to afford the crude product. Purification via flash chromatography [cyclo-hexane/EA (10/1), R_f = 0.32] gave **S10** (2.16 g, 11.2 mmol, 71%) as a colourless oil.

Benzyl (1-^{13}C)-3-oxobutanoate (S10). ^{1}H NMR (500 MHz, $CDCl_3$): δ = 7.40 – 7.31 (m, 5H, 5x CH), 5.18 (d, $^3J_{C,H}$ = 3.3 Hz, 2H, CH_2), 3.50 (d, $^2J_{C,H}$ = 7.4 Hz, 2H, CH_2), 2.25 (s, 3H, CH_3) ppm. ^{13}C NMR (126 MHz, $CDCl_3$): δ = 200.48 (d, $^2J_{C,C}$= 2.3 Hz, C_q), 167.07 ($^{13}C_q$) , 135.38 (d, $^3J_{C,C}$ = 2.2 Hz, C_q), 128.77 (2x CH), 128.63 (CH), 128.53 (2x CH), 67.30 (d, $^2J_{C,C}$ = 2.5 Hz, CH_2), 50.19 (d, $^1J_{C,C}$ = 58.6 Hz, CH_2), 30.32 (CH_3) ppm. GC (HP-5MS): *I* = 1498. MS (EI, 70 eV): *m*/*z* (%) = 193 (3), 164 (14), 107 (100), 91 (83), 79 (38), 65 (14), 58 (19), 51 (8), 43 (25).

Benzyl (1-^{13}C)-2-(2,5,5-trimethyl-1,3-dioxan-2-yl)acetate (S11)

To a solution of neopentyl glycol (2.56 g, 24.58 mmol, 2.2 eq.) and **S10** (2.16 g, 11.2 mmol) in DCM (60 mL) was added TMSCl (5.35 g, 49.3 mmol, 4.4 eq.) dropwise at room temperature. The solution was then heated to reflux for 12 h. After cooling to room temperature, aq. $NaHCO_3$ (5%, 60 mL) was added and the product was extracted with Et_2O (3x 80 mL). The combined organic layers were washed with brine, dried with $MgSO_4$ and concentrated under reduced pressure. Product **S11** (2.9 g, 10.38 mmol, 93%) was obtained by purification on flash column chromatography [cyclohexane/EA (10/1), R_f = 0.28].

Benzyl (1-^{13}C)-2-(2,5,5-trimethyl-1,3-dioxan-2-yl)acetate (S11). ^{1}H NMR (500 MHz, C_6D_6): δ = 7.22 – 7.19 (m, 2H, 2x CH), 7.12 – 7.00 (m, 3H, 3x CH), 5.03 (d, $^3J_{C,H}$ = 3.3 Hz, 2H, CH_2), 3.38 – 3.23 (m, 4H, 2x CH_2), 2.79 (d, $^2J_{C,H}$ = 6.9 Hz, 2H, CH_2), 1.65 (s, 3H, CH_3), 0.75 (s, 3H, CH_3), 0.62 (s, 3H, CH_3) ppm. ^{13}C NMR (126 MHz, C_6D_6): δ = 169.05 ($^{13}C_q$), 97.76 (C_q), 70.56 (2x CH_2), 66.29 (d, $^2J_{C,C}$= 2.5 Hz, CH_2), 41.55 (d, $^1J_{C,C}$ = 59.2 Hz, CH_2), 29.73 (C_q), 23.49 (CH_3), 22.61 (CH_3), 22.37 (CH_3) ppm. GC (HP-5MS): *I* = 1911. MS (EI, 70 eV): *m*/*z* (%) = 264 (24), 194 (4), 164 (2), 144 (5), 129 (100), 107 (24), 91 (81), 86 (6), 79 (3), 69 (35), 56 (2), 43 (33).

(1-^{13}C,1,1-^{2}H$_2$)-2-(2,5,5-trimethyl-1,3-dioxan-2-yl)ethan-1-ol (S12)

Following the same procedure as for **S4**, **S11** (2.9 g, 10.4 mmol) was converted into **S12** (1.78 g, 10.10 mmol, 97%).

(1-^{13}C,1,1-^{2}H$_2$)-2-(2,5,5-trimethyl-1,3-dioxan-2-yl)ethan-1-ol (S12). TLC: R_f [cyclohexane/EA (2/1)] = 0.25. ^{1}H NMR (500 MHz, C_6D_6): δ = 3.36 – 3.01 (m, 4H, 2x CH_2), 2.78 (d, $^2J_{C,H}$ = 3.2 Hz, 1H, OH), 1.85 (d, $^2J_{C,H}$ = 4.1 Hz, 2H, CH_2), 1.16 (s, 3H, CH_3), 0.96 (s, 3H,

CH_3), 0.39 (s, 3H, CH_3). ^{13}C NMR (126 MHz, C_6D_6): δ = 100.10 (d, $^2J_{C,C}$ = 1.7 Hz, C_q), 70.23 (2x CH_2), 58.09 (p, $^1J_{C,D}$ = 21.8 Hz, $^{13}C_q$), 42.30 (d, $^1J_{C,C}$ = 36.1 Hz, CH_2), 29.74 (C_q), 22.81 (CH_3), 22.03 (CH_3), 19.07 (d, $^3J_{C,C}$ = 1.8 Hz, CH_3) ppm. GC (HP-5MS): *I* = 1237. MS (EI, 70 eV): *m*/*z* (%) = 179 (1), 162 (31), 129 (45), 92 (28), 76 (46), 69 (66), 56 (46), 43 (100).

(1-^{13}C,1-$^2H_2$)-2-(2,5,5-Trimethyl-1,3-dioxan-2-yl)acetaldehyde (S13)
IBX (3.93 g, 14.0 mmol, 1.4 eq.) was dissolved in DMSO (60 mL) and **S12** (1.78 g, 10.10 mmol, in 10 mL DMSO) was added dropwise at room temperature. The reaction mixture was stirred at room temperature overnight and then diluted with Et_2O (50 mL), cooled to 0 °C, followed by the addition of sat. aq. $NaHCO_3$ (100 mL). The aqueous layer was extracted with Et_2O (2x 80 mL). The combined organic layers were dried with $MgSO_4$ and concentrated under reduced pressure. The residue was purified by silica gel chromatography [cyclohexane/EA (5/1), R_f = 0.37] to afford **S13** (1.39 g, 8.03 mmol, 80%) as a colourless oil.
(1-^{13}C,1-$^2H_2$)-2-(2,5,5-Trimethyl-1,3-dioxan-2-yl)acetaldehyde (S13). ^{1}H NMR (500 MHz,C_6D_6): δ = 3.25 – 3.08 (m, 4H, 2x CH_2), 2.45 (d, $^2J_{C,H}$ = 6.6 Hz, 2H, CH_2), 1.16 (s, 3H, CH_3), 0.77 (s, 3H, CH_3), 0.51 (s, 3H, CH_3) ppm. ^{13}C NMR (126 MHz, C_6D_6): δ = 198.81 (t, $^1J_{C,D}$ = 27.0 Hz, ^{13}CD), 97.65 (d, $^2J_{C,C}$ = 1.0 Hz, C_q), 70.34 (2x CH_2), 51.60 (dt, $^1J_{C,C}$ = 40.2 Hz, $^2J_{C,D}$ = 3.5 Hz, CH_2), 29.61 (C_q), 22.79 (CH_3), 22.22 (CH_3), 20.82 (d, $^3J_{C,C}$ = 1.6 Hz, CH_3) ppm. GC (HP-5MS): *I* = 1182. MS (EI, 70 eV): *m*/*z* (%) = 159 (45), 129 (76), 89 (15), 73 (44), 69 (82), 56 (84), 43 (100).

(*R*)- and (*S*)-(1-^{13}C,1-$^2H_2$)-2-(2,5,5-Trimethyl-1,3-dioxan-2-yl)ethan-1-ol (S14)
According to published article,[11] the aldehyde **S13** (1.39 g, 8.03 mmol, in 2 mL THF) was added to a solution of (*S*)-Alpine borane (0.5 M in THF, 19.3 mL, 9.64 mmol, 1.2 eq.) dropwise at 0 °C. The solution was then allowed warm to room temperature and stirred for 6 h. The reaction was quenched by adding 218 μL acetic aldehyde, followed by removing of the solvent under reduced pressure. The residue was subjected to high vacuum for 1 h. The residue was taken up with Et_2O (10 mL) and cooled to 0 °C, followed by addition of 526 μL ethanolamine. The mixture was stirred at 0 °C during which a precipitate formed, followed by filtration. The filter cake was washed several times with Et_2O. The filtrate was washed with H_2O (10 mL) and brine, and concentrated under reduced pressure. Purification via flash column chromatography [cyclohexane/EA (2/1), R_f = 0.25] gave (*R*)-**S14** (1.19 g, 6.79 mmol, 85%, 90% *ee*) as a colourless oil.
Following the same procedure, **S13** (1.62 g, 9.30 mmol) was converted into (*S*)-**S14** (1.39 g, 7.89 mmol, 85%, 94% *ee*) using (*R*)-Alpine borane solution (0.5 M in THF, 23.0 mL, 11.5 mmol, 1.2 eq.).
The enantiomeric excesses were determined by ^{1}H NMR analysis of Mosher esters obtained from (*S*)-(+)-MTPA-Cl[12] (Figure S100).
(*R*)- and (*S*)-(1-^{13}C,1-$^2H_2$)-2-(2,5,5-Trimethyl-1,3-dioxan-2-yl)ethan-1-ol (S14). Both compounds showed the same analytical data. ^{1}H NMR (500 MHz, C_6D_6): δ = 4.12 – 3.71 (m, 1H, ^{13}CDH), 3.34 – 3.08 (m, 4H, 2x CH_2), 2.78 (br s, 1H, OH), 1.86 (t, $^2J_{C,H}$ = 4.9 Hz, $^3J_{H,H}$ = 4.9 Hz, 2H, CH_2), 1.16 (s, 3H, CH_3), 0.96 (s, 3H, CH_3), 0.39 (s, 3H, CH_3) ppm. ^{13}C NMR (126 MHz, C_6D_6): δ = 100.10 (d, $^2J_{C,C}$ = 2.0 Hz, C_q), 70.23 (2x CH_2), 58.44 (t, $^1J_{C,D}$ = 21.7 Hz, ^{13}CDH), 42.40 (d, $^1J_{C,C}$ = 36.3 Hz, CH_2), 29.74 (C_q), 22.81 (CH_3), 22.03 (CH_3), 19.08 (d, $^3J_{C,C}$ = 1.5 Hz, CH_3) ppm. GC (HP-5MS): *I* = 1238. MS (EI, 70 eV): *m*/*z* (%) = 161 (35), 129 (44), 91 (28), 75 (44), 73 (23), 69 (62), 56 (43), 43 (100), 41 (41).

Compounds **S15 – S17** and (*R*)- and (*S*)-(1-^{13}C,1-^{2}H)-**7ab** were synthesized following the same procedures as for the preparation of **S5 – S7** and (1,1-$^2H_2$)-**7ab**.

(*R*)- and (*S*)-*tert*-Butyldiphenyl((1-^{13}C,1-^{2}H)-2-(2,5,5-trimethyl-1,3-dioxan-2-yl)ethoxy)silane (S15)

(*R*)-S15. Yield: 2.69 g (6.49 mmol, 96%). ^{1}H NMR (500 MHz, C_6D_6): δ = 7.86 – 7.79 (m, 4H, 4x CH), 7.25 – 7.18 (m, 6H, 6x CH), 4.06 (dt, $^{1}J_{C,H}$ = 142.6 Hz, $^{3}J_{H,H}$ = 7.3 Hz, 1H, ^{13}CDH), 3.32 – 3.10 (m, 4H, 2x CH_2), 2.23 (dd, $^{3}J_{H,H}$ = 7.3 Hz, $^{2}J_{C,H}$ = 5.7 Hz, 2H, CH_2), 1.34 (s, 3H, CH_3), 1.20 (s, 9H, 3x CH_3), 0.72 (s, 3H, CH_3), 0.64 (s, 3H, CH_3) ppm. ^{13}C NMR (126 MHz, C_6D_6): δ = 136.06 (4x CH), 134.46 (2x C_q), 129.92 (2x CH), 128.09 (4x CH), 98.24 (C_q), 70.20 (2x CH_2), 60.23 (t, $^{1}J_{C,D}$ = 27.8 Hz, ^{13}CDH), 40.45 (d, $^{1}J_{C,C}$= 39.4 Hz, CH_2), 29.79 (C_q), 27.17 (3x CH_3), 22.68 (CH_3), 22.53 (CH_3), 21.83 (CH_3), 19.46 (C_q) ppm. GC (HP-5MS): *I* = 2573. MS (EI, 70 eV): *m*/*z* (%) = 399 (2), 357 (1), 283 (2), 271 (100), 253 (3), 239 (31), 227 (2), 221 (3), 213 (4), 199 (30), 193 (61), 181 (5), 175 (5), 167 (8), 161 (8), 139 (12), 129 (25), 121 (3), 115 (3), 105 (3), 93 (2), 77 (5), 69 (10), 57 (2), 43 (12).

(*S*)-S15. Yield: 3.15 g (7.60 mmol, 96%). Same analytical data as for (*R*)-**S15**.

(*R*)- and (*S*)-(4-^{13}C,4-^{2}H)-4-((*tert*-Butyldiphenylsilyl)oxy)butan-2-one (S16)

(*R*)-S16. Yield: 1.73 g (5.27 mmol, 81%). ^{1}H NMR (500 MHz, C_6D_6): δ = 7.79 – 7.74 (m, 4H, 4x CH), 7.28 – 7.20 (m, 6H, 6x CH), 3.82 (dt, $^{1}J_{C,H}$ =144.0 Hz, $^{3}J_{H,H}$ = 6.2 Hz, 1H, ^{13}CDH), 2.18 (dd, $^{3}J_{H,H}$ = 6.1 Hz, $^{2}J_{C,H}$ = 4.7 Hz, 2H, CH_2), 1.70 (s, 3H, CH3), 1.14 (s, 9H, 3x CH_3) ppm. ^{13}C NMR (126 MHz, C_6D_6): δ = 205.09 (d, $^{2}J_{C,C}$ = 2.0 Hz, C_q), 136.01 (4x CH), 133.99 (2x C_q), 130.07 (2x CH), 128.14 (4x CH), 59.65 (t, $^{1}J_{C,D}$ = 22.0 Hz, ^{13}CDH), 45.94 (d, $^{1}J_{C,C}$ = 39.4 Hz, CH_2), 30.09 (CH_3), 27.05 (3x CH_3), 19.42 (C_q) ppm. GC (HP-5MS): *I* = 2203. MS (EI, 70 eV): *m*/*z* (%) = 271 (100), 253 (3), 239 (56), 227 (2), 221 (5), 213 (6), 199 (49), 193 (69), 181 (10), 175 (7), 167 (8), 161 (13), 151 (2), 139 (11), 121 (4), 115 (4), 105 (3), 93 (2), 77 (8), 57 (1), 45 (3).

(*S*)-S16. Yield: 2.14 g (6.51 mmol, 86%). Same analytical data as for (*R*)-**S16**.

(*R*,*E*)-, (*R*,*Z*)-, (*S*,*E*)- and (*S*,*Z*)-(1-^{13}C,1-^{2}H)-3-Methylpent-3-en-1-ol ((*R*)- and (*S*)-(1-^{13}C,1-^{2}H)-7ab)

The protected alcohols (*R*)- and (*S*)-**S16** were converted into (*R*)- and (*S*)-**S17** that were obtained as *E*,*Z* mixtures. These mixtures were used for the synthesis of (*R*)- and (*S*)-(1-^{13}C,1-^{2}H)-**7a** and (*R*)- and (*S*)-(1-^{13}C,1-^{2}H)-**7b** that were obtained in pure form by separation of the *E* and *Z* stereoisomers through column chromatography.

(*R*)-(1-^{13}C,1-^{2}H)-7a. Yield: 80 mg (0.78 mmol, 27%). ^{1}H NMR (500 MHz, C_6D_6): δ = 5.22 – 5.16 (m, 1H, CH), 3.60 – 3.28 (m, 1H, ^{13}CDH), 2.05 (t, $^{3}J_{H,H}$ = 6.4 Hz, $^{2}J_{C,H}$ = 6.4 Hz, 2H, CH_2), 1.46 (d, $^{3}J_{H,H}$ = 6.7 Hz, 3H, CH_3), 1.43 (s, 3H, CH_3), 0.97 (br s, 1H, OH) ppm. ^{13}C NMR (126 MHz, C_6D_6): δ = 132.64 (d, $^{2}J_{C,C}$ = 2.5 Hz, C_q), 121.31 (d, $^{3}J_{C,C}$ = 3.0 Hz, CH), 60.23 (t, $^{1}J_{C,D}$ = 21.7 Hz, ^{13}CDH), 43.14 (d, $^{1}J_{C,C}$ = 35.8 Hz, CH_2), 15.51 (d, $^{3}J_{C,C}$ = 1.3 Hz, CH_3), 13.49 (CH_3) ppm. GC (HP-5MS): *I* = 856. MS (EI, 70 eV): *m*/*z* (%) = 102 (37), 84 (28), 69 (100), 55 (33), 41 (86), 33 (9).

(*S*)-(1-^{13}C,1-^{2}H)-7a. Yield: 120 mg (1.17 mmol, 25%). Same analytical data as for (*S*)-(1-^{13}C,1-^{2}H)-**7a**.

(*R*)-(1-^{13}C,1-^{2}H)-7b. Yield: 40 mg (0.39 mmol, 13%). ^{1}H NMR (500 MHz, C_6D_6): δ = 5.29 – 5.23 (m, 1H, CH), 3.59 – 3.24 (m, 1H, ^{13}CDH), 2.12 (t, $^{3}J_{H,H}$ = 6.1 Hz, $^{2}J_{C,H}$ = 6.1 Hz, 2H, CH_2), 1.61 – 1.55 (m, 3H, CH_3), 1.50 (d, $^{3}J_{H,H}$=6.8 Hz, 3H, CH_3), 0.83 (br s, 1H, OH) ppm. ^{13}C NMR (126 MHz, C_6D_6): δ = 132.57 (d, $^{2}J_{C,C}$ = 2.4 Hz, C_q), 121.81 (d, $^{3}J_{C,C}$ = 2.8 Hz, CH), 60.30 (t, $^{1}J_{C,D}$ = 21.8 Hz, ^{13}CDH), 35.08 (d, $^{1}J_{C,C}$ = 35.9 Hz, CH_2), 23.67 (CH_3), 13.50 (CH_3) ppm. GC (HP-5MS): *I* = 850. MS (EI, 70 eV): *m*/*z* (%) = 102 (33), 84 (25), 69 (100), 55 (39), 41 (90), 33 (10).

(*S*)-(1-^{13}C,1-^{2}H)-7b. Yield: 80 mg (0.78 mmol, 16%). Same analytical data as for (*R*)-(1-^{13}C,1-^{2}H)-**7b.**

Trisammonium (*R*,*E*)-, (*R*,*Z*)-, (*S*,*E*)- and (*S*,*Z*)-(1-^{13}C,1-^{2}H)-4-methyl-IPP ((*R*)- and (*S*)-(1-^{13}C,1-^{2}H)-8ab)

The alcohol (*R*)-(1-^{13}C,1-^{2}H)-**7a** (80 mg, 0.78 mmol) was dissolved in trichloroacetonitrile (2 mL). A solution of TEAP,[13] prepared from 3.64 mL solution A (2.5 mL H_3PO_4, 9.4 mL acetonitrile) and 6 mL solution B (11 mL NEt_3, 10 mL acetonitrile), was added in three portions of 2 mL in intervals of 5 min. After that, the mixture was directly purified by silica gel chromotagraphy [*i*PrOH/25% NH_3/H_2O (6/2.5/1), R_f = 0.1]. The collected fractions containing the product were concentrated under reduced pressure and lyophilized to afford (*R*)-(1-^{13}C,1-^{2}H)-**8a** (70 mg, 0.23 mmol, 29%) as a pale yellow powder. Following the same procedure, (*R*)-(1-^{13}C,1-^{2}H)-**7b** (50 mg, 0.49 mmol), (*S*)-(1-^{13}C,1-^{2}H)-**7a** (120 mg, 1.17 mmol) and (*S*)-(1-^{13}C,1-^{2}H)-**7b** (80 mg, 0.78 mmol) were converted into (*R*)-(1-^{13}C,1-^{2}H)-**8b** (60 mg, 0.19 mmol, 39%), (*S*)-(1-^{13}C,1-^{2}H)-**8a** (120 mg, 0.38 mmol, 32%) and (*S*)-(1-^{13}C,1-^{2}H)-**8b** (90 mg, 0.29 mmol, 37%), respectively.

(*R*)-(1-^{13}C,1-^{2}H)-**8a** and (*S*)-(1-^{13}C,1-^{2}H)-**8a** had the same analytical data: ^{1}H NMR (500 MHz, D_2O): δ = 5.45 – 5.39 (m, 1H, CH), 4.00 (dq, $^{1}J_{C,H}$ = 146.7 Hz, $^{3}J_{H,H}$ =6.9 Hz, 1H, ^{13}CDH), 2.38 – 2.34 (m, 2H, CH_2), 1.68 (s, 3H, CH_3), 1.60 (d, $^{3}J_{H,H}$ = 6.7 Hz, 3H) ppm. ^{13}C NMR (126 MHz, D_2O): δ = 133.45 (C_q), 121.26 (d, $^{2}J_{C,C}$ = 3.5 Hz, CH), 64.20 (td, $^{1}J_{C,D}$ = 22.4 Hz, $^{2}J_{P,C}$ = 5.7 Hz, ^{13}CDH), 39.69 (dd, $^{1}J_{C,C}$ = 37.9 Hz, $^{3}J_{P,C}$ = 7.5 Hz), 14.97 (CH_3), 12.76 (CH_3) ppm. ^{31}P NMR (202 MHz, D_2O): δ = –7.52 (br s, 1P), –10.52 (d, $^{2}J_{P,P}$ = 22.0 Hz, 1P) ppm.

(*R*)-(1-^{13}C,1-^{2}H)-**8b** and (*S*)-(1-^{13}C,1-^{2}H)-**8b** had the same analytical data: ^{1}H NMR (500 MHz, D_2O): δ = 5.44 (q, $^{3}J_{H,H}$ = 7.0 Hz, 1H, CH), 3.99 (dq, $^{1}J_{C,H}$ = 147.3 Hz, $^{3}J_{H,H}$ =7.3 Hz, 1H, ^{13}CDH), 2.44 (t, $^{3}J_{H,H}$ = 6.2 Hz, 2H, CH_2), 1.75 (s, 3H, CH_3), 1.62 (d, $^{3}J_{H,H}$ = 6.7 Hz, 3H, CH_3) ppm. ^{13}C NMR (126 MHz, D_2O): δ = 133.33 (d, $^{2}J_{C,C}$ = 2.0 Hz, C_q), 121.99 (d, $^{3}J_{C,C}$ = 2.9 Hz, CH), 64.04 (td, $^{1}J_{C,D}$ = 22.5 Hz, $^{2}J_{P,C}$ = 5.9 Hz, ^{13}CDH), 32.02 (dd, $^{1}J_{C,C}$ = 37.9 Hz, $^{3}J_{P,C}$ = 6.8 Hz, CH_2), 22.78 (CH_3), 12.77 (CH_3) ppm. ^{31}P NMR (202 MHz, D_2O): δ = –9.03 (s, 1P), –10.69 (d, $^{2}J_{P,P}$ = 21.8 Hz, 1P) ppm.

Incubation experiments with isotopically labelled substrates and TmS

Isotopic labelling experiments were performed with amounts of ca. 1 mg labelled FPP (or its precursors) in 25 mM NH_4HCO_3 (1 mL), incubation buffer (5 mL), enzyme elution fractions and binding buffer (to 9 mL total volume) with the substrates and enzyme preparations as listed in Table S2. After incubation with shaking at 28 °C overnight the products were extracted with C_6D_6 (600 μL and 300 μL), the extracts were dried with $MgSO_4$ and analysed by NMR and/or GC/MS.

Table S2. Isotopic labelling experiments with TmS.

entry	substrates	enzymes	results shown in
1	(7-^{13}C)GPP[14] + (1,1-$^{2}H_2$)-**8a**	TmS, FPPS	Figures S96 and S98
2	(7-^{13}C)GPP + (1,1-$^{2}H_2$)-**8b**	TmS, FPPS	Figures S97 and S99
3	GPP + (*R*)-(1-^{13}C,1-^{2}H)-**8a**	TmS, FPPS	Figures S101, S102 and S106
4	GPP + (*S*)-(1-^{13}C,1-^{2}H)-**8a**	TmS, FPPS	Figures S101, S102 and S106
5	GPP + (*R*)-(1-^{13}C,1-^{2}H)-**8b**	TmS, FPPS	Figures S103, S104 and S105
6	GPP + (*S*)-(1-^{13}C,1-^{2}H)-**8b**	TmS, FPPS	Figures S103, S104 and S105
7	(3-^{13}C,2-^{2}H)GPP[15] + **8b**	TmS, FPPS	Figure S107
8	(2-^{2}H)DMAPP[16] + **8b**	TmS, FPPS	Figure S108
9	(*R*)-(1-^{13}C,1-^{2}H)GPP[17] + **8a**	TmS, FPPS	Figures S109, S112 and S113
10	(*S*)-(1-^{13}C,1-^{2}H)GPP[17] + **8a**	TmS, FPPS	Figures S109, S112 and S113
11	(*R*)-(1-^{13}C,1-^{2}H)GPP + **8b**	TmS, FPPS	Figures S110, S111 and S114
12	(*S*)-(1-^{13}C,1-^{2}H)GPP + **8b**	TmS, FPPS	Figures S110, S111 and S114

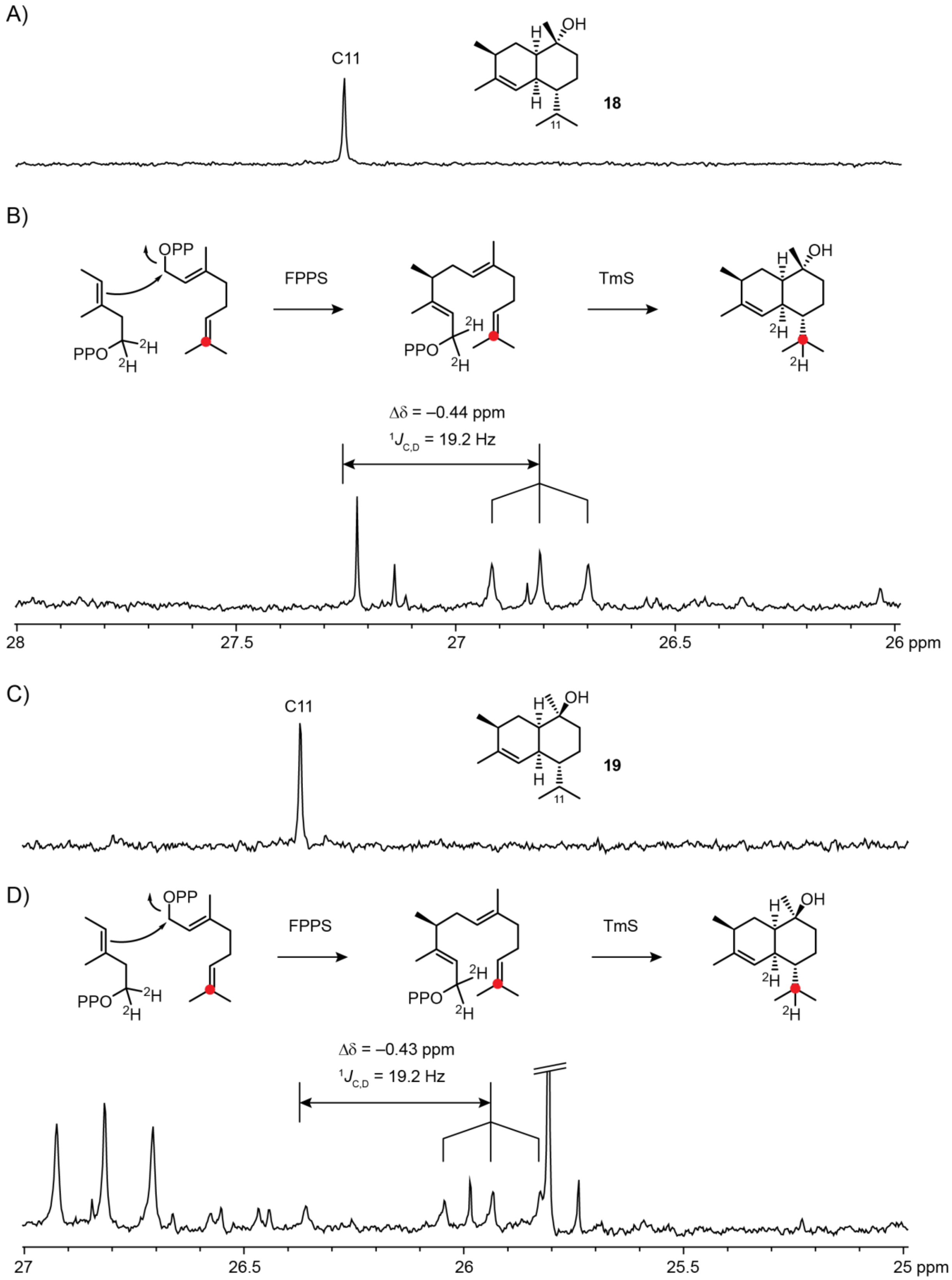

Figure S96. 1,3-Hydride shifts in the formation of products from **10a** with TmS. ^{13}C-NMR spectra showing A) the signal for C11 of unlabelled **18**, B) the signal for deuterated C11 of labelled **18** obtained from (1,1-$^{2}H_2$)-**8a** and (7-^{13}C)GPP, C) the signal for C11 of unlabelled **19**, and D) the signal for deuterated C11 of labelled **19** obtained from (1,1-$^{2}H_2$)-**8a** and (7-^{13}C)GPP. The slightly upfield shifted triplets are indicative for a direct ^{13}C-^{2}H bond and support the proposed 1,3-hydride shift.

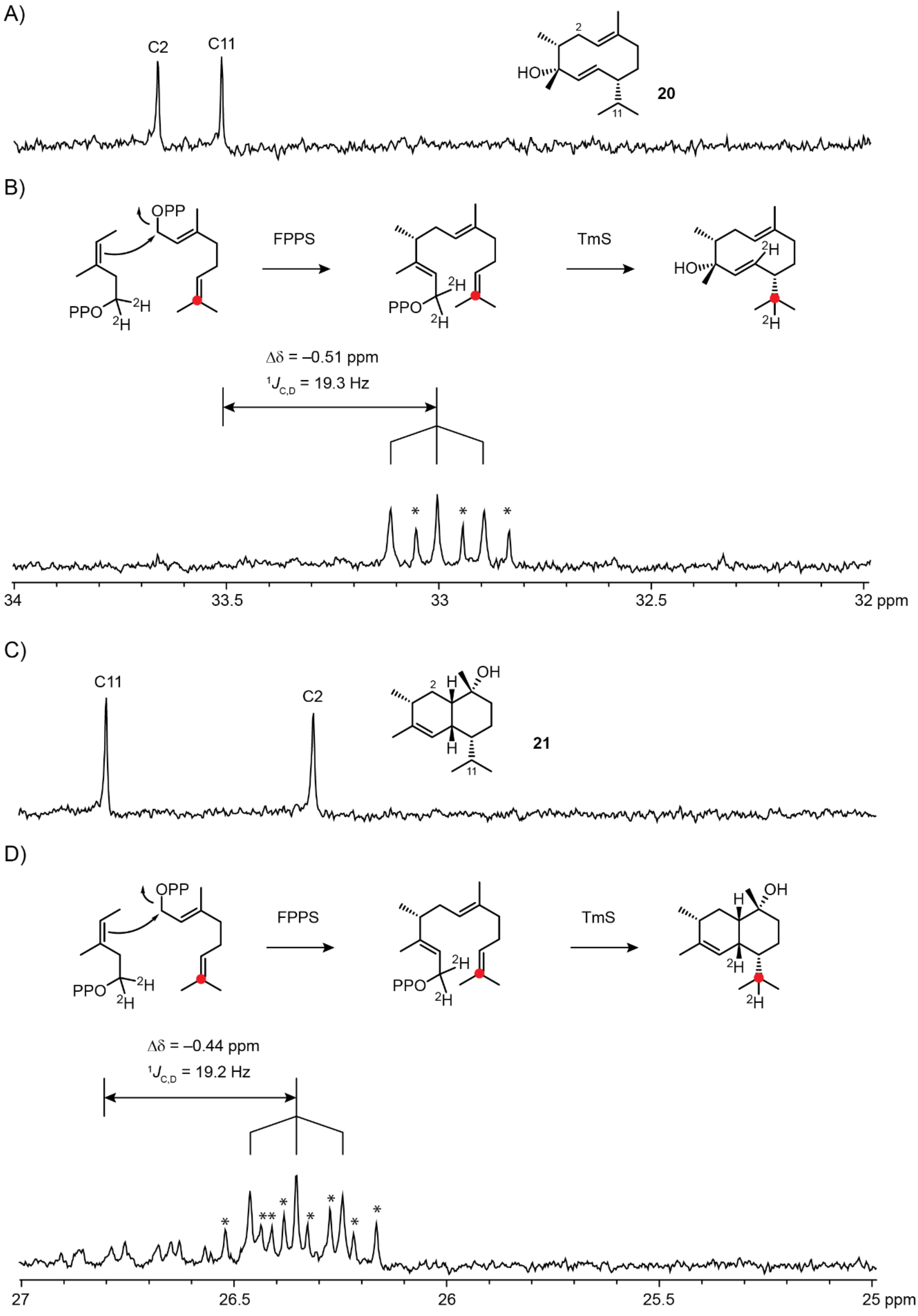

Figure S97. 1,3-Hydride shifts in the formation of products from **10b** with TmS. ^{13}C-NMR spectra showing A) the signal for C11 of unlabelled **20**, B) the signal for deuterated C11 of labelled **20** obtained from (1,1-$^{2}H_{2}$)-**8b** and (7-^{13}C)GPP, C) the signal for C11 of unlabelled **21**, and D) the signal for deuterated C11 of labelled **21** obtained from (1,1-$^{2}H_{2}$)-**8b** and (7-^{13}C)GPP. The slightly upfield shifted triplets are indicative for a direct ^{13}C-^{2}H bond and support the proposed 1,3-hydride shift.

E)

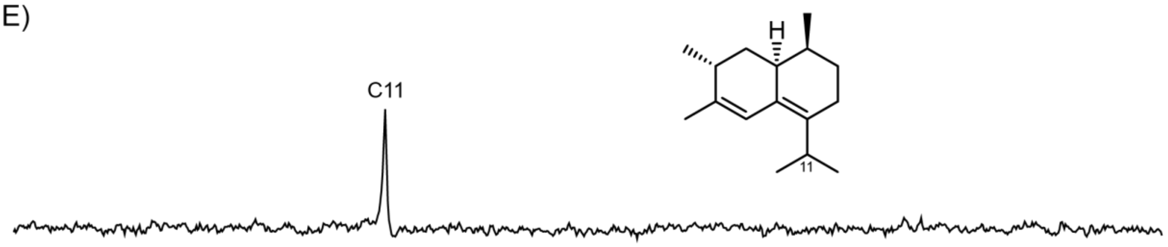

F)

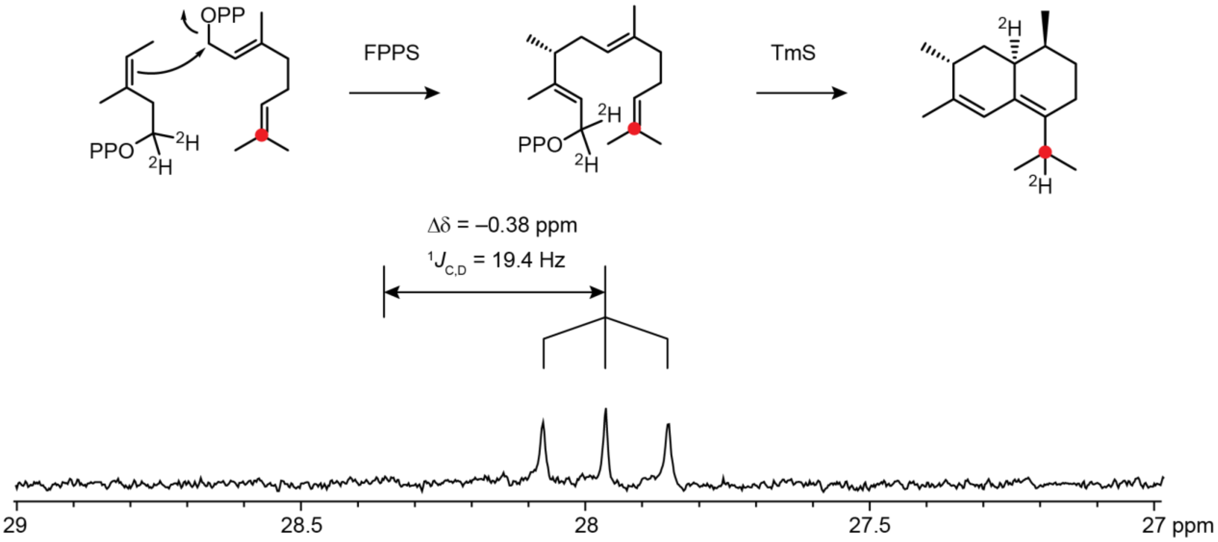

Figure S97 (continued). 1,3-Hydride shifts in the formation of products from **10b** with TmS. ^{13}C-NMR spectra showing E) the signal for C11 of unlabelled **22**, and F) the signal for deuterated C11 of labelled **22** obtained from (1,1-$^{2}H_{2}$)-**8b** and (7-^{13}C)GPP. The slightly upfield shifted triplet is indicative for a direct ^{13}C-^{2}H bond and supports the proposed 1,3-hydride shift.

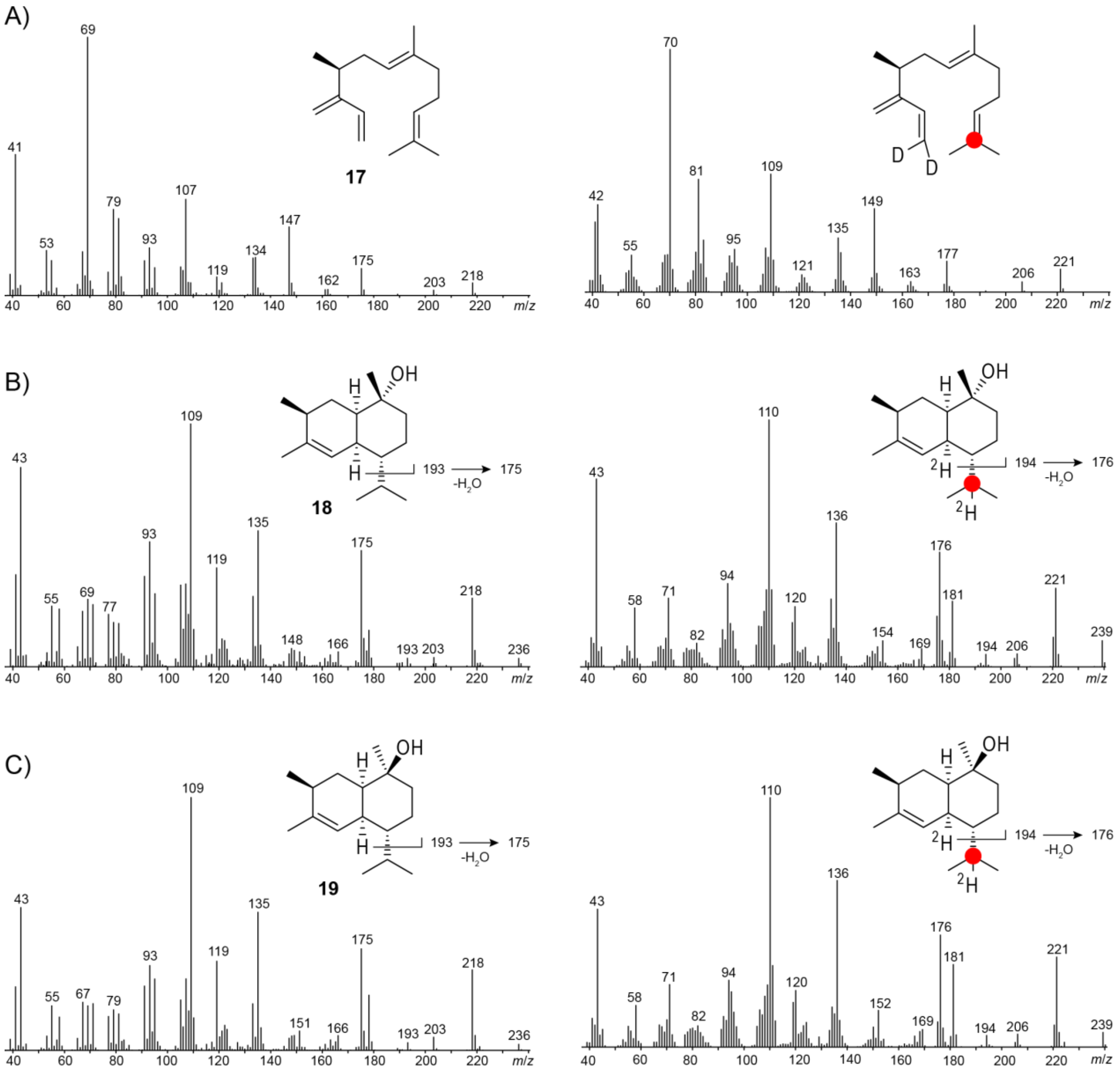

Figure S98. EI mass spectra of A) unlabelled and labelled **17**, B) unlabelled and labelled **18**, and C) unlabelled and labelled **19**. Labelled compounds were obtained from (1,1-$^2H_2$)-**8a** and (7-^{13}C)GPP. The fragment ions arising by cleavage of the *i*Pr group and loss of water locate one deuterium with the *i*Pr group.

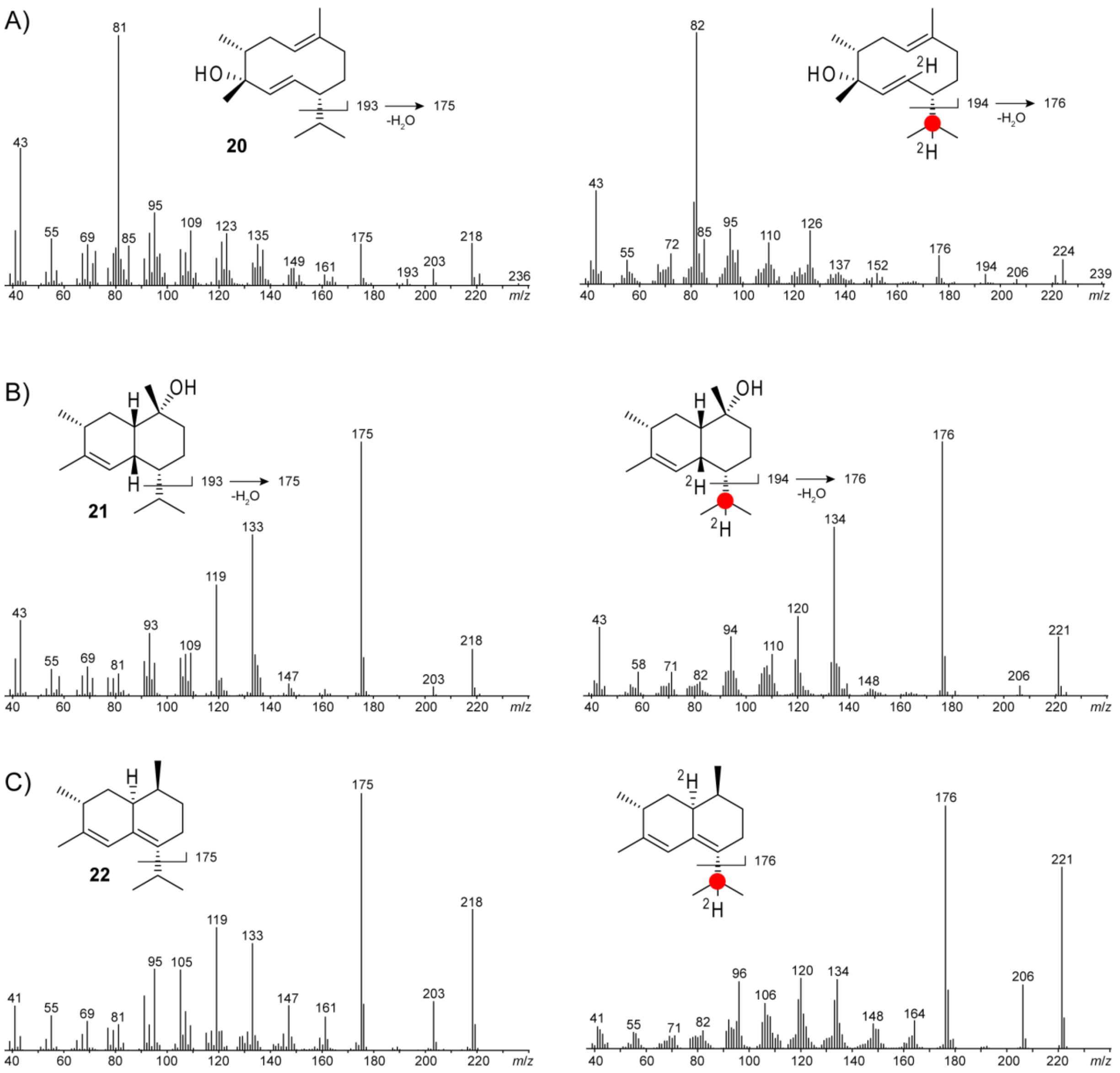

Figure S99. EI mass spectra of A) unlabelled and labelled **20**, B) unlabelled and labelled **21**, and C) unlabelled and labelled **22**. Labelled compounds were obtained from (1,1-$^{2}H_2$)-**8b** and (7-^{13}C)GPP. The fragment ions arising by cleavage of the *i*Pr group and loss of water locate one deuterium with the *i*Pr group.

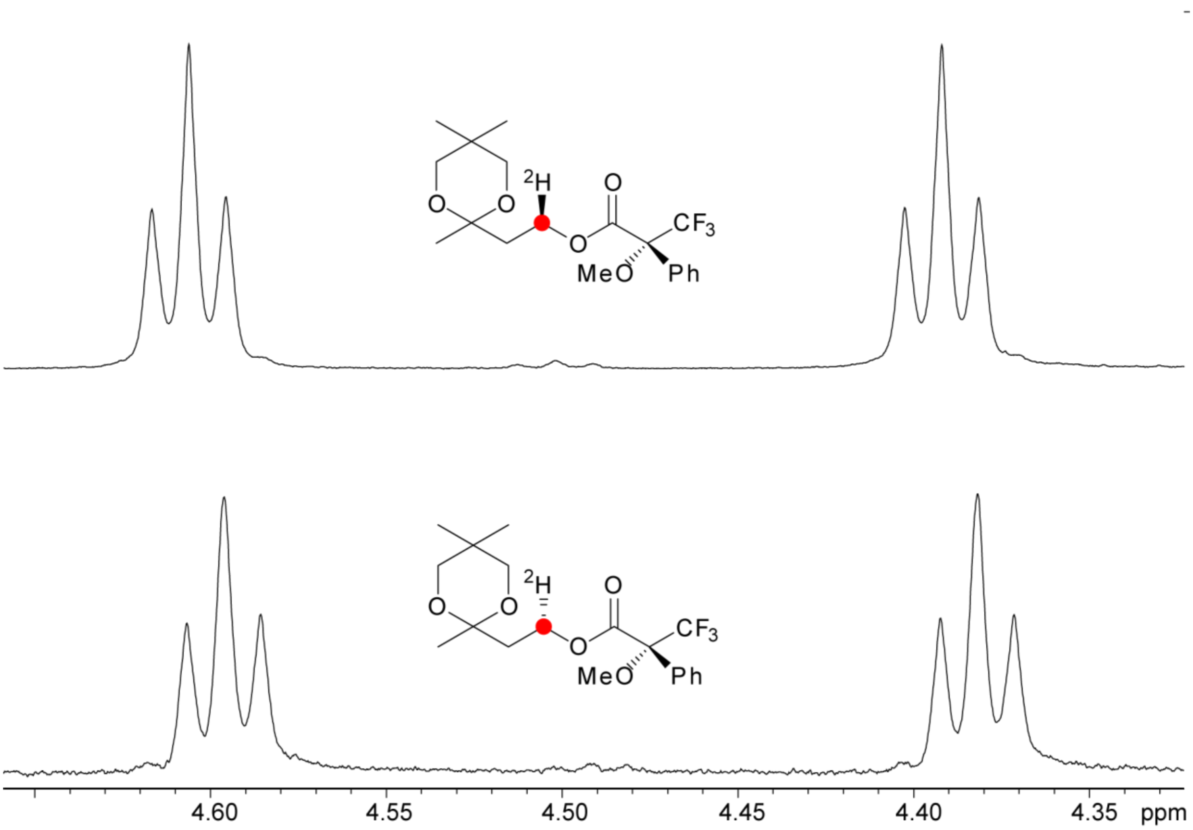

Figure S100. ^{1}H-NMR spectra of Mosher ester derivatives obtained with (*S*)-(+)-MTPA-Cl from A) (*R*)-(1-^{13}C,1-^{2}H)-**S14**, B) (*S*)-(1-^{13}C,1-^{2}H)-**S14**.[12] Characteristic signals at δ = 4.62 ppm and 4.59 pm indicate the diastereomeric composition of each sample. The enantiomeric excesses were determined to be ca. 90% *ee* for (*R*)-(1-^{13}C,1-^{2}H)-**S14** and ca. 94% *ee* for (*S*)-(1-^{13}C,1-^{2}H)-**S14**.

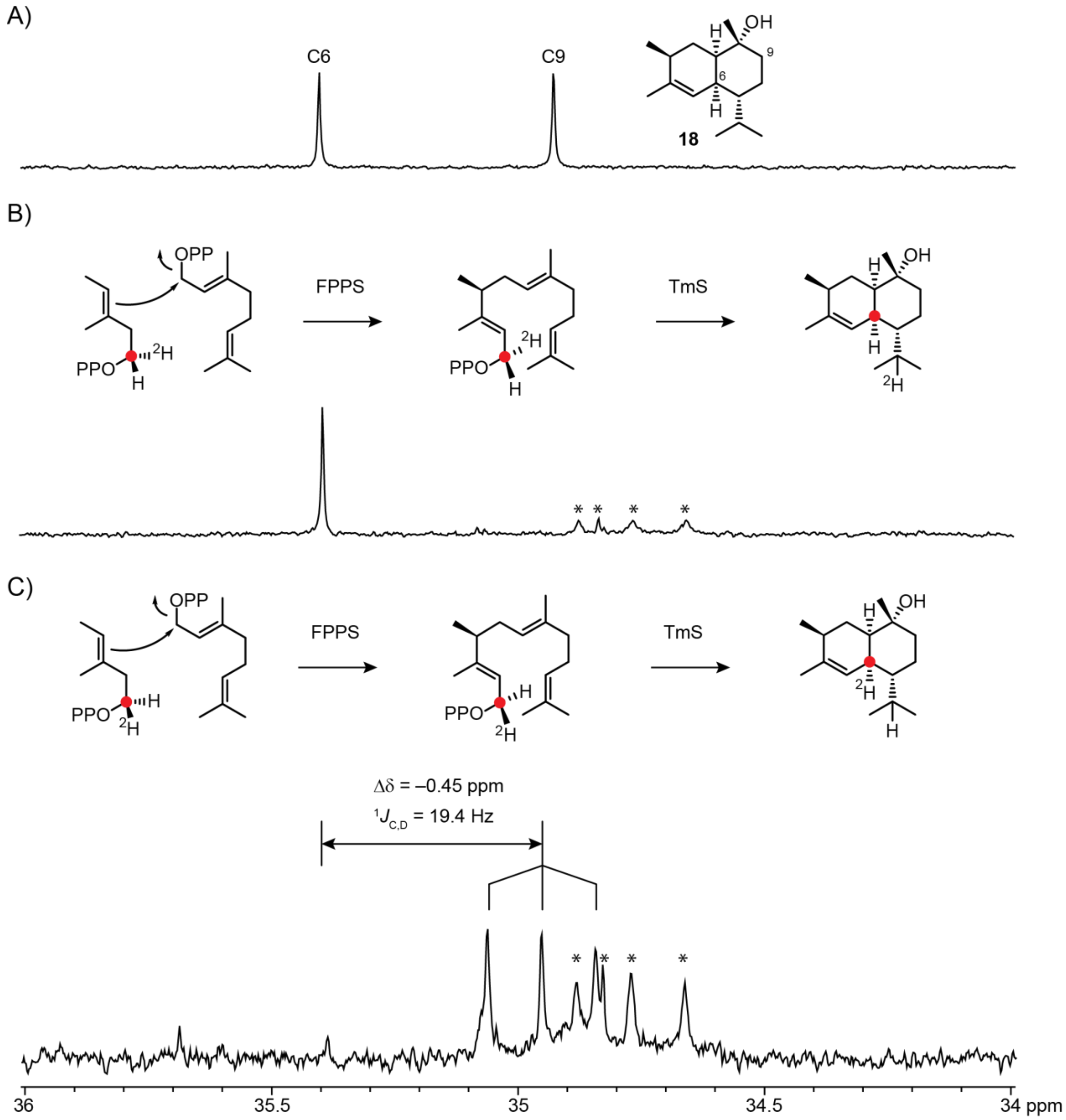

Figure S101. Selective 1,3-hydride migration of the 1-*pro*-*S* hydrogen of **10a** or into the *i*Pr group of **18**. ^{13}C-NMR spectra showing A) the signal for C6 of unlabelled **18**, B) the signal for non-deuterated C6 of labelled **18** obtained from GPP and (*S*)-(1-^{13}C,1-^{2}H)-**8a**, C) the signal for C6 of labelled **18** obtained from GPP and (*R*)-(1-^{13}C,1-^{2}H)-**8a**. The upfield shift (Δδ = –0.45 ppm together with the triplet coupling $^1J_{C,D}$ = 19.4 Hz indicates a direct ^{13}C-^{2}H bond. Asterisks indicate signals of unknown origin.

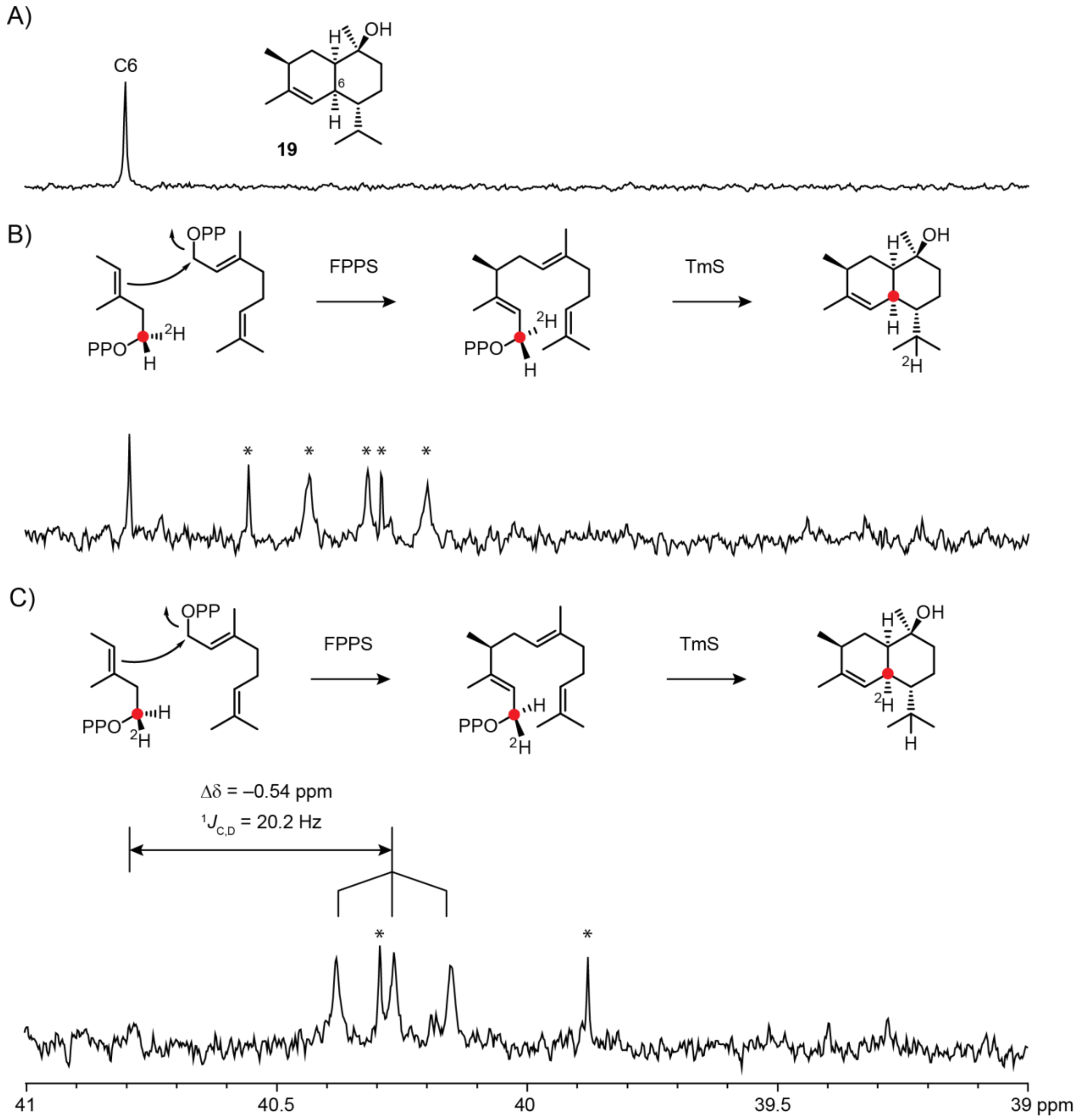

Figure S102. Selective 1,3-hydride migration of the 1-*pro*-*S* hydrogen of **10a** or into the *i*Pr group of **19**. ^{13}C-NMR spectra showing A) the signal for C6 of unlabelled **19**, B) the signal for non-deuterated C6 of labelled **19** obtained from GPP and (*S*)-(1-^{13}C,1-^{2}H)-**8a**, C) the signal for C6 of labelled **19** obtained from GPP and (*R*)-(1-^{13}C,1-^{2}H)-**8a**. The upfield shift (Δδ = –0.54 ppm together with the triplet coupling $^1J_{C,D}$ = 20.2 Hz indicates a direct ^{13}C-^{2}H bond. Asterisks indicate signals of unknown origin.

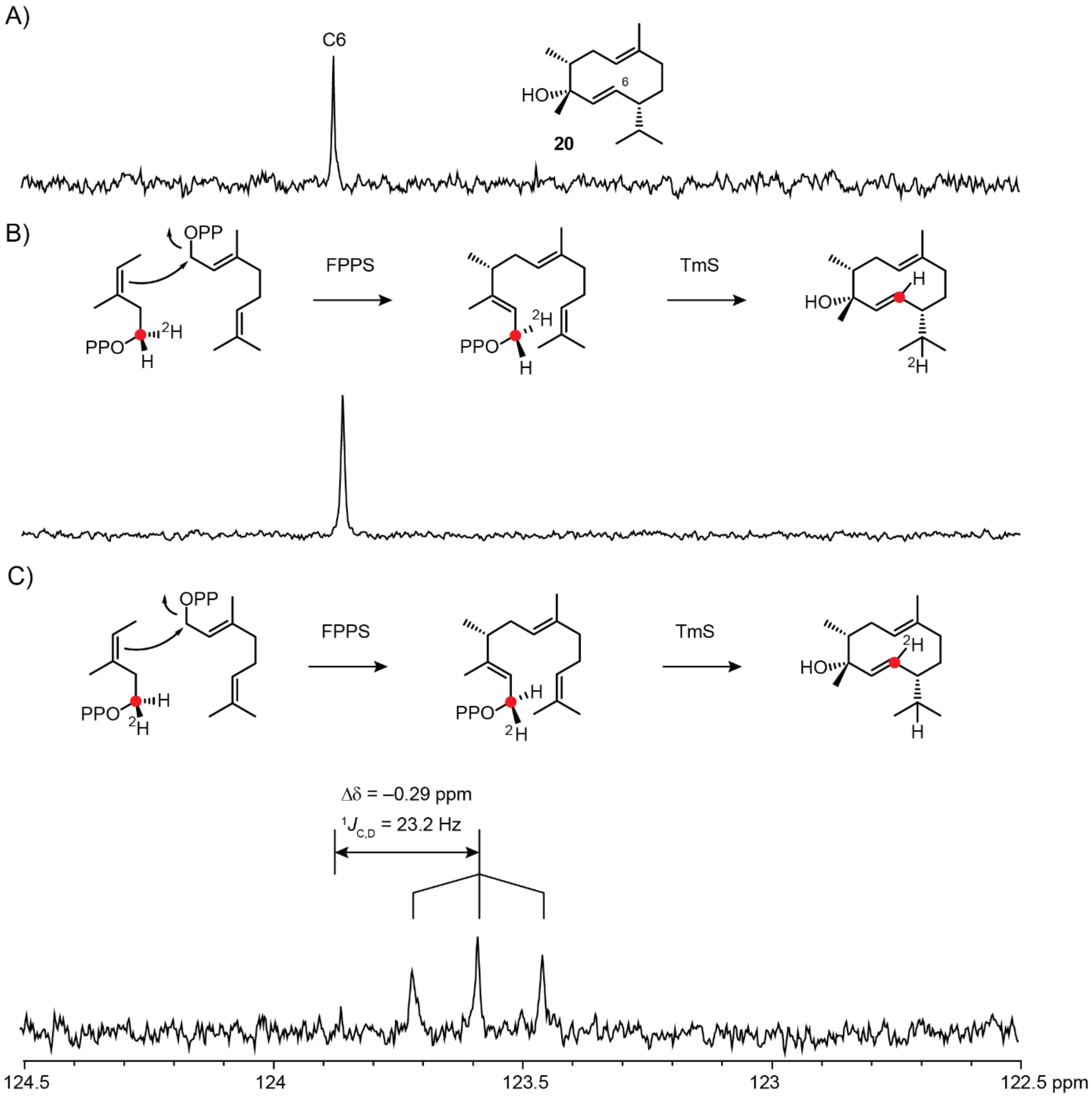

Figure S103. Selective 1,3-hydride migration of the 1-*pro*-*S* hydrogen of **10b** or into the *i*Pr group of **20**. ^{13}C-NMR spectra showing A) the signal for C6 of unlabelled **20**, B) the signal for non-deuterated C6 of labelled **20** obtained from GPP and (*S*)-(1-^{13}C,1-^{2}H)-**8b**, C) the signal for C6 of labelled **20** obtained from GPP and (*R*)-(1-^{13}C,1-^{2}H)-**8b**. The upfield shift ($\Delta\delta$ = –0.29 ppm together with the triplet coupling $^{1}J_{C,D}$ = 23.2 Hz indicates a direct ^{13}C-^{2}H bond.

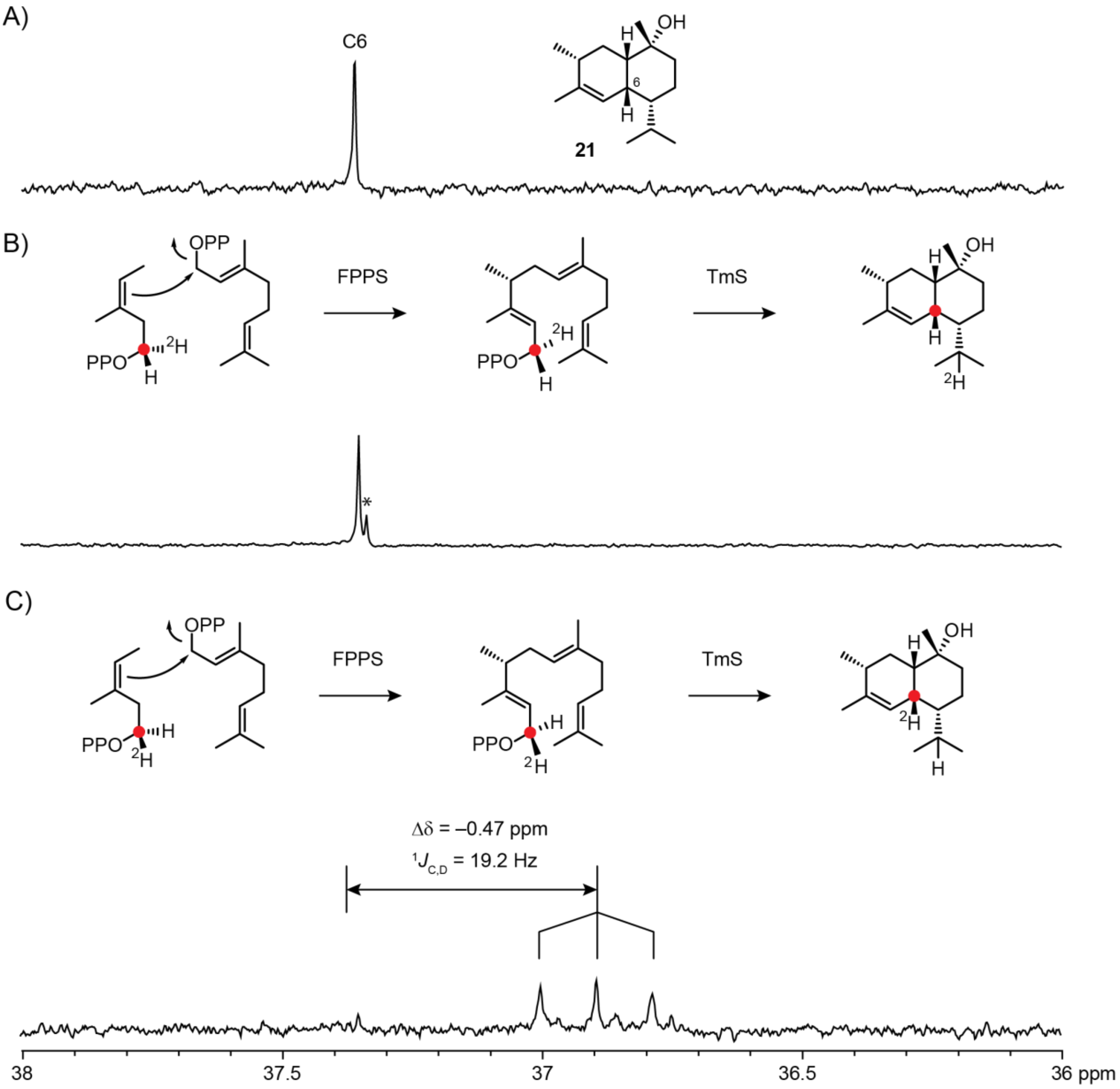

Figure S104. Selective 1,3-hydride migration of the 1-*pro*-*S* hydrogen of **10b** or into the *i*Pr group of **21**. ^{13}C-NMR spectra showing A) the signal for C6 of unlabelled **21**, B) the signal for non-deuterated C6 of labelled **21** obtained from GPP and (*S*)-(1-^{13}C,1-^{2}H)-**8b**, C) the signal for C6 of labelled **21** obtained from GPP and (*R*)-(1-^{13}C,1-^{2}H)-**8b**. The upfield shift ($\Delta\delta$ = –0.47 ppm together with the triplet coupling $^{1}J_{C,D}$ = 19.2 Hz indicates a direct ^{13}C-^{2}H bond. Asterisks indicate signals of unknown origin.

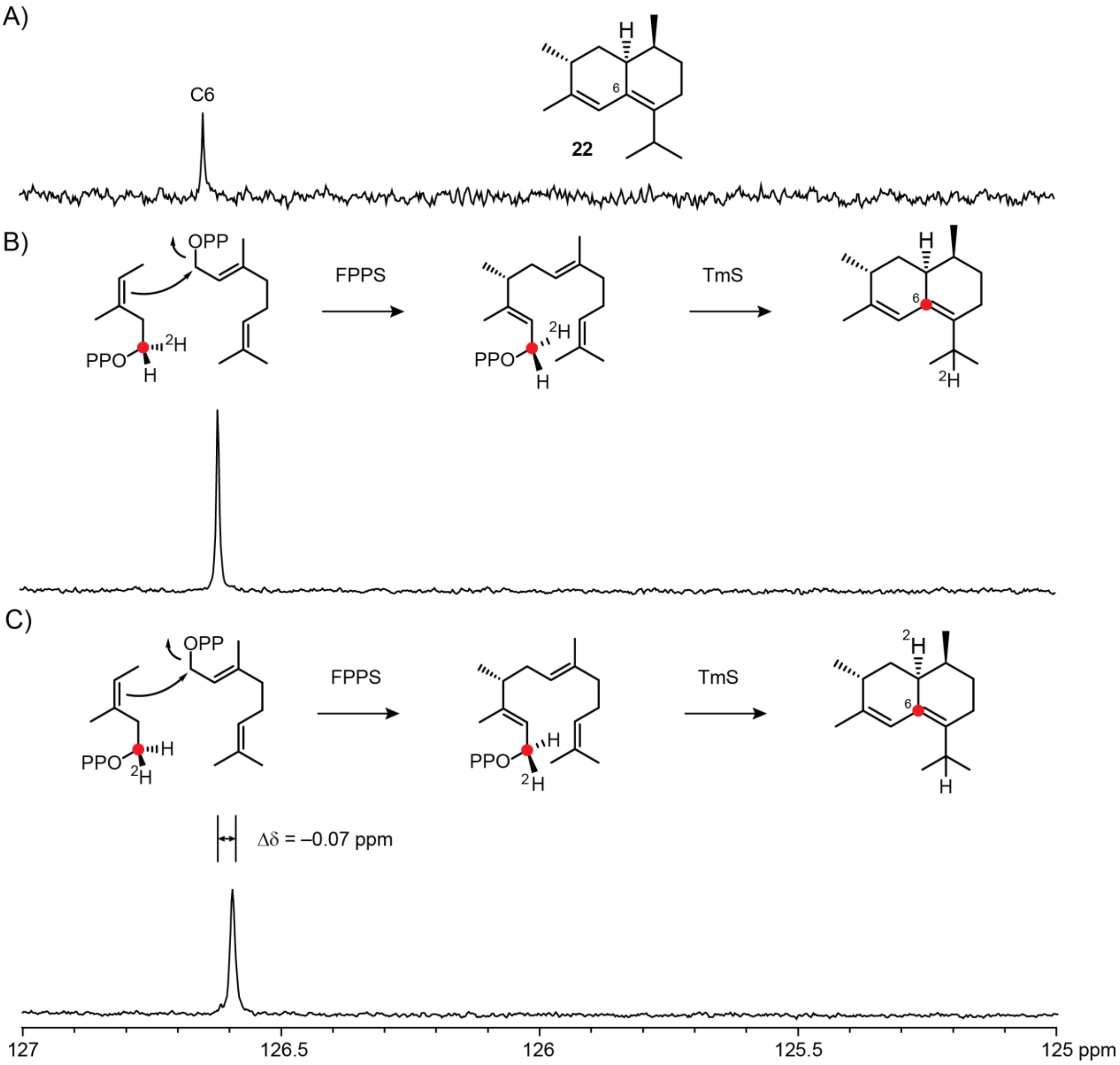

Figure S105. Selective 1,3-hydride migration of the 1-*pro*-*S* hydrogen of **10b** or into the *i*Pr group of **22**. ^{13}C-NMR spectra showing A) the signal for C6 of unlabelled **22**, B) the signal for C6 of labelled **22** obtained from GPP and (*S*)-(1-^{13}C,1-^{2}H)-**8b**, C) the signal for C6 of labelled **22** obtained from GPP and (*R*)-(1-^{13}C,1-^{2}H)-**8b**. The characteristic $\Delta\delta$ = –0.07 ppm for the labelled carbon indicated a deuterium atom located in a neighbouring position (C1).

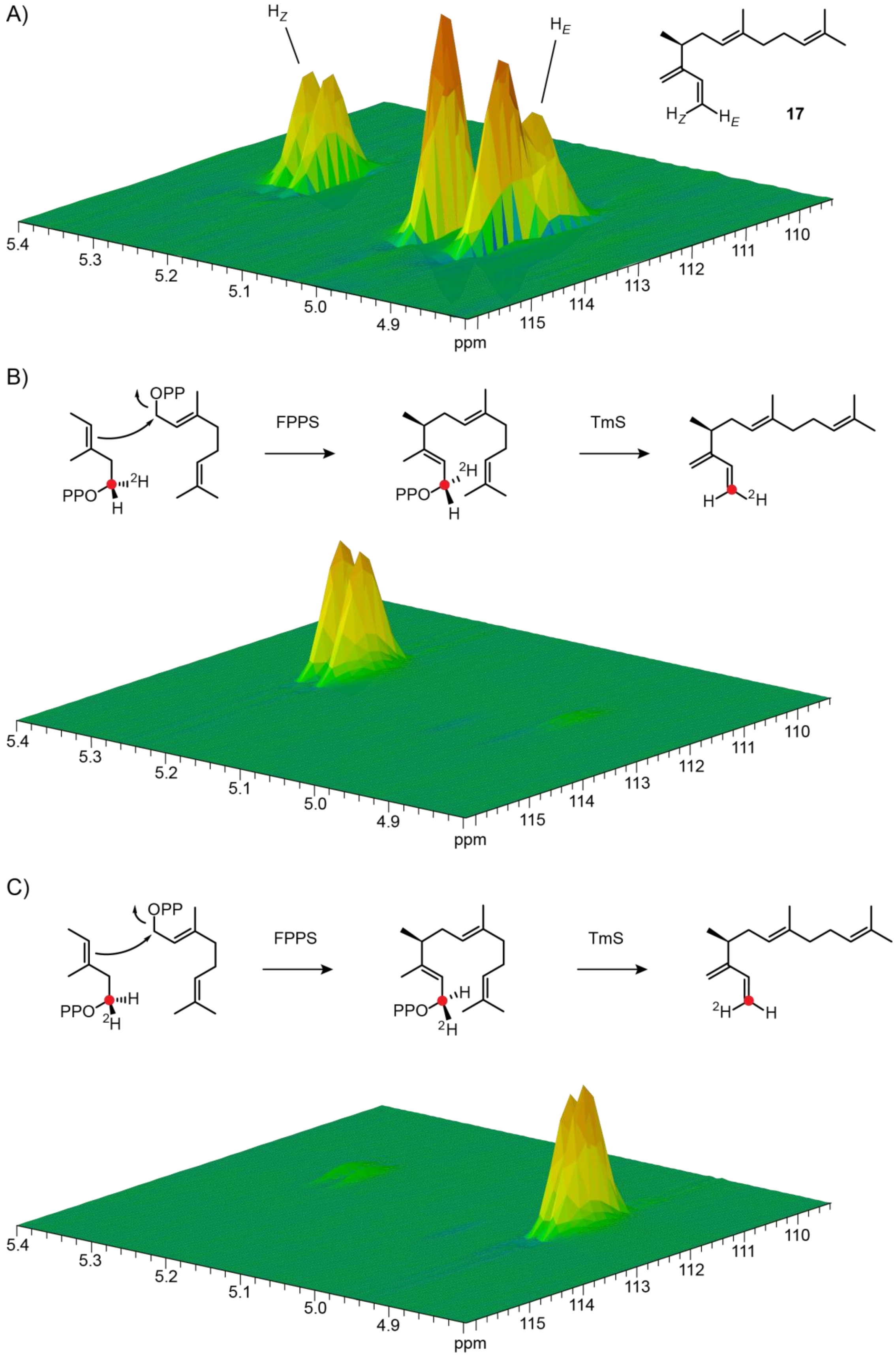

Figure S106. Stereochemcial fate of the C1 hydrogens of **10a** in the TmS catalysed formation of **17**. Partial HSQC spectra of A) unlabelled **17** showing crosspeaks for H1$_Z$ and H1$_E$, B) labelled **17** obtained from GPP and (*R*)-(1-^{13}C,1-^{2}H)-**8a** with FPPS and TmS, and C) labelled **17** obtained from GPP and (*S*)-(1-^{13}C,1-^{2}H)-**8a** with FPPS and TmS. The data demonstrate that the 1-*pro-R* hydrogen ends up in the H1$_Z$ and the 1-*pro-S* hydrogen in the H1$_E$ position.

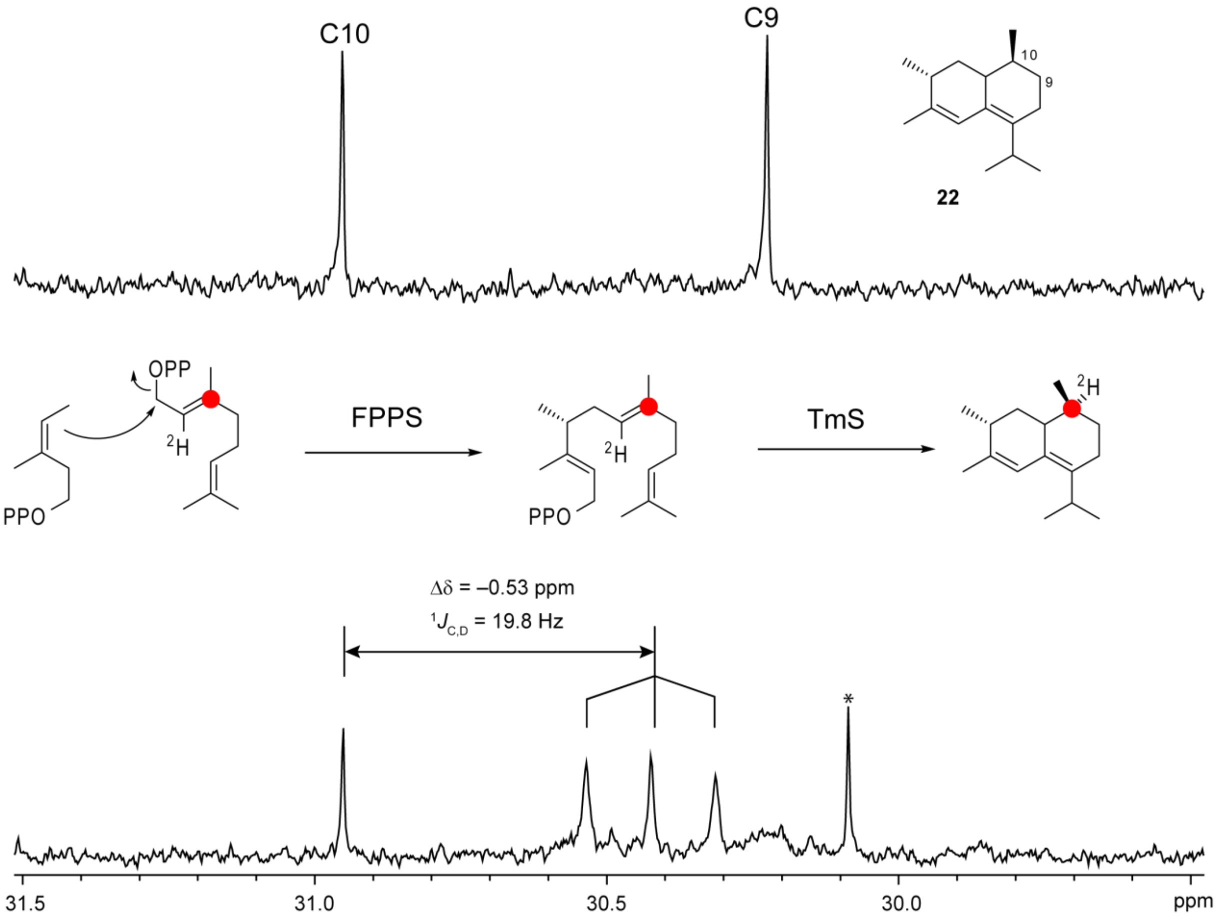

Figure S107. 1,2-Hydride shift in the biosynthesis of **22**. ^{13}C-NMR spectra showing A) the signal for C10 of unlabelled **22**, and B) the signal for C10 of labelled **22** obtained from (3-^{13}C,2-^{2}H)GPP and **8b** with FPPS and TmS. The upfield shifted triplet indicates a direct ^{13}C-^{2}H bond and gives evidence for the proposed 1,2-hydride shift.

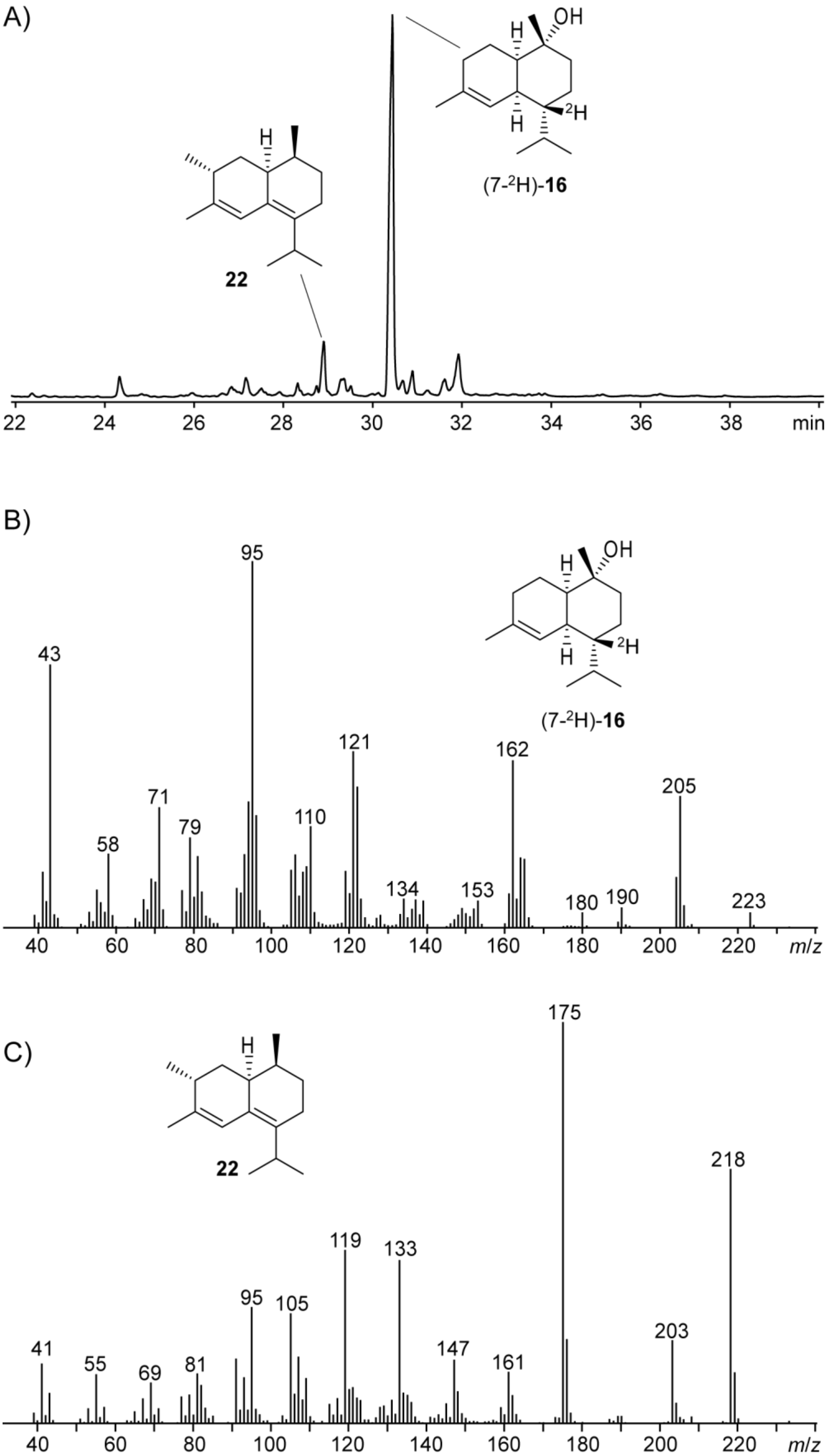

Figure S108. Deprotonation from C7 in the biosynthesis of **22**. A) Total ion chromatogram of the products obtained from (2-^{2}H)DMAPP, IPP and **8b** with FPPS and TmS, B) EI mass spectrum of labelled (7-^{2}H)-**16**, and C) EI mass spectrum of unlabelled **22** obtained in this reaction. The loss of labelling for **22** indicates deprotonation from C7.

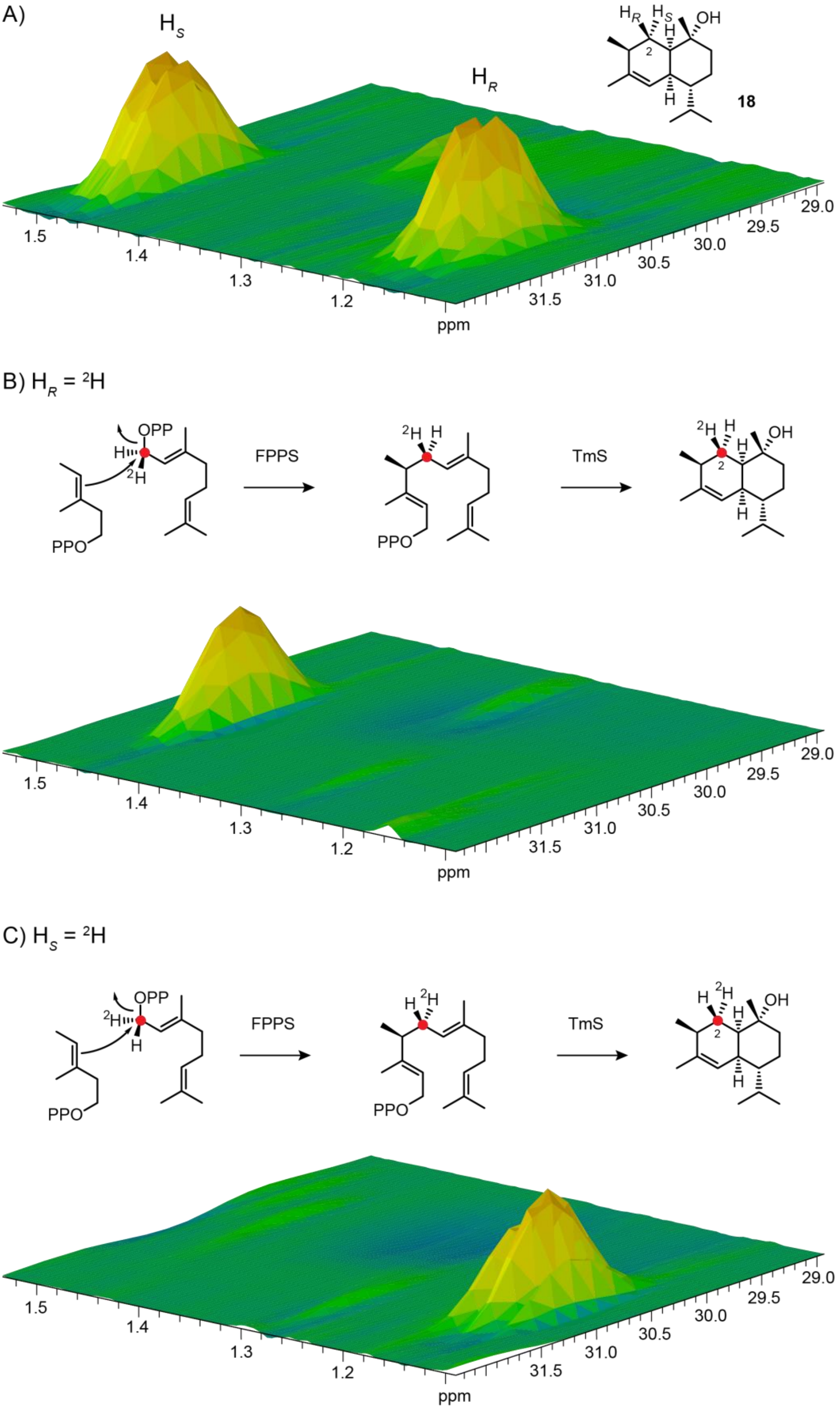

Figure S109. Inversion of configuration at C1 of GPP in the elongation with **8a** by FPPS. Partial HSQC spectra of A) C2 of unlabelled **18**, B) C2 of labelled **18** obtained from (*R*)-(1-^{13}C,1-^{2}H)GPP and **8a** with FPPS and TmS, and C) C2 of labelled **18** obtained from (*S*)-(1-^{13}C,1-^{2}H)GPP and **8a** with FPPS and TmS. Together with NOESY based assignments of H2$_{\alpha}$ and H2$_{\beta}$ these data give evidence for the inversion of configuration at C1 of GPP.

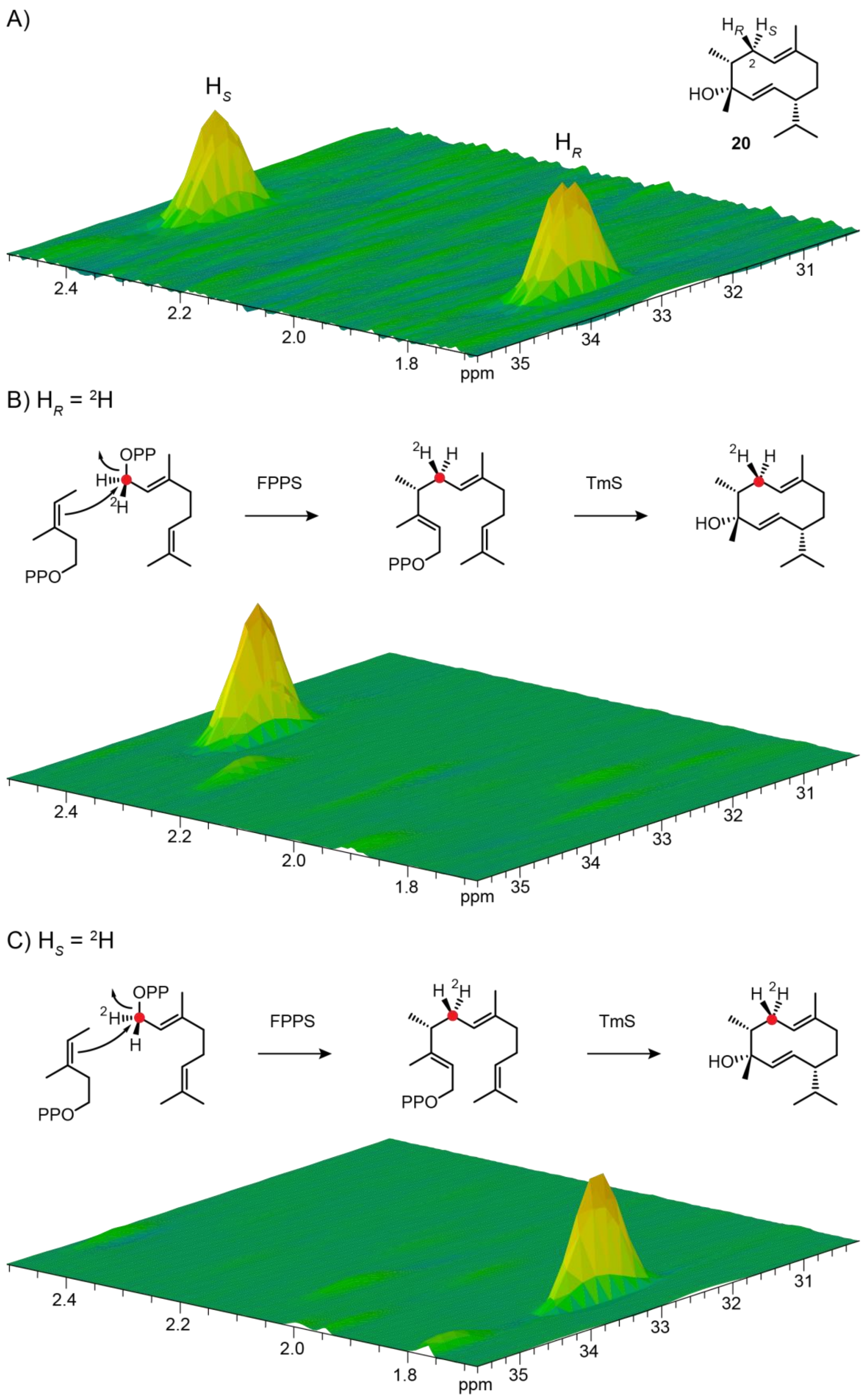

Figure S110. Inversion of configuration at C1 of GPP in the elongation with **8b** by FPPS. Partial HSQC spectra of A) C2 of unlabelled **20**, B) C2 of labelled **20** obtained from (*R*)-(1-^{13}C,1-^{2}H)GPP and **8b** with FPPS and TmS, and C) C2 of labelled **20** obtained from (*S*)-(1-^{13}C,1-^{2}H)GPP and **8b** with FPPS and TmS. Together with NOESY based assignments of $H2_\alpha$ and $H2_\beta$ these data give evidence for the inversion of configuration at C1 of GPP.

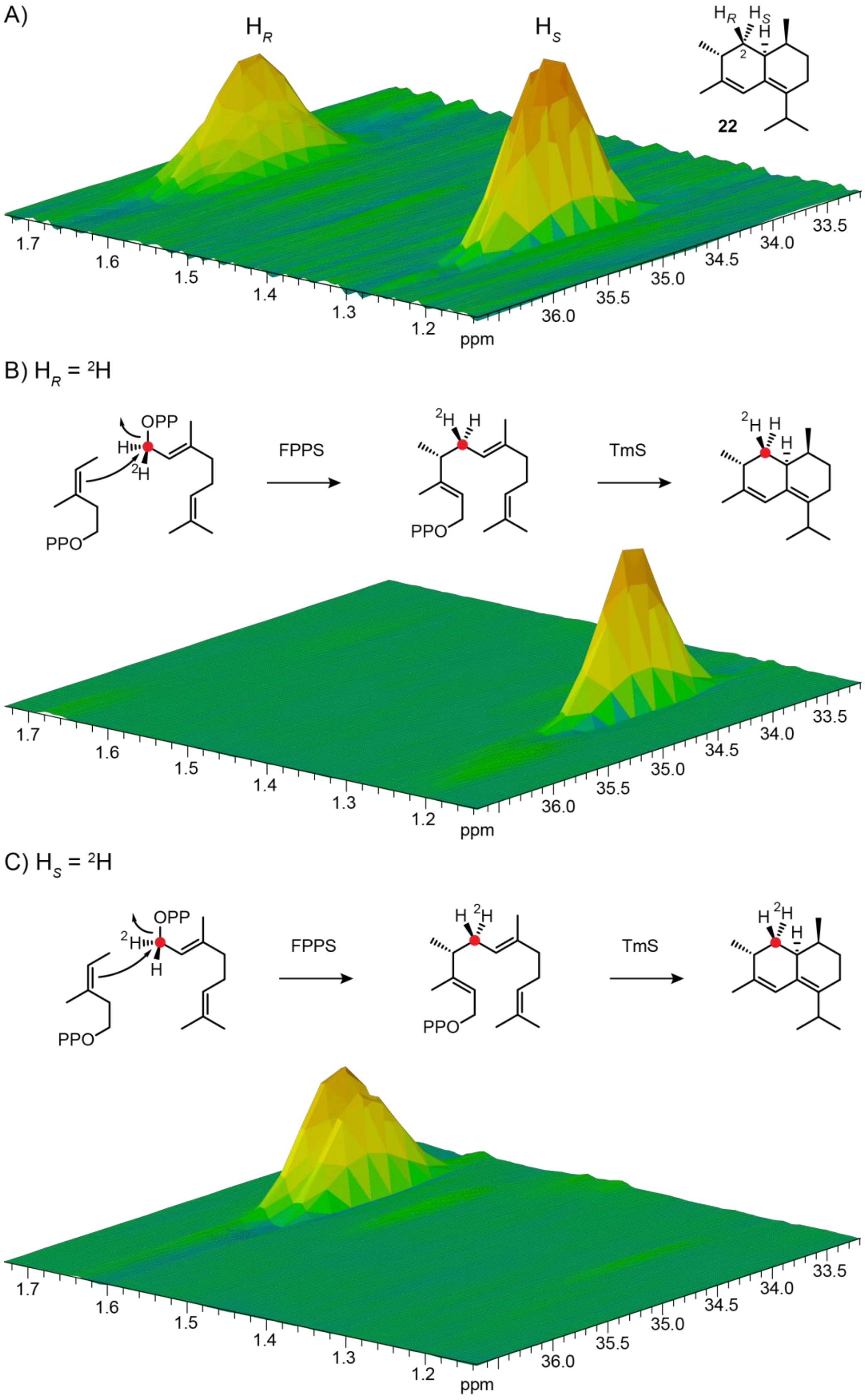

Figure S111. Inversion of configuration at C1 of GPP in the elongation with **8b** by FPPS. Partial HSQC spectra of A) C2 of unlabelled **22**, B) C2 of labelled **22** obtained from (*R*)-(1-^{13}C,1-^{2}H)GPP and **8b** with FPPS and TmS, and C) C2 of labelled **22** obtained from (*S*)-(1-^{13}C,1-^{2}H)GPP and **8b** with FPPS and TmS. Together with NOESY based assignments of H2$_{\alpha}$ and H2$_{\beta}$ these data give evidence for the inversion of configuration at C1 of GPP.

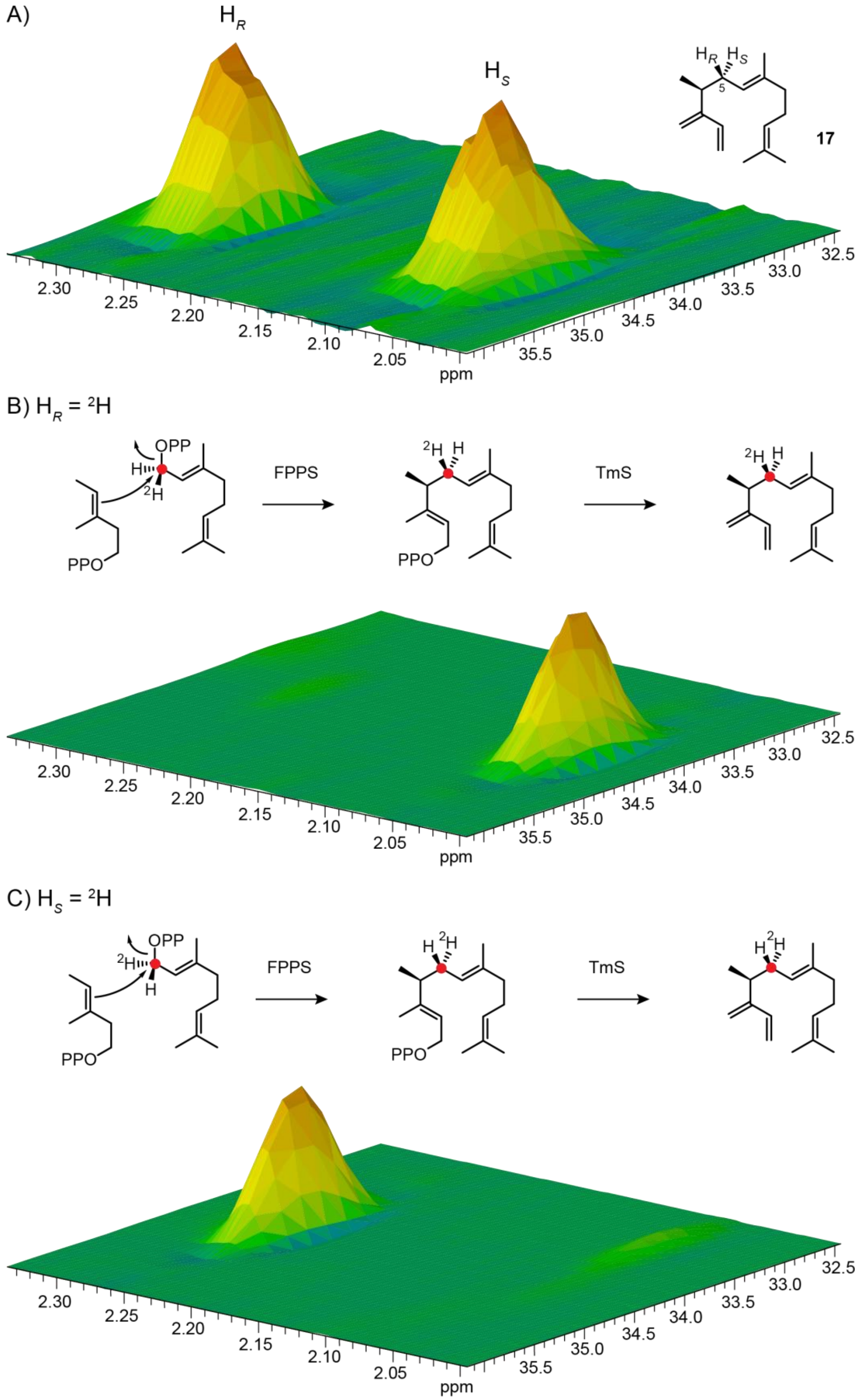

Figure S112. NMR data assignment for the diastereotopic C5 hydrogens of **17** by labelling experiments. HSQC spectra of A) C5 of unlabelled **17**, B) C5 of labelled **17** obtained from (*R*)-(1-^{13}C,1-^{2}H)GPP and **8a** with FPPS and TmS, and C) C5 of labelled **17** obtained from (*S*)-(1-^{13}C,1-^{2}H)GPP and **8a** with FPPS and TmS. Together with the known inversion of configuration at C1 of GPP in the FPPS catalysed elongation with **8a** (Figures S109 – S111) the ^{1}H-NMR chemical shifts for the diastereotopic C5 hydrogens could be assigned.

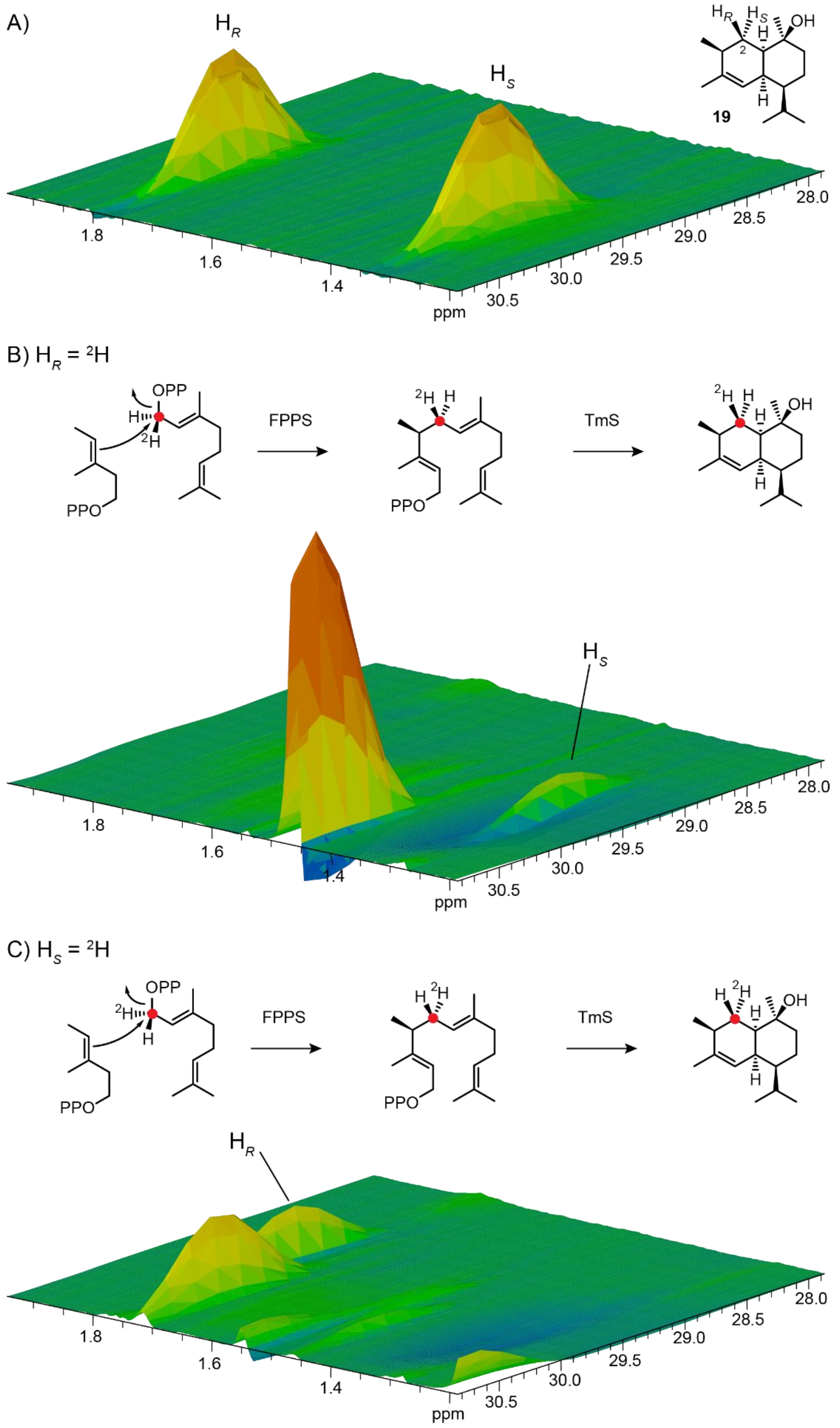

Figure S113. NMR data assignment for the diastereotopic C2 hydrogens of **19** by labelling experiments. HSQC spectra of A) C2 of unlabelled **19**, B) C2 of labelled **19** obtained from (*R*)-(1-^{13}C,1-^{2}H)GPP and **8a** with FPPS and TmS, and C) C2 of labelled **19** obtained from (*S*)-(1-^{13}C,1-^{2}H)GPP and **8a** with FPPS and TmS. Together with the known inversion of configuration at C1 of GPP in the FPPS catalysed elongation with **8a** (Figures S109 – S111) the ^{1}H-NMR chemical shifts for the diastereotopic C2 hydrogens could be assigned.

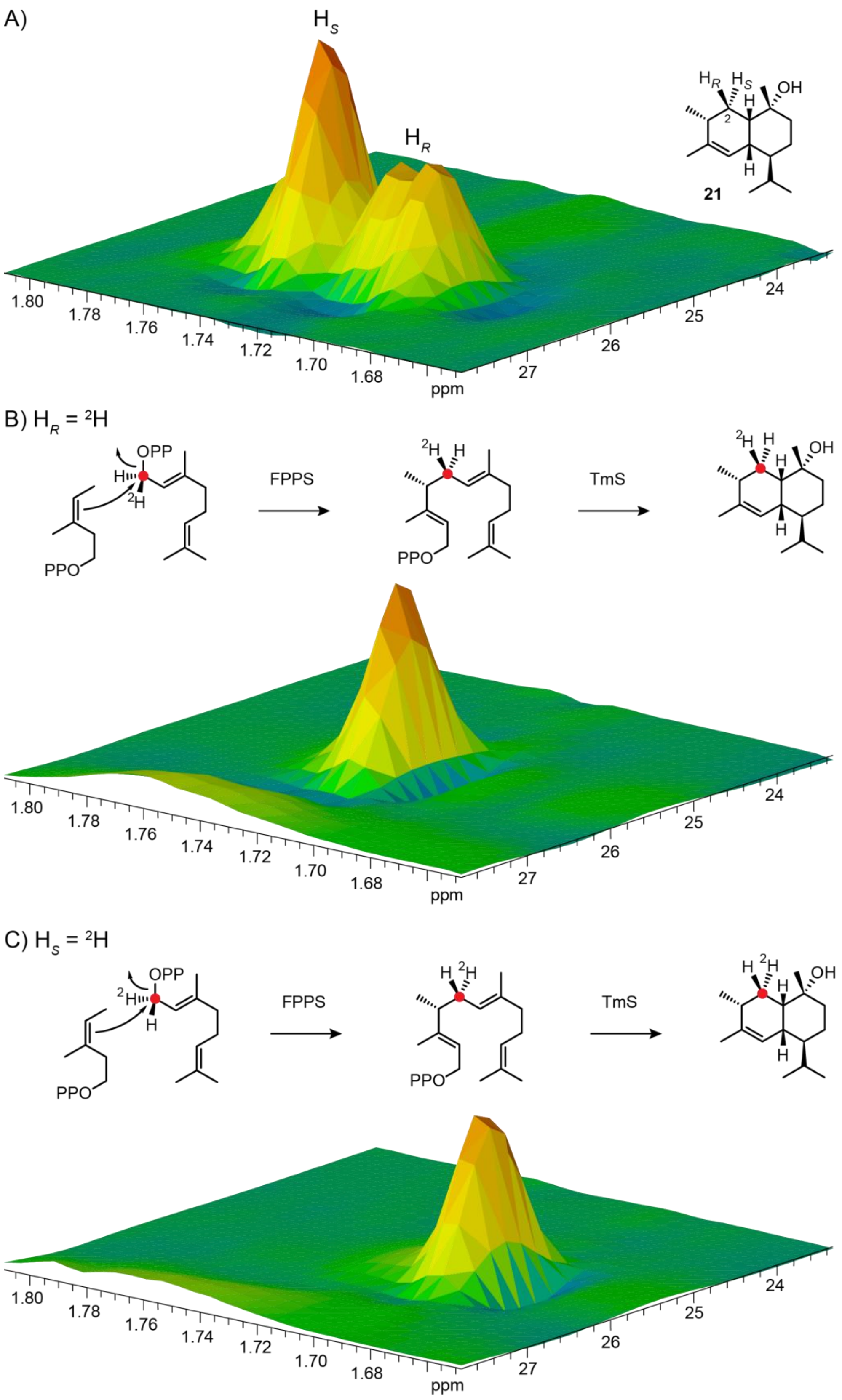

Figure S114. NMR data assignment for the diastereotopic C2 hydrogens of **21** by labelling experiments. HSQC spectra of A) C2 of unlabelled **21**, B) C2 of labelled **21** obtained from (*R*)-(1-^{13}C,1-^{2}H)GPP and **8b** with FPPS and TmS, and C) C2 of labelled **21** obtained from (*S*)-(1-^{13}C,1-^{2}H)GPP and **8b** with FPPS and TmS. Together with the known inversion of configuration at C1 of GPP in the FPPS catalysed elongation with **8a** (Figures S107 – S109) the only slightly different ^{1}H-NMR chemical shifts for the diastereotopic C2 hydrogens could be assigned.

References

[1] C. Xu, Z. Liu, S. Torker, X. Shen, D. Xu, A. H. Hoveyda, *J. Am. Chem. Soc.* **2017**, *139*, 15640.

[2] V. J. Davisson, A. B. Woodside, T. R. Neal, K. E. Stremler, M. Muehlbacher, C. D. Poulter, *J. Org. Chem.* **1986**, *51*, 4768.

[3] P. Rabe, J. Rinkel, B. Nubbemeyer, T. G. Köllner, F. Chen, J. S. Dickschat, *Angew. Chem. Int. Ed*. **2016**, *55*, 15420.

[4] G. R. Fulmer, A. J. M. Miller, N. H. Sherden, H. E. Gottlieb, A. Nudelman, B. M. Stoltz, J. E. Bercaw, K. I. Goldberg, *Organometallics* **2010**, *29*, 2176.

[5] B. Neumann, A. Pospiech, H. U. Schairer, *Trends Genet*. **1992**, *8*, 332.

[6] J. S. Dickschat, K. A. K. Pahirulzaman, P. Rabe, T. A. Klapschinski, *ChemBioChem* **2014**, *15*, 810.

[7] R. D. Giets, R. H. Schiestl, *Nat. Protoc*. **2007**, *2*, 31.

[8] P. Rabe, T. Schmitz, J. S. Dickschat, Beilstein *J. Org. Chem*. **2016**, *12*, 1839.

[9] B. Schmidt, M. Pohler, *Org. Biomo. Chem.* **2003**, *1*, 2512.

[10] G. Schwertz, M. C. Witschel, M. Rottmann, R. Bonnert, U. Leartsakulpanich, P. Chitnumsub, A.Jaruwat, W. Ittarat, A. Schäfer, R. A. Aponte, S. A. Charman, K. White, A. Kundu, S. Sadhukhan, M. Lloyd, G. M. Freiberg, M. Srikumaran, M. Siggel, A. Zwyssig, P. Chaiyen, F. Diederich, *J. Med. Chem.* **2017**, *60*, 12, 4840

[11] Edelstein, R. L.; Weller, V. A.; Distefano, M. D.; Tung, J. S. *J. Org. Chem.* **1998**, *63*, 5298.

[12] Hoye, T. R.; Jeffrey, C. S.; Shao, F. *Nat. Protoc.* **2007**, *2*, 2451

[13] Thulasiram, H. V.; Phan, R. M.; Rivera, S. B.; Poulter, C. D. *J. Org. Chem.* **2006**, *71*, 1739

[14] T. Mitsuhashi, J. Rinkel, M. Okada, I. Abe, J. S. Dickschat, *Chem. Eur. J*. **2017**, *23*, 10053

[15] J. Rinkel, L. Lauterbach, P. Rabe, J. S. Dickschat, *Angew. Chem. Int. Ed*. **2018**, *57*, 3238.

[16] J. Rinkel, P. Rabe, X. Chen, T. G. Köllner, F. Chen, J. S. Dickschat, *Chem. Eur. J.* **2017**, *23*, 10501.

[17] P. Rabe, J. Rinkel, E. Dolja, T. Schmitz, B. Nubbemeyer, T. H. Luu, J. S. Dickschat, *Angew. Chem. Int. Ed*. **2017**, *56*, 2776.
